# Targeting the Exoskeleton Elementome to Track Tick Geographic Origins

**DOI:** 10.3389/fphys.2020.572758

**Published:** 2020-09-23

**Authors:** Iván Pacheco, Pelayo Acevedo, Eduardo Prado, Andrei Daniel Mihalca, José de la Fuente

**Affiliations:** ^1^SaBio, Instituto de Investigación en Recursos Cinegéticos, Consejo Superior de Investigaciones Científicas, Universidad de Castilla-La Mancha, Junta de Comunidades de Castilla-La Mancha, Ciudad Real, Spain; ^2^Escuela Técnica Superior de Ingenieros Agrónomos, Universidad de Castilla-La Mancha, Ciudad Real, Spain; ^3^Department of Applied Physics, Faculty of Chemical Sciences and Technologies, Universidad de Castilla-La Mancha, Ciudad Real, Spain; ^4^Department of Parasitology and Parasitic Diseases, University of Agricultural Sciences and Veterinary Medicine of Cluj-Napoca, Cluj-Napoca, Romania; ^5^Department of Veterinary Pathobiology, Center for Veterinary Health Sciences, Oklahoma State University, Stillwater, OK, United States

**Keywords:** tick, exoskeleton, energy dispersive spectroscopy, elementomics, SEM

## Abstract

Understanding the origin of ticks is essential for evaluating the risk of tick-borne disease introduction into new territories. However, when collecting engorged ticks from a host, it is virtually impossible to identify the geographical location where this tick was acquired. Recently, the elementome of tick exoskeleton was characterized by using scanning electron microscopy (SEM) and energy dispersive spectroscopy analysis (EDS). The objective of our preliminary proof-of-concept study was to evaluate the use of SEM-EDS for the analysis of tick exoskeleton elementome to gain insight into the tick geographic and host origin. For this preliminary analysis we used 10 samples of engorged ticks (larvae and nymphs of six species from three genera) collected from various resident hosts and locations. The elementome of the tick exoskeleton was characterized in dorsal and ventral parts with three scans on each part using an EDS 80 mm^2^ detector at 15 kV in a field emission scanning electron microscope. We used principal component analysis (PCA) (varimax rotation) to reduce the redundancy of data under the premise of losing information as little as possible. The PCA was used to test whether the different variables (tick species, stages, hosts, or geographic locations) differ in the composition of exoskeleton elementome (C, O, P, Cl, and Na). Analyses were carried out using SPSS. The PCA analysis explained a high percentage of variance using the first two factors, C and O (86.13%). The first PC (PC-1; 63.12%) was positively related to P, Cl, and Na, and negatively related to C. The second principal component (23.01%) was mainly positively related to C. In the space defined by the two extracted PC (PC-1 and PC-2), the elementome of tick samples was clearly associated with tick species, but not with developmental stages, hosts or geographic locations. A differentiated elementome pattern was observed within Romanian regions (CJ and TL) for the same tick species. The use of the SEM-EDS methodological approach provided additional information about the tick exoskeleton elementome with possible applications to the identification of tick origin host and location.

## Introduction

Understanding the geographical origin of ticks is essential for evaluating the risk of tick-bornedisease introduction to new territories. In general, the non-parasitic tick stages do not move too much, and the main territorial spread of ticks is by hosts (Ogden et al., 2013). Hence, the distance to which a tick is transported from its environmental origin where it has naturally detached (and eventually collected), highly depends on the movement and migration pattern of the hosts (de la Fuente et al., 2015; Abid et al., 2019). For long distance migrators, such as birds, dispersal can be significant, and the origin of ticks can be very distant. It has been hypothesized for instance that migratory birds, among others, could play a role in the dispersal and range extension of *Ixodes scapularis* in Canada (Ogden et al., 2008) and United States (Tonelli and Dearborn, 2019), of *Ixodes ricinus* in Europe (Sándor et al., 2014; Ciebiera et al., 2019), or *Hyalomma* spp. from northern Africa to Southern and Northern Europe (England et al., 2016), to name only a few. However, when collecting engorged ticks from a host, it is virtually impossible to identify the geographical location where this tick was acquired by the host and suppositions were made based on expected migration routes of birds or by modeling. Although birds carry mainly the immature stages of such ticks, some of them are spending a significant time feeding, which in case of two hosts ticks such as *Hy. marginatum* and *Hy. rufipes* can reach up to 26 days (Hoogstraal, 1979) and allow theoretically even long distance spreading.

A promising tool to track the origin and route of migration in birds, mammals, and insects is the measurement of stable isotopes in the tissues such as feathers, hair, or the chitinous integument, respectively (Hobson and Wassenaar, 2018). However, such methods are difficult to apply due to technological limitations in small organisms such as a larval tick. To overcome these limitations, the characterization of the chemical composition (or elementome) could be an alternative option.

In ticks, as in other terrestrial arthropods, the relative amount of chitin fibrils and protein matrix, protein composition, pH/water content of the matrix, composition of chemical elements, and cross-linking of the matrix protein affect the properties of the exoskeleton (Cribb et al., 2010; Flynn and Kaufman, 2015; Gallant et al., 2016; Gallant and Hochberg, 2017; Suppan et al., 2018). It has been shown that mechanical properties of tick cuticle change during tick feeding to support the increase in body size and mass during this process (Flynn and Kaufman, 2015).

Differences between tick species in the mechanical properties of tick cuticle have been reported (Kaufman and Flynn, 2018). Recently, the elementome of tick exoskeleton, cement and salivary glands were characterized using scanning electron microscopy (SEM) and energy dispersive spectroscopy analysis (EDS; de la Fuente et al., 2020; Villar et al., 2020). Additionally, elemental X-ray microanalysis was performed on ticks to show that hematin granules were derived from the blood meal (Agbede, 1986) and to show that salivary gland secretory granules have high calcium content commensurate with a role in secretory protein packaging (Mans et al., 2001).

**FIGURE 1:**
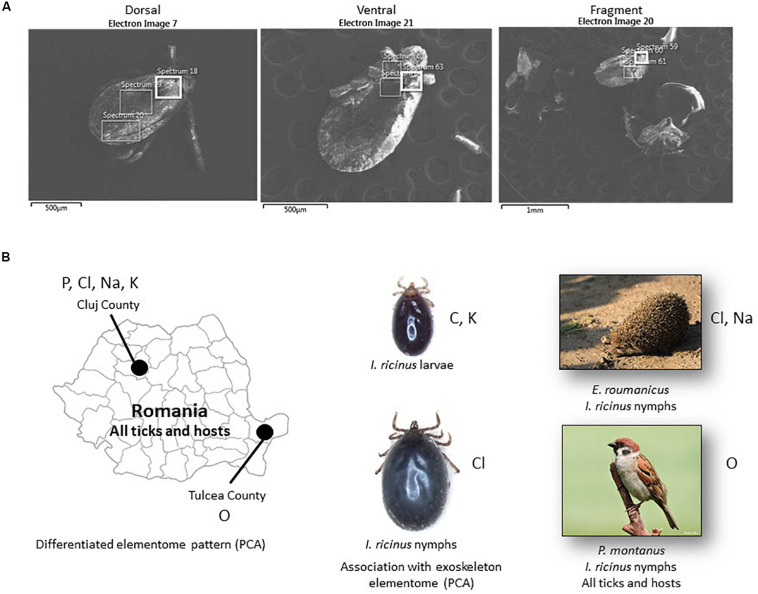
SEM-EDS analysis of tick exoskeleton elementome. **(A)** Representative electron microscopy images used for SEM-EDS analysis of the elementome in tick exoskeleton dorsal, ventral and fragment parts. **(B)** Summary of the results obtained by SEM-EDS analysis of tick exoskeleton elementome. Chemical elements identified with higher levels in the different comparisons and analyses are shown close to each of the geographic locations, tick developmental stages and hosts. Only statistically significant differences are shown (Supplementary Data S1, S3).

The objective of our proof-of-concept study was to evaluate the use of SEM-EDS for the analysis of tick exoskeleton elementome to gain insight into the tick geographic location origin and relation to the host. Herein, the combined SEM-EDS approach was used for the analysis of the exoskeleton elementome in samples collected from different tick species, developmental stages, hosts and geographic locations (Supplementary Data S1, S2). The elementome was characterized in tick exoskeleton dorsal, ventral and fragment parts (Figure 1A). Finally, the PCA was used to test whether the different variables (tick species, stages, hosts, or geographic locations) differ in the composition of exoskeleton elementome. The preliminary results showed that the SEM-EDS methodological approach provides information about the tick exoskeleton elementome with putative implications in the tracking of tick origin.

## Materials and Methods

### Ticks

For this study, 10 randomly selected samples of engorged ticks (larvae and nymphs of six species from three genera) collected from various resident hosts and locations were used (Table 1 and Supplementary Data S1). Ticks were identified based on morphological keys by Estrada-Peña et al. (2018). Ticks were preserved in absolute ethanol before individual examination.

**TABLE 1:** Analysis of tick exoskeleton elementome by SEM-EDS.

Sample No.	Tick species	Stage	Host	Location	Number of spectra	Chemical elements relative abundance				
						C	O	P	Cl	Na
1	*I. ricinus*	L	*Erinaceus roumanicus*	Cluj, Romania	9	75.4 ± 2.1^a^	16.7 ± 2.7	0.5 ± 0.2	3.0 ± 1.2^a^	3.9 ± 1.6
2	*I. ricinus*	N	*Erinaceus roumanicus*	Cluj, Romania	4	70.2 ± 4.2^a^	14.8 ± 1.5^b^	0.5 ± 0.2	5.7 ± 2.1^a,b^	6.3 ± 2.4^b^
3	*I. ricinus*	N	*Passer montanus*	Tulcea, Romania	4	70.3 ± 5.8	19.8 ± 3.8^b^	0.3 ± 0.2	2.3 ± 1.8^b^	3.3 ± 1.8^b^
4	*R. sanguineus*	L	*Erinaceus roumanicus*	Tulcea, Romania	9	66.7 ± 6.3	25.5 ± 2.7	0.1 ± 0.0	0.2 ± 0.1	0.7 ± 0.3
5	*H. punctata*	L	*Passer domesticus*	Bucharest, Romania	9	73.1 ± 1.6	26.4 ± 1.7^c,k^	0.1 ± 0.0	0.3 ± 0.2^c,k^	0.3 ± 0.12^c,k^
6	*H. sulcata*	N	*Martes foina*	Tulcea, Romania	9	75.1 ± 5.2	18.1 ± 1.9^c,k^	0.2 ± 0.1	1,7 ± 0.6^c,k^	2.2 ± 0.82^c,k^
7	*R. sanguineus*	N	*Canis familiaris*	Mount Kulal, Kenya	10	78.0 ± 2.2^d,l^	20.0 ± 1.8^d,l^	0.1 ± 0.0	0.4 ± 0.2	1.1 ± 0.2
8	*R. pulchellus*	N	*Equus asinus*	Lamu, Kenya	9	71.2 ± 4.8^d,l^	22.3 ± 1.2^d,l^	0.1 ± 0.0	0.4 ± 0.2	1.1 ± 0.3
9	*R. annulatus*	N	*Bos taurus*	Asfour, Algeria	9	79.3 ± 1.4^e^	18.8 ± 1.4^e^	0.1 ± 0.0	0.5 ± 0.2^e^	1.3 ± 0.4^e^
10	*R. annulatus*	L	*Bos taurus*	Asfour, Algeria	9	71.4 ± 5.4^e^	25.7 ± 1.3^e^	0.1 ± 0.0	0.3 ± 0.1^e^	0.5 ± 0.3^e^

*The relative abundance of chemical elements (average ± SD percent of total atoms in the sample) were compared between groups by Student’s *t*-test with unequal variance (a,b,c,d,e superindexes for significant difference, *p* < 0.05, *n* = 4–9 biological replicates) and one-way ANOVA test (https://www.socscistatistics.com/tests/anova/default2.aspx) when the number of spectra was higher than 5 (k,l superindexes for significant difference, *p* < 0.05, *n* = 6–10 biological replicates). Only chemical elements identified in all samples were included (Data S1). L, larva; N, nymph.*

### Scanning Electron Microscopy and Energy Dispersive Spectroscopy Analysis

Whole ticks were dehydrated in an incubator at 37°C for 24 h as a preparation for SEM photography. Specimens were mounted onto standard aluminum SEM stubs using conductive carbon adhesive tabs. Ticks were observed and photographed with a field emission scanning electron microscope (Zeiss GeminiSEM 500, Oberkochen, Germany) operating in high vacuum mode at an accelerating voltage of 2 kV and with no metallic coating. The elementome of the tick exoskeleton was characterized in dorsal and ventral parts (Figure 1A). Some tick specimens fractionated during preparation for analysis and were included as tick fragments (Figure 1A). The elementome was determined with three scans on each part using an EDS 80 mm^2^ detector at 15 kV (Oxford Instruments, Abingdon, United Kingdom). The relative abundance of chemical elements (percent of total atoms in the sample) were compared between groups by Student’s *t*-test with unequal variance and one-way ANOVA test^1^ when the number of spectra was higher than 5 (*p* < 0.05, *n* = 6–10 biological replicates) (Supplementary Data S1, S2).

### Principal Component Analysis

The principal component analysis (PCA) is a dimension reduction technique for data analysis (Jolliffe and Cadima, 2016). Principal component analysis maps *n*-dimensional features to *k*-dimensional features (*k* ≤ *n*). The *k*-dimensional features are new orthogonal factors, called principal components (PC), which are reconstructed from the original *n*-dimensional features. The essence of the PCA is to reduce the redundancy of data under the premise of losing information as little as possible. In this study, we used PCA (varimax rotation) to analyze the chemical composition of the tick exoskeleton using chemical elements identified in all samples (C, O, P, Cl, and Na). The objective was to test whether the different tick species, stages, hosts or origin geographic locations differ in the exoskeleton elementome characterized with the PC. Analyses were carried out using SPSS (IBM SPSS Statistics for Windows, Version 25.0. IBM Corp. Armonk, NY; released 2017).

## Results and Discussion

### Components of the Exoskeleton Elementome Show Differences Between Tick Developmental Stages, Hosts and Geographic Locations

As in previous studies (Kaufman and Flynn, 2018; de la Fuente et al., 2020), the results of the analysis of tick exoskeleton elementome showed the presence with high relative abundance (>10 atomic %) of C, O, and N in tick samples (Supplementary Data S1, S2). Other chemical elements with low relative abundance (<10 atomic %) in tick samples included S, P, Cl, Na, K, Ca, Br, Si, Mo, and Mg (Supplementary Data S1, S2).

Despite the limited number of samples included in the analysis, matched-pair analyses showed some differences in the exoskeleton composition of some chemical elements between tick developmental stages (C, Cl, and K in *I. ricinus* larvae and nymphs; *p* < 0.05, Supplementary Data S1 reference 1 vs. 2) and hosts (O, Cl, Na in *I. ricinus* from *Erinaceus roumanicus* and *Passer montanus*; *p* < 0.05, Supplementary Data S1 reference 2 vs. 3) (Table 1 and Figure 1B). Other analyses by grouping all tick samples for comparison between different geographic locations and hosts showed differences between Romanian CJ and TL counties (O, P, Cl, Na, K; *p* < 0.005, Supplementary Data S1 reference 1,2 vs. 3,4,6) and between *E. roumanicus* and *P. montanus* hosts (O; *p* = 0.01, Supplementary Data S1 reference 1,2,4 vs. 3,5) (Table 1 and Figure 1B).

Some of these results may have functional implications. Our results for *I. ricinus* showed that Cl increased and C decreased in relative abundance in nymphs when compared to larvae (Figure 1B). Nymphs have a larger blood meal than larvae, which correlates with previous findings that Cl relative abundance increases with feeding in both salivary glands and cement while the main source for some of the elementome chemical elements such as C are tick and host derived proteins (de la Fuente et al., 2020). It has been demonstrated that the blood of mammals and birds living at higher altitudes show an increased O affinity which improves the O transport when compared between individuals of the same species but living at different elevations (Snyder, 1992). This could explain the difference between Cluj (484 m) and Tulcea (57 m). However, this variability can also be due to differences in metabolic activity or cross-linking mediated by O.

These preliminary analyses showed significant differences in the abundance of some exoskeleton chemical elements between tick developmental stages, hosts and geographic locations (Figure 1B). However, in most cases the matched-pair analyses were not conclusive due to multiple and/or unknown variables. Therefore, the only chemical elements present in samples from all geographic locations (C, O, P, Cl, and Na) were then used for PCA.

### Principal Component Analyses Showed That the Differences in the Exoskeleton Elementome Are Mainly Associated With Tick Species

The PCA analysis explained a high percentage of variance using the first two factors, C and O (86.13%; Supplementary Data S3). The first PC (PC-1; 63.12%) was positively related to P, Cl, and Na, and negatively related to C (Table 2). The second principal component (23.01%; Supplementary Data S3) was mainly positively related to C (Table 2).

**TABLE 2:** Factor loading of the chemical elements in the principal components.

Chemical elements	PC-1	PC-2
C	0.107	0.969
O	–0.823	–0.376
P	0.807	–0.130
Cl	0.964	–0.147
Na	0.942	–0.176

*The larger the absolute value of the loading, the more important the chemical element is in calculating each component; the relationship between the element and the component can be positive or negative.*

**FIGURE 2:**
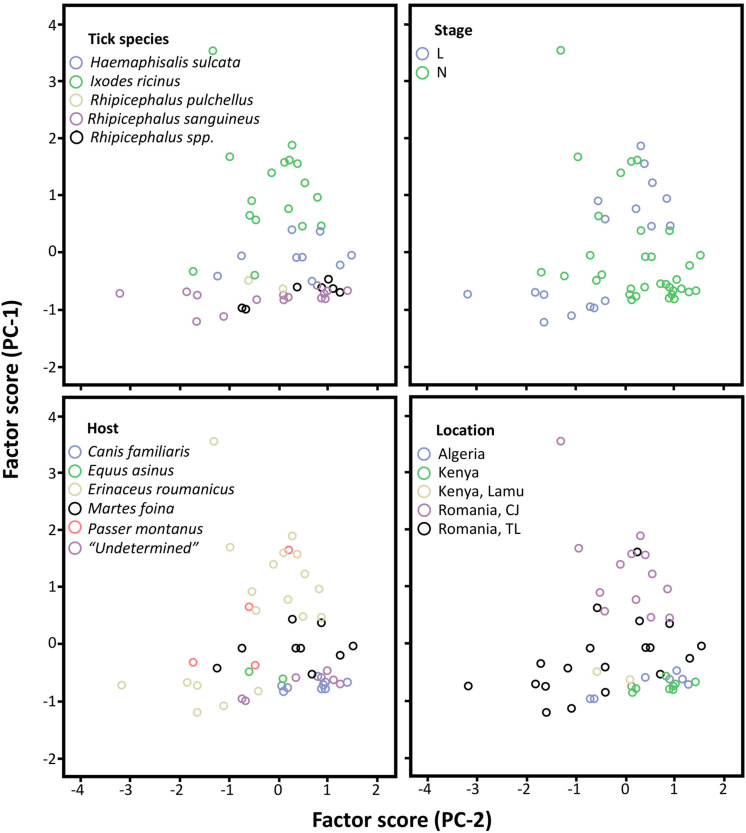
Principal component analyses of tick exoskeleton elementome. Representation of the samples by tick species, developmental stage (L and N), host and geographic location in relation to the two axes extracted from the principal component analyses (PC-1 and PC-2) that are related with the chemical composition of the tick exoskeleton elementome. This plot allows to visually cluster the samples in relation to the chemical space defined by PC-1 and PC-2. Higher clustering along PC-1 suggests that samples are grouped by differences in P, Cl, Na, and O, and along PC-2 that are mainly grouped by C (see Table 2). The category “Undetermined” in Host analysis refers to ticks with unknown host origin (Data S1 and S3). L, larvae; N, nymphs.

In the space defined by the two extracted PC (PC-1 and PC-2), the elementome of tick samples was clearly associated with tick species, but not with developmental stages, hosts or geographic locations (Figure 2). Nevertheless, as shown with matched-pair analyses (Figure 1B), within Romanian regions (CJ and TL), a clearly differentiated elementome pattern was observed (Figure 2).

## Conclusion

The use of the SEM-EDS methodological approach provided additional information about the tick exoskeleton elementome and showed variations in the abundance of chemical elements. These variations were associated with tick developmental stages, hosts and geographic locations (Figure 1B). However, the PCA analysis showed that the exoskeleton elementome was associated with tick species but not with developmental stages, hosts or geographic locations. Although preliminary, these results together with previous reports (de la Fuente et al., 2020; Villar et al., 2020) support the use of SEM-EDS for the characterization of tick elementome in different organs and biomolecular complex structures such as cement and exoskeleton with possible applications to the identification of tick origin host and location.

Supported by this proof-of-concept study, future experiments should characterize tick exoskeleton elementome with a higher number of samples including laboratory-rated controls and combining both matched-pair and PCA analyses to get insight into tick origins.

## Data Availability Statement

All datasets presented in this study are included in the article/Supplementary Material.

## Ethics Statement

Ethical review and approval was not required for the animal study because collection of ticks from vertebrates does not fall under the regulations of ethical permission, as all hosts were released and remained unharmed after the ticks were collected. The tick removal is not a painful procedure.

## Author Contributions

IP, PA, and EP performed the analysis and wrote the draft of the manuscript. AM collected the tick and developed the ideas of the work. JF coordinated the lab work. All authors contributed to the writing of the manuscript.

## Conflict of Interest

The authors declare that the research was conducted in the absence of any commercial or financial relationships that could be construed as a potential conflict of interest. The reviewer BM declared a past co-authorship with one of the authors JF to the handling editor.

## Footnotes

1 https://www.socscistatistics.com/tests/anova/default2.aspx

## Supplementary Material

DATA S1 Tick exoskeleton elementome analysis by SEM-EDS.

DATA S2 Quantitative spectra of chemical elements of tick exoskeleton.

DATA S3 Results of the Principal Component Analysis (PCA).
