# Supplementary material for: Targeting the Exoskeleton Elementome to Track Tick Geographic Origins
Source: Front Physiol. 2020 Sep 23;11:572758. doi: 10.3389/fphys.2020.572758 (PMC7538837; doi:10.3389/fphys.2020.572758)

22/01/2020 10:25:33
Project 1
Rumania 1
Dorso 1 15 Kv

Electron Image 1

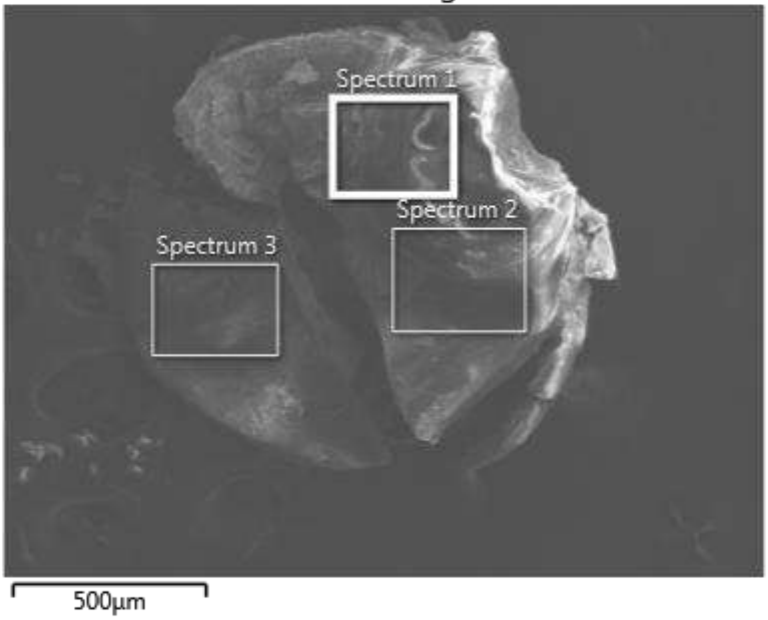

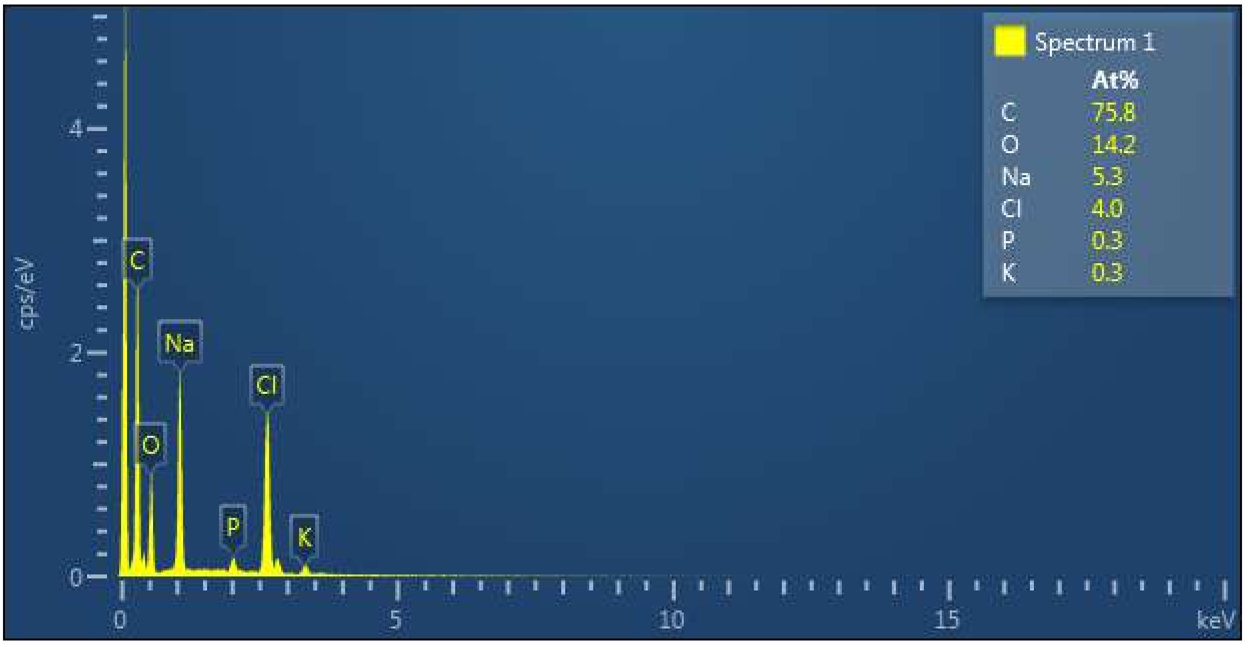

Label:	Spectrum 1
Source:	Acquired
Created:	22/01/2020 10:25:33
Livetime:	60.5s
Process Time:	4
Accelerating Voltage:	15.00kV
Magnification:	57 x
Working Distance:	10.0mm
Specimen Tilt (degrees):	0.0
Elevation (degrees):	35.0
Azimuth (degrees):	0.0
Number Of Channels:	2048
Energy Range (keV):	20 keV
Energy per Channel (eV):	10.0eV
Detector Type Id:	29
Detector Type:	X-Max
Window Type:	SATW
Pulse Pile Up Correction:	Succeeded
Primary Detector:	2617
Primary Detector Serial Number:	77871-X080

Element	Line Type	Apparent Concentration	k Ratio	Wt%	Wt% Sigma	Atomic %	Standard Label	Factory Standard	Standard Calibration Date
C	K series	4.53	0.04533	63.98	0.72	75.84	C Vit	Yes	
O	K series	2.53	0.00852	16.01	0.49	14.25	SiO2	Yes	
Na	K	3.04	0.0128	8.54	0.21	5.29	Albite	Yes	

	series		1						
P	K series	0.27	0.00149	0.67	0.06	0.31	GaP	Yes	
Cl	K series	2.61	0.02282	10.02	0.24	4.03	NaCl	Yes	
K	K series	0.21	0.00174	0.78	0.07	0.28	KBr	Yes	
Total:				100.00		100.00			

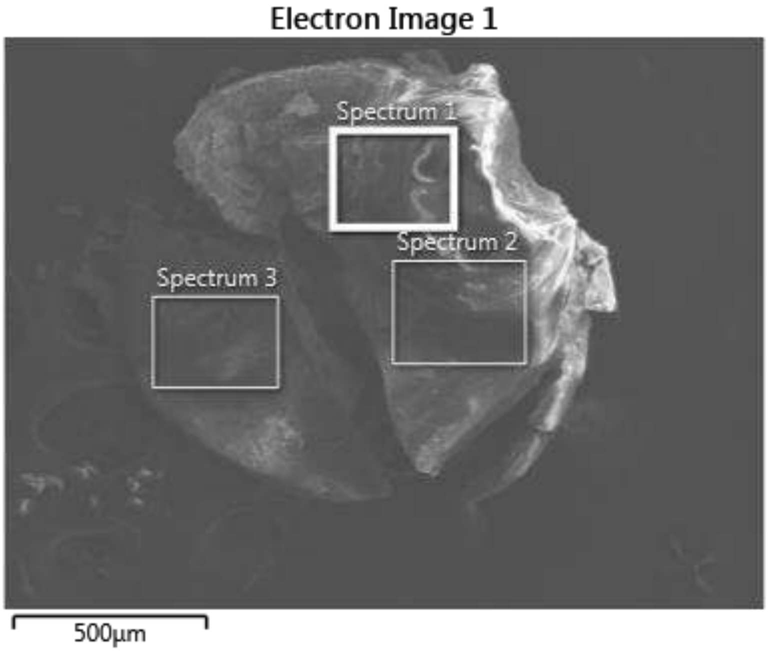

22/01/2020 10:26:44
Project 1
Rumania 1
Dorso 1 15 Kv

Electron Image 1

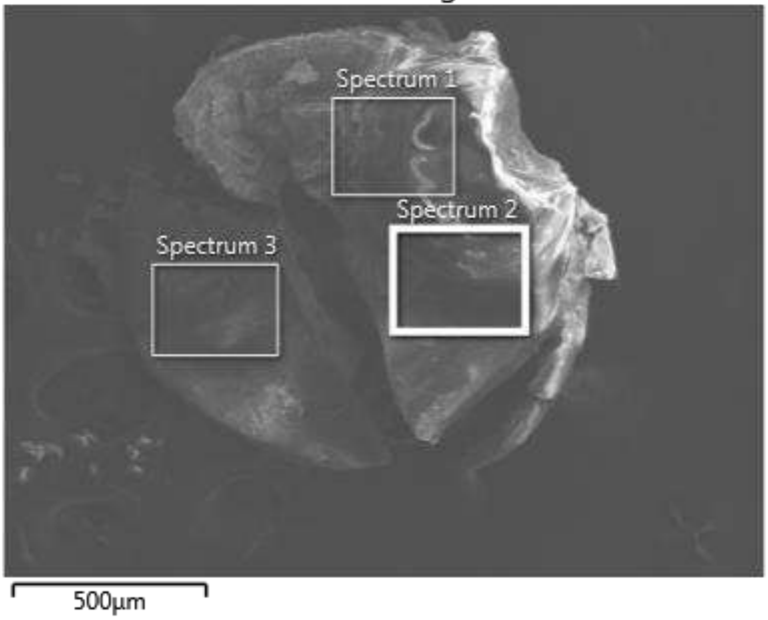

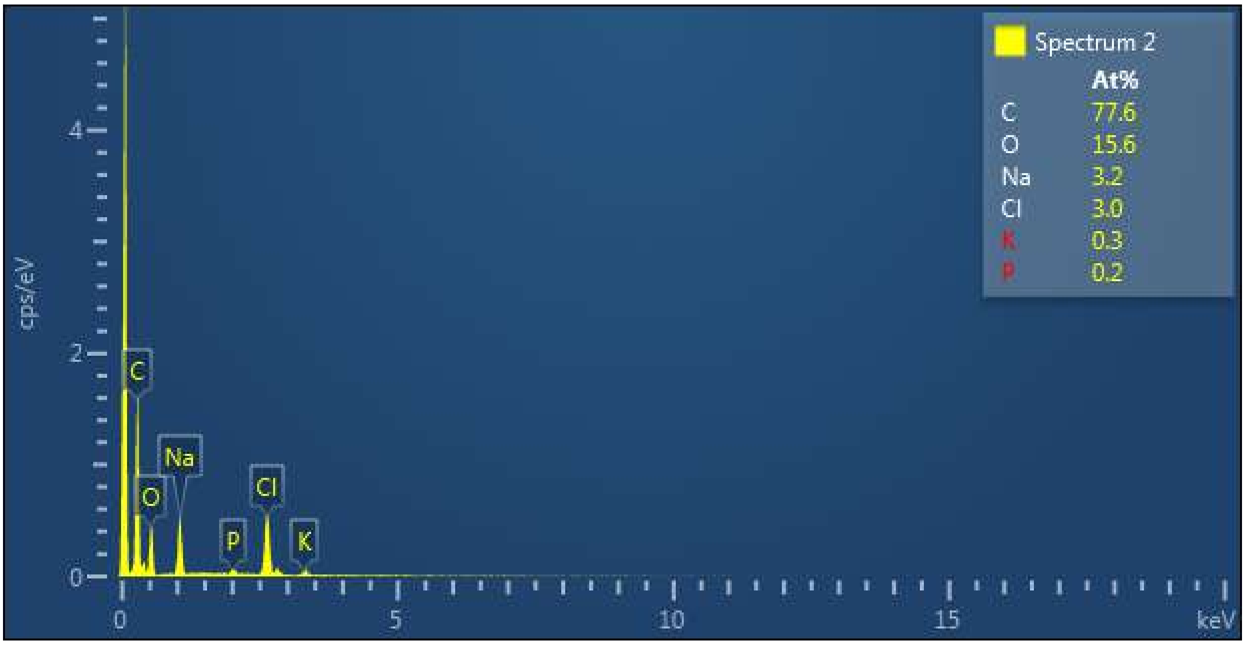

Label:	Spectrum 2
Source:	Acquired
Created:	22/01/2020 10:26:44
Livetime:	60.1s
Process Time:	4
Accelerating Voltage:	15.00kV
Magnification:	57 x
Working Distance:	10.0mm
Specimen Tilt (degrees):	0.0
Elevation (degrees):	35.0
Azimuth (degrees):	0.0
Number Of Channels:	2048
Energy Range (keV):	20 keV
Energy per Channel (eV):	10.0eV
Detector Type Id:	29
Detector Type:	X-Max
Window Type:	SATW
Pulse Pile Up Correction:	Succeeded
Primary Detector:	2617
Primary Detector Serial Number:	77871-X080

Element	Line Type	Apparent Concentration	k Ratio	Wt%	Wt% Sigma	Atomic %	Standard Label	Factory Standard	Standard Calibration Date
C	K series	2.82	0.02817	67.50	0.94	77.65	C Vit	Yes	
O	K series	1.35	0.00453	18.05	0.73	15.59	SiO2	Yes	
Na	K	0.89	0.0037	5.37	0.23	3.23	Albite	Yes	

	series		4						
P	K series	0.10	0.00056	0.53	0.09	0.23	GaP	Yes	
Cl	K series	0.96	0.00840	7.74	0.29	3.02	NaCl	Yes	
K	K series	0.10	0.00086	0.80	0.12	0.28	KBr	Yes	
Total:				100.00		100.00			

Electron Image 1

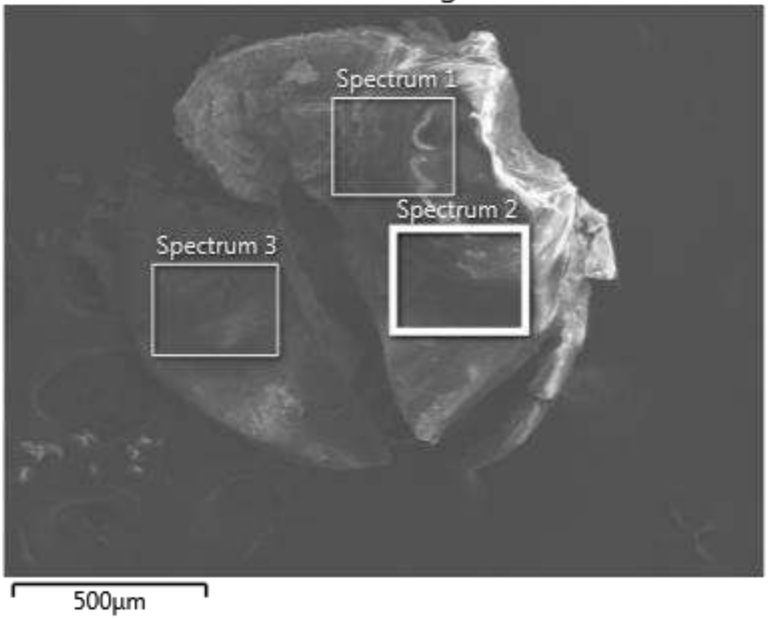

22/01/2020 10:27:53
Project 1
Rumania 1
Dorso 1 15 Kv

Electron Image 1

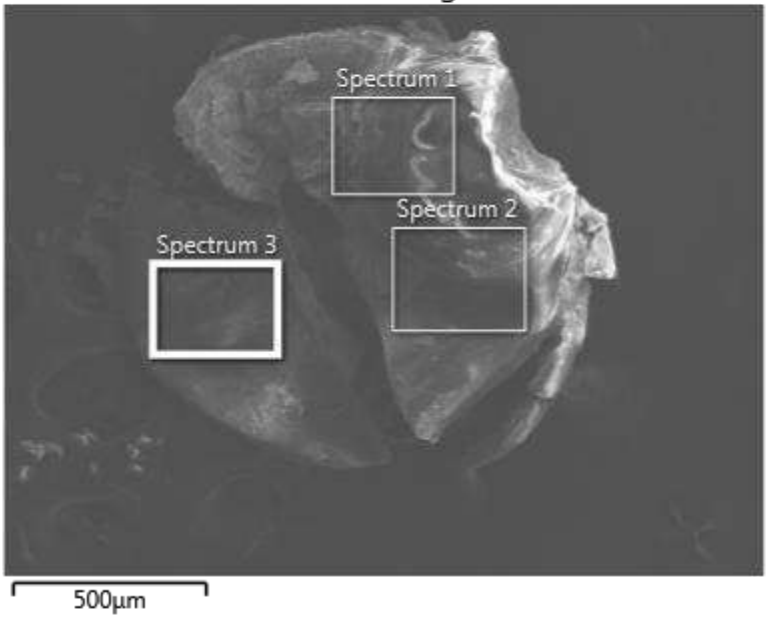

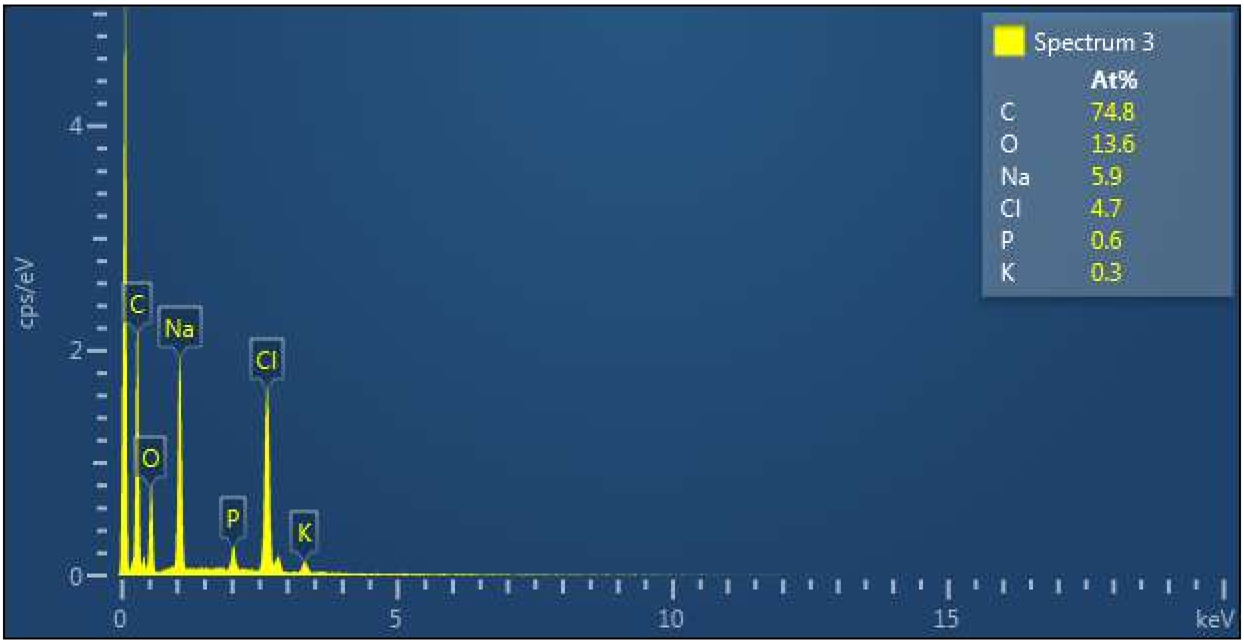

Label:	Spectrum 3
Source:	Acquired
Created:	22/01/2020 10:27:53
Livetime:	60.0s
Process Time:	4
Accelerating Voltage:	15.00kV
Magnification:	57 x
Working Distance:	10.0mm
Specimen Tilt (degrees):	0.0
Elevation (degrees):	35.0
Azimuth (degrees):	0.0
Number Of Channels:	2048
Energy Range (keV):	20 keV
Energy per Channel (eV):	10.0eV
Detector Type Id:	29
Detector Type:	X-Max
Window Type:	SATW
Pulse Pile Up Correction:	Succeeded
Primary Detector:	2617
Primary Detector Serial Number:	77871-X080

Element	Line Type	Apparent Concentration	k Ratio	Wt%	Wt% Sigma	Atomic %	Standard Label	Factory Standard	Standard Calibration Date
C	K series	3.80	0.03800	61.92	0.78	74.82	C Vit	Yes	
O	K series	2.32	0.00781	14.99	0.48	13.60	SiO2	Yes	
Na	K	3.32	0.0140	9.41	0.24	5.94	Albite	Yes	

	series		2						
P	K series	0.47	0.00261	1.19	0.08	0.56	GaP	Yes	
Cl	K series	2.95	0.02578	11.54	0.28	4.73	NaCl	Yes	
K	K series	0.24	0.00204	0.93	0.08	0.35	KBr	Yes	
Total:				100.00		100.00			

Electron Image 1

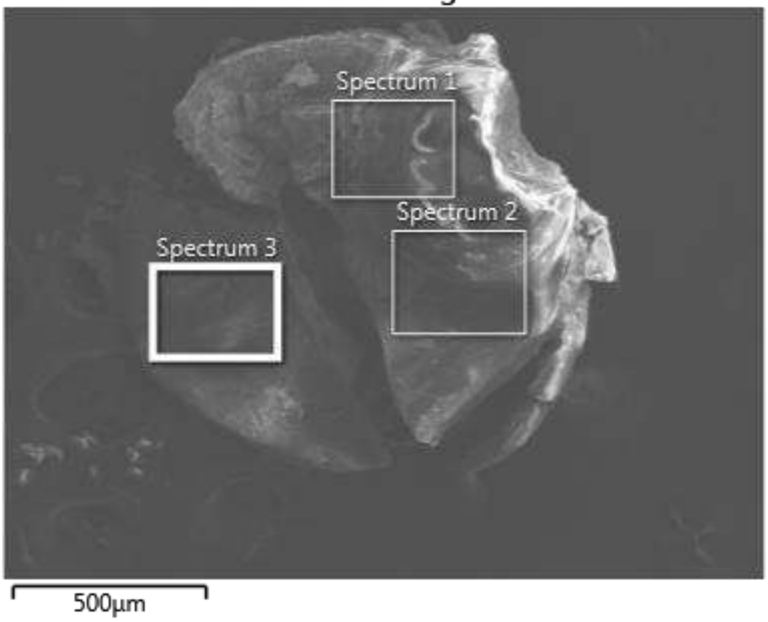

22/01/2020 10:38:53
Project 1
Rumania 2
Trozos 15 Kv

Electron Image 2

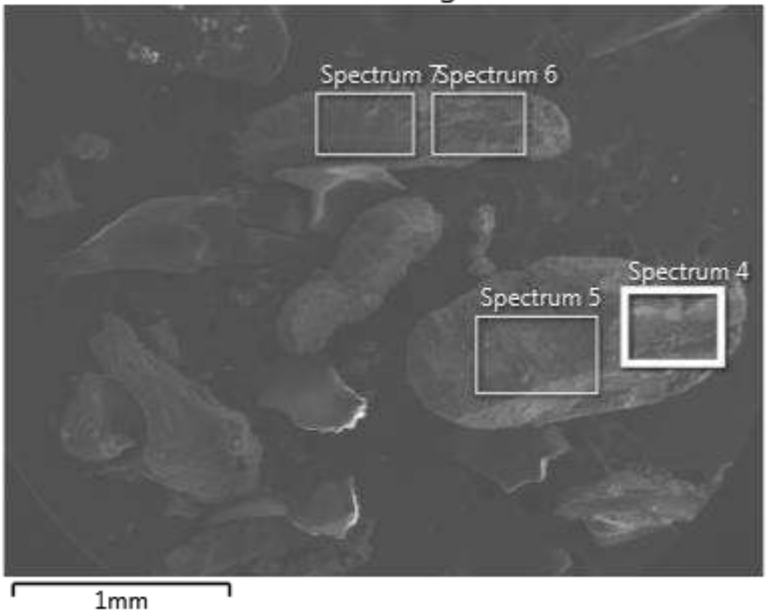

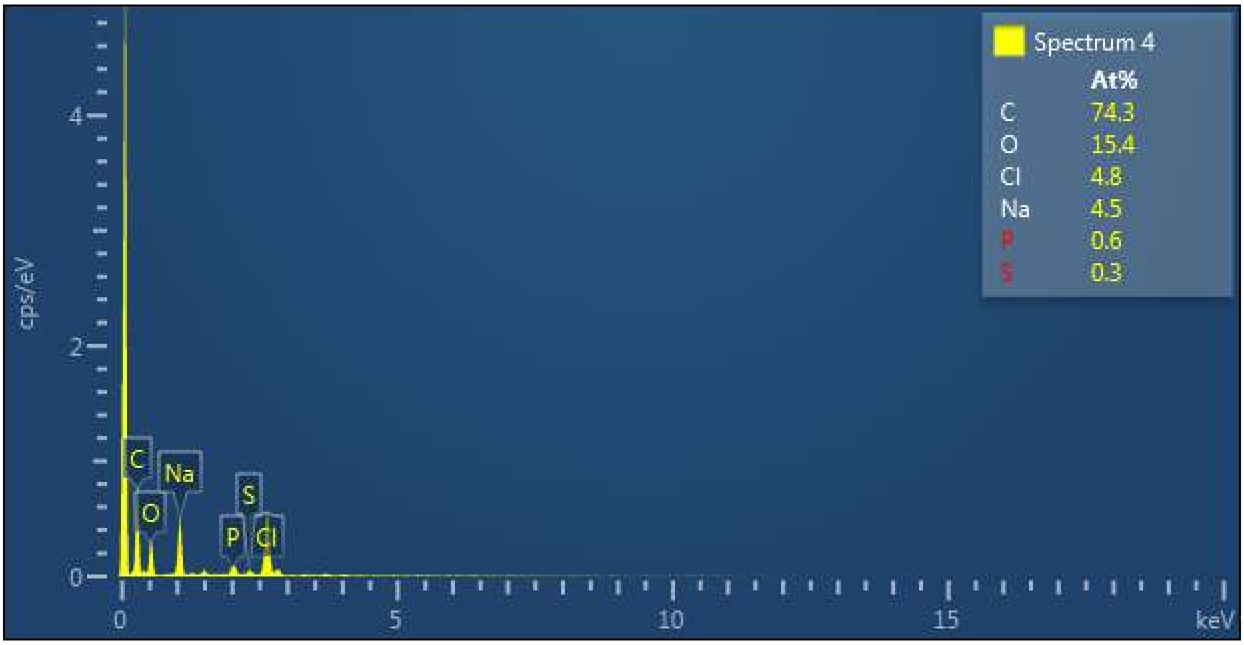

Label:	Spectrum 4
Source:	Acquired
Created:	22/01/2020 10:38:53
Livetime:	60.5s
Process Time:	4
Accelerating Voltage:	15.00kV
Magnification:	32 x
Working Distance:	10.0mm
Specimen Tilt (degrees):	0.0
Elevation (degrees):	35.0
Azimuth (degrees):	0.0
Number Of Channels:	2048
Energy Range (keV):	20 keV
Energy per Channel (eV):	10.0eV
Detector Type Id:	29
Detector Type:	X-Max
Window Type:	SATW
Pulse Pile Up Correction:	Succeeded
Primary Detector:	2617
Primary Detector Serial Number:	77871-X080

Element	Line Type	Apparent Concentration	k Ratio	Wt%	Wt% Sigma	Atomic %	Standard Label	Factory Standard	Standard Calibration Date
C	K series	1.22	0.01218	61.84	1.10	74.30	C Vit	Yes	
O	K series	0.89	0.00299	17.10	0.83	15.43	SiO2	Yes	
Na	K	0.83	0.0035	7.24	0.31	4.55	Albite	Yes	

	series		1						
P	K series	0.17	0.00096	1.31	0.14	0.61	GaP	Yes	
S	K series	0.05	0.00045	0.59	0.12	0.26	FeS2	Yes	
Cl	K series	1.01	0.00880	11.91	0.43	4.85	NaCl	Yes	
Total:				100.00		100.00			

Electron Image 2

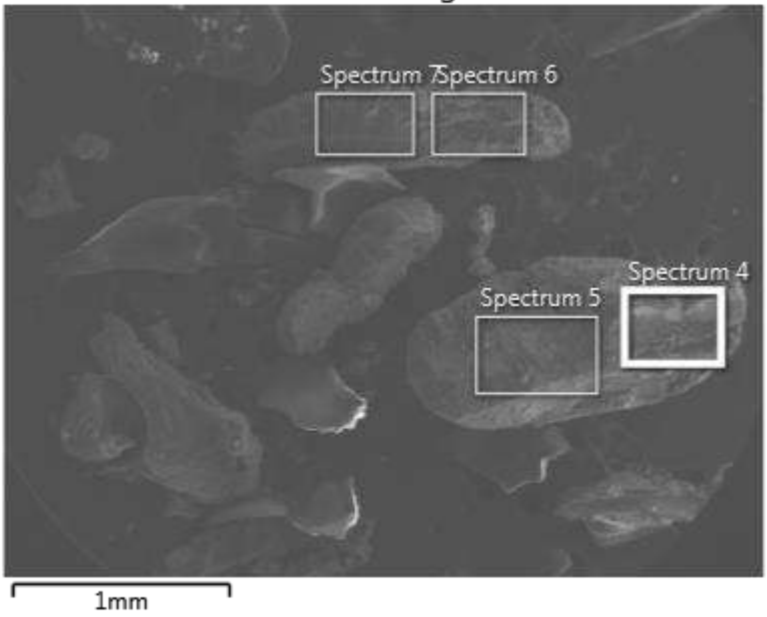

22/01/2020 10:40:05
Project 1
Rumania 2
Trozos 15 Kv

Electron Image 2

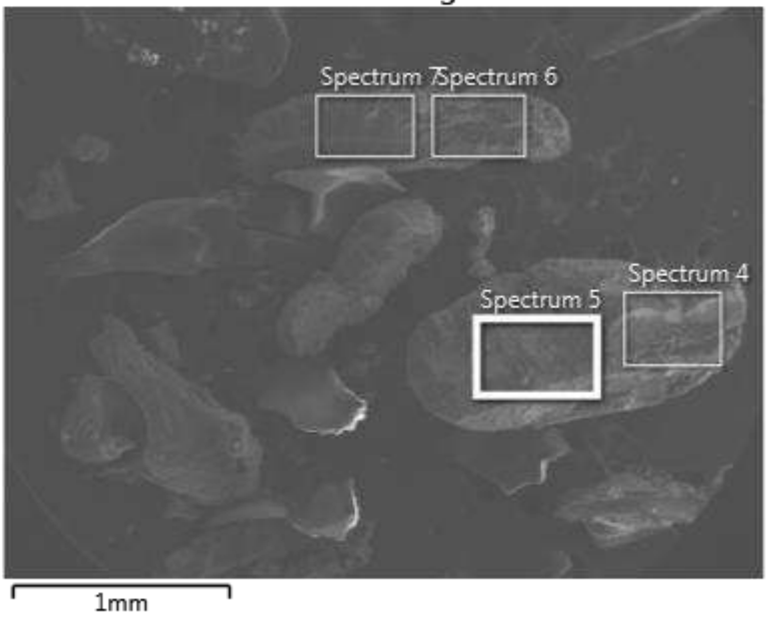

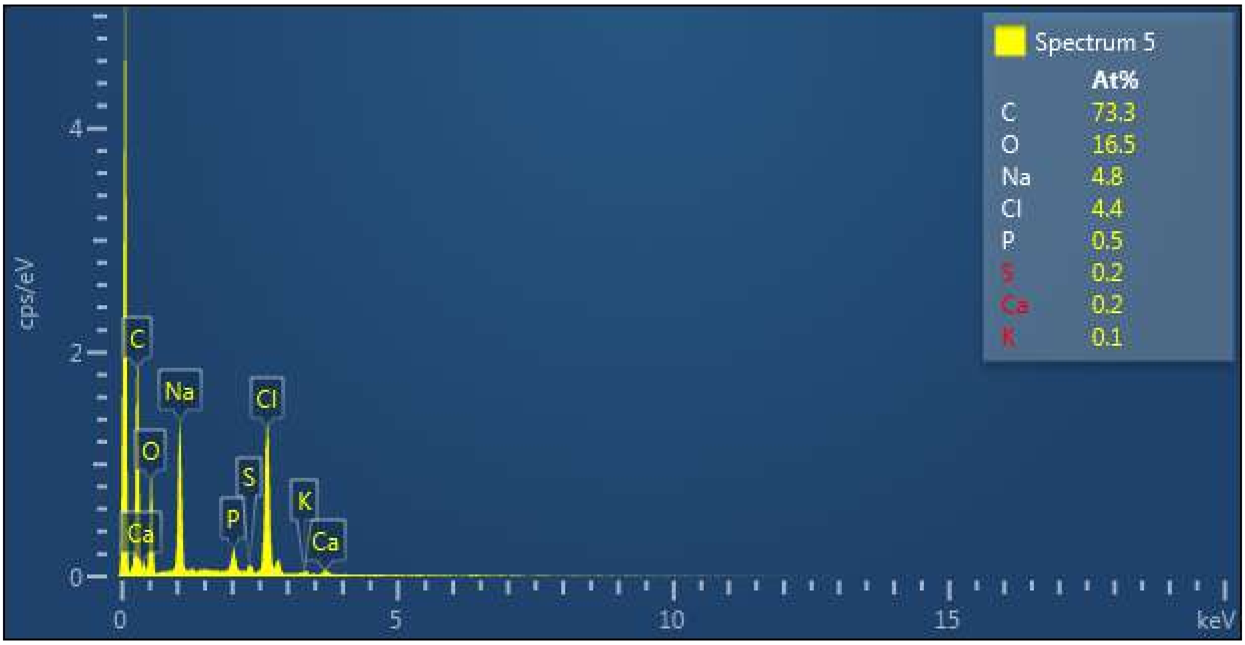

Label:	Spectrum 5
Source:	Acquired
Created:	22/01/2020 10:40:05
Livetime:	60.2s
Process Time:	4
Accelerating Voltage:	15.00kV
Magnification:	32 x
Working Distance:	10.0mm
Specimen Tilt (degrees):	0.0
Elevation (degrees):	35.0
Azimuth (degrees):	0.0
Number Of Channels:	2048
Energy Range (keV):	20 keV
Energy per Channel (eV):	10.0eV
Detector Type Id:	29
Detector Type:	X-Max
Window Type:	SATW
Pulse Pile Up Correction:	Succeeded
Primary Detector:	2617
Primary Detector Serial Number:	77871-X080

Element	Line Type	Apparent Concentration	k Ratio	Wt%	Wt% Sigma	Atomic %	Standard Label	Factory Standard	Standard Calibration Date
C	K series	3.43	0.03432	60.91	0.86	73.26	C Vit	Yes	
O	K series	2.57	0.00864	18.31	0.56	16.53	SiO2	Yes	
Na	K	2.32	0.0097	7.62	0.22	4.79	Albite	Yes	

	series		8						
P	K series	0.41	0.00229	1.18	0.08	0.55	GaP	Yes	
S	K series	0.11	0.00097	0.47	0.06	0.21	FeS2	Yes	
Cl	K series	2.42	0.02112	10.73	0.28	4.37	NaCl	Yes	
K	K series	0.06	0.00050	0.26	0.07	0.10	KBr	Yes	
Ca	K series	0.12	0.00103	0.52	0.08	0.19	Wollastonite	Yes	
Total:				100.00		100.00			

Electron Image 2

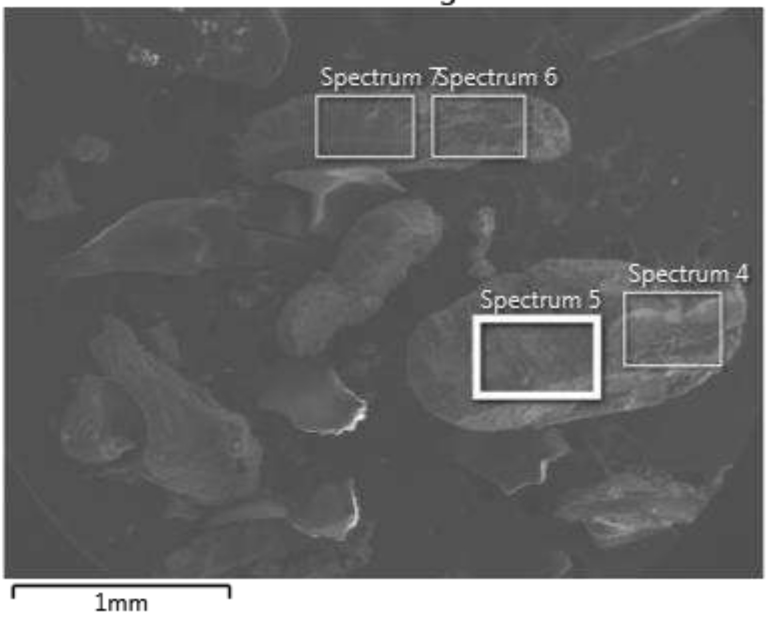

22/01/2020 10:41:15
Project 1
Rumania 2
Trozos 15 Kv

Electron Image 2

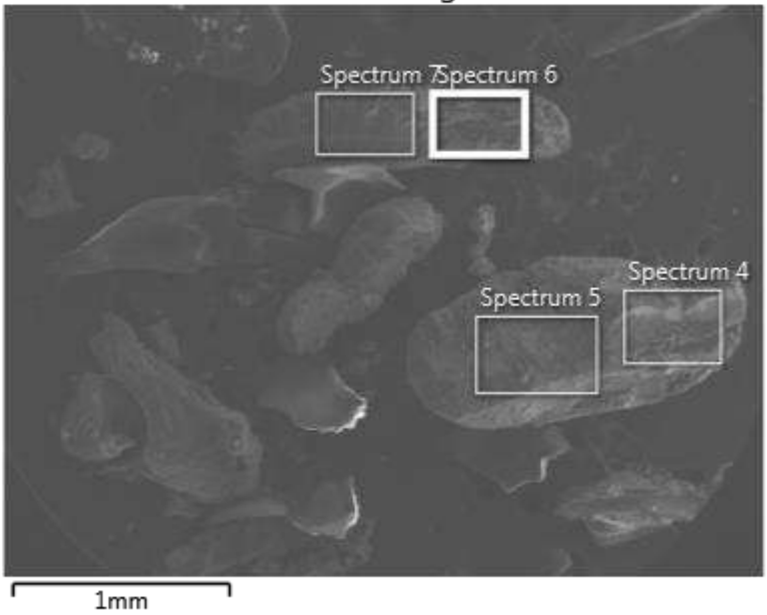

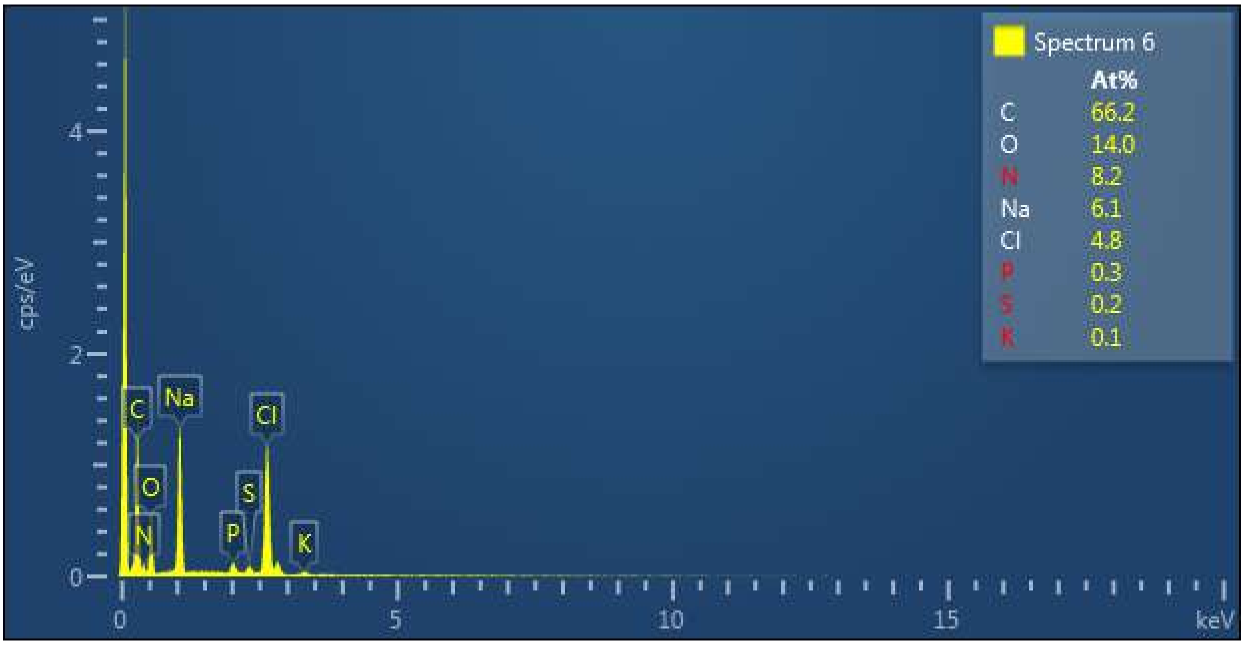

Label:	Spectrum 6
Source:	Acquired
Created:	22/01/2020 10:41:15
Livetime:	60.0s
Process Time:	4
Accelerating Voltage:	15.00kV
Magnification:	32 x
Working Distance:	10.0mm
Specimen Tilt (degrees):	0.0
Elevation (degrees):	35.0
Azimuth (degrees):	0.0
Number Of Channels:	2048
Energy Range (keV):	20 keV
Energy per Channel (eV):	10.0eV
Detector Type Id:	29
Detector Type:	X-Max
Window Type:	SATW
Pulse Pile Up Correction:	Succeeded
Primary Detector:	2617
Primary Detector Serial Number:	77871-X080

Element	Line Type	Apparent Concentration	k Ratio	Wt%	Wt% Sigma	Atomic %	Standard Label	Factory Standard	Standard Calibration Date
C	K series	2.26	0.02258	54.15	1.40	66.15	C Vit	Yes	
N	K series	0.87	0.00154	7.84	1.49	8.21	BN	Yes	
O	K	1.60	0.0053	15.30	0.64	14.03	SiO2	Yes	

	series		7						
Na	K series	2.32	0.00978	9.60	0.33	6.13	Albite	Yes	
P	K series	0.19	0.00107	0.71	0.08	0.33	GaP	Yes	
S	K series	0.09	0.00077	0.48	0.08	0.22	FeS2	Yes	
Cl	K series	2.06	0.01799	11.60	0.39	4.80	NaCl	Yes	
K	K series	0.06	0.00049	0.32	0.08	0.12	KBr	Yes	
Total:				100.00		100.00			

Electron Image 2

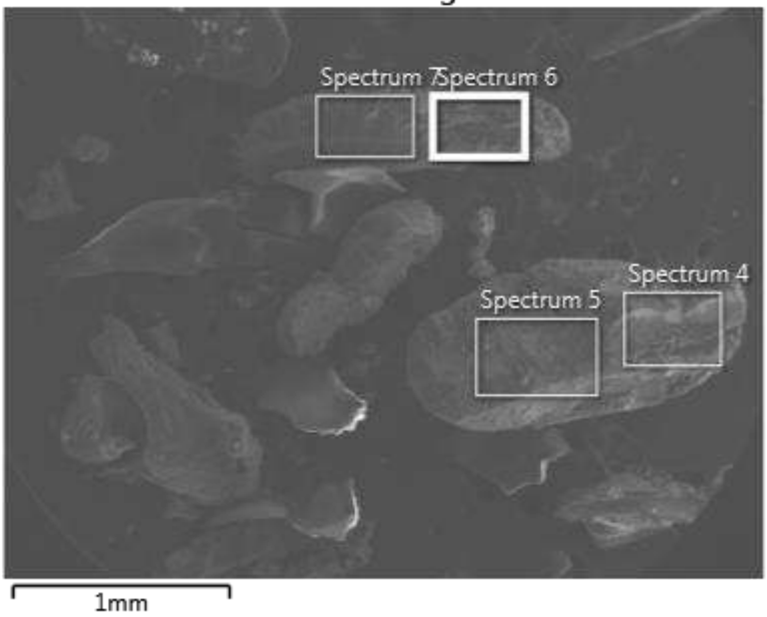

22/01/2020 10:42:27
Project 1
Rumania 2
Trozos 15 Kv

Electron Image 2

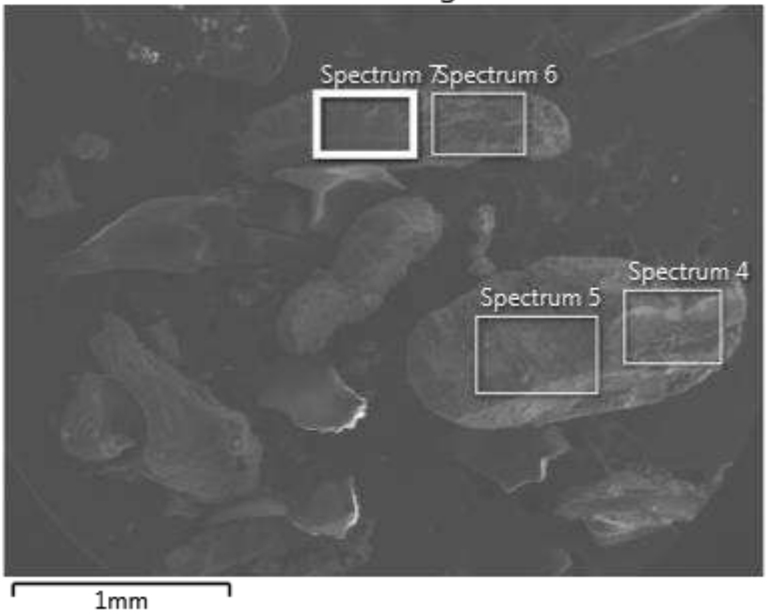

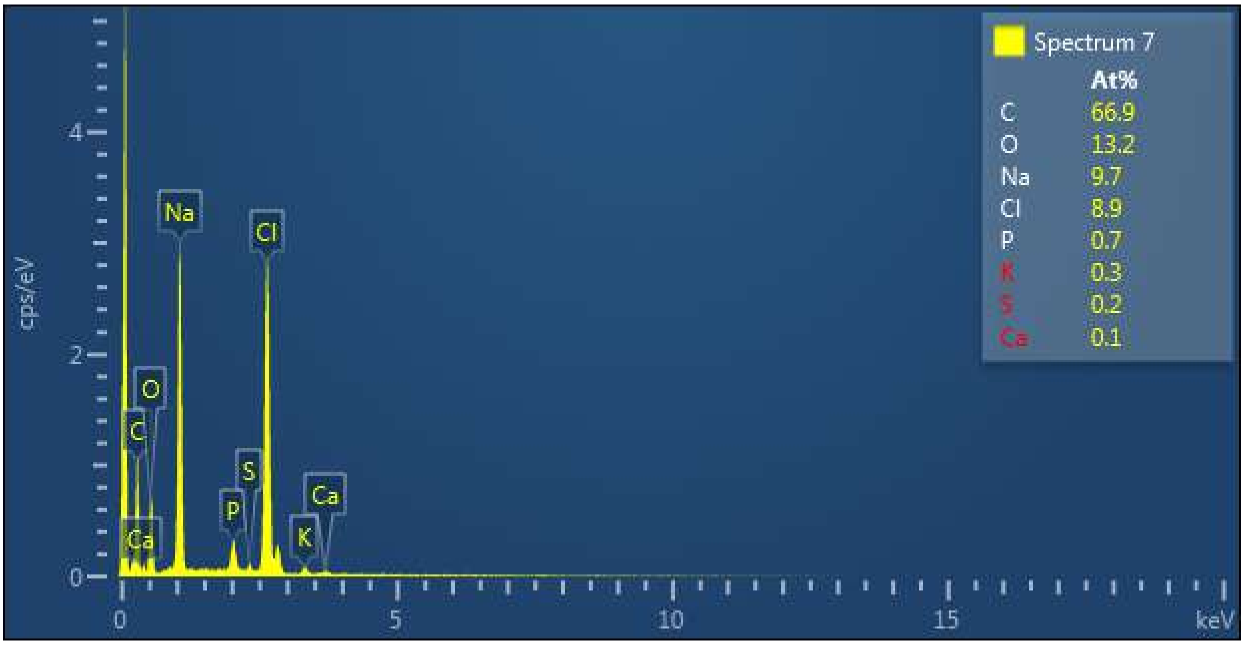

Label:	Spectrum 7
Source:	Acquired
Created:	22/01/2020 10:42:27
Livetime:	60.3s
Process Time:	4
Accelerating Voltage:	15.00kV
Magnification:	32 x
Working Distance:	10.0mm
Specimen Tilt (degrees):	0.0
Elevation (degrees):	35.0
Azimuth (degrees):	0.0
Number Of Channels:	2048
Energy Range (keV):	20 keV
Energy per Channel (eV):	10.0eV
Detector Type Id:	29
Detector Type:	X-Max
Window Type:	SATW
Pulse Pile Up Correction:	Succeeded
Primary Detector:	2617
Primary Detector Serial Number:	77871-X080

Element	Line Type	Apparent Concentration	k Ratio	Wt%	Wt% Sigma	Atomic %	Standard Label	Factory Standard	Standard Calibration Date
C	K series	1.93	0.01935	50.29	1.15	66.87	C Vit	Yes	
O	K series	2.11	0.00709	13.25	0.48	13.23	SiO2	Yes	
Na	K	5.10	0.0215	14.00	0.36	9.72	Albite	Yes	

	series		1						
P	K series	0.55	0.00308	1.41	0.09	0.72	GaP	Yes	
S	K series	0.10	0.00084	0.36	0.06	0.18	FeS2	Yes	
Cl	K series	5.06	0.04423	19.76	0.49	8.90	NaCl	Yes	
K	K series	0.16	0.00139	0.65	0.08	0.26	KBr	Yes	
Ca	K series	0.07	0.00064	0.29	0.07	0.12	Wollastonite	Yes	
Total:				100.00		100.00			

Electron Image 2

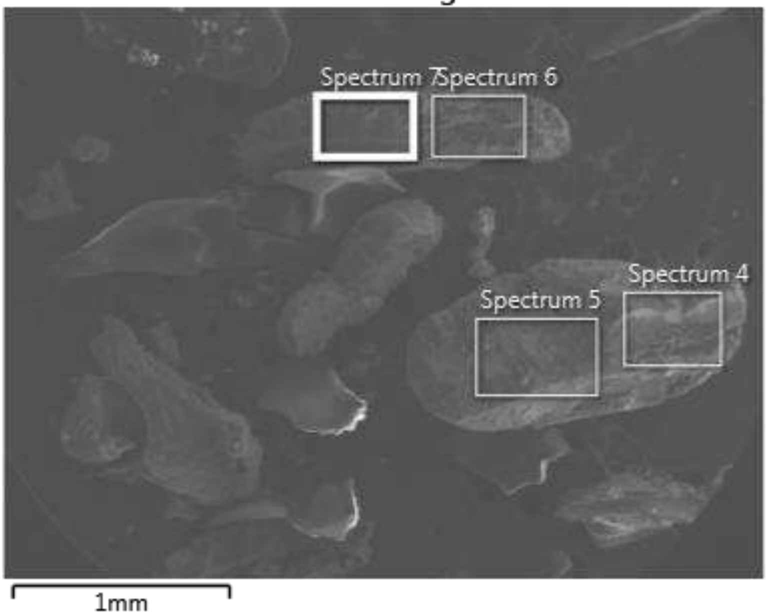

22/01/2020 10:47:06
Project 1
Rumania 3
Trozos 15 Kv

Electron Image 3

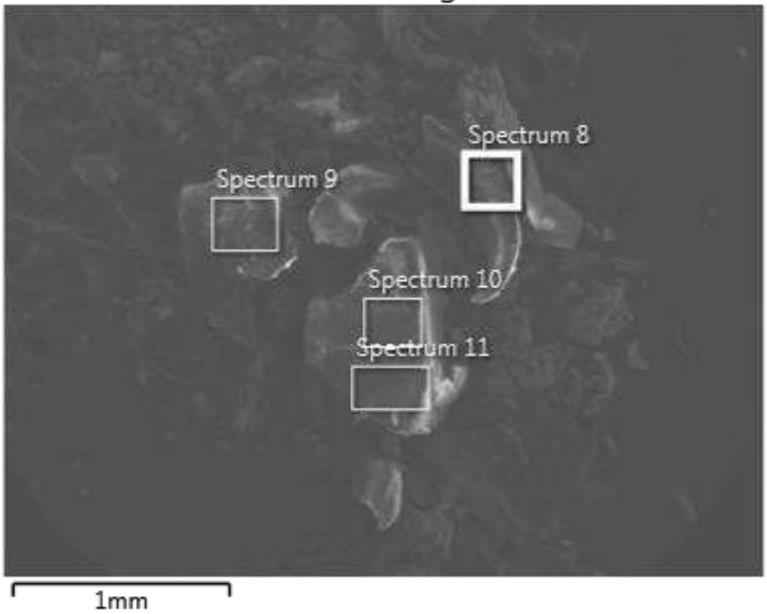

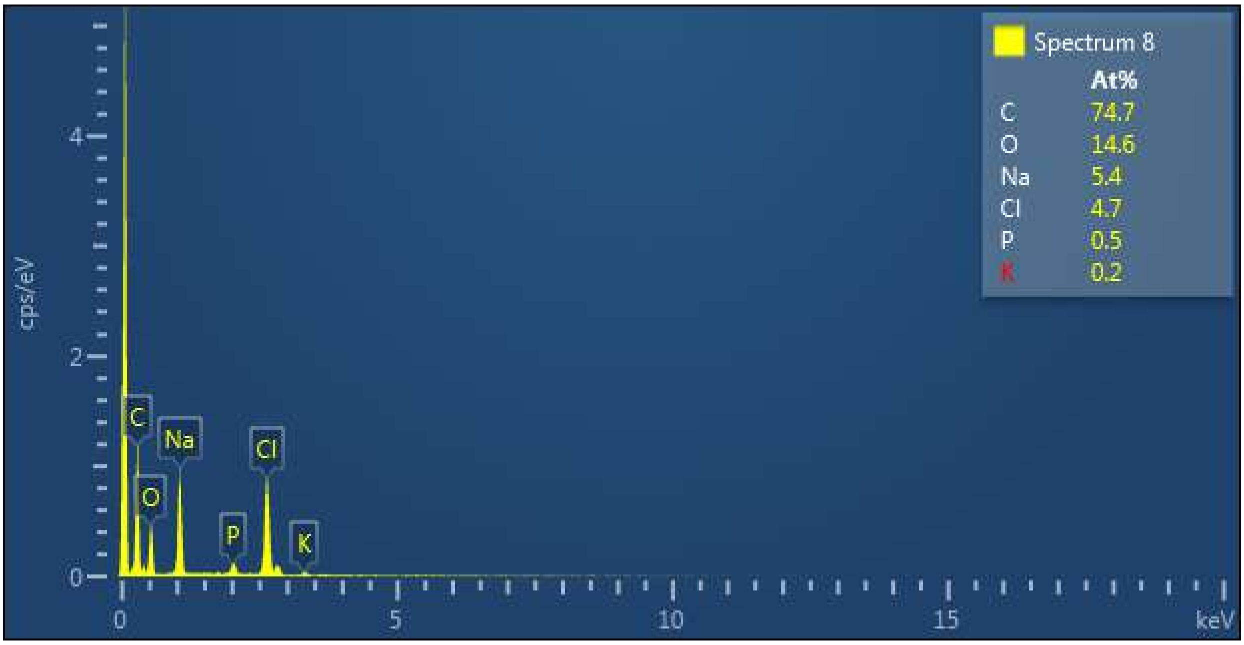

Label:	Spectrum 8
Source:	Acquired
Created:	22/01/2020 10:47:06
Livetime:	60.3s
Process Time:	4
Accelerating Voltage:	15.00kV
Magnification:	32 x
Working Distance:	10.0mm
Specimen Tilt (degrees):	0.0
Elevation (degrees):	35.0
Azimuth (degrees):	0.0
Number Of Channels:	2048
Energy Range (keV):	20 keV
Energy per Channel (eV):	10.0eV
Detector Type Id:	29
Detector Type:	X-Max
Window Type:	SATW
Pulse Pile Up Correction:	Succeeded
Primary Detector:	2617
Primary Detector Serial Number:	77871-X080

Element	Line Type	Apparent Concentration	k Ratio	Wt%	Wt% Sigma	Atomic %	Standard Label	Factory Standard	Standard Calibration Date
C	K series	2.03	0.02026	62.18	1.08	74.70	C Vit	Yes	
O	K series	1.32	0.00444	16.15	0.70	14.56	SiO2	Yes	
Na	K	1.56	0.0065	8.52	0.31	5.35	Albite	Yes	

	series		8						
P	K series	0.21	0.00119	1.03	0.10	0.48	GaP	Yes	
Cl	K series	1.55	0.01354	11.57	0.39	4.71	NaCl	Yes	
K	K series	0.07	0.00062	0.54	0.10	0.20	KBr	Yes	
Total:				100.00		100.00			

Electron Image 3

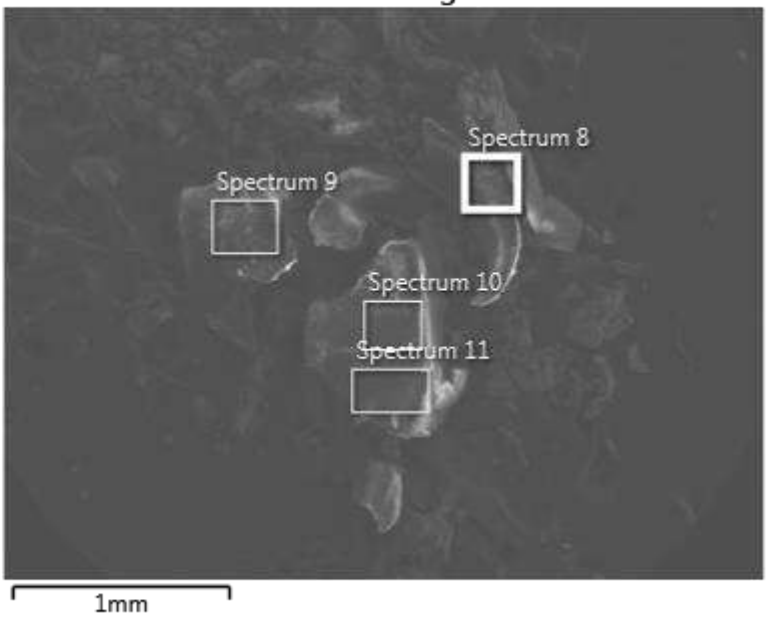

22/01/2020 10:48:16
Project 1
Rumania 3
Trozos 15 Kv

Electron Image 3

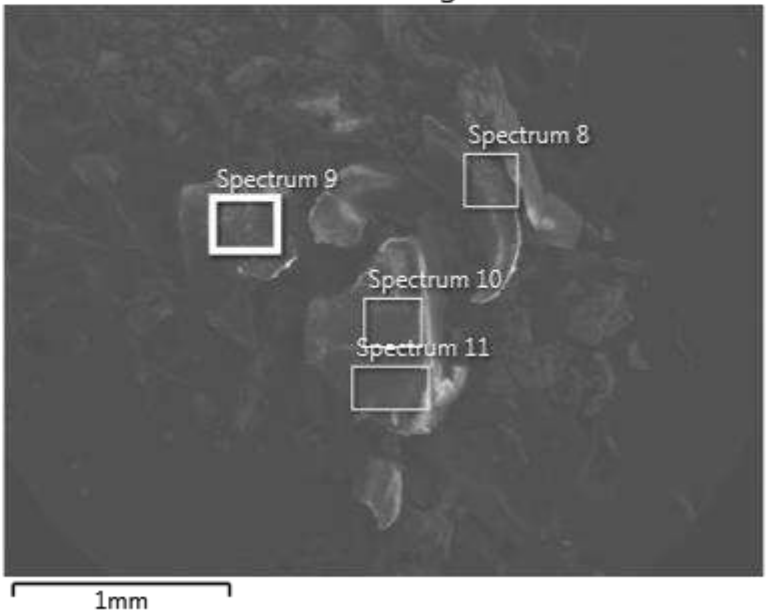

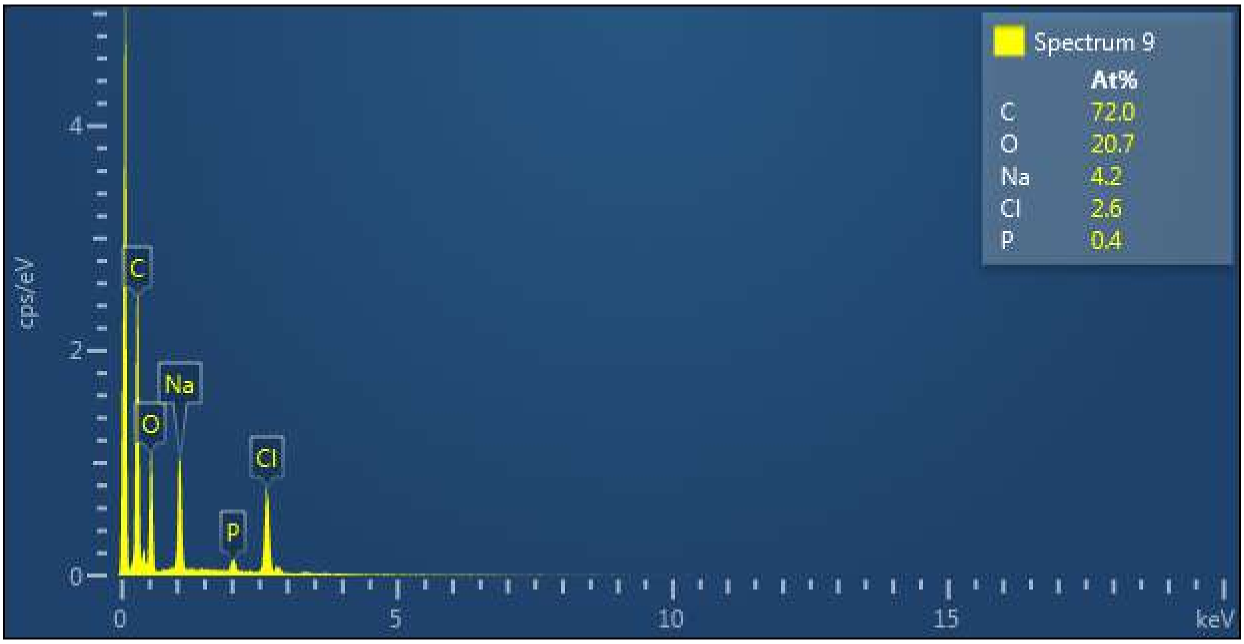

Label:	Spectrum 9
Source:	Acquired
Created:	22/01/2020 10:48:16
Livetime:	60.3s
Process Time:	4
Accelerating Voltage:	15.00kV
Magnification:	32 x
Working Distance:	10.0mm
Specimen Tilt (degrees):	0.0
Elevation (degrees):	35.0
Azimuth (degrees):	0.0
Number Of Channels:	2048
Energy Range (keV):	20 keV
Energy per Channel (eV):	10.0eV
Detector Type Id:	29
Detector Type:	X-Max
Window Type:	SATW
Pulse Pile Up Correction:	Succeeded
Primary Detector:	2617
Primary Detector Serial Number:	77871-X080

Element	Line Type	Apparent Concentration	k Ratio	Wt%	Wt% Sigma	Atomic %	Standard Label	Factory Standard	Standard Calibration Date
C	K series	4.15	0.04153	61.83	0.61	72.03	C Vit	Yes	
O	K series	3.14	0.01055	23.72	0.55	20.74	SiO2	Yes	
Na	K	1.79	0.0075	6.91	0.19	4.21	Albite	Yes	

	serie s		5						
P	K serie s	0.25	0.0014 0	0.83	0.08	0.37	GaP	Yes	
Cl	K serie s	1.32	0.0115 7	6.71	0.18	2.65	NaCl	Yes	
Total:				100.0 0		100.0 0			

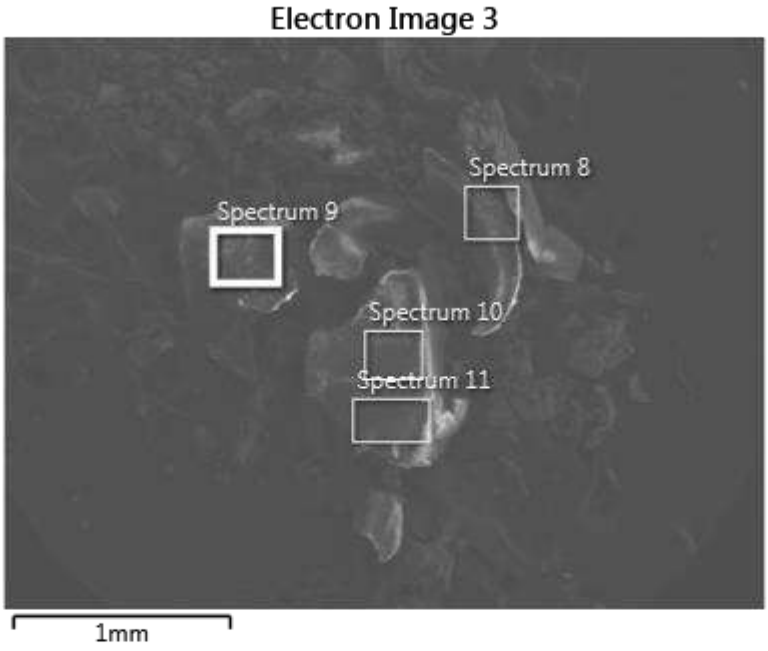

22/01/2020 10:49:27
Project 1
Rumania 3
Trozos 15 Kv

Electron Image 3

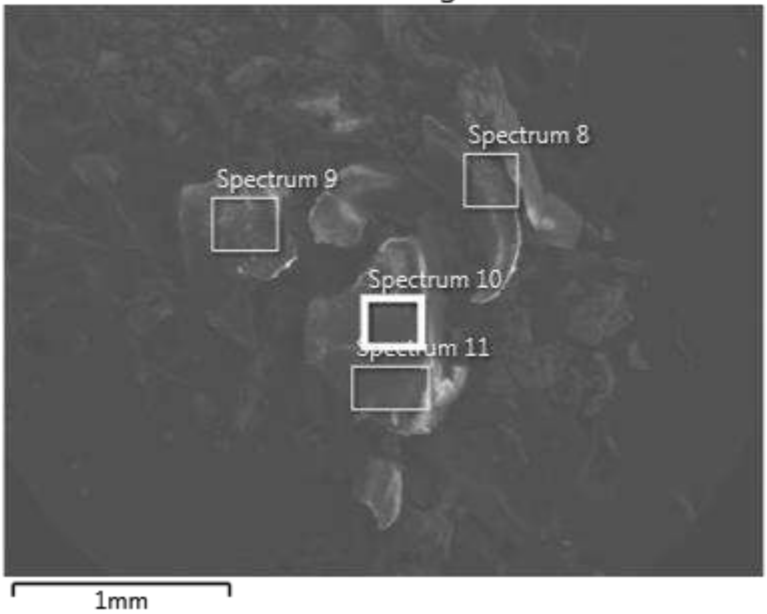

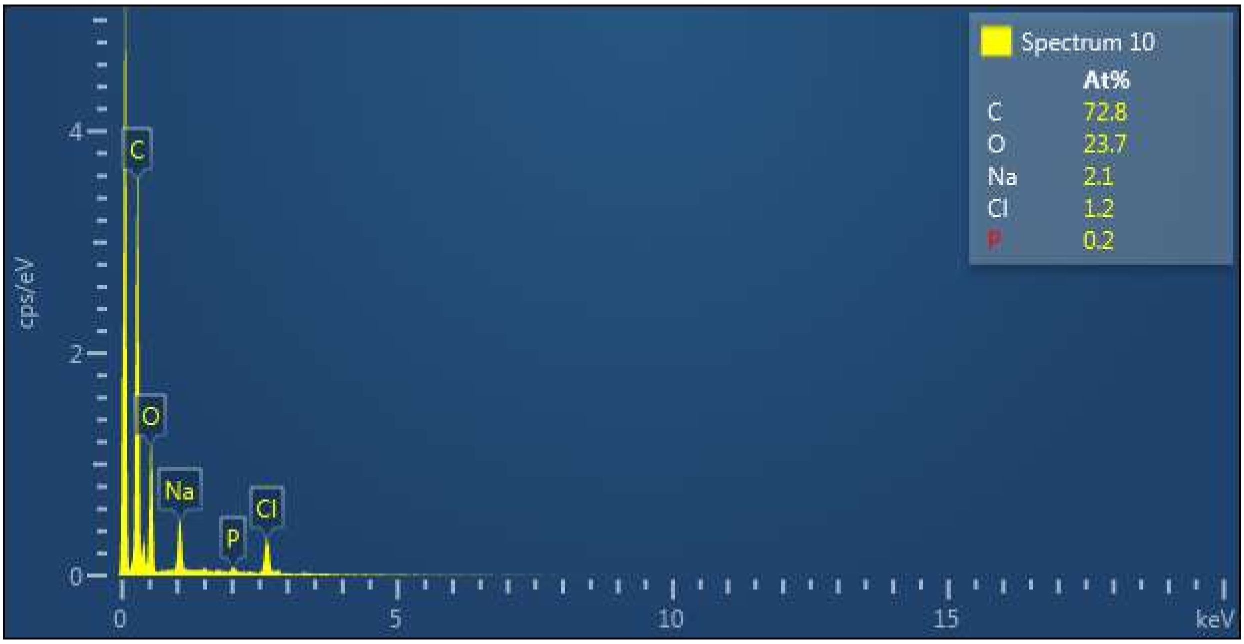

Label:	Spectrum 10
Source:	Acquired
Created:	22/01/2020 10:49:27
Livetime:	60.1s
Process Time:	4
Accelerating Voltage:	15.00kV
Magnification:	32 x
Working Distance:	10.0mm
Specimen Tilt (degrees):	0.0
Elevation (degrees):	35.0
Azimuth (degrees):	0.0
Number Of Channels:	2048
Energy Range (keV):	20 keV
Energy per Channel (eV):	10.0eV
Detector Type Id:	29
Detector Type:	X-Max
Window Type:	SATW
Pulse Pile Up Correction:	Succeeded
Primary Detector:	2617
Primary Detector Serial Number:	77871-X080

Element	Line Type	Apparent Concentration	k Ratio	Wt%	Wt% Sigma	Atomic %	Standard Label	Factory Standard	Standard Calibration Date
C	K series	5.67	0.05671	64.78	0.60	72.82	C Vit	Yes	
O	K series	3.39	0.01139	28.08	0.59	23.69	SiO2	Yes	
Na	K	0.80	0.0033	3.59	0.15	2.11	Albite	Yes	

	serie s		6						
P	K serie s	0.10	0.0005 6	0.37	0.07	0.16	GaP	Yes	
Cl	K serie s	0.56	0.0049 0	3.18	0.13	1.21	NaCl	Yes	
Total:				100.0 0		100.0 0			

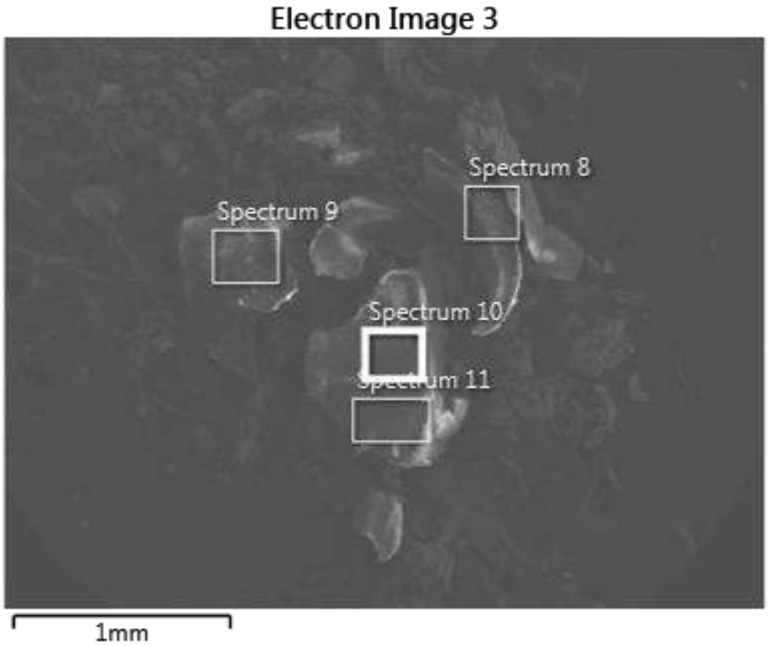

22/01/2020 10:50:37
Project 1
Rumania 3
Trozos 15 Kv

Electron Image 3

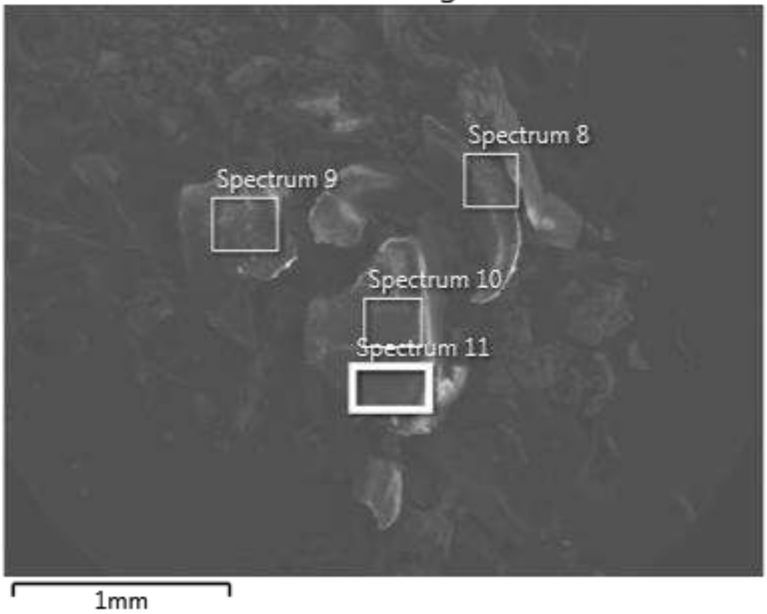

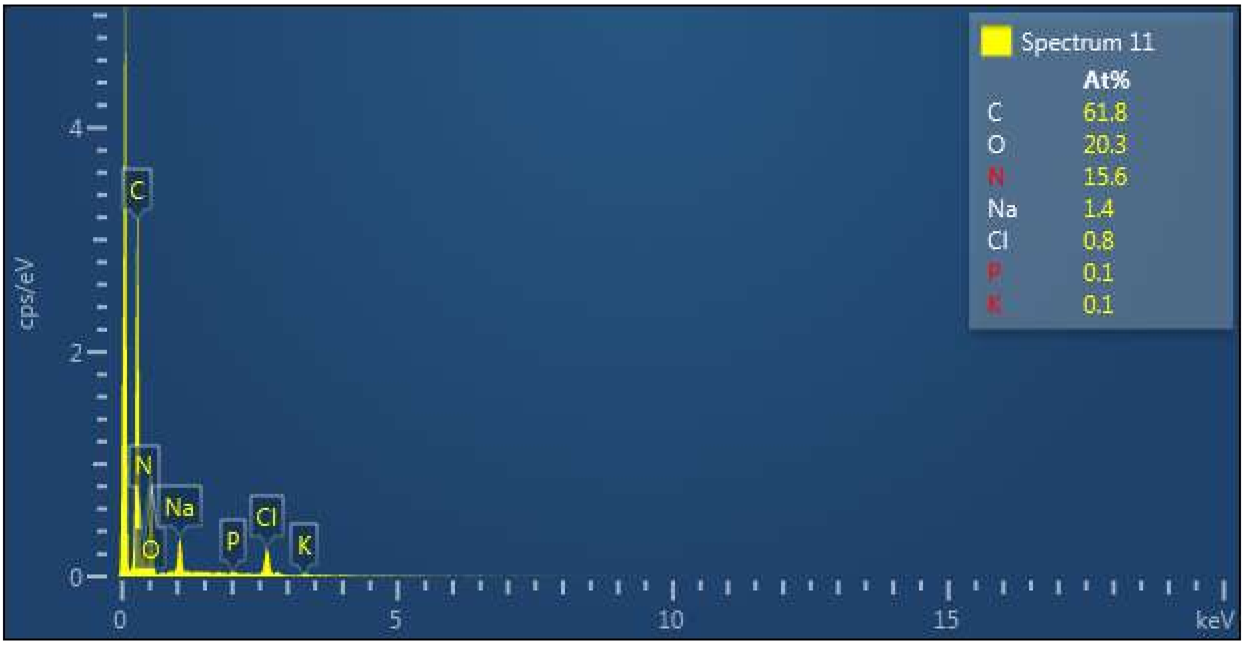

Label:	Spectrum 11
Source:	Acquired
Created:	22/01/2020 10:50:37
Livetime:	60.1s
Process Time:	4
Accelerating Voltage:	15.00kV
Magnification:	32 x
Working Distance:	10.0mm
Specimen Tilt (degrees):	0.0
Elevation (degrees):	35.0
Azimuth (degrees):	0.0
Number Of Channels:	2048
Energy Range (keV):	20 keV
Energy per Channel (eV):	10.0eV
Detector Type Id:	29
Detector Type:	X-Max
Window Type:	SATW
Pulse Pile Up Correction:	Succeeded
Primary Detector:	2617
Primary Detector Serial Number:	77871-X080

Element	Line Type	Apparent Concentration	k Ratio	Wt%	Wt% Sigma	Atomic %	Standard Label	Factory Standard	Standard Calibration Date
C	K series	5.89	0.05888	54.99	1.23	61.84	C Vit	Yes	
N	K series	2.28	0.00405	16.14	1.56	15.56	BN	Yes	
O	K	2.63	0.0088	24.00	0.75	20.26	SiO2	Yes	

	series		4						
Na	K series	0.54	0.00226	2.30	0.13	1.35	Albite	Yes	
P	K series	0.05	0.00026	0.16	0.05	0.07	GaP	Yes	
Cl	K series	0.42	0.00367	2.22	0.12	0.85	NaCl	Yes	
K	K series	0.04	0.00031	0.19	0.06	0.06	KBr	Yes	
Total:				100.00		100.00			

Electron Image 3

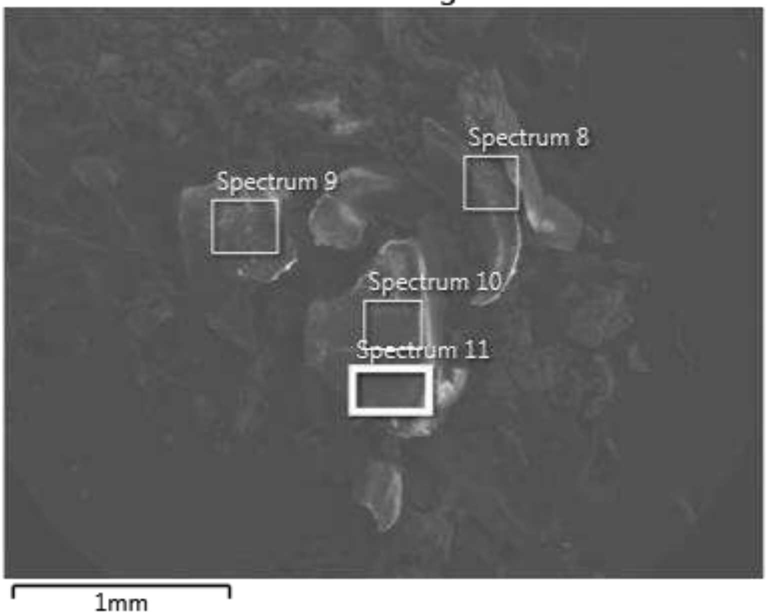

22/01/2020 10:53:40
Project 1
Rumania 4
Dorso 1 15 Kv

Electron Image 4

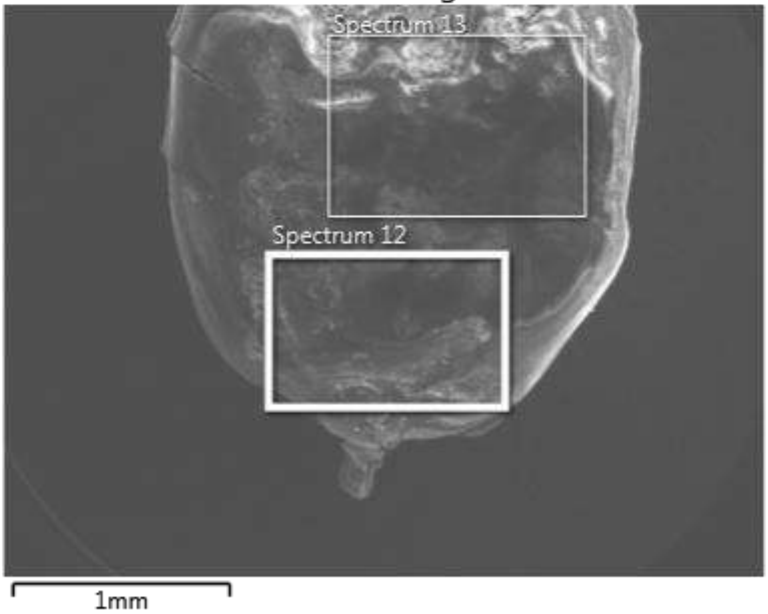

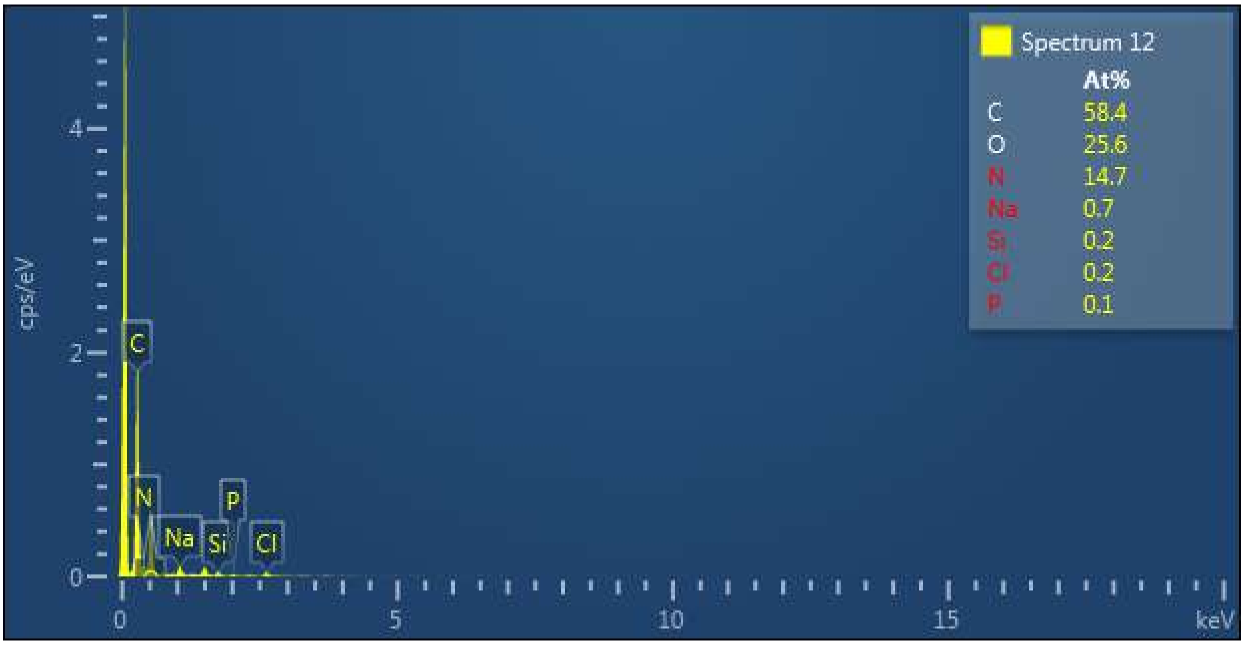

Label:	Spectrum 12
Source:	Acquired
Created:	22/01/2020 10:53:40
Livetime:	60.4s
Process Time:	4
Accelerating Voltage:	15.00kV
Magnification:	32 x
Working Distance:	10.0mm
Specimen Tilt (degrees):	0.0
Elevation (degrees):	35.0
Azimuth (degrees):	0.0
Number Of Channels:	2048
Energy Range (keV):	20 keV
Energy per Channel (eV):	10.0eV
Detector Type Id:	29
Detector Type:	X-Max
Window Type:	SATW
Pulse Pile Up Correction:	Succeeded
Primary Detector:	2617
Primary Detector Serial Number:	77871-X080

Element	Line Type	Apparent Concentration	k Ratio	Wt%	Wt% Sigma	Atomic %	Standard Label	Factory Standard	Standard Calibration Date
C	K series	3.08	0.03076	51.98	1.56	58.44	C Vit	Yes	
N	K series	1.14	0.00204	15.29	2.20	14.74	BN	Yes	
O	K	1.74	0.0058	30.36	1.14	25.62	SiO2	Yes	

	series		5						
Na	K series	0.12	0.00053	1.15	0.14	0.67	Albite	Yes	
Si	K series	0.04	0.00036	0.47	0.07	0.23	SiO2	Yes	
P	K series	0.03	0.00016	0.21	0.06	0.09	GaP	Yes	
Cl	K series	0.05	0.00044	0.55	0.09	0.21	NaCl	Yes	
Total:				100.00		100.00			

Electron Image 4

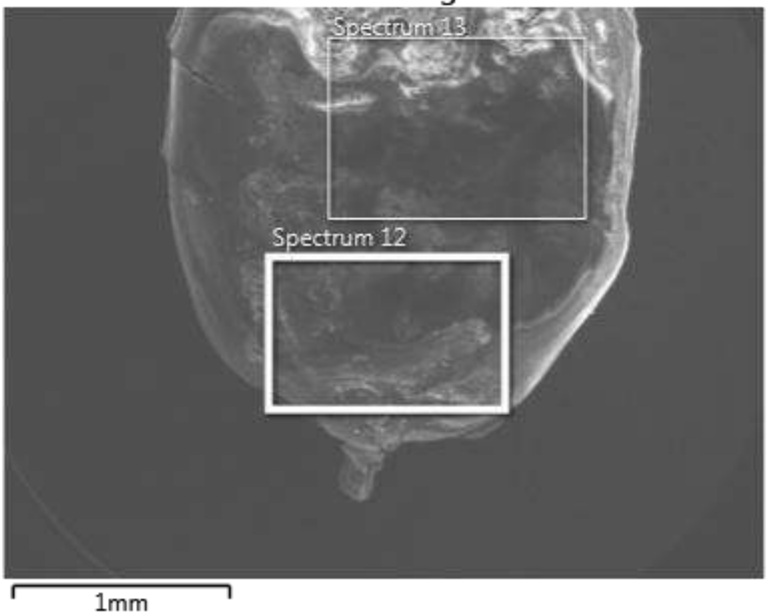

22/01/2020 10:54:47
Project 1
Rumania 4
Dorso 1 15 Kv

Electron Image 4

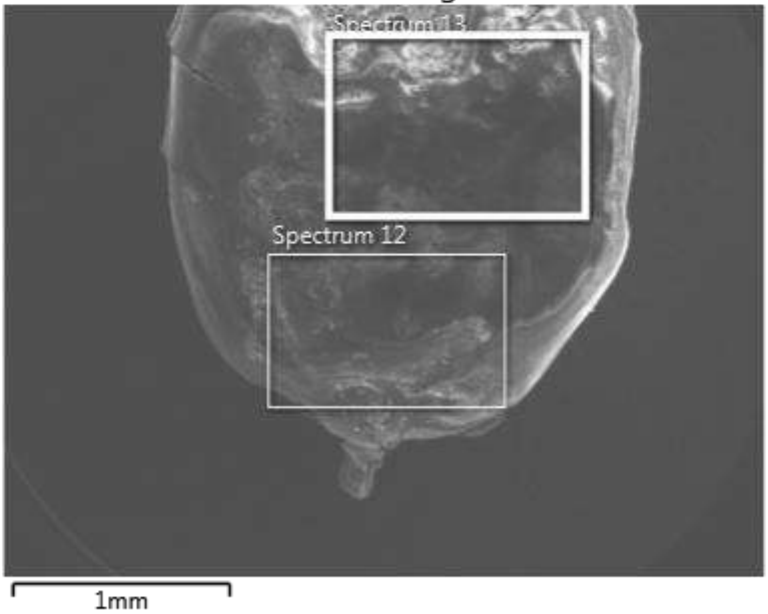

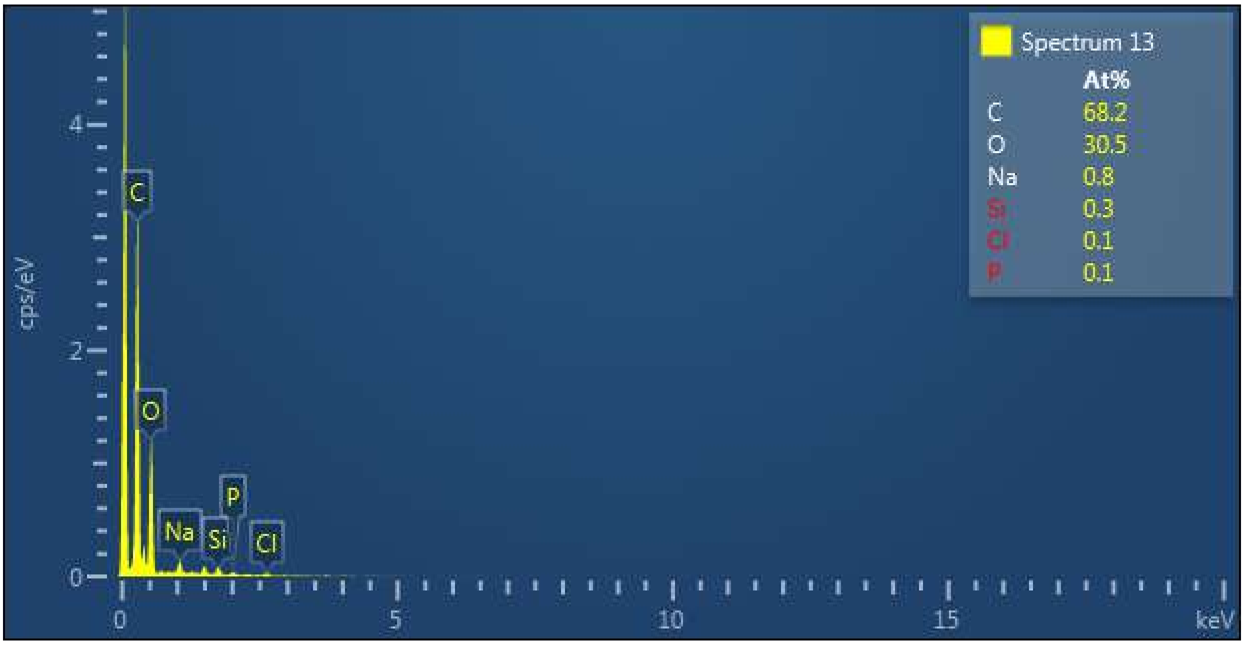

Label:	Spectrum 13
Source:	Acquired
Created:	22/01/2020 10:54:47
Livetime:	60.4s
Process Time:	4
Accelerating Voltage:	15.00kV
Magnification:	32 x
Working Distance:	10.0mm
Specimen Tilt (degrees):	0.0
Elevation (degrees):	35.0
Azimuth (degrees):	0.0
Number Of Channels:	2048
Energy Range (keV):	20 keV
Energy per Channel (eV):	10.0eV
Detector Type Id:	29
Detector Type:	X-Max
Window Type:	SATW
Pulse Pile Up Correction:	Succeeded
Primary Detector:	2617
Primary Detector Serial Number:	77871-X080

Element	Line Type	Apparent Concentration	k Ratio	Wt%	Wt% Sigma	Atomic %	Standard Label	Factory Standard	Standard Calibration Date
C	K series	4.98	0.04980	61.07	0.70	68.19	C Vit	Yes	
O	K series	3.44	0.01157	36.40	0.69	30.51	SiO2	Yes	
Na	K	0.19	0.0008	1.30	0.13	0.76	Albite	Yes	

	series		1						
Si	K series	0.09	0.00072	0.69	0.08	0.33	SiO_2	Yes	
P	K series	0.05	0.00026	0.25	0.07	0.11	GaP	Yes	
Cl	K series	0.04	0.00032	0.29	0.07	0.11	NaCl	Yes	
Total:				100.00		100.00			

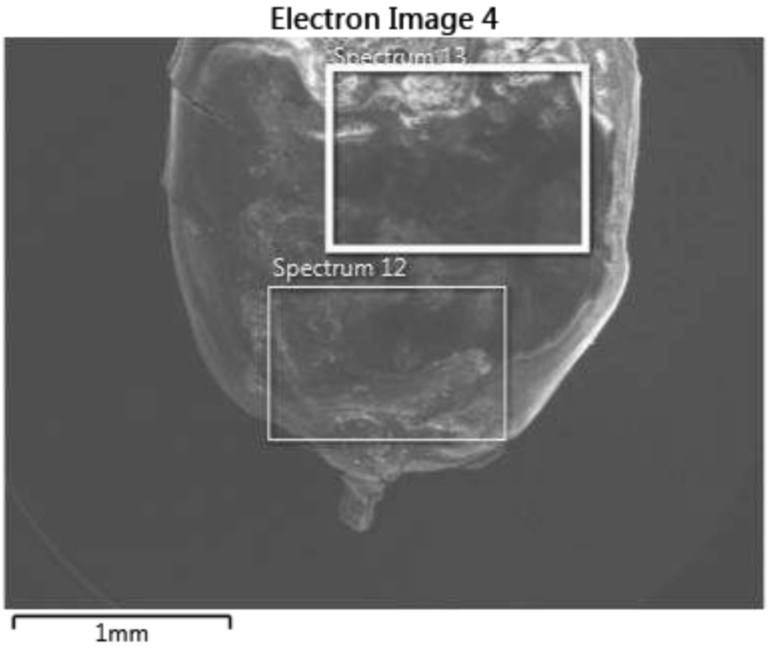

22/01/2020 11:49:38
Project 1
Rumania 4
Dorso 2 15 Kv

Electron Image 5

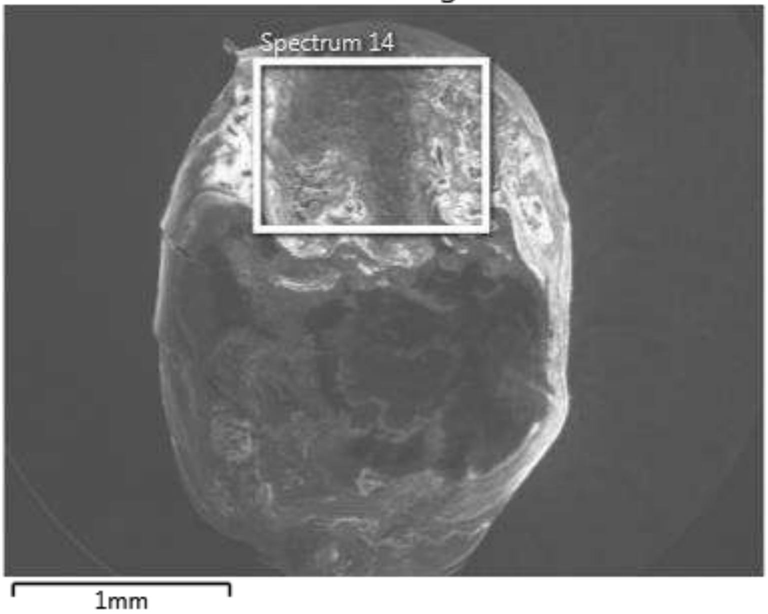

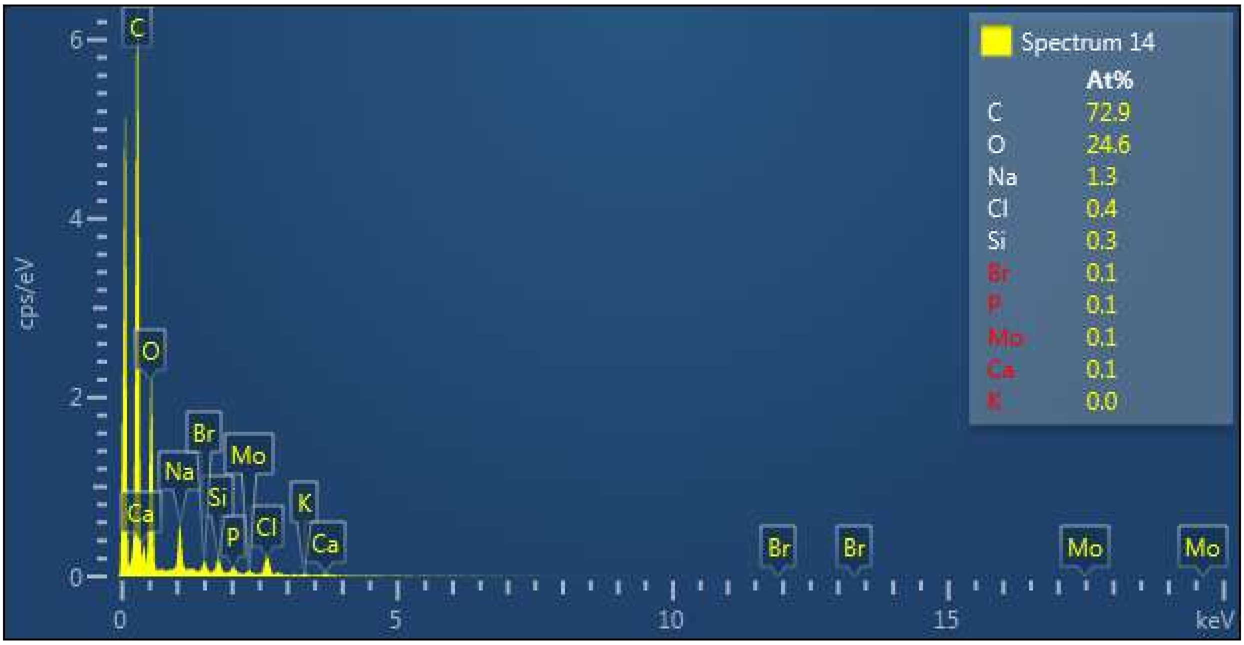

Label:	Spectrum 14
Source:	Acquired
Created:	22/01/2020 11:49:38
Livetime:	60.4s
Process Time:	4
Accelerating Voltage:	15.00kV
Magnification:	32 x
Working Distance:	10.0mm
Specimen Tilt (degrees):	0.0
Elevation (degrees):	35.0
Azimuth (degrees):	0.0
Number Of Channels:	2048
Energy Range (keV):	20 keV
Energy per Channel (eV):	10.0eV
Detector Type Id:	29
Detector Type:	X-Max
Window Type:	SATW
Pulse Pile Up Correction:	Succeeded
Primary Detector:	2617
Primary Detector Serial Number:	77871-X080

Element	Line Type	Apparent Concentration	k Ratio	Wt%	Wt% Sigma	Atomic %	Standard Label	Factory Standard	Standard Calibration Date
C	K series	11.65	0.11645	64.89	0.57	72.92	C Vit	Yes	
O	K series	6.31	0.02125	29.17	0.52	24.61	SiO2	Yes	
Na	K	0.86	0.0036	2.20	0.09	1.29	Albite	Yes	

	series		4						
Si	K series	0.20	0.00155	0.59	0.05	0.29	SiO2	Yes	
P	K series	0.14	0.00077	0.29	0.04	0.13	GaP	Yes	
Cl	K series	0.36	0.00313	1.15	0.07	0.44	NaCl	Yes	
K	K series	0.05	0.00039	0.14	0.04	0.05	KBr	Yes	
Ca	K series	0.05	0.00044	0.15	0.04	0.05	Wollastonite	Yes	
Br	L series	0.22	0.00196	0.85	0.09	0.14	KBr	Yes	
Mo	L series	0.14	0.00141	0.56	0.12	0.08	Mo	Yes	
Total:				100.00		100.00			

Electron Image 5

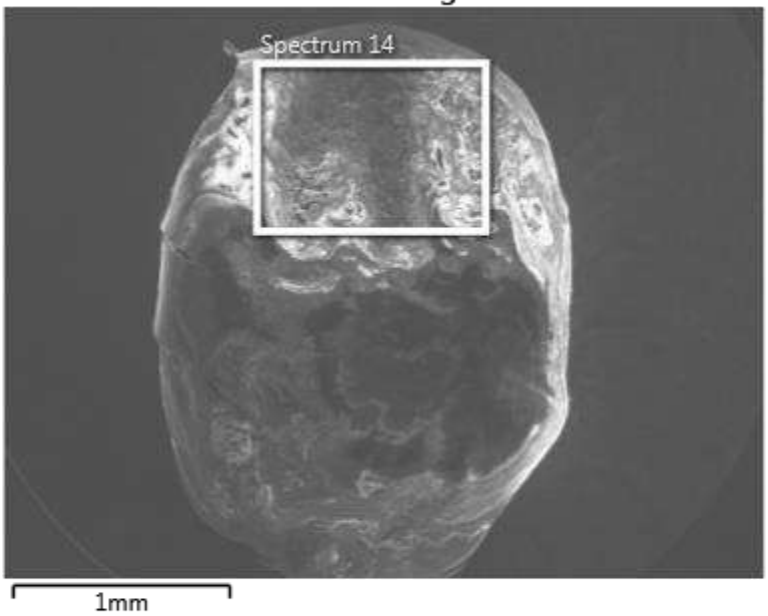

22/01/2020 11:52:36
Project 1
Rumania 5
Dorso 1 15 Kv

Electron Image 6

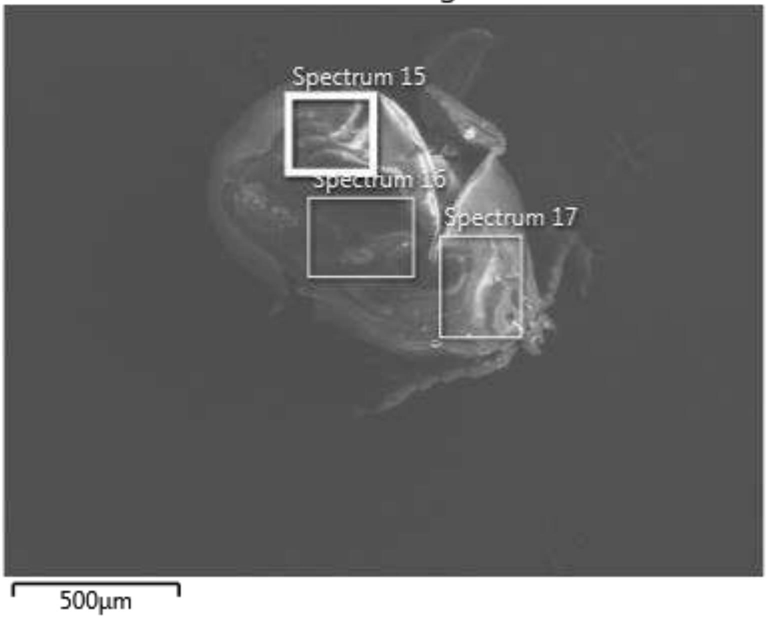

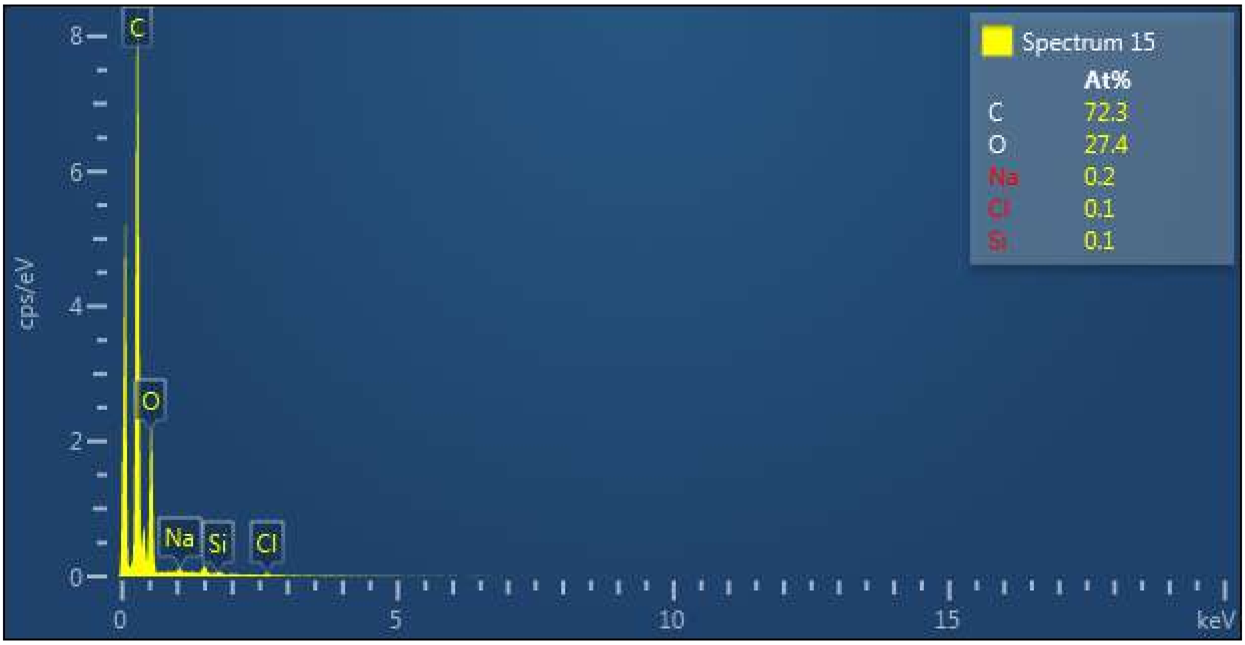

Label:	Spectrum 15
Source:	Acquired
Created:	22/01/2020 11:52:36
Livetime:	60.5s
Process Time:	4
Accelerating Voltage:	15.00kV
Magnification:	49 x
Working Distance:	10.0mm
Specimen Tilt (degrees):	0.0
Elevation (degrees):	35.0
Azimuth (degrees):	0.0
Number Of Channels:	2048
Energy Range (keV):	20 keV
Energy per Channel (eV):	10.0eV
Detector Type Id:	29
Detector Type:	X-Max
Window Type:	SATW
Pulse Pile Up Correction:	Succeeded
Primary Detector:	2617
Primary Detector Serial Number:	77871-X080

Element	Line Type	Apparent Concentration	k Ratio	Wt%	Wt% Sigma	Atomic %	Standard Label	Factory Standard	Standard Calibration Date
C	K series	13.23	0.13226	65.98	0.48	72.25	C Vit	Yes	
O	K series	6.40	0.02152	33.34	0.47	27.41	SiO2	Yes	
Na	K	0.11	0.0004	0.35	0.07	0.20	Albite	Yes	

	series		7						
Si	K series	0.04	0.00034	0.15	0.04	0.07	SiO2	Yes	
Cl	K series	0.05	0.00045	0.19	0.04	0.07	NaCl	Yes	
Total:				100.00		100.00			

Electron Image 6

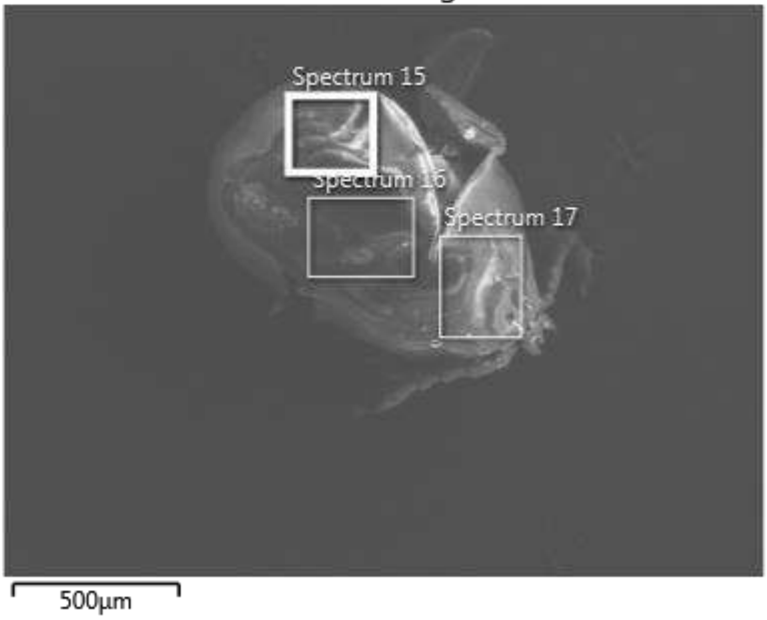

22/01/2020 11:53:50
Project 1
Rumania 5
Dorso 1 15 Kv

Electron Image 6

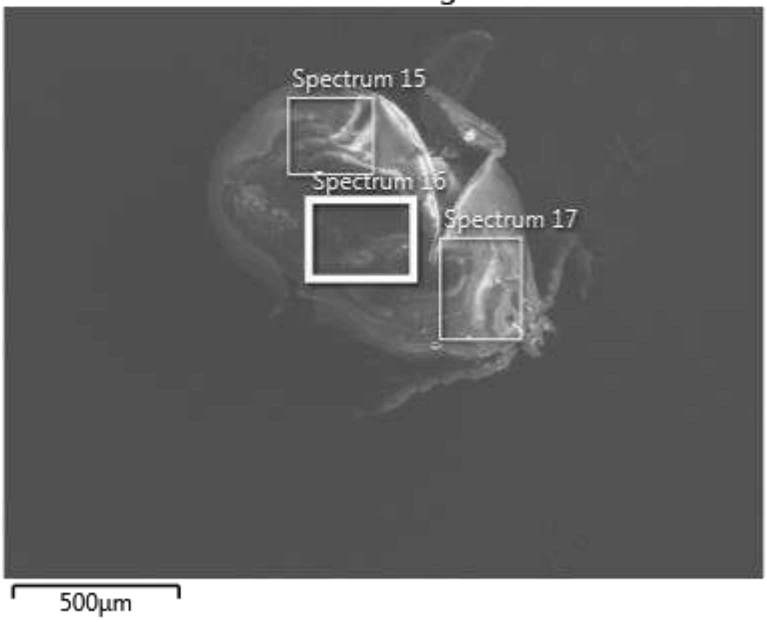

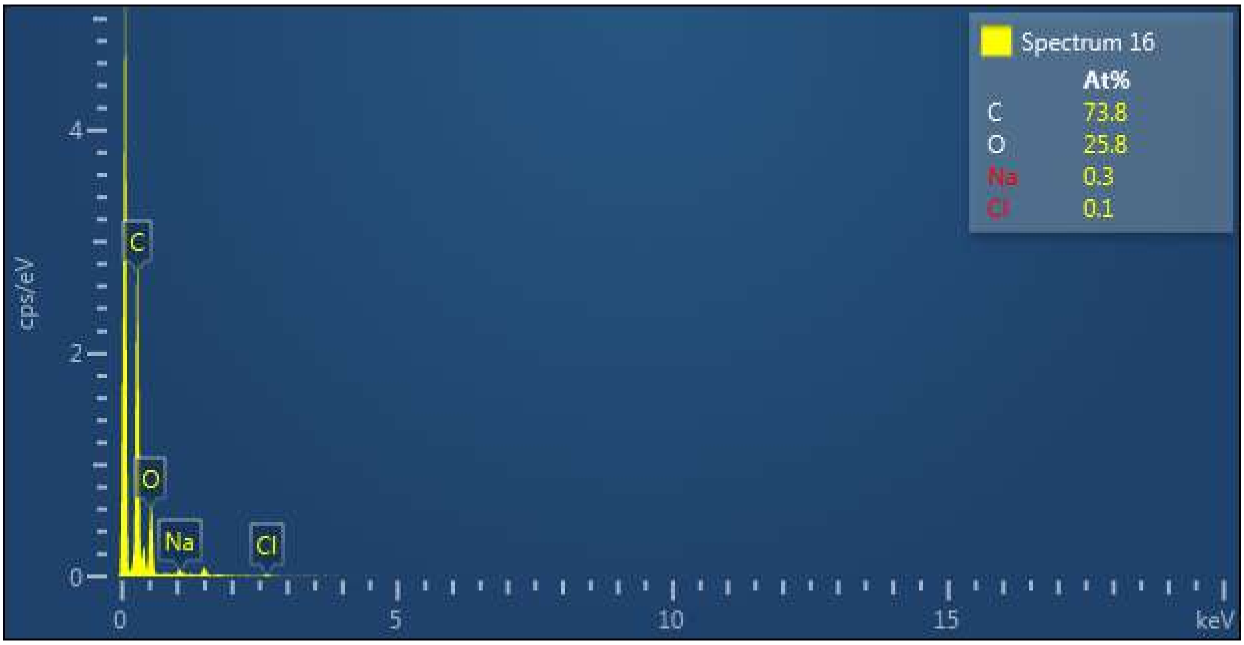

Label:	Spectrum 16
Source:	Acquired
Created:	22/01/2020 11:53:50
Livetime:	60.4s
Process Time:	4
Accelerating Voltage:	15.00kV
Magnification:	49 x
Working Distance:	10.0mm
Specimen Tilt (degrees):	0.0
Elevation (degrees):	35.0
Azimuth (degrees):	0.0
Number Of Channels:	2048
Energy Range (keV):	20 keV
Energy per Channel (eV):	10.0eV
Detector Type Id:	29
Detector Type:	X-Max
Window Type:	SATW
Pulse Pile Up Correction:	Succeeded
Primary Detector:	2617
Primary Detector Serial Number:	77871-X080

Element	Line Type	Apparent Concentration	k Ratio	Wt%	Wt% Sigma	Atomic %	Standard Label	Factory Standard	Standard Calibration Date
C	K series	4.44	0.04438	67.63	0.84	73.75	C Vit	Yes	
O	K series	1.91	0.00643	31.53	0.84	25.82	SiO2	Yes	
Na	K	0.06	0.0002	0.59	0.13	0.34	Albite	Yes	

	series		6						
Cl	K series	0.02	0.00019	0.25	0.07	0.09	NaCl	Yes	
Total:				100.00		100.00			

Electron Image 6

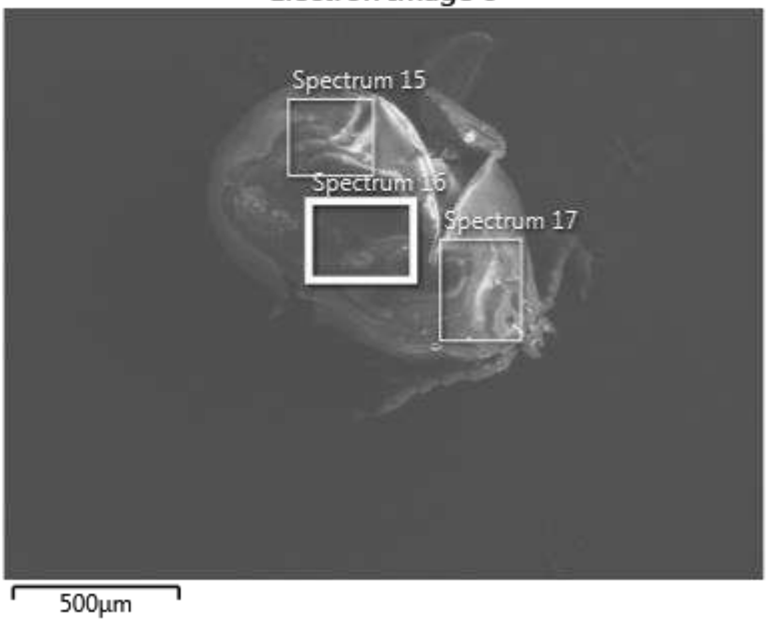

22/01/2020 11:55:01
Project 1
Rumania 5
Dorso 1 15 Kv

Electron Image 6

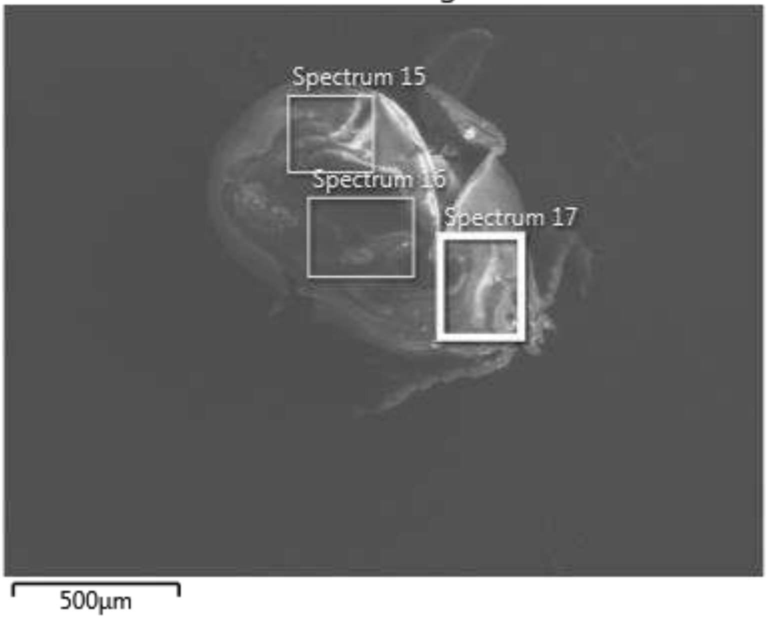

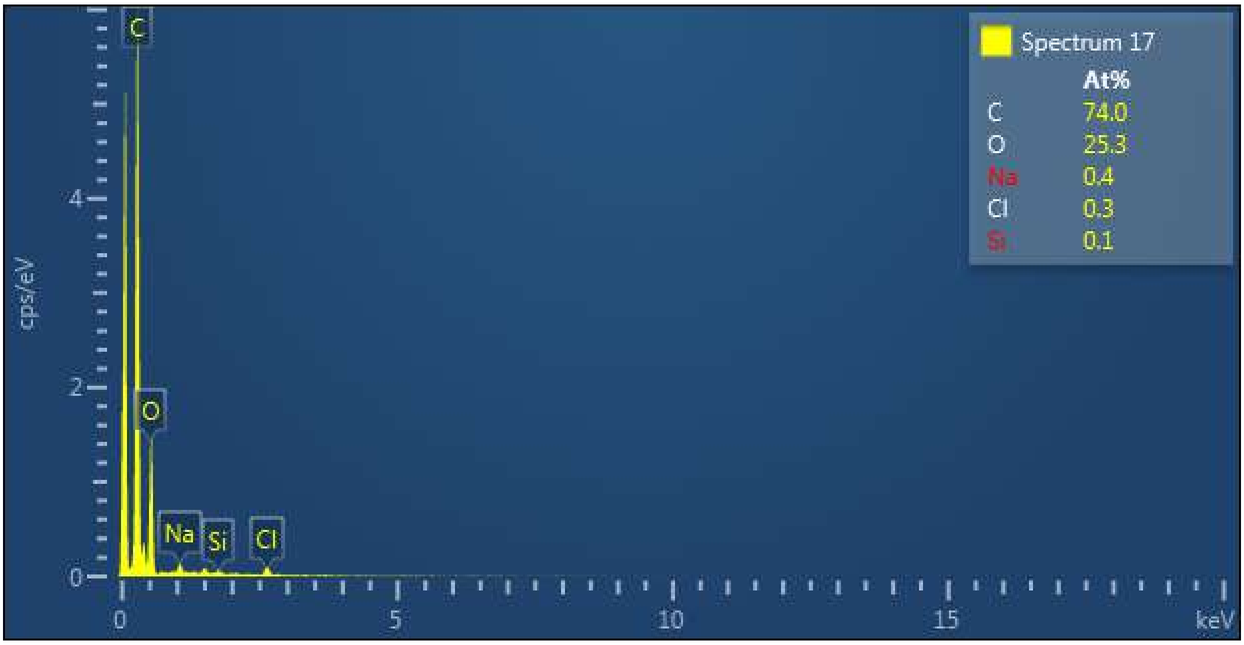

Label:	Spectrum 17
Source:	Acquired
Created:	22/01/2020 11:55:01
Livetime:	60.5s
Process Time:	4
Accelerating Voltage:	15.00kV
Magnification:	49 x
Working Distance:	10.0mm
Specimen Tilt (degrees):	0.0
Elevation (degrees):	35.0
Azimuth (degrees):	0.0
Number Of Channels:	2048
Energy Range (keV):	20 keV
Energy per Channel (eV):	10.0eV
Detector Type Id:	29
Detector Type:	X-Max
Window Type:	SATW
Pulse Pile Up Correction:	Succeeded
Primary Detector:	2617
Primary Detector Serial Number:	77871-X080

Element	Line Type	Apparent Concentration	k Ratio	Wt%	Wt% Sigma	Atomic %	Standard Label	Factory Standard	Standard Calibration Date
C	K series	9.45	0.09449	67.59	0.54	73.95	C Vit	Yes	
O	K series	4.24	0.01425	30.80	0.54	25.30	SiO2	Yes	
Na	K	0.16	0.0006	0.64	0.08	0.37	Albite	Yes	

	series		7						
Si	K series	0.04	0.00034	0.20	0.05	0.09	SiO2	Yes	
Cl	K series	0.15	0.00133	0.76	0.07	0.28	NaCl	Yes	
Total:				100.00		100.00			

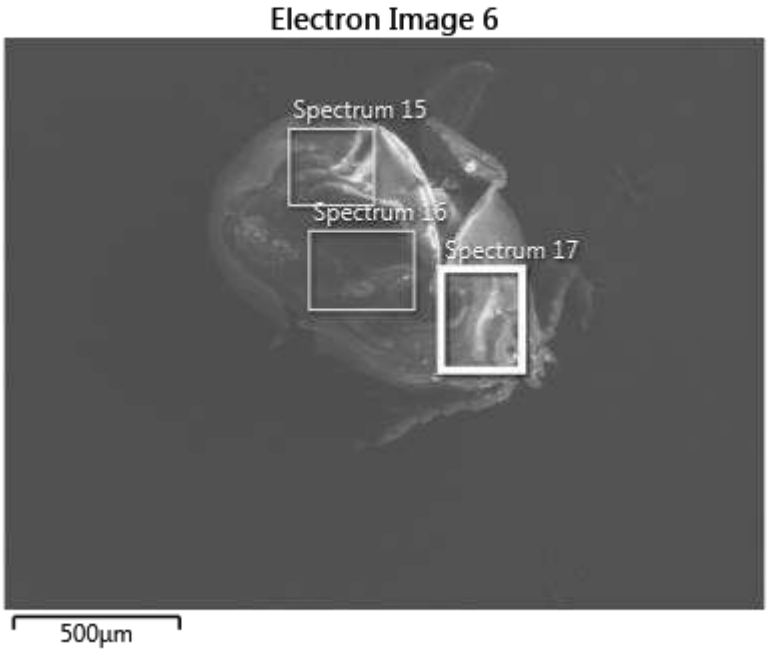

22/01/2020 11:57:31
Project 1
Rumania 6
Dorso 1 15 Kv

Electron Image 7

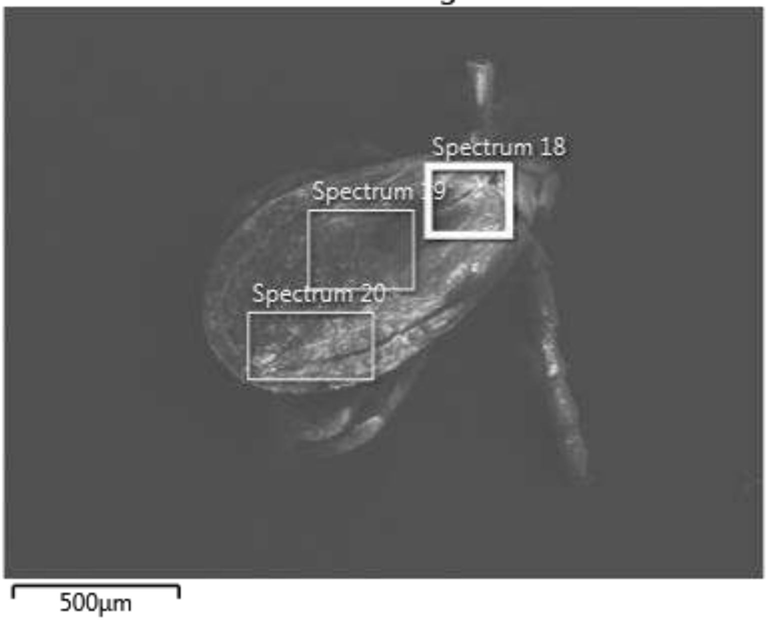

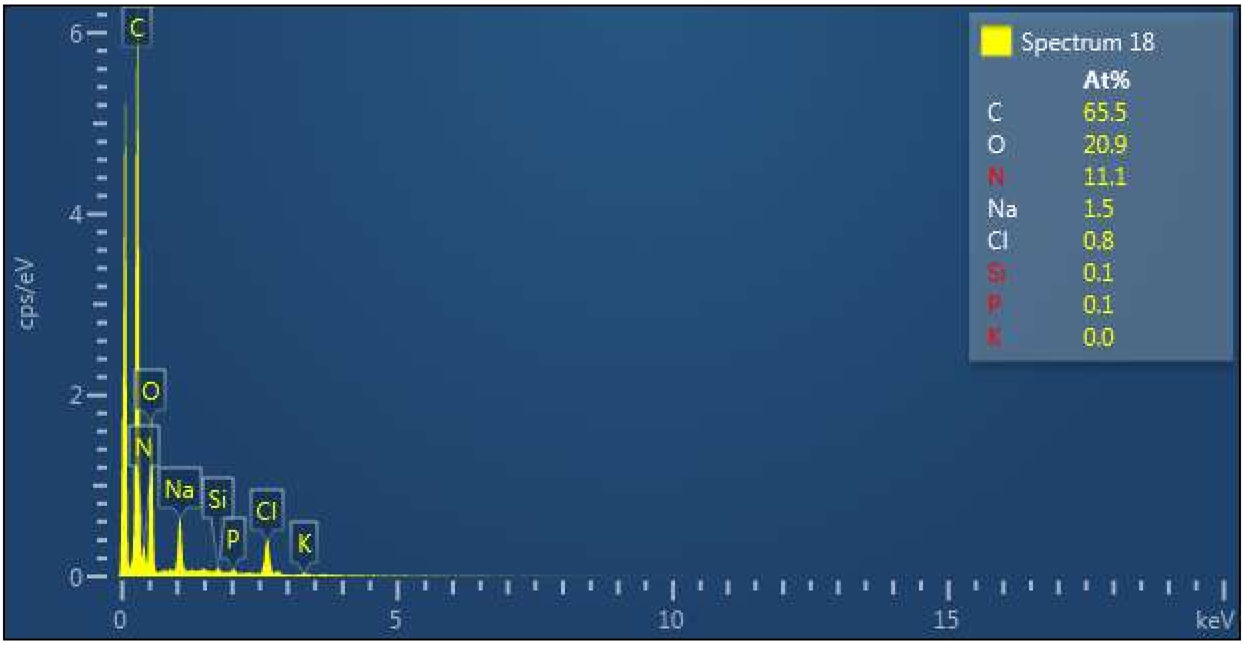

Label:	Spectrum 18
Source:	Acquired
Created:	22/01/2020 11:57:31
Livetime:	60.3s
Process Time:	4
Accelerating Voltage:	15.00kV
Magnification:	49 x
Working Distance:	10.0mm
Specimen Tilt (degrees):	0.0
Elevation (degrees):	35.0
Azimuth (degrees):	0.0
Number Of Channels:	2048
Energy Range (keV):	20 keV
Energy per Channel (eV):	10.0eV
Detector Type Id:	29
Detector Type:	X-Max
Window Type:	SATW
Pulse Pile Up Correction:	Succeeded
Primary Detector:	2617
Primary Detector Serial Number:	77871-X080

Element	Line Type	Apparent Concentration	k Ratio	Wt%	Wt% Sigma	Atomic %	Standard Label	Factory Standard	Standard Calibration Date
C	K series	11.05	0.11048	58.50	0.98	65.52	C Vit	Yes	
N	K series	2.68	0.00478	11.55	1.25	11.10	BN	Yes	
O	K	4.98	0.0167	24.88	0.58	20.92	SiO2	Yes	

	series		6						
Na	K series	1.03	0.00434	2.49	0.10	1.46	Albite	Yes	
Si	K series	0.06	0.00051	0.18	0.04	0.09	SiO2	Yes	
P	K series	0.10	0.00055	0.20	0.04	0.08	GaP	Yes	
Cl	K series	0.68	0.00597	2.06	0.08	0.78	NaCl	Yes	
K	K series	0.04	0.00038	0.13	0.04	0.04	KBr	Yes	
Total:				100.00		100.00			

Electron Image 7

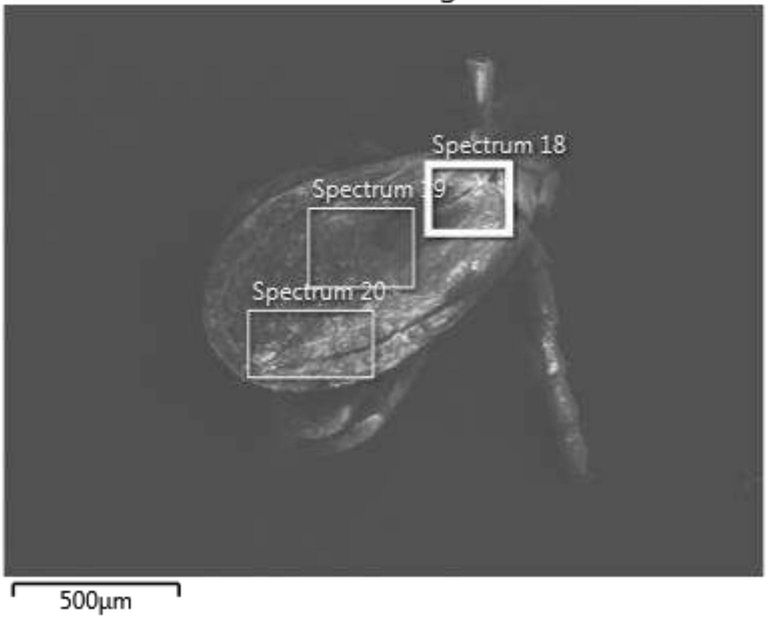

22/01/2020 11:58:45
Project 1
Rumania 6
Dorso 1 15 Kv

Electron Image 7

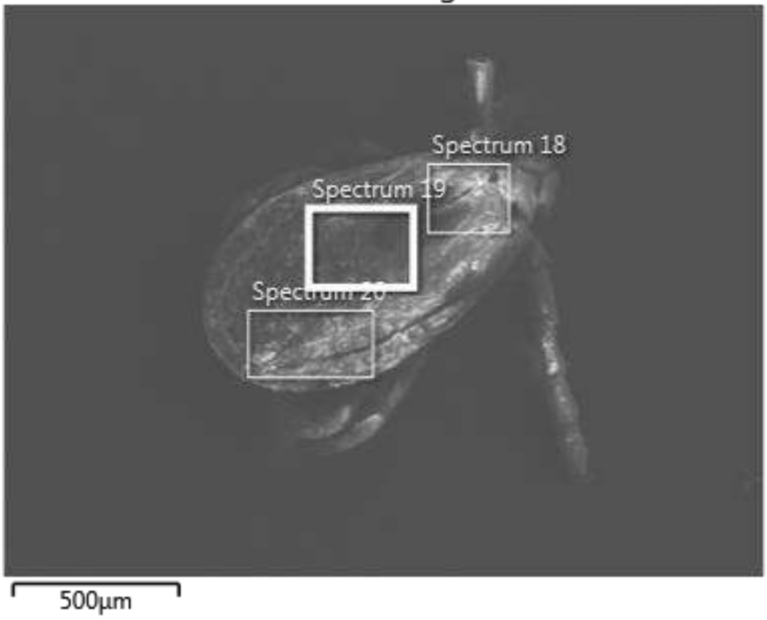

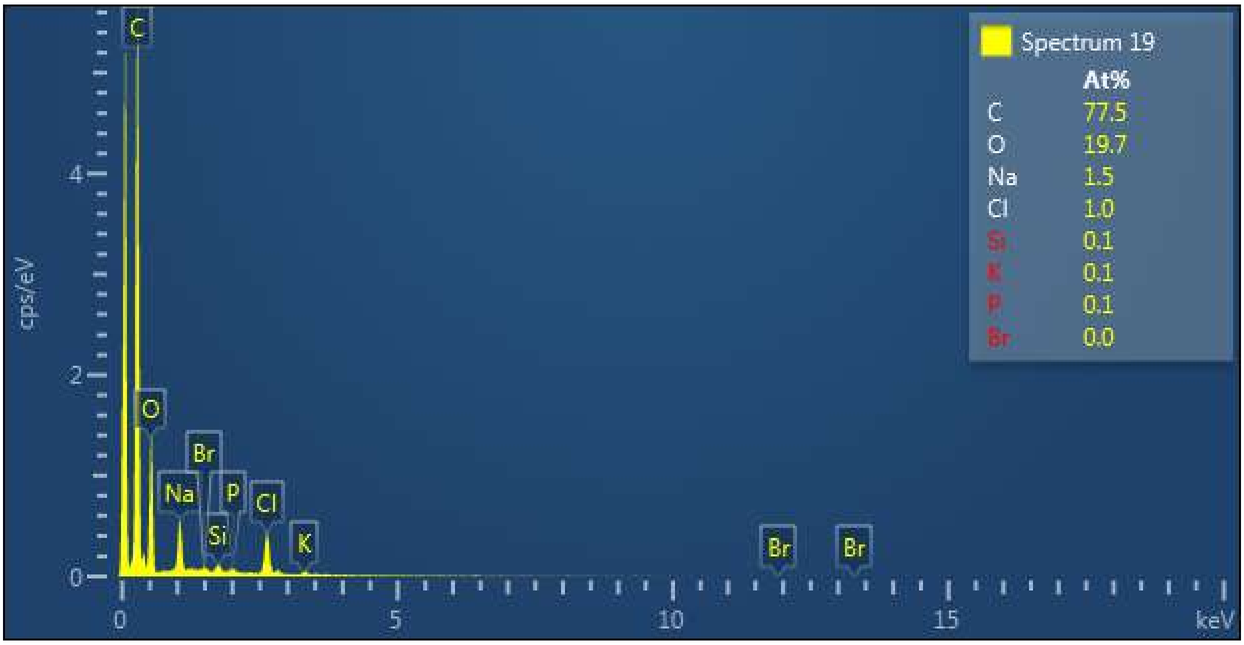

Label:	Spectrum 19
Source:	Acquired
Created:	22/01/2020 11:58:45
Livetime:	60.4s
Process Time:	4
Accelerating Voltage:	15.00kV
Magnification:	49 x
Working Distance:	10.0mm
Specimen Tilt (degrees):	0.0
Elevation (degrees):	35.0
Azimuth (degrees):	0.0
Number Of Channels:	2048
Energy Range (keV):	20 keV
Energy per Channel (eV):	10.0eV
Detector Type Id:	29
Detector Type:	X-Max
Window Type:	SATW
Pulse Pile Up Correction:	Succeeded
Primary Detector:	2617
Primary Detector Serial Number:	77871-X080

Element	Line Type	Apparent Concentration	k Ratio	Wt%	Wt% Sigma	Atomic %	Standard Label	Factory Standard	Standard Calibration Date
C	K series	9.89	0.09894	70.01	0.55	77.48	C Vit	Yes	
O	K series	3.93	0.01323	23.66	0.51	19.66	SiO2	Yes	
Na	K	0.86	0.0036	2.57	0.11	1.48	Albite	Yes	

	series		4						
Si	K series	0.09	0.00069	0.31	0.05	0.15	SiO2	Yes	
P	K series	0.06	0.00036	0.16	0.05	0.07	GaP	Yes	
Cl	K series	0.71	0.00623	2.72	0.10	1.02	NaCl	Yes	
K	K series	0.07	0.00062	0.27	0.06	0.09	KBr	Yes	
Br	L series	0.06	0.00056	0.29	0.09	0.05	KBr	Yes	
Total:				100.00		100.00			

Electron Image 7

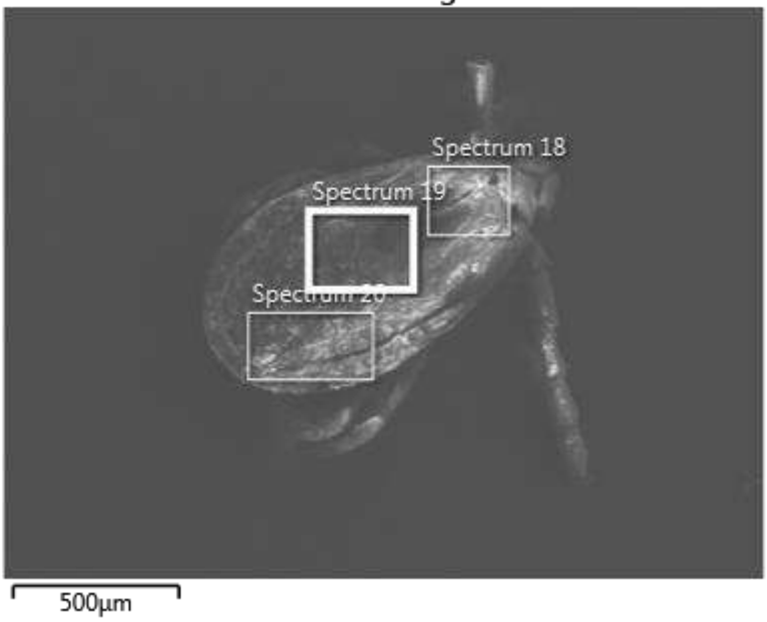

22/01/2020 11:59:57
Project 1
Rumania 6
Dorso 1 15 Kv

Electron Image 7

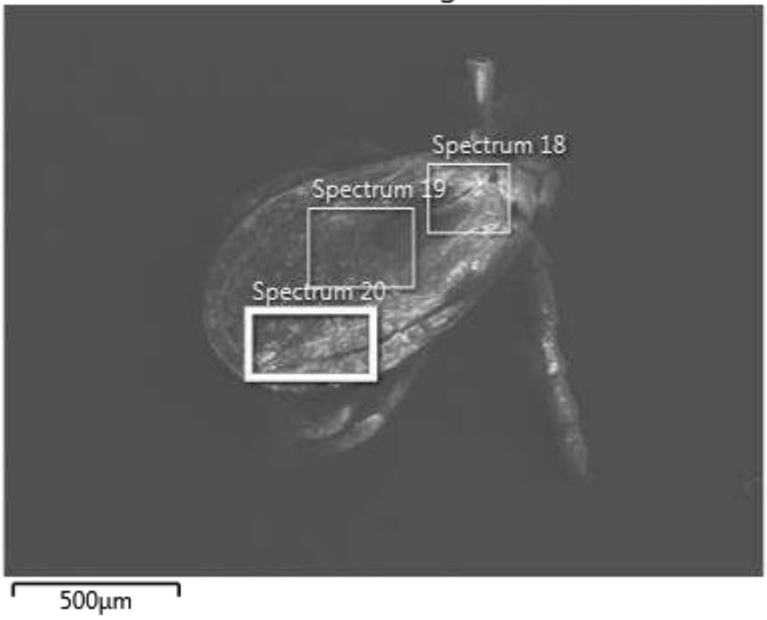

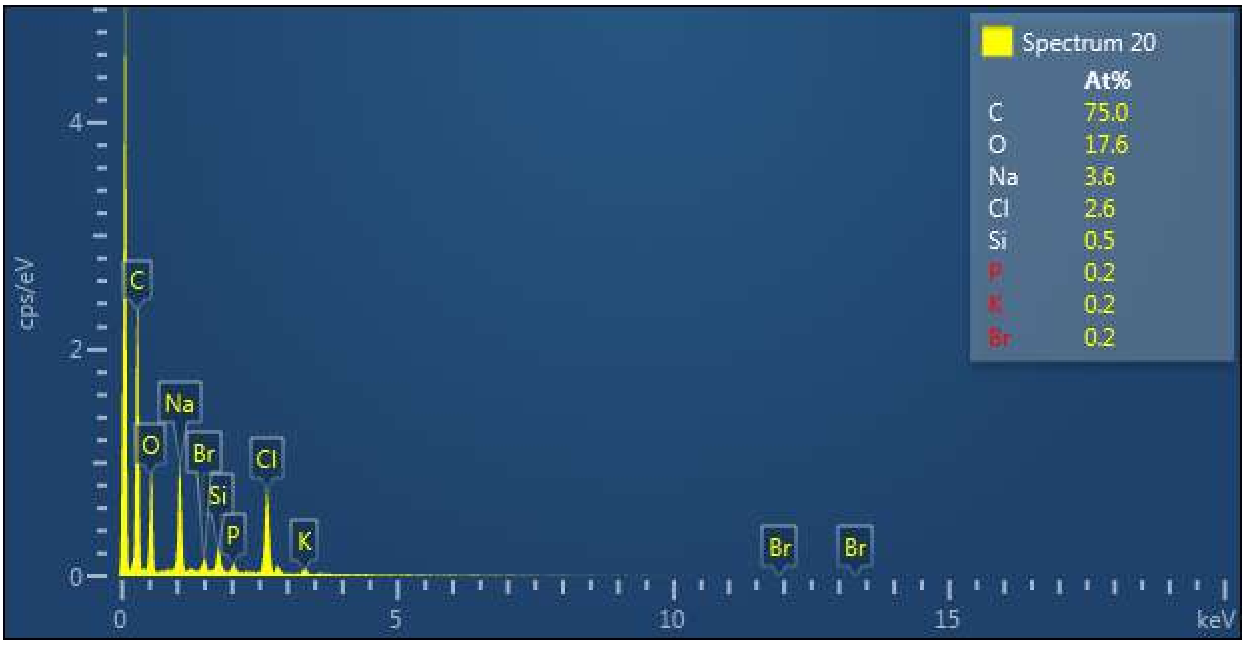

Label:	Spectrum 20
Source:	Acquired
Created:	22/01/2020 11:59:57
Livetime:	60.1s
Process Time:	4
Accelerating Voltage:	15.00kV
Magnification:	49 x
Working Distance:	10.0mm
Specimen Tilt (degrees):	0.0
Elevation (degrees):	35.0
Azimuth (degrees):	0.0
Number Of Channels:	2048
Energy Range (keV):	20 keV
Energy per Channel (eV):	10.0eV
Detector Type Id:	29
Detector Type:	X-Max
Window Type:	SATW
Pulse Pile Up Correction:	Succeeded
Primary Detector:	2617
Primary Detector Serial Number:	77871-X080

Element	Line Type	Apparent Concentration	k Ratio	Wt%	Wt% Sigma	Atomic %	Standard Label	Factory Standard	Standard Calibration Date
C	K series	4.35	0.04354	64.11	0.74	74.97	C Vit	Yes	
O	K series	2.60	0.00875	20.07	0.57	17.62	SiO2	Yes	
Na	K	1.62	0.0068	5.96	0.19	3.64	Albite	Yes	

	series		5						
Si	K series	0.23	0.00186	1.09	0.08	0.55	SiO2	Yes	
P	K series	0.16	0.00092	0.53	0.07	0.24	GaP	Yes	
Cl	K series	1.31	0.01146	6.46	0.19	2.56	NaCl	Yes	
K	K series	0.13	0.00106	0.60	0.08	0.22	KBr	Yes	
Br	L series	0.20	0.00175	1.17	0.13	0.21	KBr	Yes	
Total:				100.00		100.00			

Electron Image 7

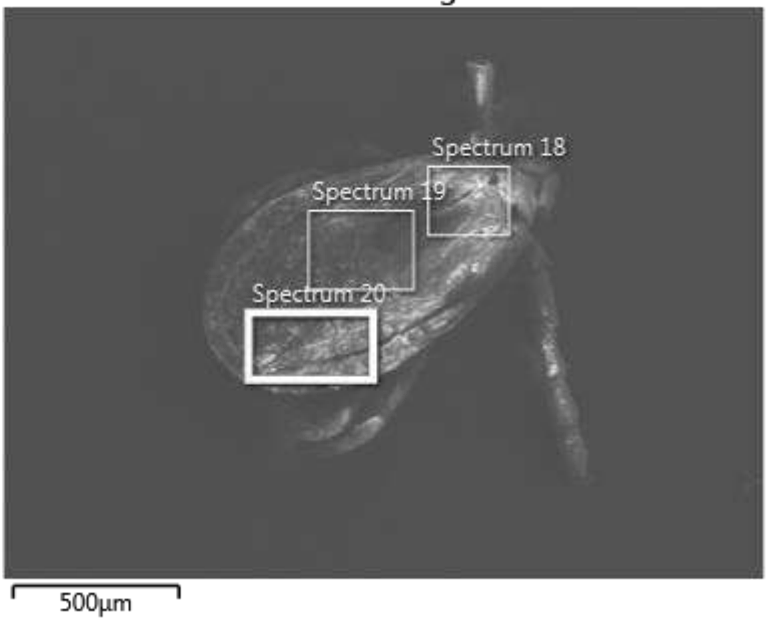

22/01/2020 12:02:29
Project 1
Rumania 7
Dorso 1 15 Kv

Electron Image 8

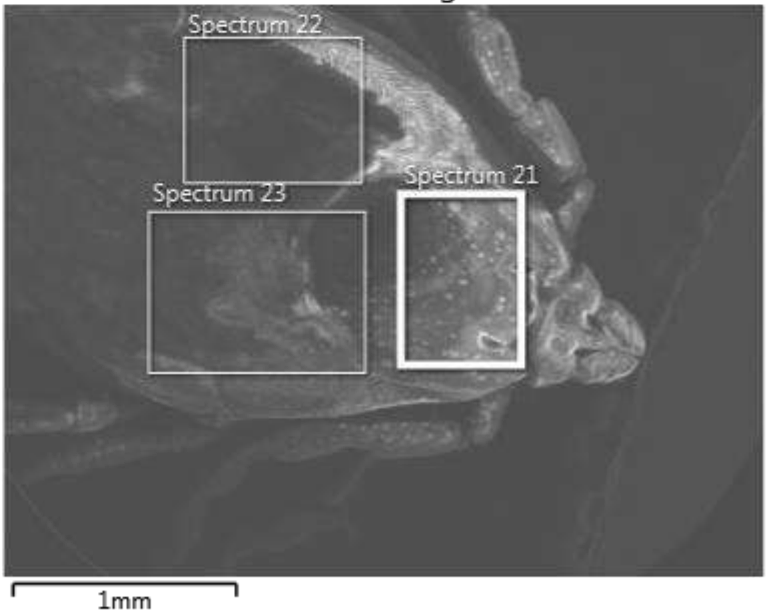

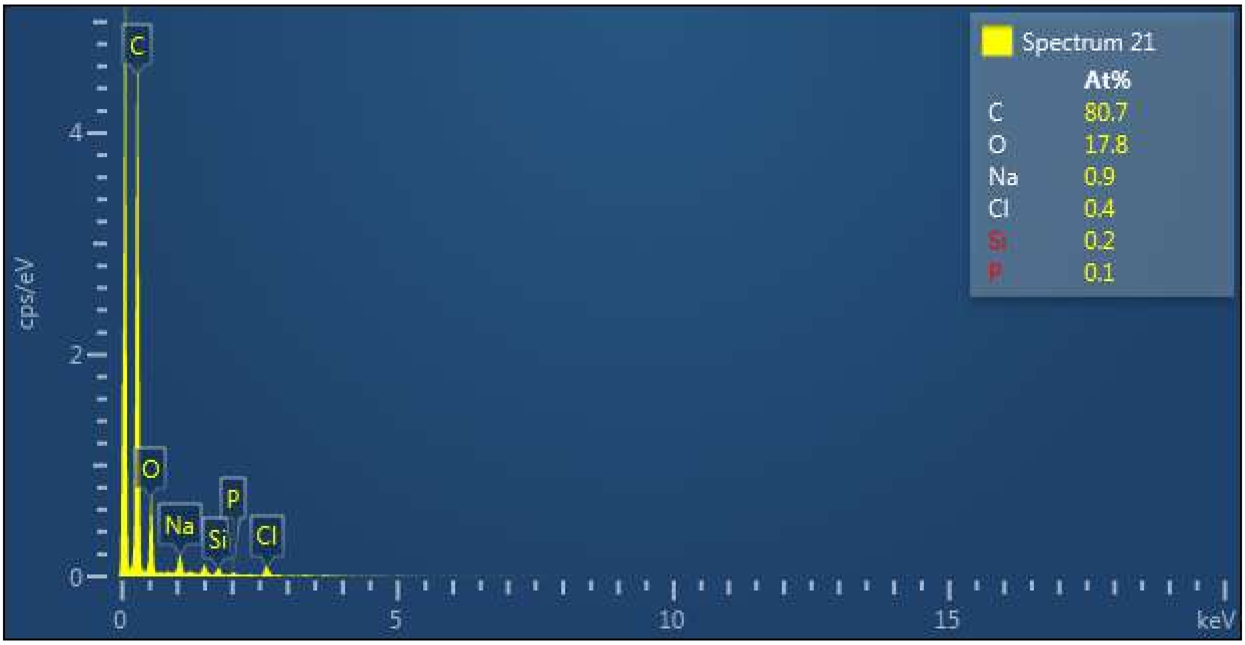

Label:	Spectrum 21
Source:	Acquired
Created:	22/01/2020 12:02:29
Livetime:	60.0s
Process Time:	4
Accelerating Voltage:	15.00kV
Magnification:	33 x
Working Distance:	10.0mm
Specimen Tilt (degrees):	0.0
Elevation (degrees):	35.0
Azimuth (degrees):	0.0
Number Of Channels:	2048
Energy Range (keV):	20 keV
Energy per Channel (eV):	10.0eV
Detector Type Id:	29
Detector Type:	X-Max
Window Type:	SATW
Pulse Pile Up Correction:	Succeeded
Primary Detector:	2617
Primary Detector Serial Number:	77871-X080

Element	Line Type	Apparent Concentration	k Ratio	Wt%	Wt% Sigma	Atomic %	Standard Label	Factory Standard	Standard Calibration Date
C	K series	7.58	0.07583	74.80	0.61	80.66	C Vit	Yes	
O	K series	2.00	0.00672	22.03	0.60	17.83	SiO2	Yes	
Na	K	0.29	0.0012	1.52	0.12	0.85	Albite	Yes	

	series		2						
Si	K series	0.08	0.00061	0.48	0.06	0.22	SiO2	Yes	
P	K series	0.04	0.00024	0.19	0.06	0.08	GaP	Yes	
Cl	K series	0.14	0.00126	0.98	0.09	0.36	NaCl	Yes	
Total:				100.00		100.00			

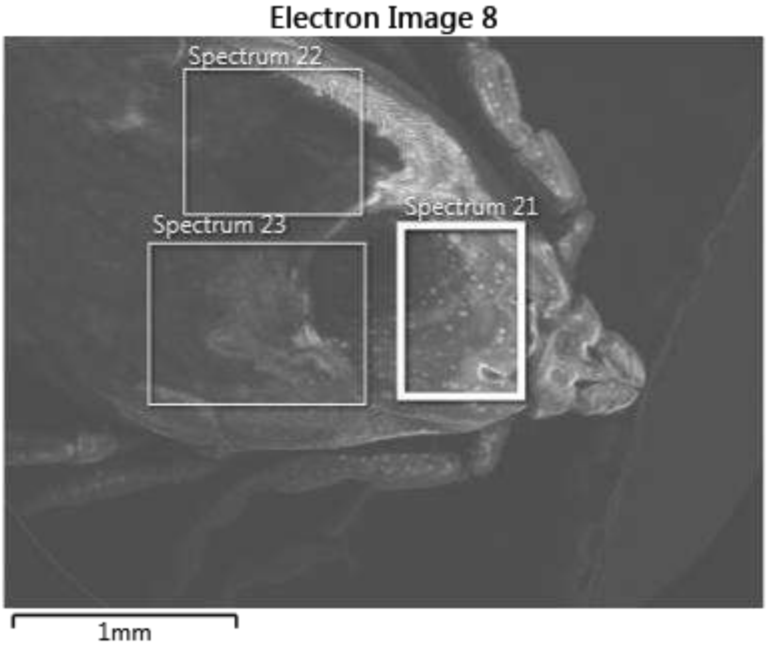

22/01/2020 12:03:39
Project 1
Rumania 7
Dorso 1 15 Kv

Electron Image 8

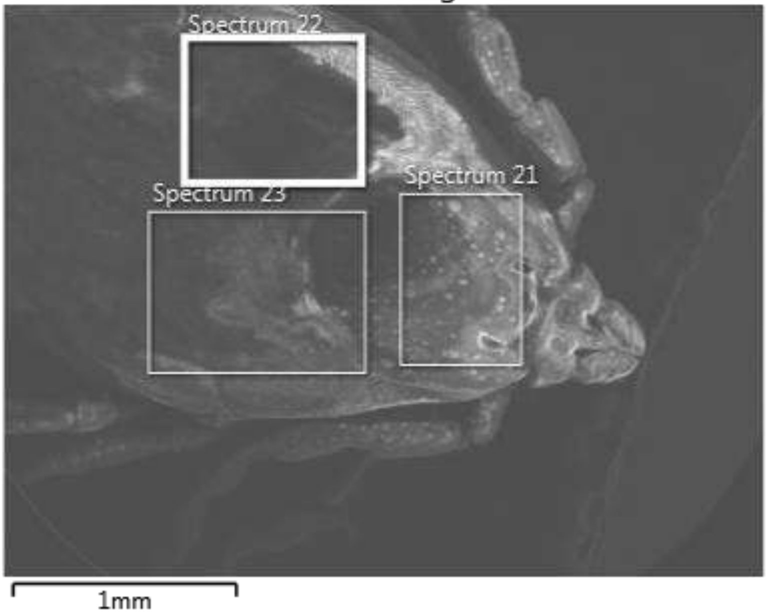

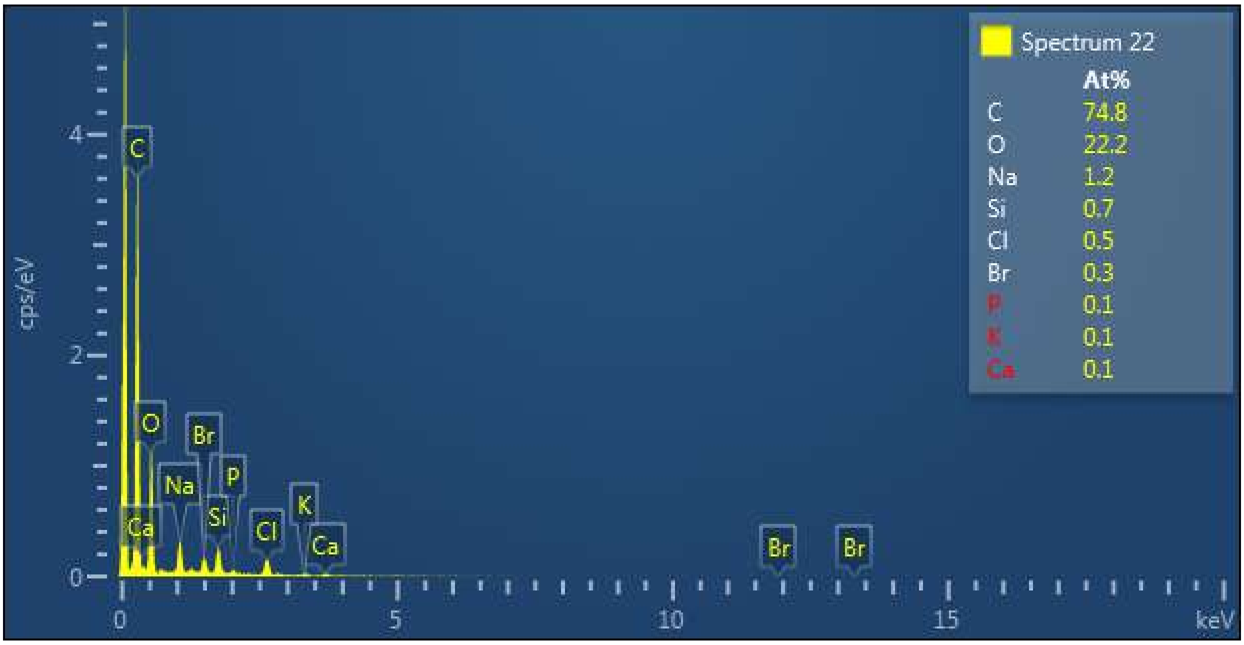

Label:	Spectrum 22
Source:	Acquired
Created:	22/01/2020 12:03:39
Livetime:	60.1s
Process Time:	4
Accelerating Voltage:	15.00kV
Magnification:	33 x
Working Distance:	10.0mm
Specimen Tilt (degrees):	0.0
Elevation (degrees):	35.0
Azimuth (degrees):	0.0
Number Of Channels:	2048
Energy Range (keV):	20 keV
Energy per Channel (eV):	10.0eV
Detector Type Id:	29
Detector Type:	X-Max
Window Type:	SATW
Pulse Pile Up Correction:	Succeeded
Primary Detector:	2617
Primary Detector Serial Number:	77871-X080

Element	Line Type	Apparent Concentration	k Ratio	Wt%	Wt% Sigma	Atomic %	Standard Label	Factory Standard	Standard Calibration Date
C	K series	6.81	0.06806	66.46	0.68	74.78	C Vit	Yes	
O	K series	3.35	0.01128	26.33	0.63	22.24	SiO2	Yes	
Na	K	0.51	0.0021	2.09	0.12	1.23	Albite	Yes	

	series		4						
Si	K series	0.28	0.00223	1.40	0.08	0.67	SiO2	Yes	
P	K series	0.07	0.00038	0.24	0.06	0.10	GaP	Yes	
Cl	K series	0.26	0.00229	1.38	0.09	0.53	NaCl	Yes	
K	K series	0.05	0.00044	0.26	0.06	0.09	KBr	Yes	
Ca	K series	0.05	0.00042	0.25	0.06	0.08	Wollastonite	Yes	
Br	L series	0.25	0.00227	1.60	0.15	0.27	KBr	Yes	
Total:				100.00		100.00			

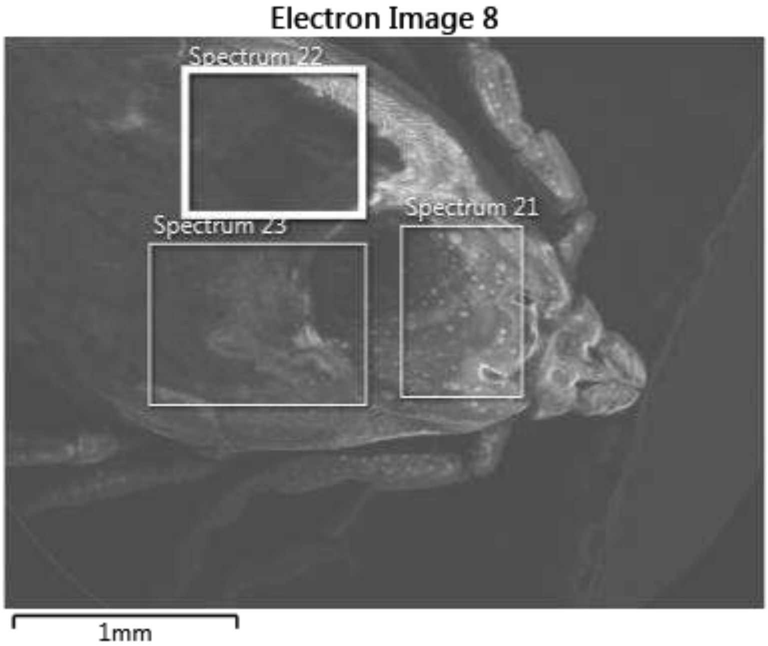

22/01/2020 12:04:46
Project 1
Rumania 7
Dorso 1 15 Kv

Electron Image 8

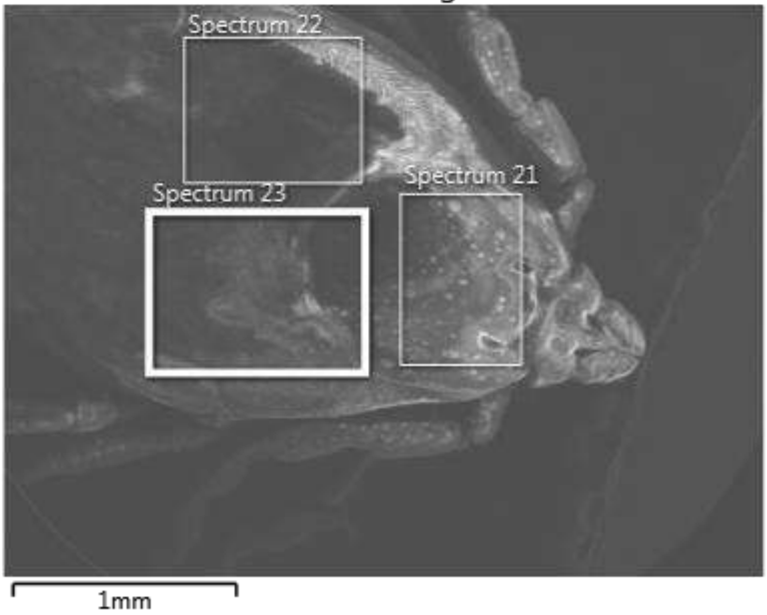

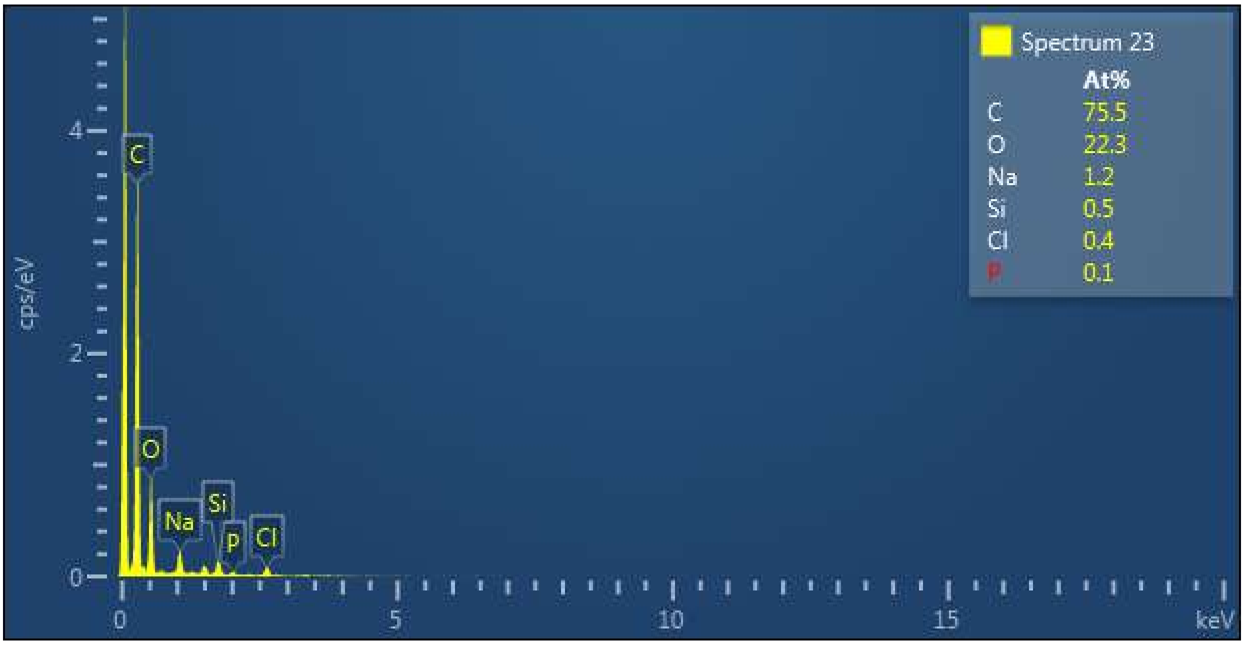

Label:	Spectrum 23
Source:	Acquired
Created:	22/01/2020 12:04:46
Livetime:	60.5s
Process Time:	4
Accelerating Voltage:	15.00kV
Magnification:	33 x
Working Distance:	10.0mm
Specimen Tilt (degrees):	0.0
Elevation (degrees):	35.0
Azimuth (degrees):	0.0
Number Of Channels:	2048
Energy Range (keV):	20 keV
Energy per Channel (eV):	10.0eV
Detector Type Id:	29
Detector Type:	X-Max
Window Type:	SATW
Pulse Pile Up Correction:	Succeeded
Primary Detector:	2617
Primary Detector Serial Number:	77871-X080

Element	Line Type	Apparent Concentration	k Ratio	Wt%	Wt% Sigma	Atomic %	Standard Label	Factory Standard	Standard Calibration Date
C	K series	5.78	0.05777	68.59	0.65	75.51	C Vit	Yes	
O	K series	2.45	0.00824	26.97	0.65	22.29	SiO2	Yes	
Na	K	0.36	0.0015	2.08	0.14	1.20	Albite	Yes	

	serie s		1						
Si	K serie s	0.16	0.0012 4	1.08	0.09	0.51	SiO2	Yes	
P	K serie s	0.06	0.0003 3	0.28	0.06	0.12	GaP	Yes	
Cl	K serie s	0.13	0.0011 7	0.99	0.09	0.37	NaCl	Yes	
Total:				100.0 0		100.0 0			

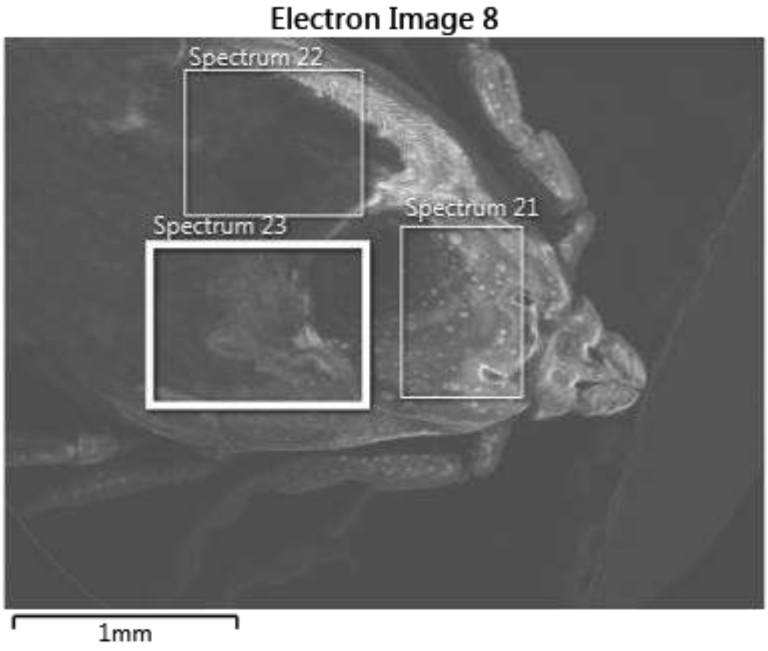

22/01/2020 12:06:47
Project 1
Rumania 7
Dorso 2 15 Kv

Electron Image 9

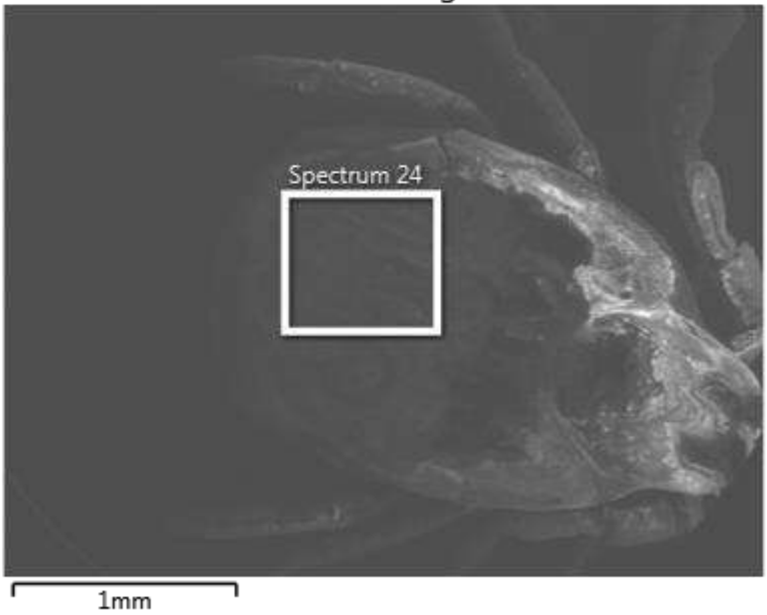

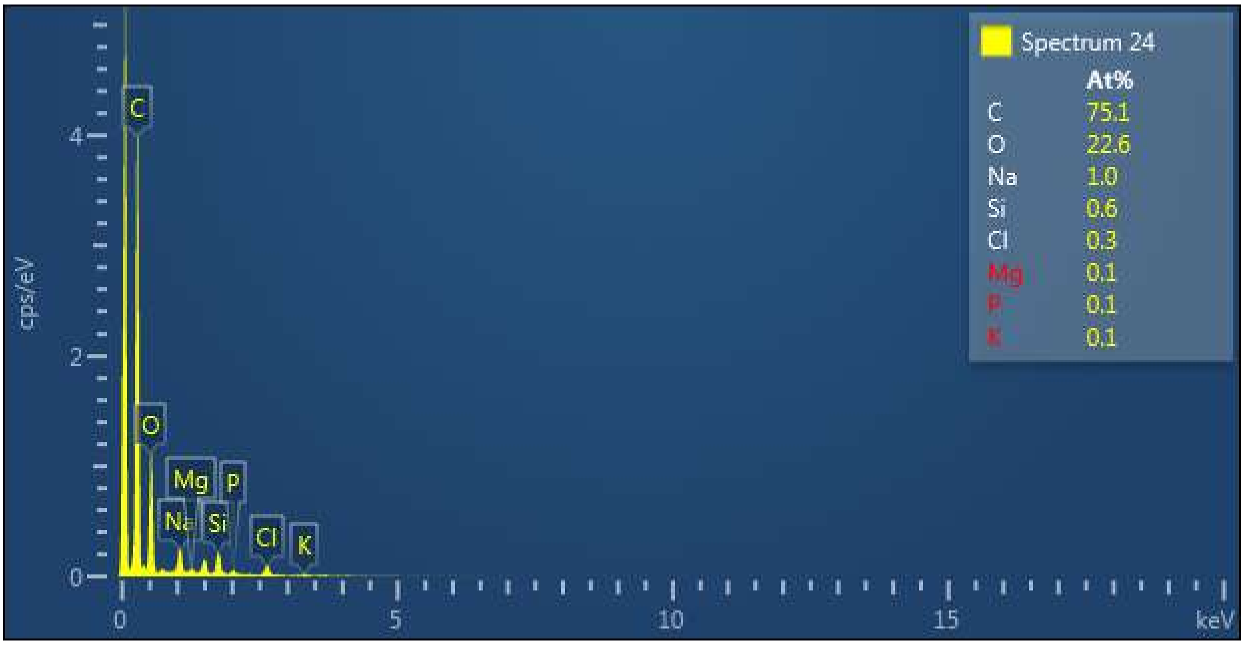

Label:	Spectrum 24
Source:	Acquired
Created:	22/01/2020 12:06:47
Livetime:	60.5s
Process Time:	4
Accelerating Voltage:	15.00kV
Magnification:	33 x
Working Distance:	10.0mm
Specimen Tilt (degrees):	0.0
Elevation (degrees):	35.0
Azimuth (degrees):	0.0
Number Of Channels:	2048
Energy Range (keV):	20 keV
Energy per Channel (eV):	10.0eV
Detector Type Id:	29
Detector Type:	X-Max
Window Type:	SATW
Pulse Pile Up Correction:	Succeeded
Primary Detector:	2617
Primary Detector Serial Number:	77871-X080

Element	Line Type	Apparent Concentration	k Ratio	Wt%	Wt% Sigma	Atomic %	Standard Label	Factory Standard	Standard Calibration Date
C	K series	7.34	0.07340	68.00	0.66	75.07	C Vit	Yes	
O	K series	3.21	0.01082	27.32	0.63	22.64	SiO2	Yes	
Na	K	0.39	0.0016	1.74	0.12	1.00	Albite	Yes	

	series		3						
Mg	K series	0.05	0.00031	0.27	0.06	0.15	MgO	Yes	
Si	K series	0.26	0.00202	1.37	0.08	0.65	SiO2	Yes	
P	K series	0.07	0.00039	0.26	0.06	0.11	GaP	Yes	
Cl	K series	0.15	0.00132	0.86	0.08	0.32	NaCl	Yes	
K	K series	0.03	0.00029	0.19	0.06	0.06	KBr	Yes	
Total:				100.00		100.00			

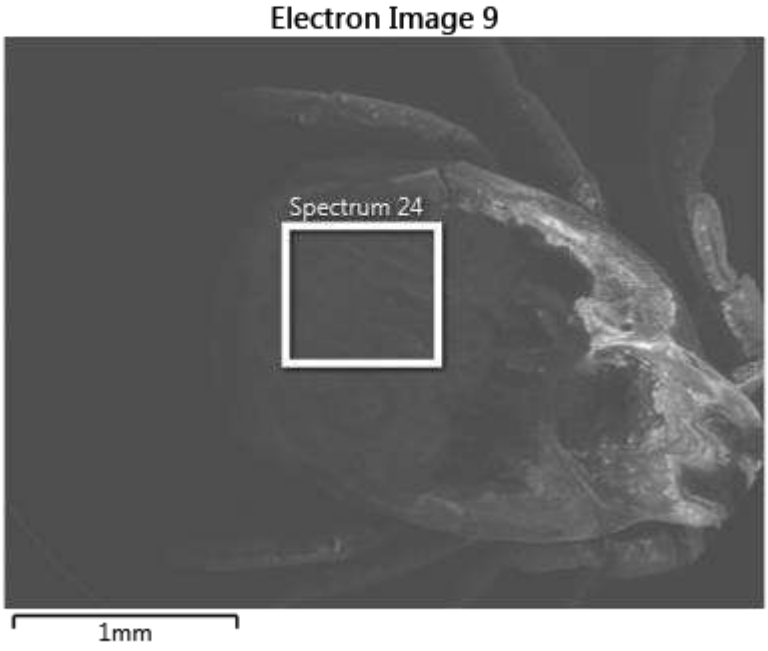

22/01/2020 12:10:01
Project 1
Rumania 8
Dorso 1 15 Kv

Electron Image 10

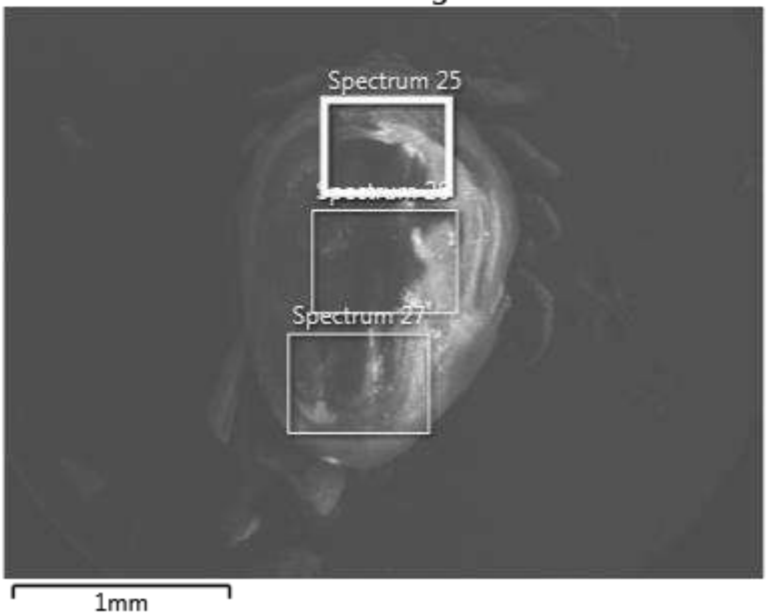

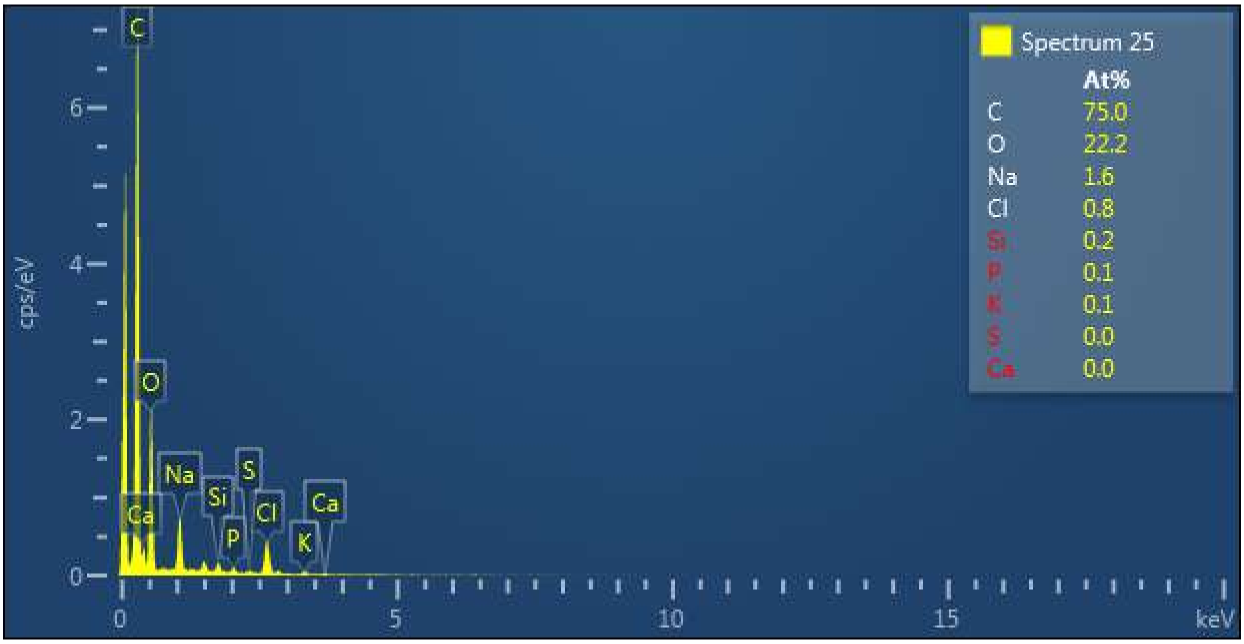

Label:	Spectrum 25
Source:	Acquired
Created:	22/01/2020 12:10:01
Livetime:	60.6s
Process Time:	4
Accelerating Voltage:	15.00kV
Magnification:	32 x
Working Distance:	10.0mm
Specimen Tilt (degrees):	0.0
Elevation (degrees):	35.0
Azimuth (degrees):	0.0
Number Of Channels:	2048
Energy Range (keV):	20 keV
Energy per Channel (eV):	10.0eV
Detector Type Id:	29
Detector Type:	X-Max
Window Type:	SATW
Pulse Pile Up Correction:	Succeeded
Primary Detector:	2617
Primary Detector Serial Number:	77871-X080

Element	Line Type	Apparent Concentration	k Ratio	Wt%	Wt% Sigma	Atomic %	Standard Label	Factory Standard	Standard Calibration Date
C	K series	13.37	0.13366	67.52	0.49	75.03	C Vit	Yes	
O	K series	6.23	0.02097	26.56	0.46	22.16	SiO2	Yes	
Na	K	1.21	0.0051	2.69	0.09	1.56	Albite	Yes	

	series		0						
Si	K series	0.14	0.00110	0.37	0.04	0.18	SiO2	Yes	
P	K series	0.13	0.00075	0.25	0.04	0.11	GaP	Yes	
S	K series	0.04	0.00036	0.11	0.03	0.05	FeS2	Yes	
Cl	K series	0.75	0.00657	2.12	0.08	0.80	NaCl	Yes	
K	K series	0.09	0.00079	0.25	0.04	0.09	KBr	Yes	
Ca	K series	0.04	0.00036	0.11	0.04	0.04	Wollastonite	Yes	
Total:				100.00		100.00			

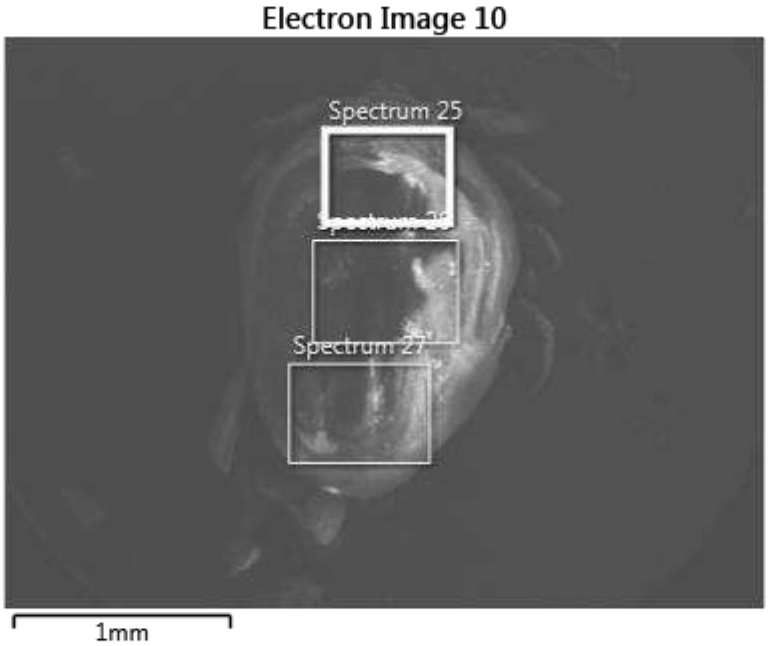

22/01/2020 12:11:11
Project 1
Rumania 8
Dorso 1 15 Kv

Electron Image 10

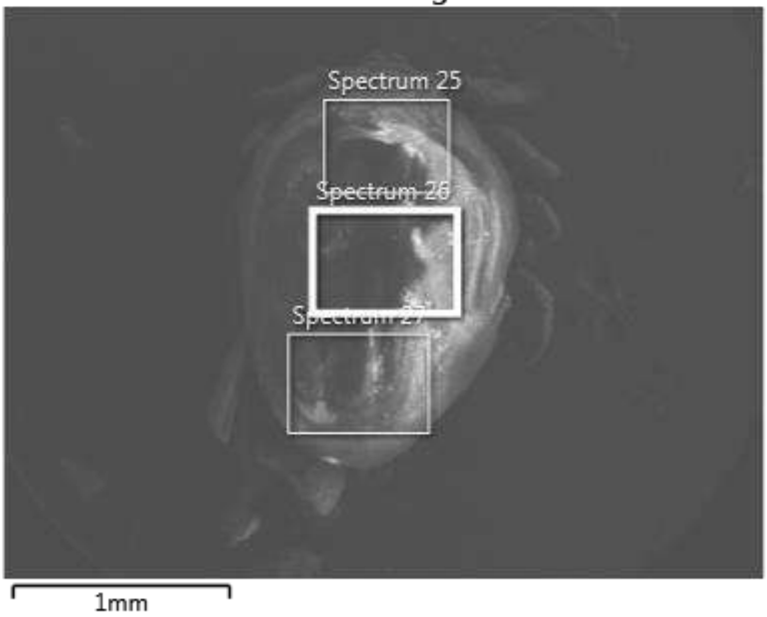

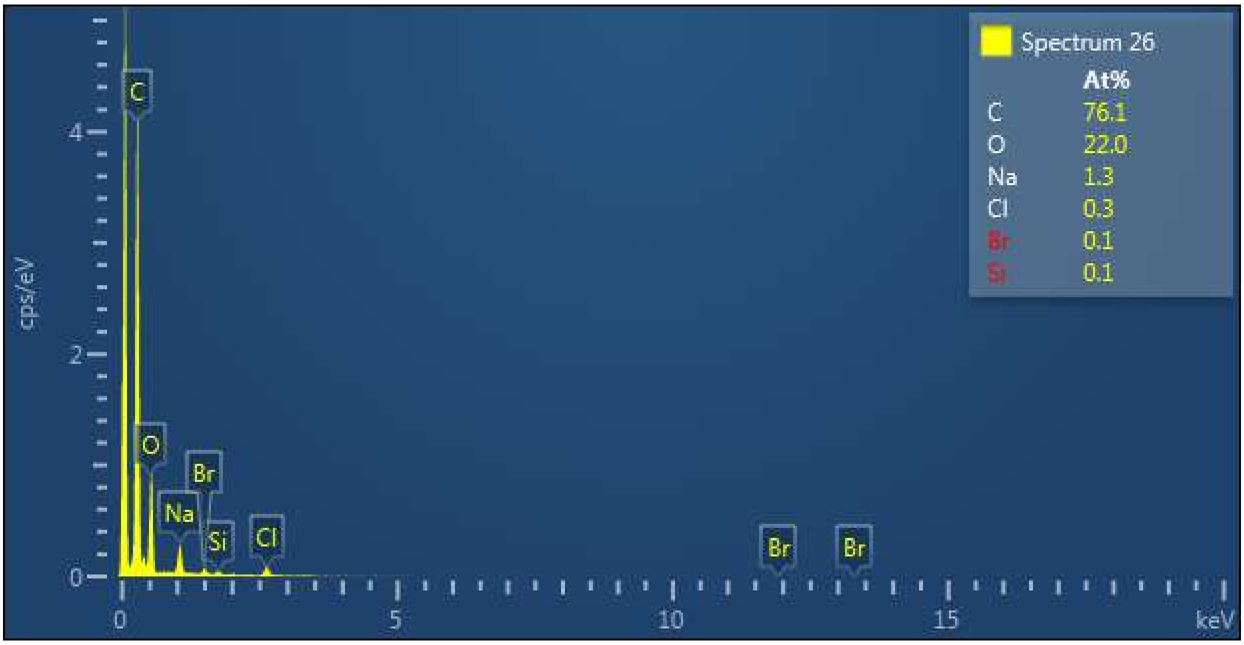

Label:	Spectrum 26
Source:	Acquired
Created:	22/01/2020 12:11:11
Livetime:	60.4s
Process Time:	4
Accelerating Voltage:	15.00kV
Magnification:	32 x
Working Distance:	10.0mm
Specimen Tilt (degrees):	0.0
Elevation (degrees):	35.0
Azimuth (degrees):	0.0
Number Of Channels:	2048
Energy Range (keV):	20 keV
Energy per Channel (eV):	10.0eV
Detector Type Id:	29
Detector Type:	X-Max
Window Type:	SATW
Pulse Pile Up Correction:	Succeeded
Primary Detector:	2617
Primary Detector Serial Number:	77871-X080

Element	Line Type	Apparent Concentration	k Ratio	Wt%	Wt% Sigma	Atomic %	Standard Label	Factory Standard	Standard Calibration Date
C	K series	6.68	0.06677	69.25	0.64	76.14	C Vit	Yes	
O	K series	2.63	0.00883	26.62	0.63	21.97	SiO2	Yes	
Na	K	0.44	0.0018	2.34	0.14	1.34	Albite	Yes	

	series		6						
Si	K series	0.03	0.00026	0.21	0.05	0.10	SiO2	Yes	
Cl	K series	0.13	0.00113	0.87	0.08	0.32	NaCl	Yes	
Br	L series	0.09	0.00078	0.71	0.13	0.12	KBr	Yes	
Total:				100.00		100.00			

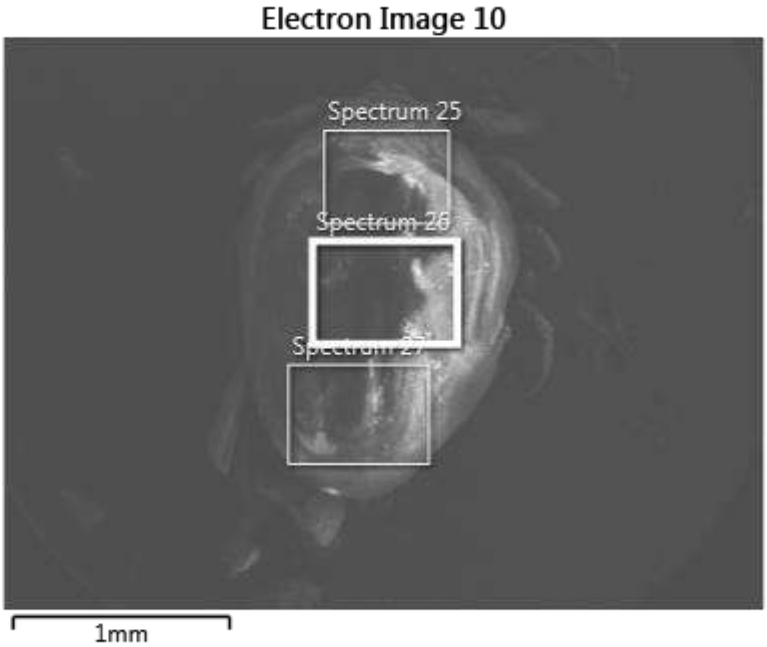

22/01/2020 12:12:21
Project 1
Rumania 8
Dorso 1 15 Kv

Electron Image 10

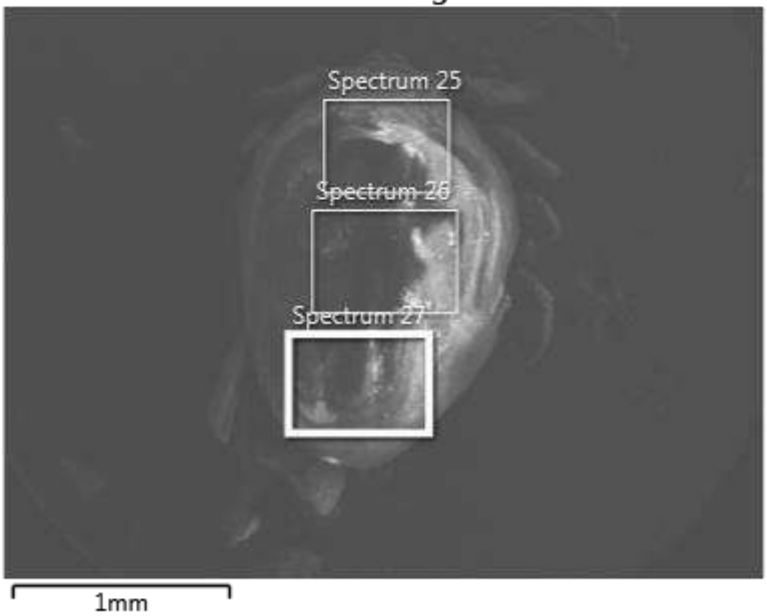

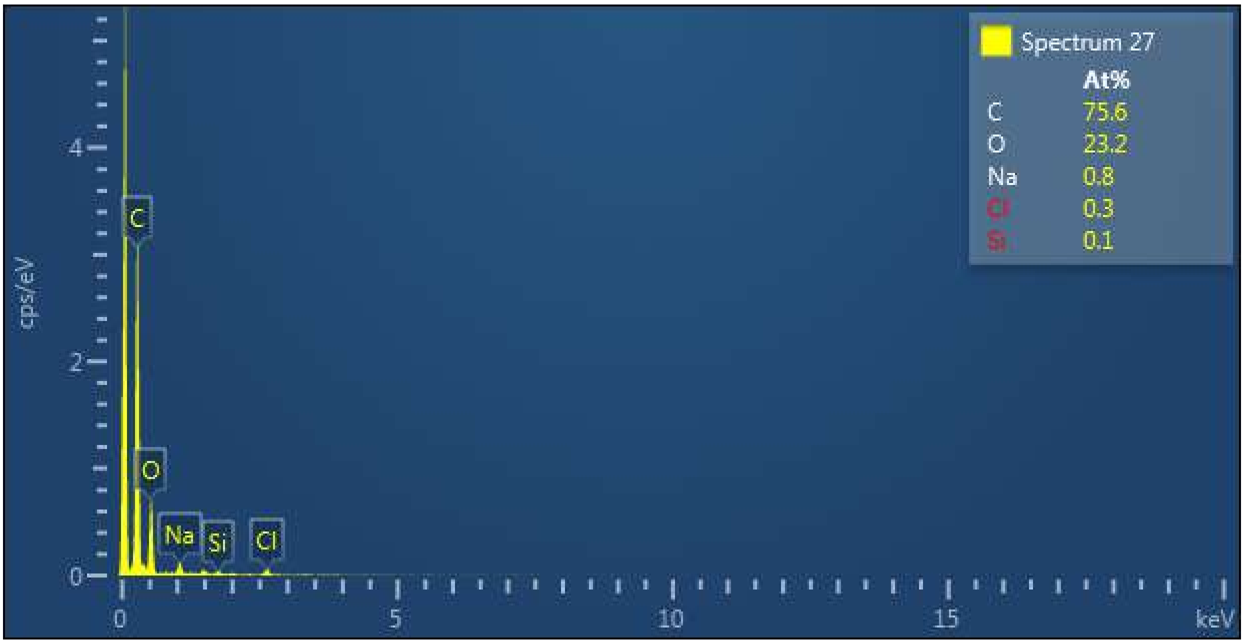

Label:	Spectrum 27
Source:	Acquired
Created:	22/01/2020 12:12:21
Livetime:	60.1s
Process Time:	4
Accelerating Voltage:	15.00kV
Magnification:	32 x
Working Distance:	10.0mm
Specimen Tilt (degrees):	0.0
Elevation (degrees):	35.0
Azimuth (degrees):	0.0
Number Of Channels:	2048
Energy Range (keV):	20 keV
Energy per Channel (eV):	10.0eV
Detector Type Id:	29
Detector Type:	X-Max
Window Type:	SATW
Pulse Pile Up Correction:	Succeeded
Primary Detector:	2617
Primary Detector Serial Number:	77871-X080

Element	Line Type	Apparent Concentration	k Ratio	Wt%	Wt% Sigma	Atomic %	Standard Label	Factory Standard	Standard Calibration Date
C	K series	5.12	0.05117	69.31	0.74	75.65	C Vit	Yes	
O	K series	2.02	0.00681	28.26	0.74	23.15	SiO2	Yes	
Na	K	0.18	0.0007	1.32	0.13	0.75	Albite	Yes	

	series		4						
Si	K series	0.03	0.00028	0.30	0.07	0.14	SiO2	Yes	
Cl	K series	0.09	0.00077	0.82	0.10	0.30	NaCl	Yes	
Total:				100.00		100.00			

Electron Image 10

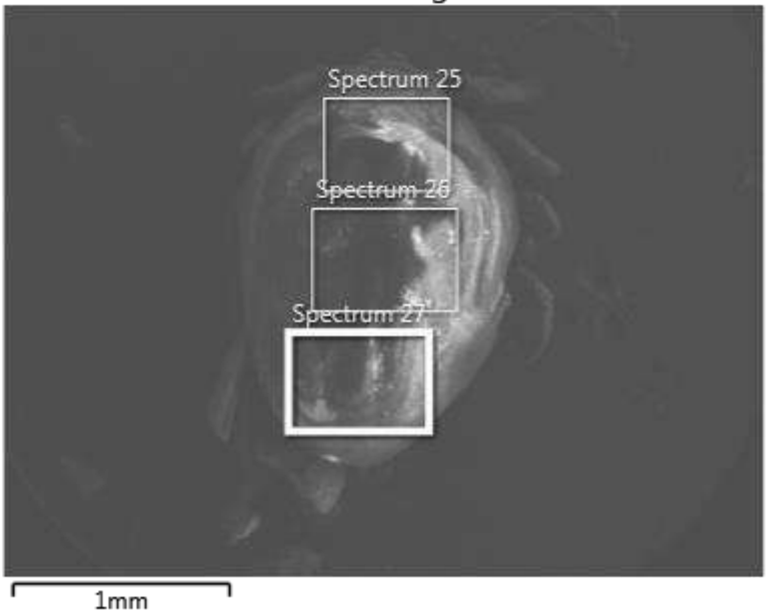

22/01/2020 12:21:52
Project 1
Rumania 9
Dorso 1 15 Kv

Electron Image 11

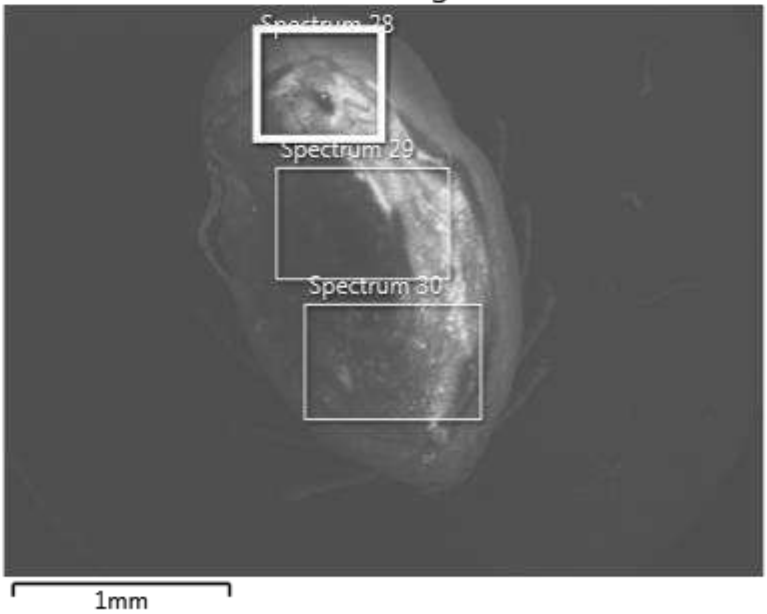

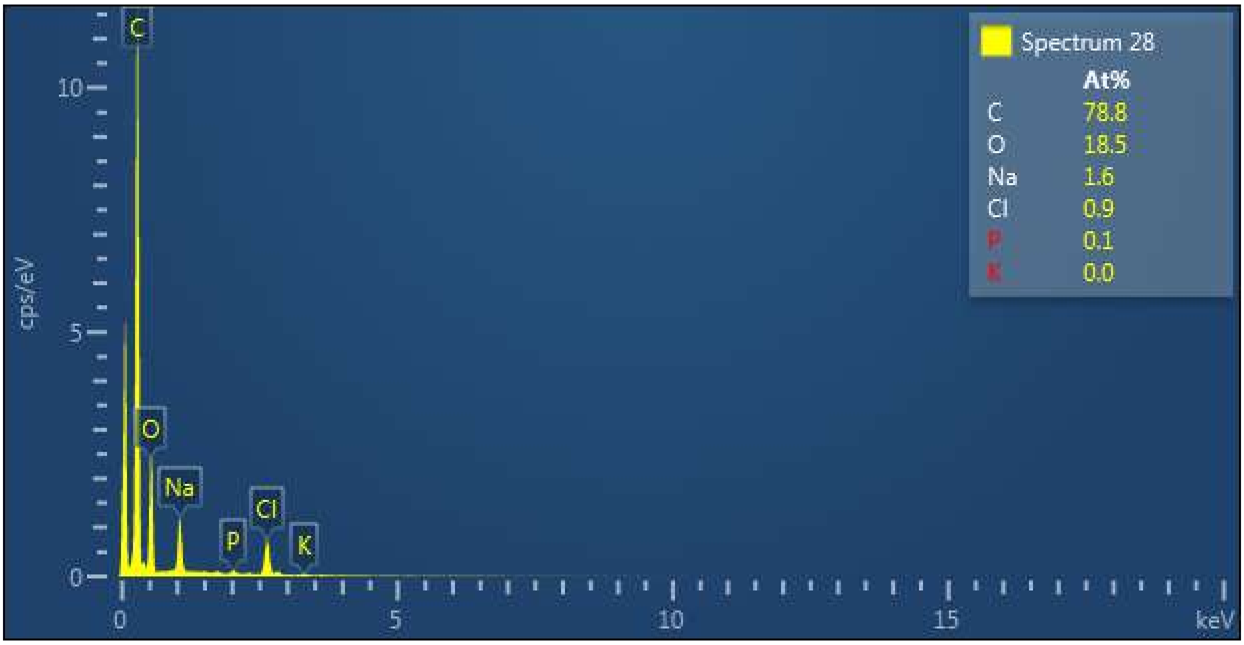

Label:	Spectrum 28
Source:	Acquired
Created:	22/01/2020 12:21:52
Livetime:	60.5s
Process Time:	4
Accelerating Voltage:	15.00kV
Magnification:	32 x
Working Distance:	10.0mm
Specimen Tilt (degrees):	0.0
Elevation (degrees):	35.0
Azimuth (degrees):	0.0
Number Of Channels:	2048
Energy Range (keV):	20 keV
Energy per Channel (eV):	10.0eV
Detector Type Id:	29
Detector Type:	X-Max
Window Type:	SATW
Pulse Pile Up Correction:	Succeeded
Primary Detector:	2617
Primary Detector Serial Number:	77871-X080

Element	Line Type	Apparent Concentration	k Ratio	Wt%	Wt% Sigma	Atomic %	Standard Label	Factory Standard	Standard Calibration Date
C	K series	20.86	0.20861	71.85	0.38	78.80	C Vit	Yes	
O	K series	7.14	0.02403	22.49	0.36	18.51	SiO2	Yes	
Na	K	1.87	0.0078	2.85	0.08	1.63	Albite	Yes	

	series		7						
P	K series	0.16	0.00088	0.20	0.03	0.09	GaP	Yes	
Cl	K series	1.27	0.01112	2.50	0.07	0.93	NaCl	Yes	
K	K series	0.06	0.00051	0.11	0.03	0.04	KBr	Yes	
Total:				100.00		100.00			

Electron Image 11

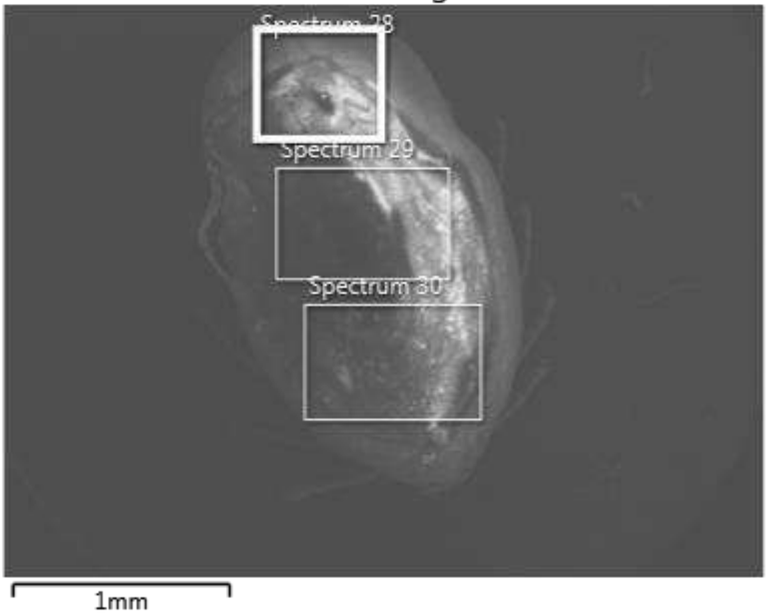

22/01/2020 12:23:03
Project 1
Rumania 9
Dorso 1 15 Kv

Electron Image 11

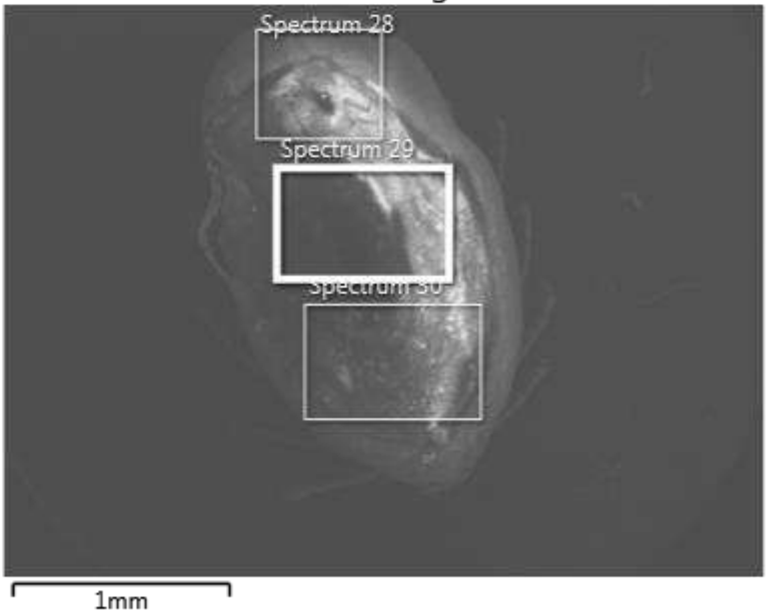

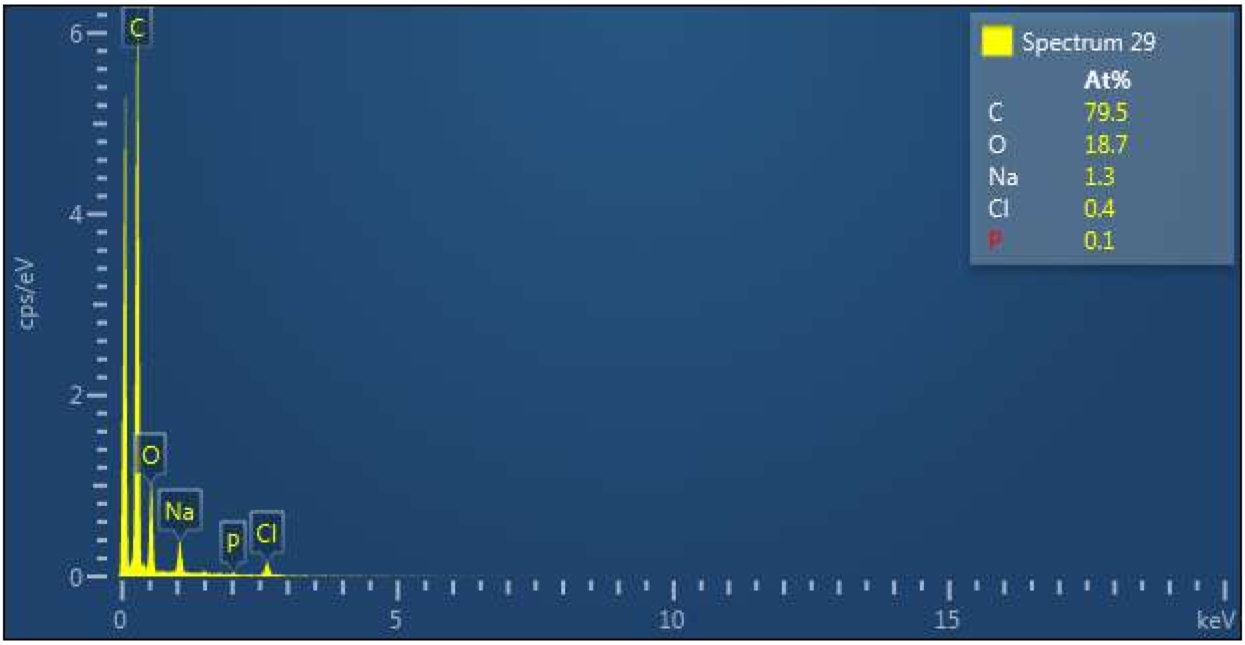

Label:	Spectrum 29
Source:	Acquired
Created:	22/01/2020 12:23:03
Livetime:	60.5s
Process Time:	4
Accelerating Voltage:	15.00kV
Magnification:	32 x
Working Distance:	10.0mm
Specimen Tilt (degrees):	0.0
Elevation (degrees):	35.0
Azimuth (degrees):	0.0
Number Of Channels:	2048
Energy Range (keV):	20 keV
Energy per Channel (eV):	10.0eV
Detector Type Id:	29
Detector Type:	X-Max
Window Type:	SATW
Pulse Pile Up Correction:	Succeeded
Primary Detector:	2617
Primary Detector Serial Number:	77871-X080

Element	Line Type	Apparent Concentration	k Ratio	Wt%	Wt% Sigma	Atomic %	Standard Label	Factory Standard	Standard Calibration Date
C	K series	10.01	0.10015	73.42	0.53	79.52	C Vit	Yes	
O	K series	2.90	0.00977	23.02	0.53	18.72	SiO2	Yes	
Na	K	0.58	0.0024	2.26	0.11	1.28	Albite	Yes	

	series		6						
P	K series	0.04	0.00023	0.14	0.04	0.06	GaP	Yes	
Cl	K series	0.23	0.00204	1.16	0.08	0.42	NaCl	Yes	
Total:				100.00		100.00			

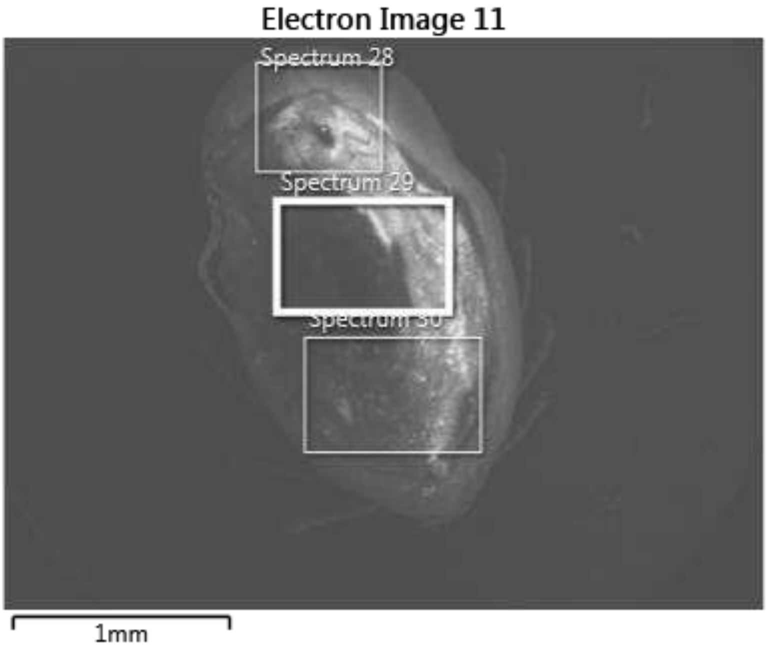

22/01/2020 12:24:12
Project 1
Rumania 9
Dorso 1 15 Kv

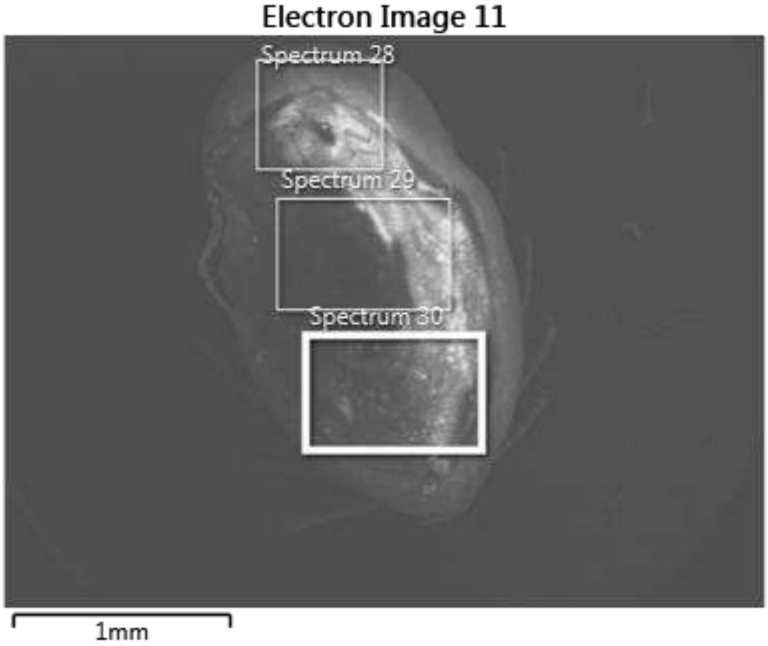

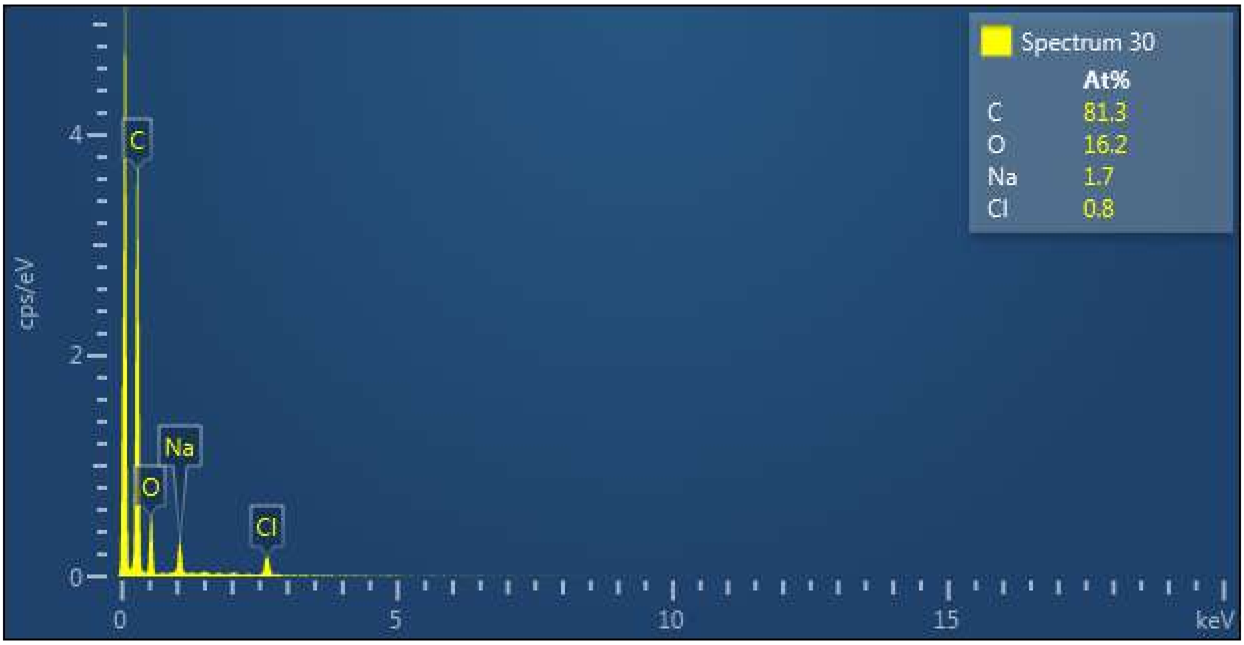

Label:	Spectrum 30
Source:	Acquired
Created:	22/01/2020 12:24:12
Livetime:	60.1s
Process Time:	4
Accelerating Voltage:	15.00kV
Magnification:	32 x
Working Distance:	10.0mm
Specimen Tilt (degrees):	0.0
Elevation (degrees):	35.0
Azimuth (degrees):	0.0
Number Of Channels:	2048
Energy Range (keV):	20 keV
Energy per Channel (eV):	10.0eV
Detector Type Id:	29
Detector Type:	X-Max
Window Type:	SATW
Pulse Pile Up Correction:	Succeeded
Primary Detector:	2617
Primary Detector Serial Number:	77871-X080

Element	Line Type	Apparent Concentration	k Ratio	Wt%	Wt% Sigma	Atomic %	Standard Label	Factory Standard	Standard Calibration Date
C	K series	6.19	0.06193	74.94	0.61	81.33	C Vit	Yes	
O	K series	1.63	0.00549	19.83	0.60	16.15	SiO2	Yes	
Na	K	0.53	0.0022	2.99	0.15	1.69	Albite	Yes	

	series		5						
Cl	K series	0.31	0.00269	2.25	0.13	0.83	NaCl	Yes	
Total:				100.00		100.00			

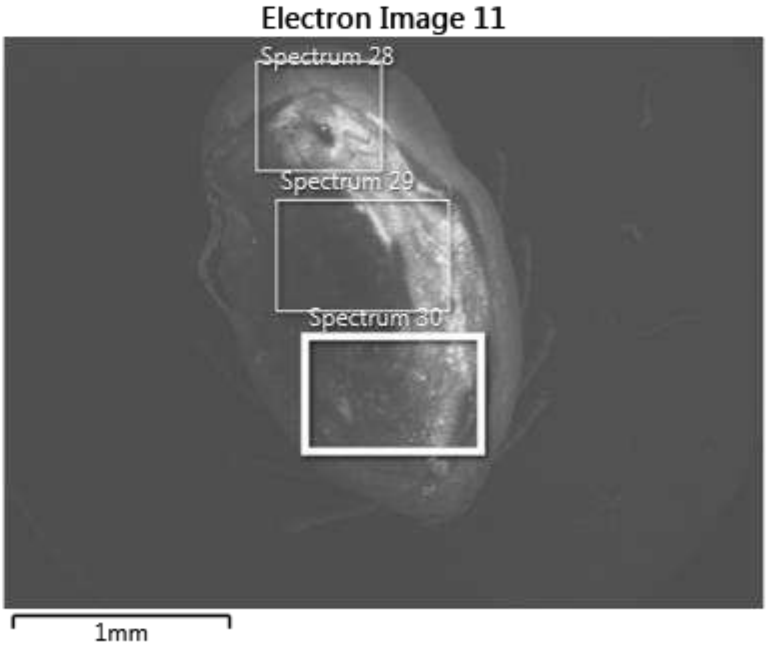

23/01/2020 09:52:09
Project 1
Rumania 1
Pecho 1 15 Kv

Electron Image 13

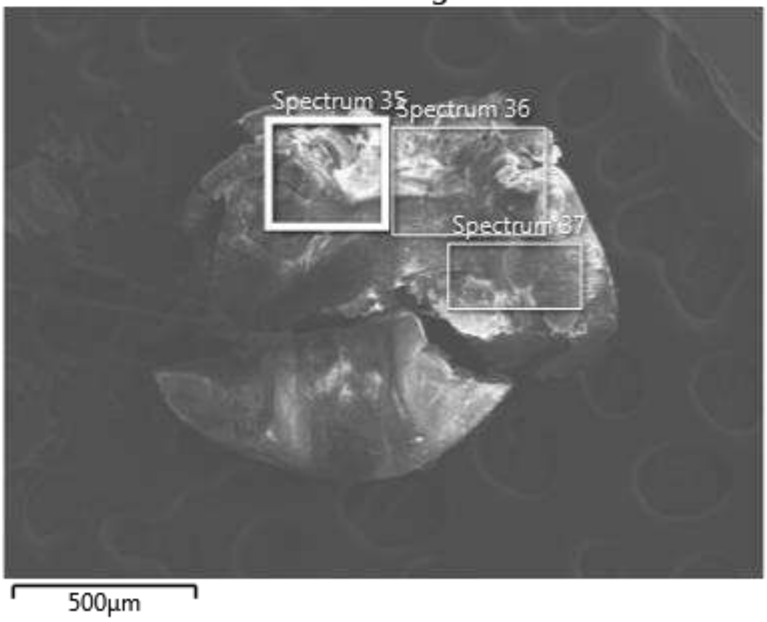

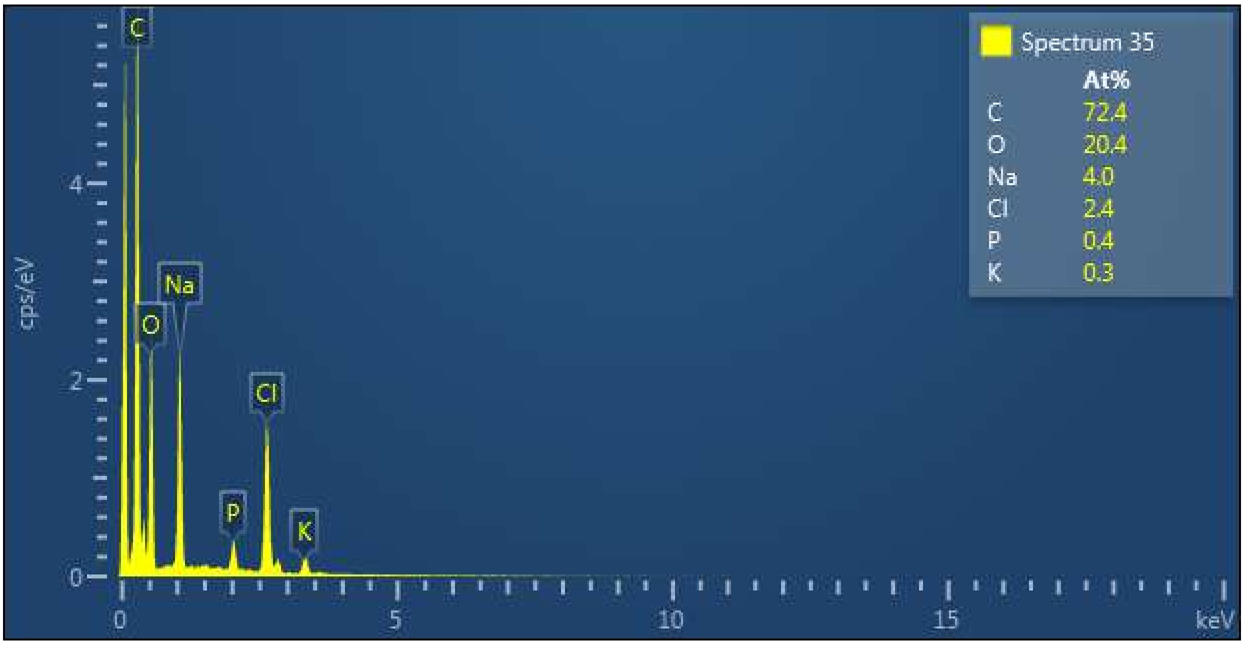

Label:	Spectrum 35
Source:	Acquired
Created:	23/01/2020 09:52:09
Livetime:	60.1s
Process Time:	4
Accelerating Voltage:	15.00kV
Magnification:	54 x
Working Distance:	10.0mm
Specimen Tilt (degrees):	0.0
Elevation (degrees):	35.0
Azimuth (degrees):	0.0
Number Of Channels:	2048
Energy Range (keV):	20 keV
Energy per Channel (eV):	10.0eV
Detector Type Id:	29
Detector Type:	X-Max
Window Type:	SATW
Pulse Pile Up Correction:	Succeeded
Primary Detector:	2617
Primary Detector Serial Number:	77871-X080

Element	Line Type	Apparent Concentration	k Ratio	Wt%	Wt% Sigma	Atomic %	Standard Label	Factory Standard	Standard Calibration Date
C	K series	9.70	0.09695	62.14	0.53	72.42	C Vit	Yes	
O	K series	6.72	0.02262	23.35	0.42	20.43	SiO2	Yes	
Na	K	3.73	0.0157	6.53	0.13	3.98	Albite	Yes	

	series		5						
P	K series	0.63	0.00354	0.95	0.05	0.43	GaP	Yes	
Cl	K series	2.66	0.02325	6.09	0.13	2.41	NaCl	Yes	
K	K series	0.42	0.00355	0.93	0.06	0.33	KBr	Yes	
Total:				100.00		100.00			

Electron Image 13

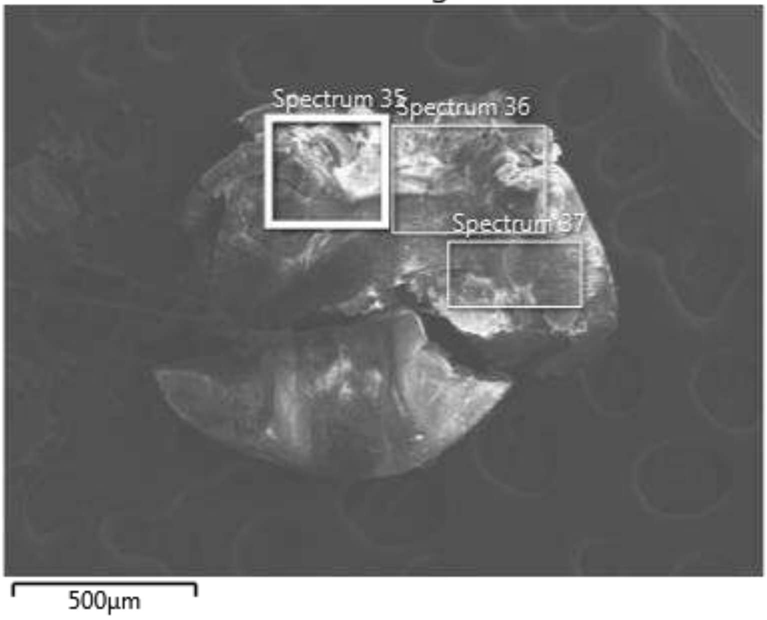

23/01/2020 09:53:19
Project 1
Rumania 1
Pecho 1 15 Kv

Electron Image 13

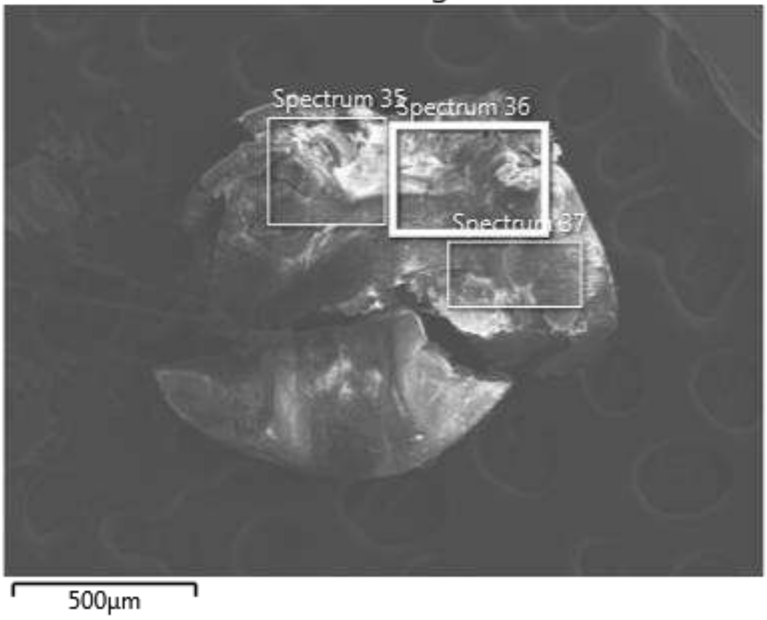

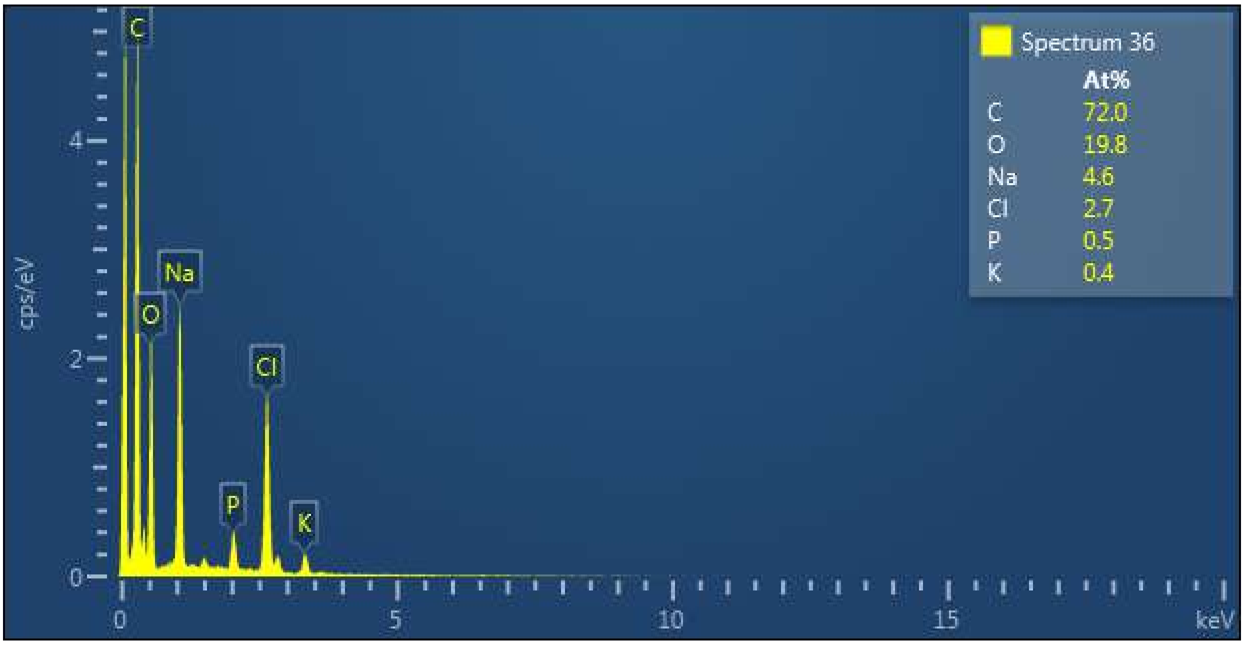

Label:	Spectrum 36
Source:	Acquired
Created:	23/01/2020 09:53:19
Livetime:	60.2s
Process Time:	4
Accelerating Voltage:	15.00kV
Magnification:	54 x
Working Distance:	10.0mm
Specimen Tilt (degrees):	0.0
Elevation (degrees):	35.0
Azimuth (degrees):	0.0
Number Of Channels:	2048
Energy Range (keV):	20 keV
Energy per Channel (eV):	10.0eV
Detector Type Id:	29
Detector Type:	X-Max
Window Type:	SATW
Pulse Pile Up Correction:	Succeeded
Primary Detector:	2617
Primary Detector Serial Number:	77871-X080

Element	Line Type	Apparent Concentration	k Ratio	Wt%	Wt% Sigma	Atomic %	Standard Label	Factory Standard	Standard Calibration Date
C	K series	8.60	0.08600	61.14	0.55	71.97	C Vit	Yes	
O	K series	6.25	0.02103	22.42	0.42	19.81	SiO2	Yes	
Na	K	4.22	0.0178	7.55	0.15	4.64	Albite	Yes	

	series		0						
P	K series	0.76	0.00423	1.17	0.06	0.53	GaP	Yes	
Cl	K series	2.83	0.02469	6.69	0.14	2.67	NaCl	Yes	
K	K series	0.45	0.00382	1.04	0.06	0.38	KBr	Yes	
Total:				100.00		100.00			

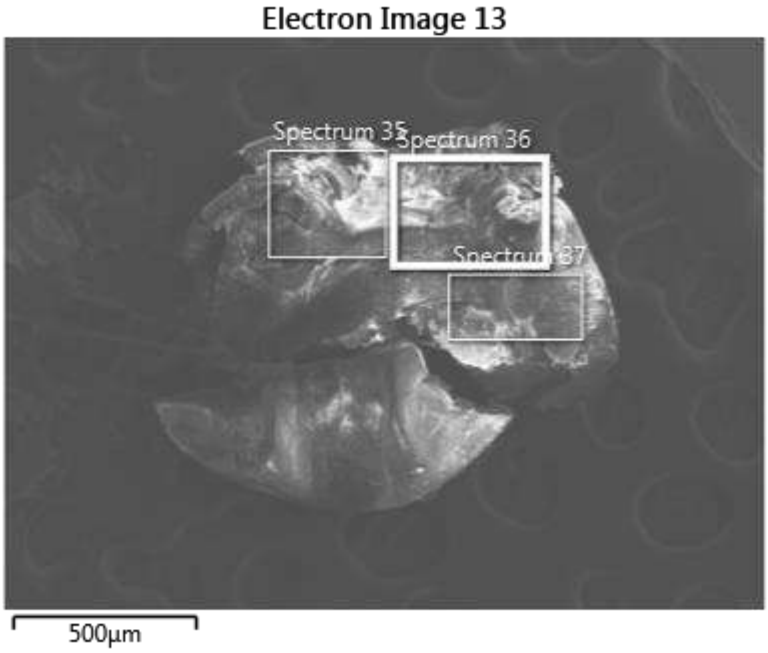

23/01/2020 09:54:28
Project 1
Rumania 1
Pecho 1 15 Kv

Electron Image 13

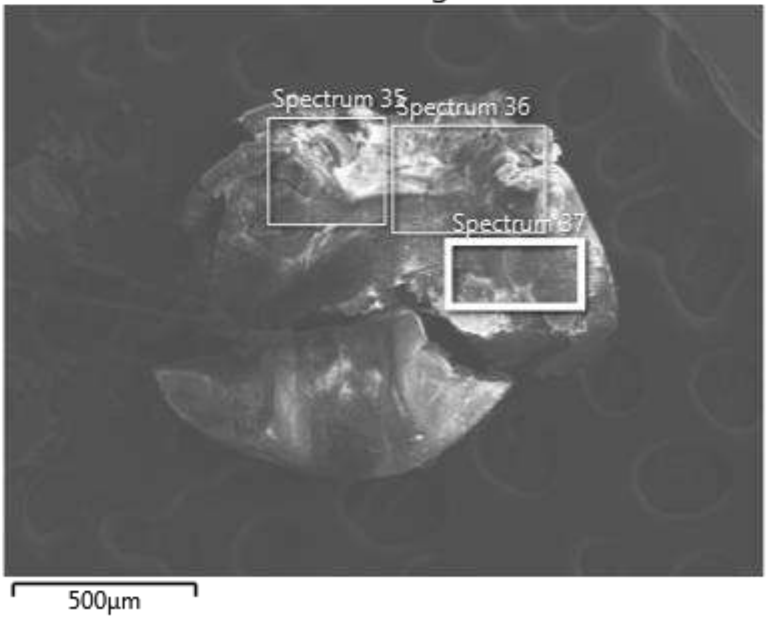

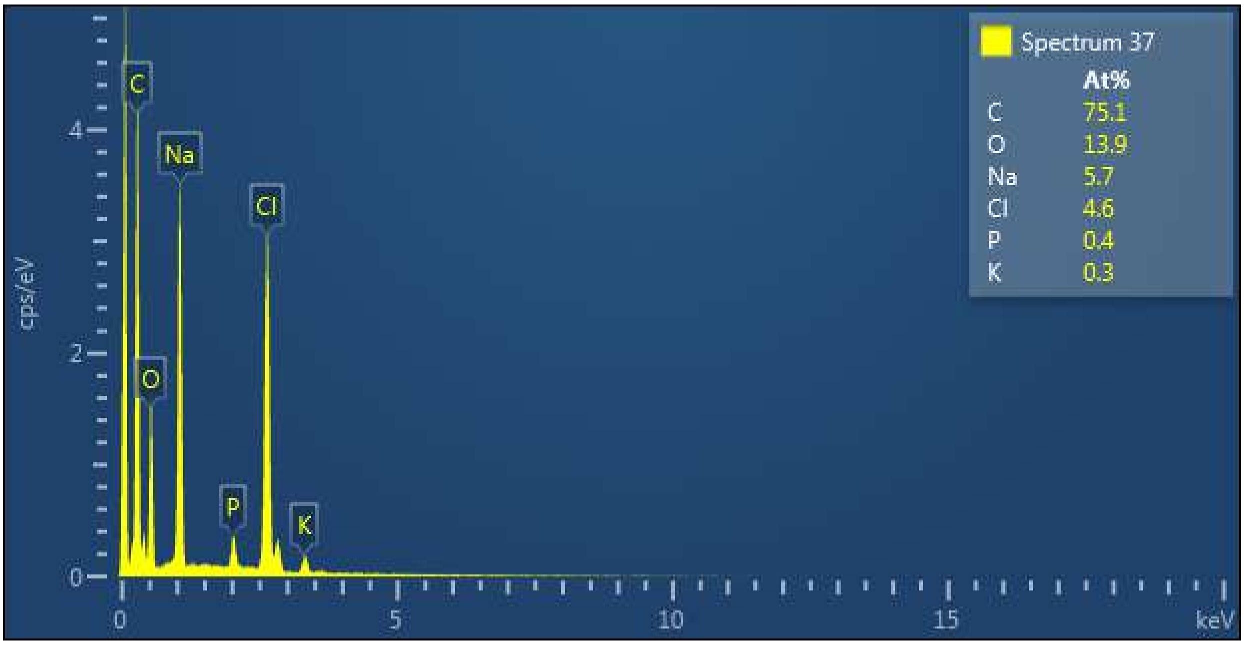

Label:	Spectrum 37
Source:	Acquired
Created:	23/01/2020 09:54:28
Livetime:	60.4s
Process Time:	4
Accelerating Voltage:	15.00kV
Magnification:	54 x
Working Distance:	10.0mm
Specimen Tilt (degrees):	0.0
Elevation (degrees):	35.0
Azimuth (degrees):	0.0
Number Of Channels:	2048
Energy Range (keV):	20 keV
Energy per Channel (eV):	10.0eV
Detector Type Id:	29
Detector Type:	X-Max
Window Type:	SATW
Pulse Pile Up Correction:	Succeeded
Primary Detector:	2617
Primary Detector Serial Number:	77871-X080

Element	Line Type	Apparent Concentration	k Ratio	Wt%	Wt% Sigma	Atomic %	Standard Label	Factory Standard	Standard Calibration Date
C	K series	7.34	0.07337	62.53	0.56	75.10	C Vit	Yes	
O	K series	4.44	0.01493	15.43	0.36	13.91	SiO2	Yes	
Na	K	5.91	0.0249	9.07	0.17	5.69	Albite	Yes	

	series		5						
P	K series	0.60	0.00333	0.82	0.05	0.38	GaP	Yes	
Cl	K series	5.39	0.04711	11.35	0.20	4.62	NaCl	Yes	
K	K series	0.38	0.00325	0.80	0.05	0.30	KBr	Yes	
Total:				100.00		100.00			

Electron Image 13

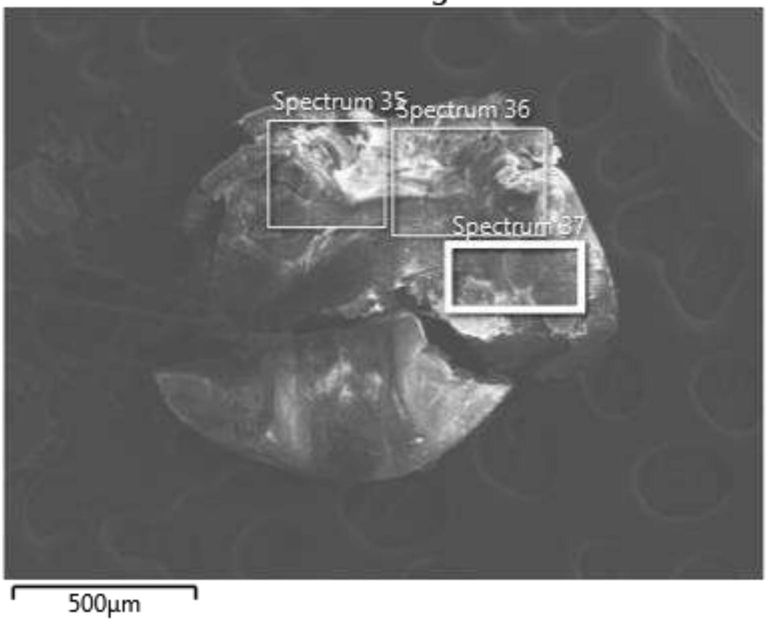

23/01/2020 09:56:31
Project 1
Rumania 1
Ventral 1 15 Kv

Electron Image 14

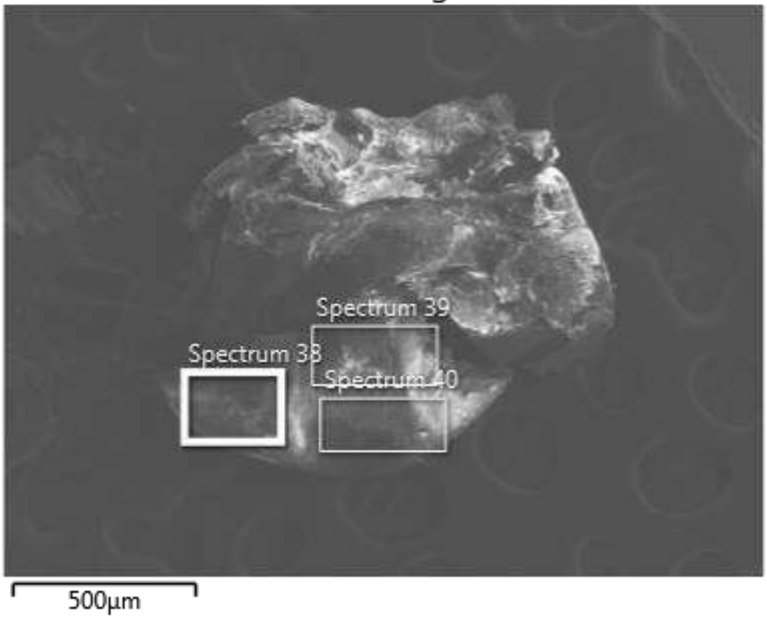

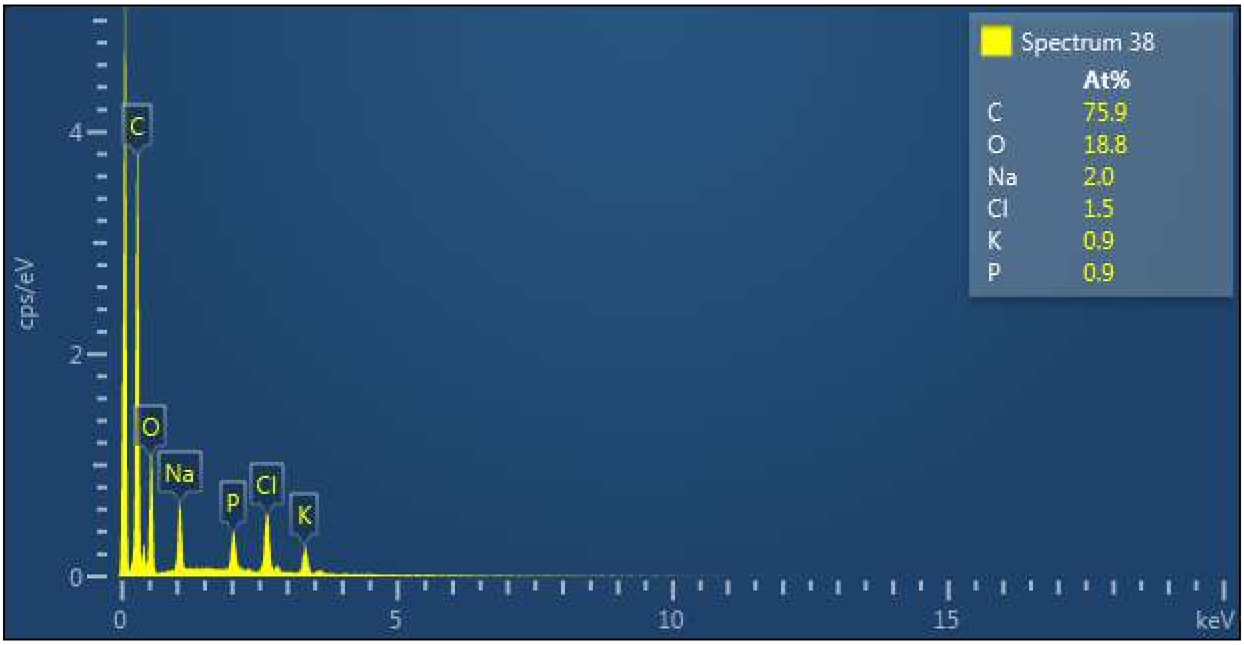

Label:	Spectrum 38
Source:	Acquired
Created:	23/01/2020 09:56:31
Livetime:	60.4s
Process Time:	4
Accelerating Voltage:	15.00kV
Magnification:	54 x
Working Distance:	10.0mm
Specimen Tilt (degrees):	0.0
Elevation (degrees):	35.0
Azimuth (degrees):	0.0
Number Of Channels:	2048
Energy Range (keV):	20 keV
Energy per Channel (eV):	10.0eV
Detector Type Id:	29
Detector Type:	X-Max
Window Type:	SATW
Pulse Pile Up Correction:	Succeeded
Primary Detector:	2617
Primary Detector Serial Number:	77871-X080

Element	Line Type	Apparent Concentration	k Ratio	Wt%	Wt% Sigma	Atomic %	Standard Label	Factory Standard	Standard Calibration Date
C	K series	6.93	0.06928	66.34	0.62	75.89	C Vit	Yes	
O	K series	3.27	0.01100	21.92	0.54	18.83	SiO2	Yes	
Na	K	1.05	0.0044	3.37	0.13	2.02	Albite	Yes	

	serie s		3						
P	K serie s	0.73	0.0040 7	1.97	0.10	0.87	GaP	Yes	
Cl	K serie s	0.94	0.0082 0	3.90	0.14	1.51	NaCl	Yes	
K	K serie s	0.62	0.0052 6	2.50	0.12	0.88	KBr	Yes	
Total:				100.0 0		100.0 0			

Electron Image 14

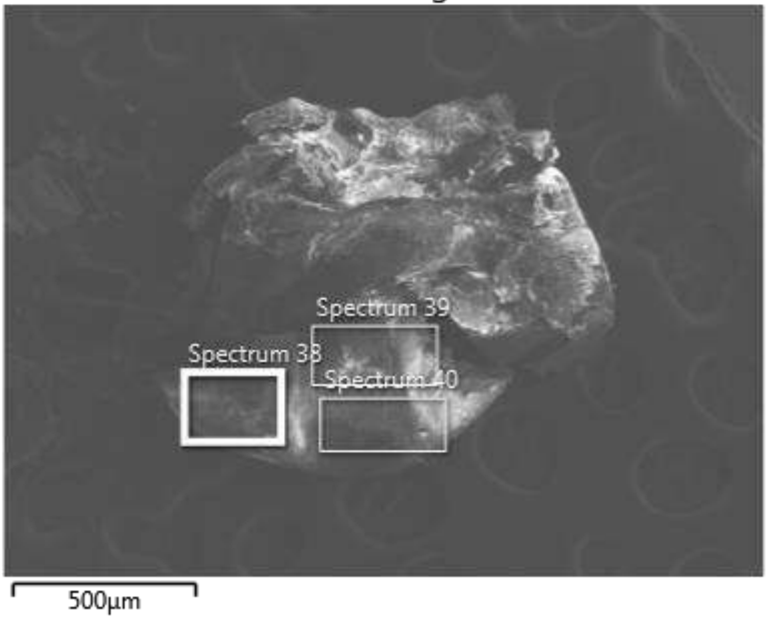

23/01/2020 09:57:43
Project 1
Rumania 1
Ventral 1 15 Kv

Electron Image 14

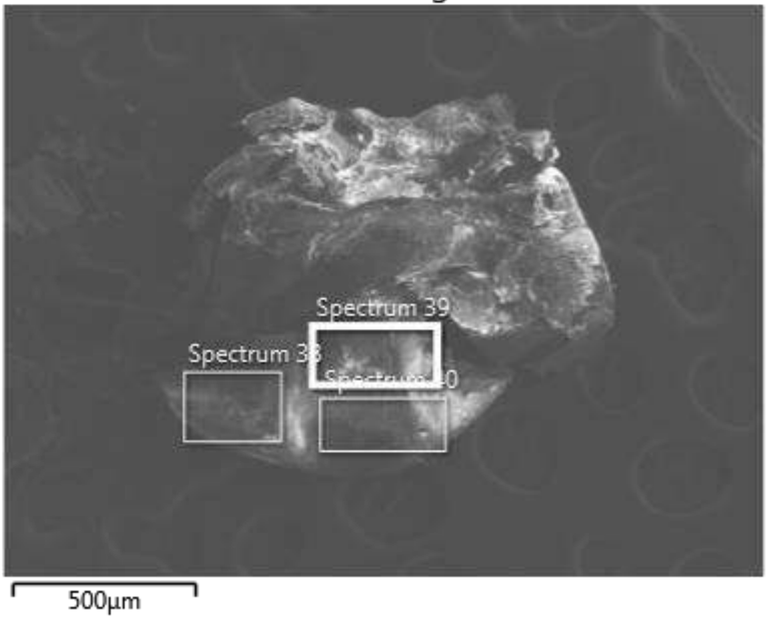

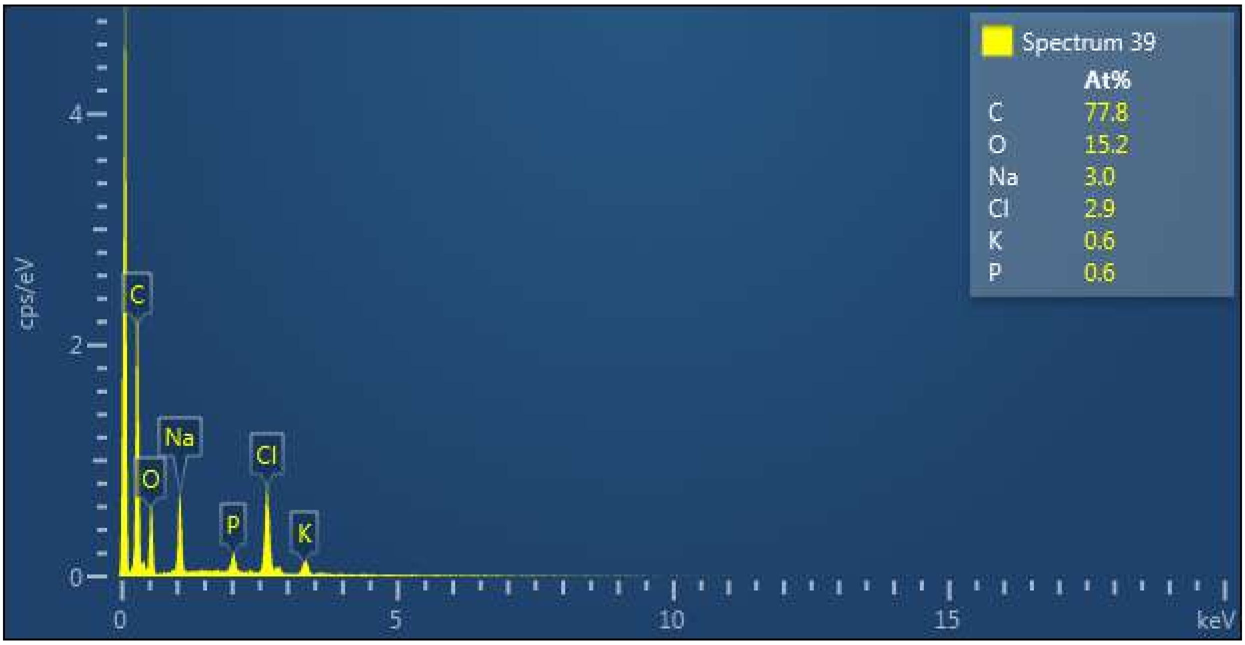

Label:	Spectrum 39
Source:	Acquired
Created:	23/01/2020 09:57:43
Livetime:	60.3s
Process Time:	4
Accelerating Voltage:	15.00kV
Magnification:	54 x
Working Distance:	10.0mm
Specimen Tilt (degrees):	0.0
Elevation (degrees):	35.0
Azimuth (degrees):	0.0
Number Of Channels:	2048
Energy Range (keV):	20 keV
Energy per Channel (eV):	10.0eV
Detector Type Id:	29
Detector Type:	X-Max
Window Type:	SATW
Pulse Pile Up Correction:	Succeeded
Primary Detector:	2617
Primary Detector Serial Number:	77871-X080

Element	Line Type	Apparent Concentration	k Ratio	Wt%	Wt% Sigma	Atomic %	Standard Label	Factory Standard	Standard Calibration Date
C	K series	3.95	0.03951	67.25	0.79	77.76	C Vit	Yes	
O	K series	1.81	0.00608	17.51	0.61	15.20	SiO2	Yes	
Na	K	1.16	0.0049	5.02	0.18	3.03	Albite	Yes	

	series		0						
P	K series	0.33	0.00186	1.24	0.10	0.56	GaP	Yes	
Cl	K series	1.27	0.01108	7.29	0.23	2.86	NaCl	Yes	
K	K series	0.30	0.00252	1.68	0.13	0.60	KBr	Yes	
Total:				100.00		100.00			

Electron Image 14

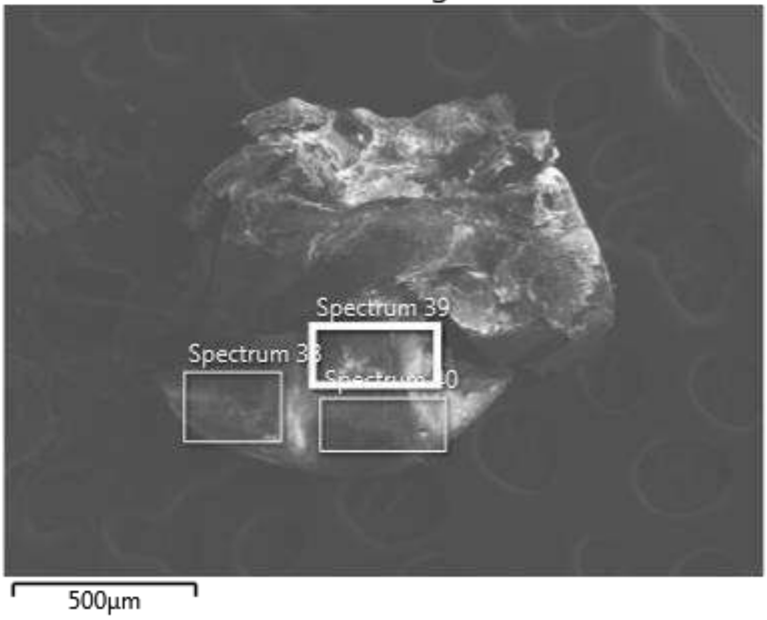

23/01/2020 09:58:53
Project 1
Rumania 1
Ventral 1 15 Kv

Electron Image 14

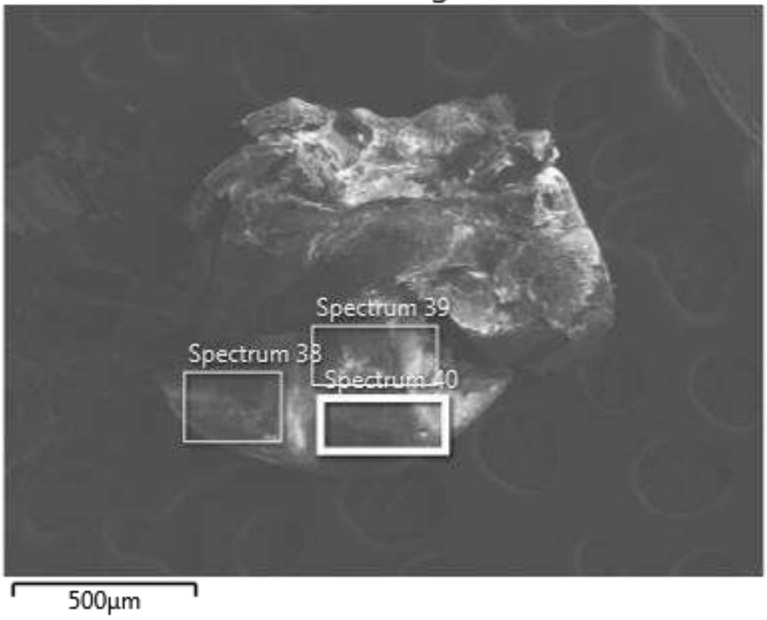

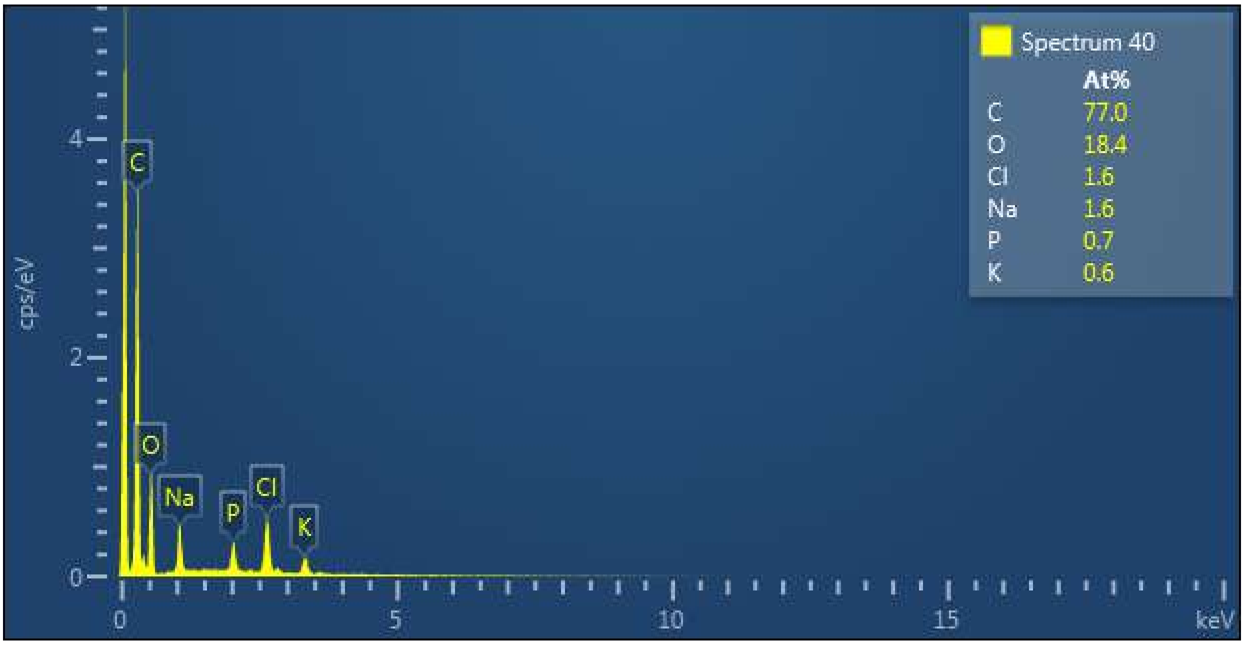

Label:	Spectrum 40
Source:	Acquired
Created:	23/01/2020 09:58:53
Livetime:	60.2s
Process Time:	4
Accelerating Voltage:	15.00kV
Magnification:	54 x
Working Distance:	10.0mm
Specimen Tilt (degrees):	0.0
Elevation (degrees):	35.0
Azimuth (degrees):	0.0
Number Of Channels:	2048
Energy Range (keV):	20 keV
Energy per Channel (eV):	10.0eV
Detector Type Id:	29
Detector Type:	X-Max
Window Type:	SATW
Pulse Pile Up Correction:	Succeeded
Primary Detector:	2617
Primary Detector Serial Number:	77871-X080

Element	Line Type	Apparent Concentration	k Ratio	Wt%	Wt% Sigma	Atomic %	Standard Label	Factory Standard	Standard Calibration Date
C	K series	6.31	0.06310	68.04	0.65	77.03	C Vit	Yes	
O	K series	2.81	0.00945	21.70	0.57	18.45	SiO2	Yes	
Na	K	0.73	0.0030	2.68	0.12	1.59	Albite	Yes	

	series		8						
P	K series	0.55	0.00305	1.68	0.10	0.74	GaP	Yes	
Cl	K series	0.87	0.00762	4.14	0.15	1.59	NaCl	Yes	
K	K series	0.38	0.00324	1.76	0.11	0.61	KBr	Yes	
Total:				100.00		100.00			

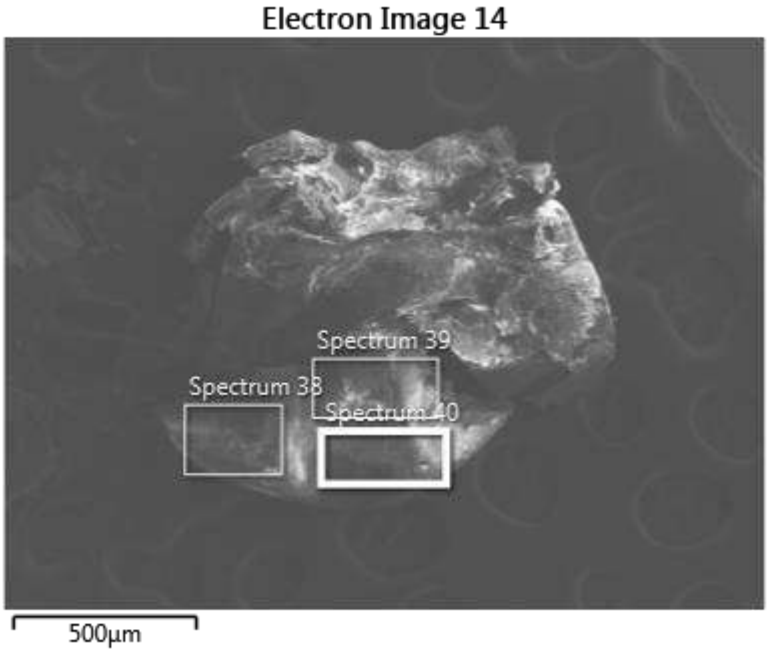

23/01/2020 10:11:43
Project 1
Rumania 10
Dorso 1 15 Kv

Electron Image 15

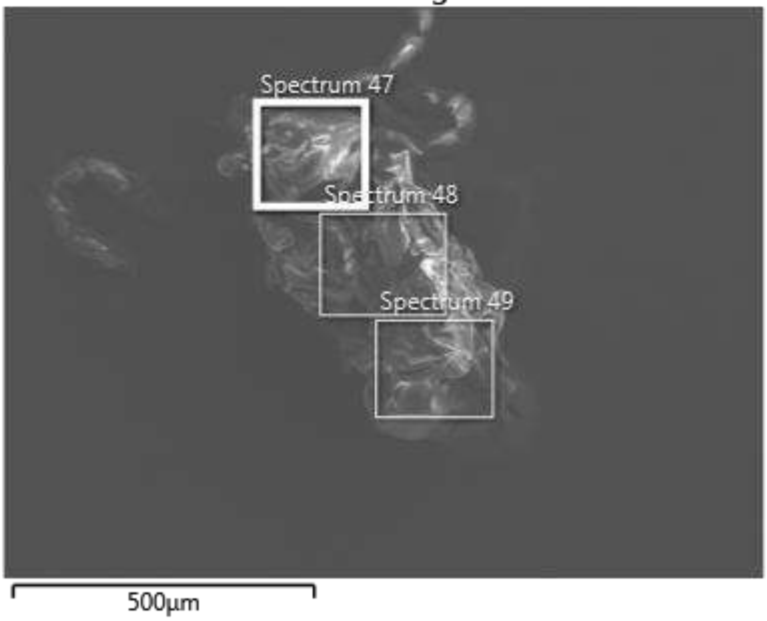

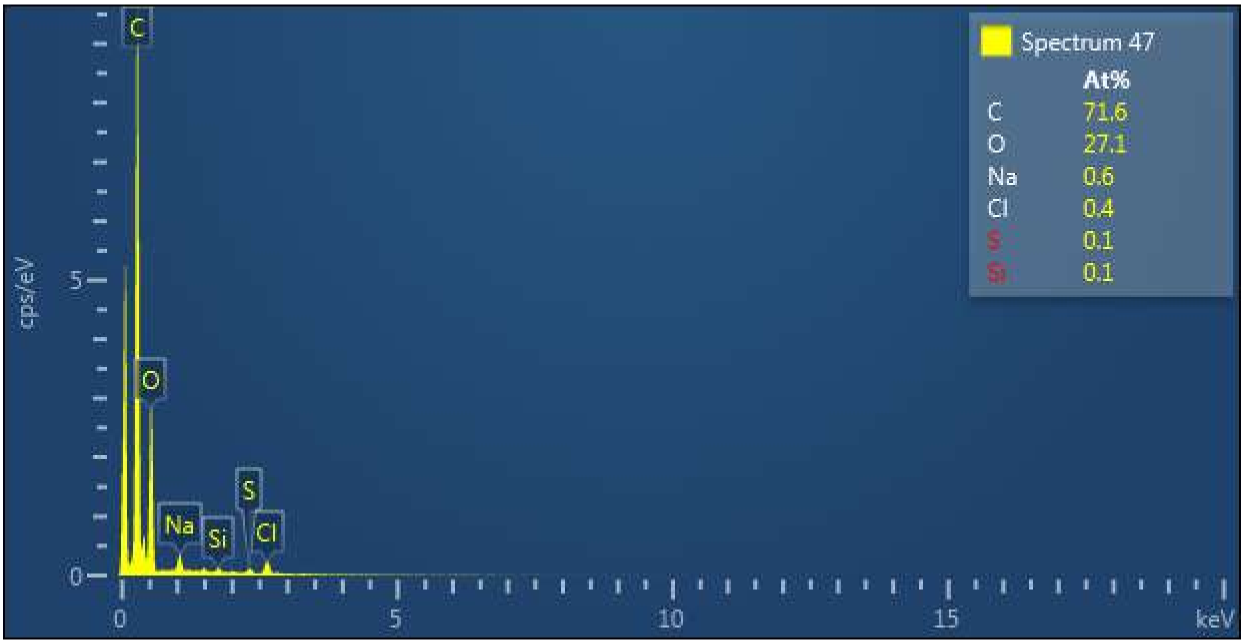

Label:	Spectrum 47
Source:	Acquired
Created:	23/01/2020 10:11:43
Livetime:	60.5s
Process Time:	4
Accelerating Voltage:	15.00kV
Magnification:	89 x
Working Distance:	10.0mm
Specimen Tilt (degrees):	0.0
Elevation (degrees):	35.0
Azimuth (degrees):	0.0
Number Of Channels:	2048
Energy Range (keV):	20 keV
Energy per Channel (eV):	10.0eV
Detector Type Id:	29
Detector Type:	X-Max
Window Type:	SATW
Pulse Pile Up Correction:	Succeeded
Primary Detector:	2617
Primary Detector Serial Number:	77871-X080

Element	Line Type	Apparent Concentration	k Ratio	Wt%	Wt% Sigma	Atomic %	Standard Label	Factory Standard	Standard Calibration Date
C	K series	14.88	0.14880	64.67	0.41	71.60	C Vit	Yes	
O	K series	8.42	0.02833	32.65	0.41	27.14	SiO2	Yes	
Na	K	0.47	0.0020	1.07	0.07	0.62	Albite	Yes	

	series		0						
Si	K series	0.08	0.00067	0.22	0.04	0.10	SiO2	Yes	
S	K series	0.13	0.00113	0.35	0.04	0.14	FeS2	Yes	
Cl	K series	0.37	0.00327	1.03	0.06	0.39	NaCl	Yes	
Total:				100.00		100.00			

Electron Image 15

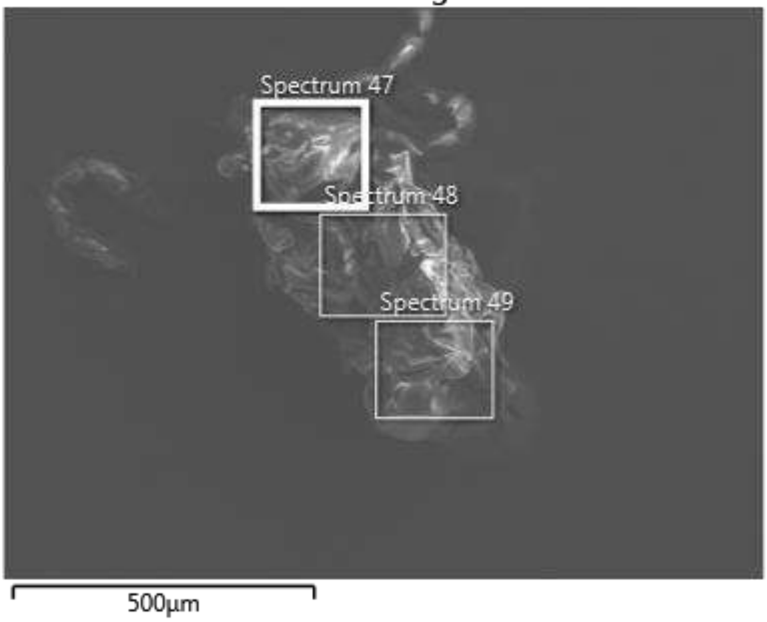

23/01/2020 10:12:55
Project 1
Rumania 10
Dorso 1 15 Kv

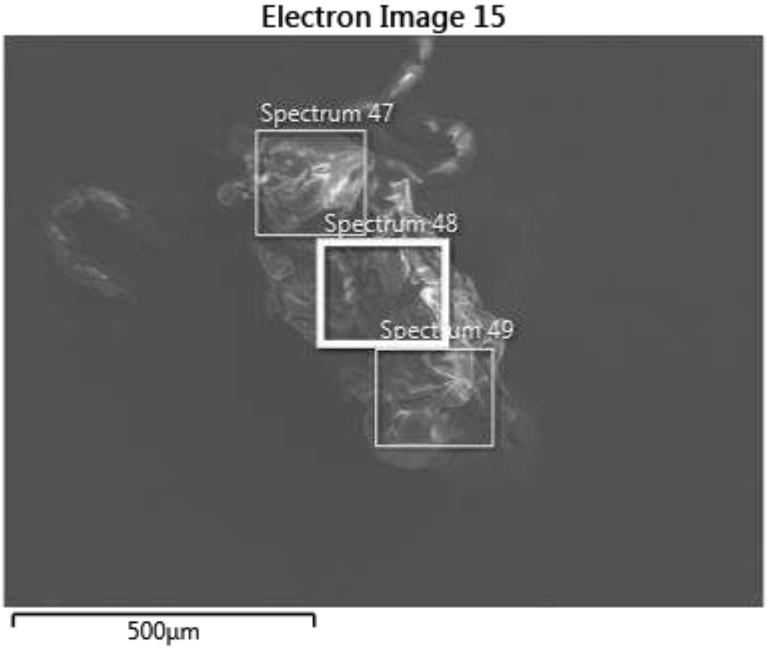

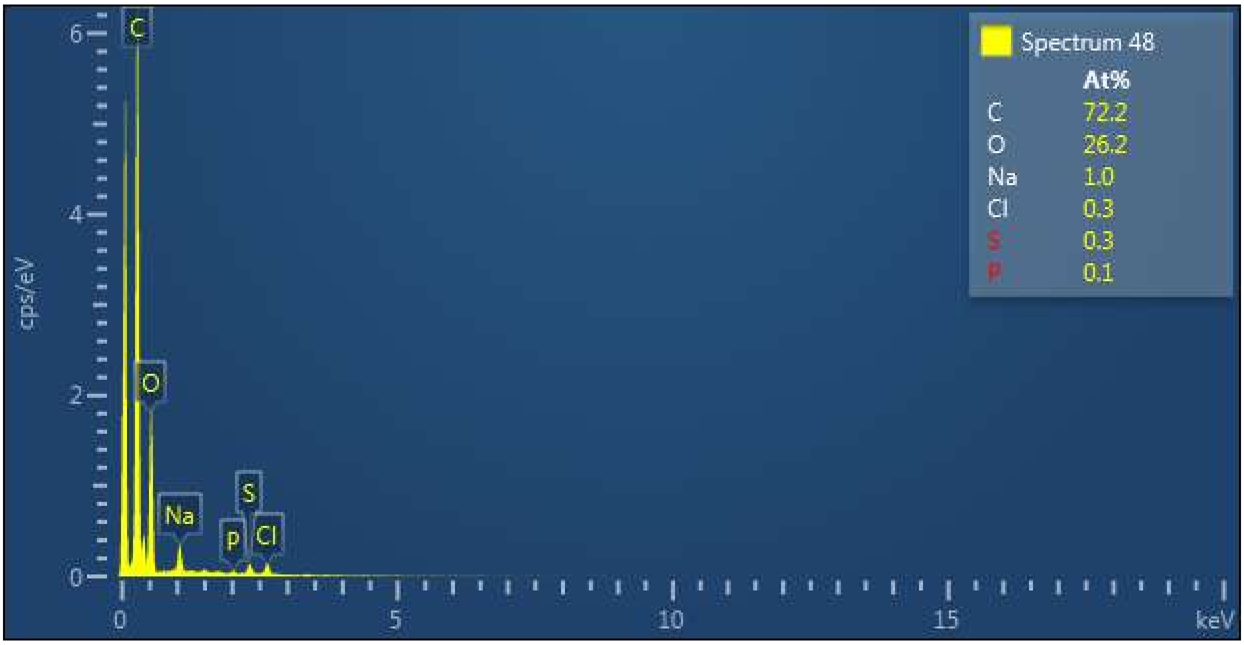

Label:	Spectrum 48
Source:	Acquired
Created:	23/01/2020 10:12:55
Livetime:	60.0s
Process Time:	4
Accelerating Voltage:	15.00kV
Magnification:	89 x
Working Distance:	10.0mm
Specimen Tilt (degrees):	0.0
Elevation (degrees):	35.0
Azimuth (degrees):	0.0
Number Of Channels:	2048
Energy Range (keV):	20 keV
Energy per Channel (eV):	10.0eV
Detector Type Id:	29
Detector Type:	X-Max
Window Type:	SATW
Pulse Pile Up Correction:	Succeeded
Primary Detector:	2617
Primary Detector Serial Number:	77871-X080

Element	Line Type	Apparent Concentration	k Ratio	Wt%	Wt% Sigma	Atomic %	Standard Label	Factory Standard	Standard Calibration Date
C	K series	9.90	0.09896	65.15	0.52	72.15	C Vit	Yes	
O	K series	5.38	0.01810	31.55	0.51	26.23	SiO2	Yes	
Na	K	0.50	0.0021	1.68	0.10	0.97	Albite	Yes	

	series		1						
P	K series	0.07	0.00042	0.20	0.05	0.09	GaP	Yes	
S	K series	0.16	0.00135	0.62	0.06	0.26	FeS2	Yes	
Cl	K series	0.19	0.00166	0.79	0.07	0.30	NaCl	Yes	
Total:				100.00		100.00			

Electron Image 15

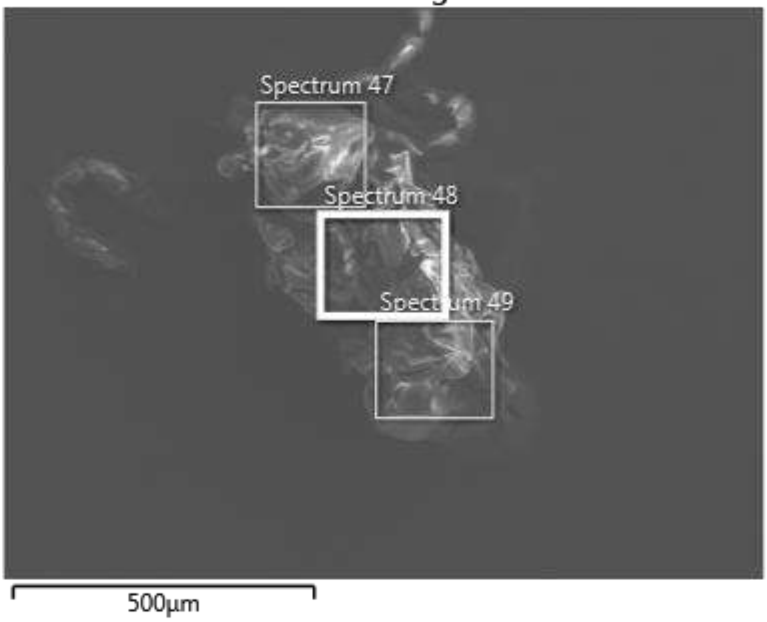

23/01/2020 10:14:05
Project 1
Rumania 10
Dorso 1 15 Kv

Electron Image 15

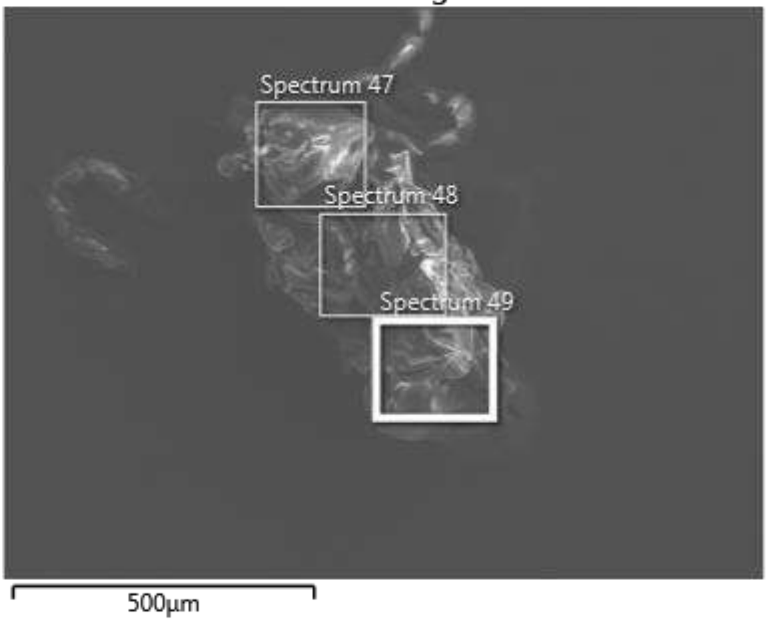

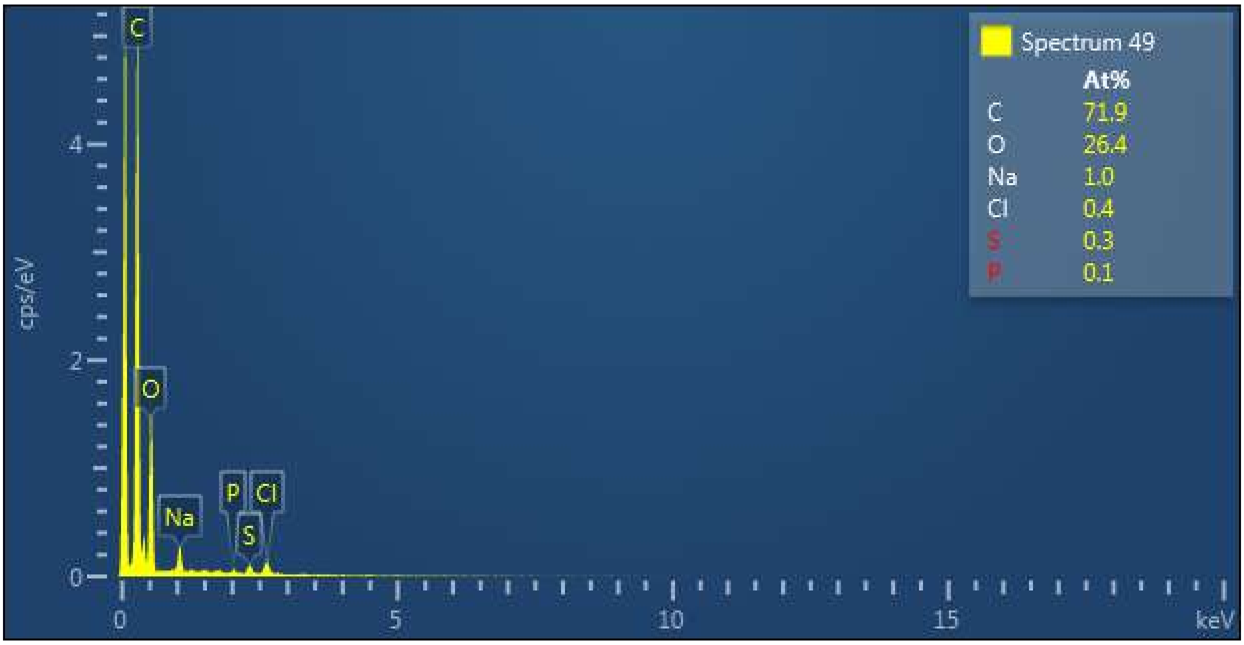

Label:	Spectrum 49
Source:	Acquired
Created:	23/01/2020 10:14:05
Livetime:	60.4s
Process Time:	4
Accelerating Voltage:	15.00kV
Magnification:	89 x
Working Distance:	10.0mm
Specimen Tilt (degrees):	0.0
Elevation (degrees):	35.0
Azimuth (degrees):	0.0
Number Of Channels:	2048
Energy Range (keV):	20 keV
Energy per Channel (eV):	10.0eV
Detector Type Id:	29
Detector Type:	X-Max
Window Type:	SATW
Pulse Pile Up Correction:	Succeeded
Primary Detector:	2617
Primary Detector Serial Number:	77871-X080

Element	Line Type	Apparent Concentration	k Ratio	Wt%	Wt% Sigma	Atomic %	Standard Label	Factory Standard	Standard Calibration Date
C	K series	7.97	0.07967	64.80	0.57	71.91	C Vit	Yes	
O	K series	4.51	0.01518	31.67	0.56	26.39	SiO2	Yes	
Na	K	0.41	0.0017	1.65	0.11	0.96	Albite	Yes	

	series		3						
P	K series	0.07	0.00037	0.21	0.06	0.09	GaP	Yes	
S	K series	0.13	0.00111	0.61	0.07	0.25	FeS2	Yes	
Cl	K series	0.21	0.00186	1.06	0.08	0.40	NaCl	Yes	
Total:				100.00		100.00			

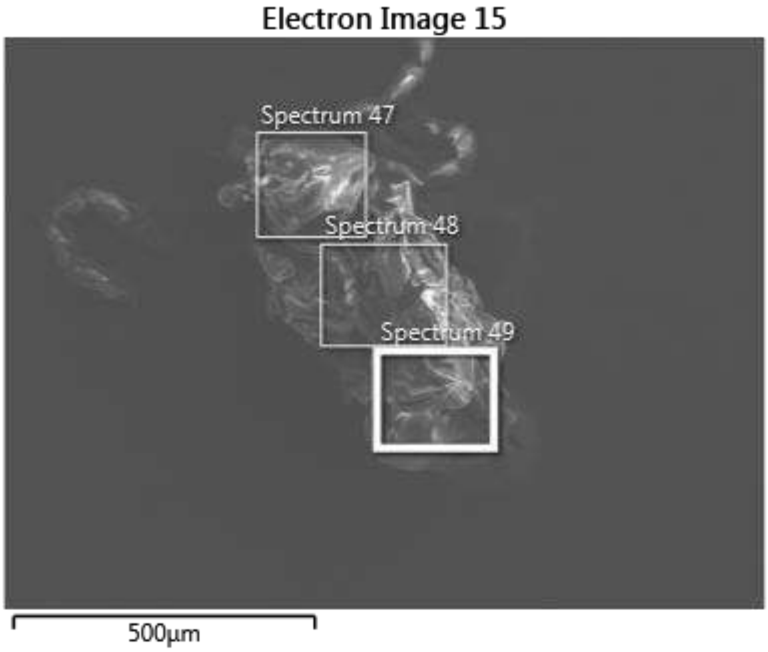

23/01/2020 10:16:54
Project 1
Rumania 4
Pecho 1 15 Kv

Electron Image 17

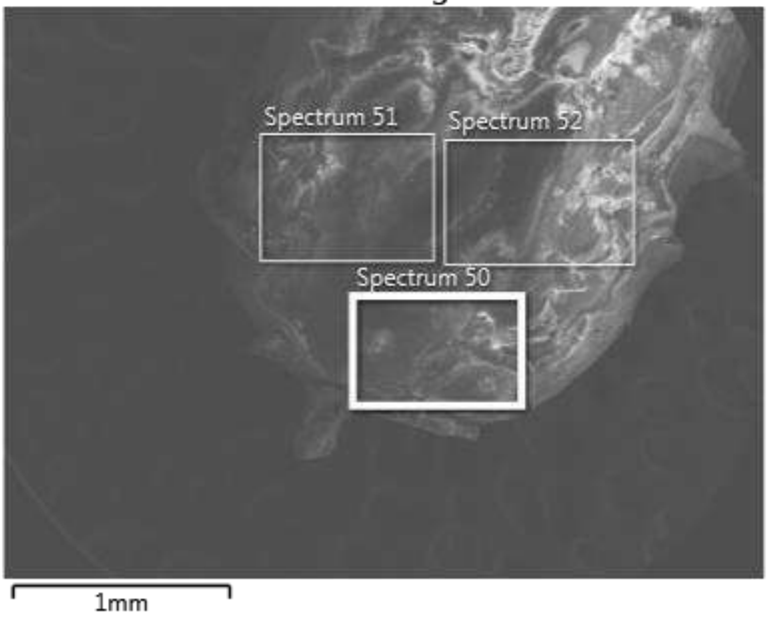

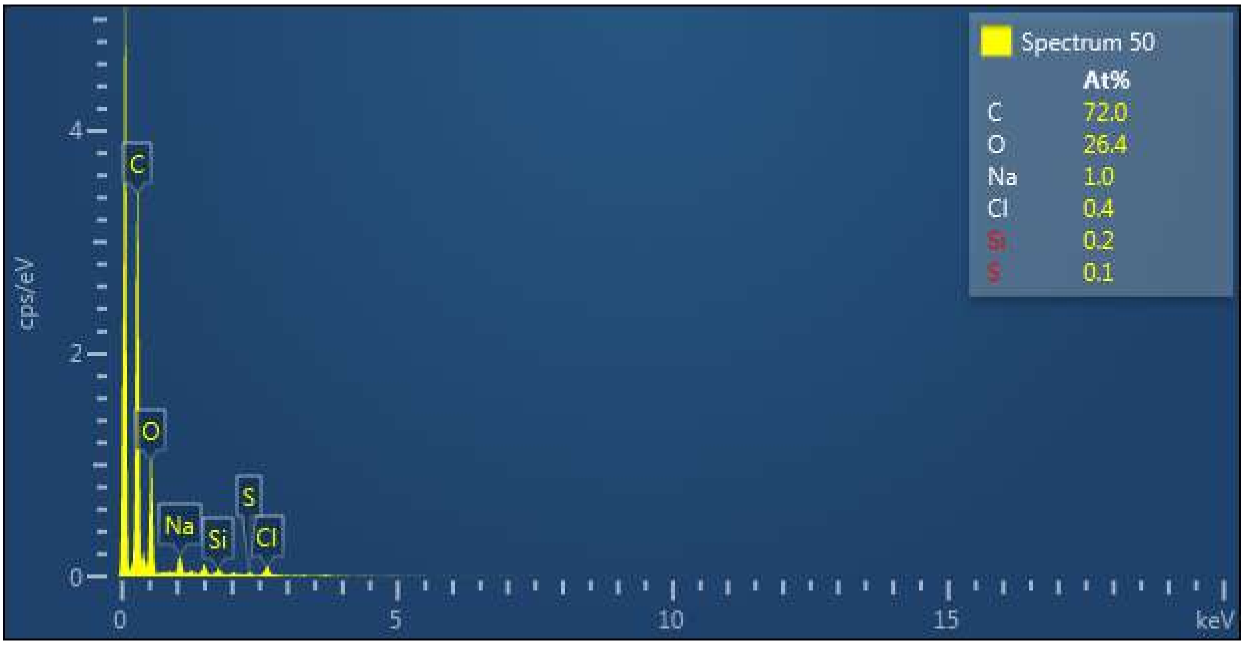

Label:	Spectrum 50
Source:	Acquired
Created:	23/01/2020 10:16:54
Livetime:	60.1s
Process Time:	4
Accelerating Voltage:	15.00kV
Magnification:	32 x
Working Distance:	10.0mm
Specimen Tilt (degrees):	0.0
Elevation (degrees):	35.0
Azimuth (degrees):	0.0
Number Of Channels:	2048
Energy Range (keV):	20 keV
Energy per Channel (eV):	10.0eV
Detector Type Id:	29
Detector Type:	X-Max
Window Type:	SATW
Pulse Pile Up Correction:	Succeeded
Primary Detector:	2617
Primary Detector Serial Number:	77871-X080

Element	Line Type	Apparent Concentration	k Ratio	Wt%	Wt% Sigma	Atomic %	Standard Label	Factory Standard	Standard Calibration Date
C	K series	5.58	0.05584	64.93	0.67	71.96	C Vit	Yes	
O	K series	3.07	0.01032	31.69	0.67	26.37	SiO2	Yes	
Na	K	0.29	0.0012	1.72	0.13	1.00	Albite	Yes	

	series		2						
Si	K series	0.06	0.00050	0.43	0.07	0.21	SiO2	Yes	
S	K series	0.04	0.00034	0.28	0.06	0.12	FeS2	Yes	
Cl	K series	0.13	0.00113	0.95	0.09	0.36	NaCl	Yes	
Total:				100.00		100.00			

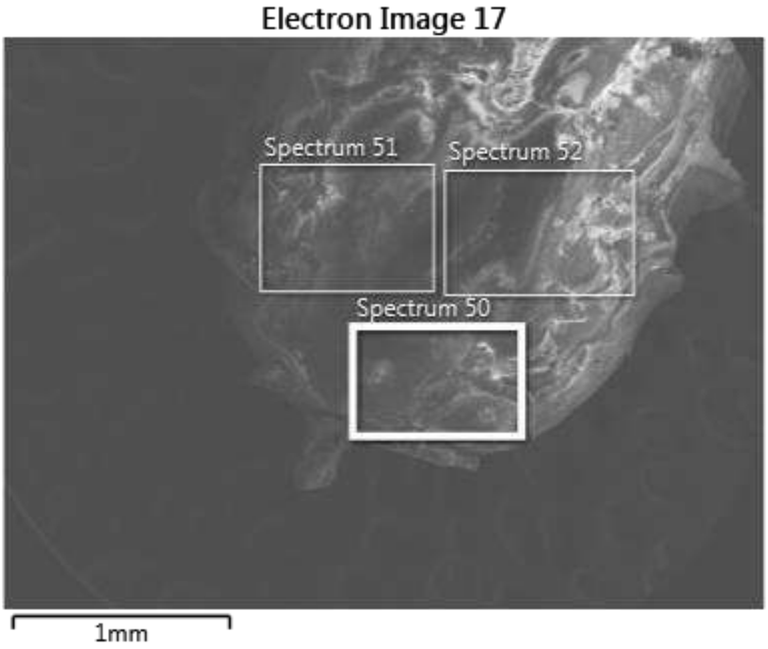

23/01/2020 10:18:02
Project 1
Rumania 4
Pecho 1 15 Kv

Electron Image 17

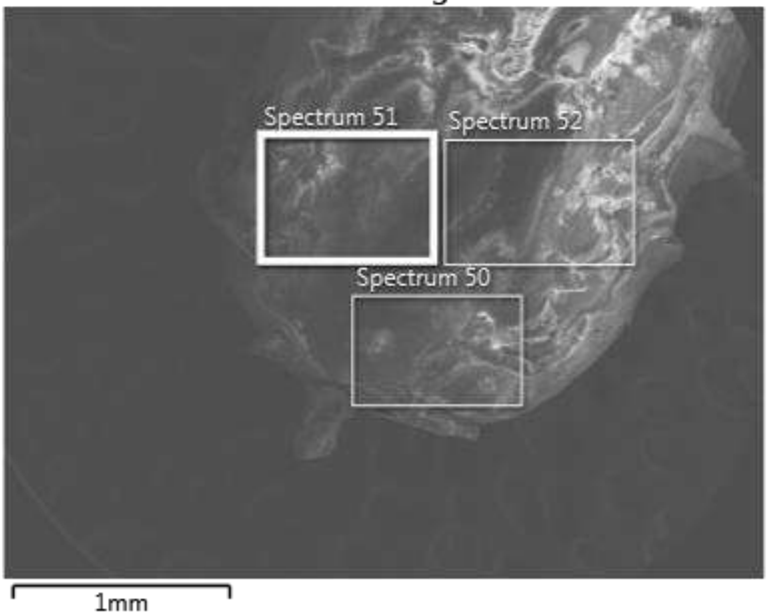

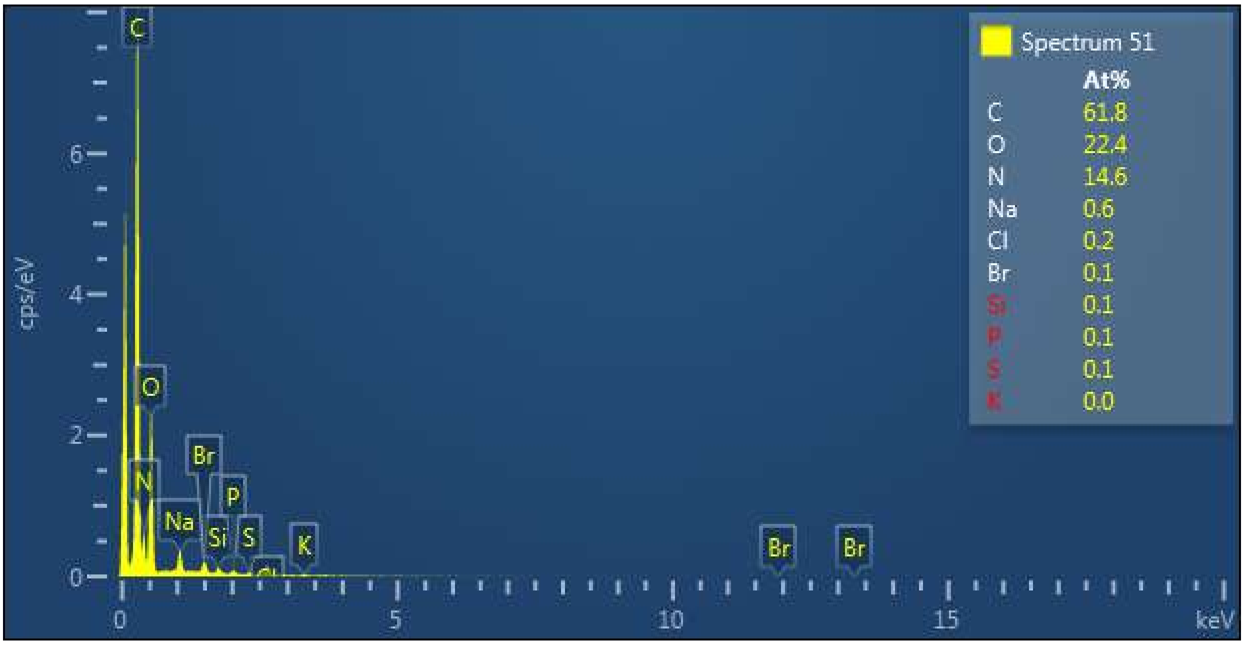

Label:	Spectrum 51
Source:	Acquired
Created:	23/01/2020 10:18:02
Livetime:	60.0s
Process Time:	4
Accelerating Voltage:	15.00kV
Magnification:	32 x
Working Distance:	10.0mm
Specimen Tilt (degrees):	0.0
Elevation (degrees):	35.0
Azimuth (degrees):	0.0
Number Of Channels:	2048
Energy Range (keV):	20 keV
Energy per Channel (eV):	10.0eV
Detector Type Id:	29
Detector Type:	X-Max
Window Type:	SATW
Pulse Pile Up Correction:	Succeeded
Primary Detector:	2617
Primary Detector Serial Number:	77871-X080

Element	Line Type	Apparent Concentration	k Ratio	Wt%	Wt% Sigma	Atomic %	Standard Label	Factory Standard	Standard Calibration Date
C	K series	14.83	0.14833	55.20	0.84	61.84	C Vit	Yes	
N	K series	4.83	0.00860	15.16	1.09	14.56	BN	Yes	
O	K	6.65	0.0223	26.60	0.54	22.37	SiO2	Yes	

	series		8						
Na	K series	0.52	0.00221	1.03	0.06	0.60	Albite	Yes	
Si	K series	0.09	0.00073	0.21	0.03	0.10	SiO2	Yes	
P	K series	0.11	0.00060	0.17	0.03	0.07	GaP	Yes	
S	K series	0.05	0.00046	0.12	0.03	0.05	FeS2	Yes	
Cl	K series	0.24	0.00214	0.59	0.04	0.22	NaCl	Yes	
K	K series	0.04	0.00037	0.10	0.03	0.03	KBr	Yes	
Br	L series	0.28	0.00250	0.82	0.08	0.14	KBr	Yes	
Total:				100.00		100.00			

Electron Image 17

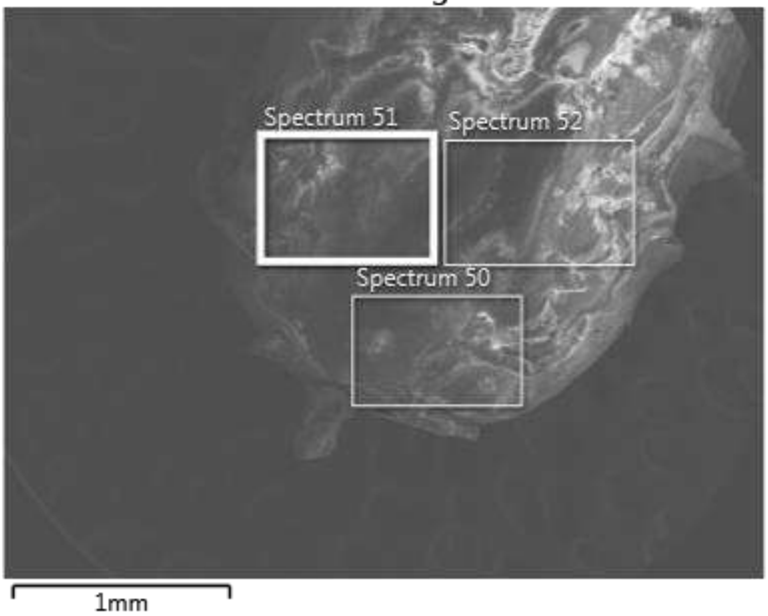

23/01/2020 10:19:11
Project 1
Rumania 4
Pecho 1 15 Kv

Electron Image 17

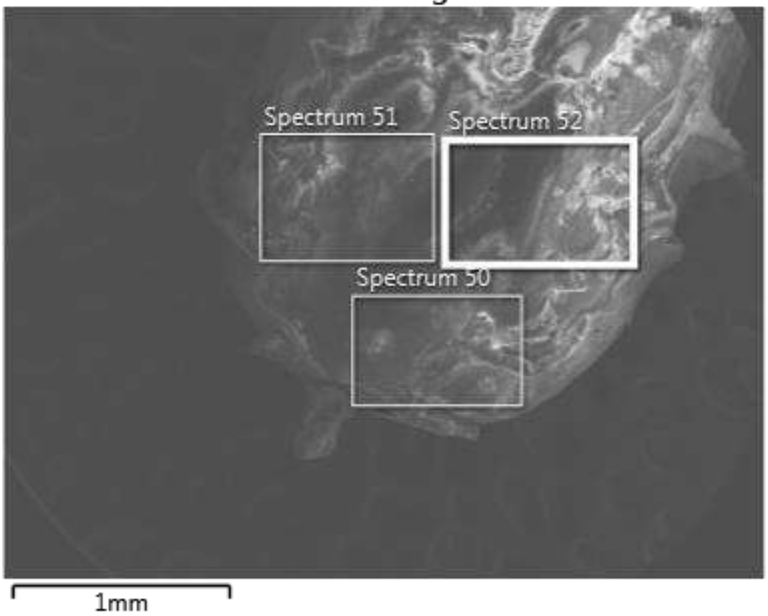

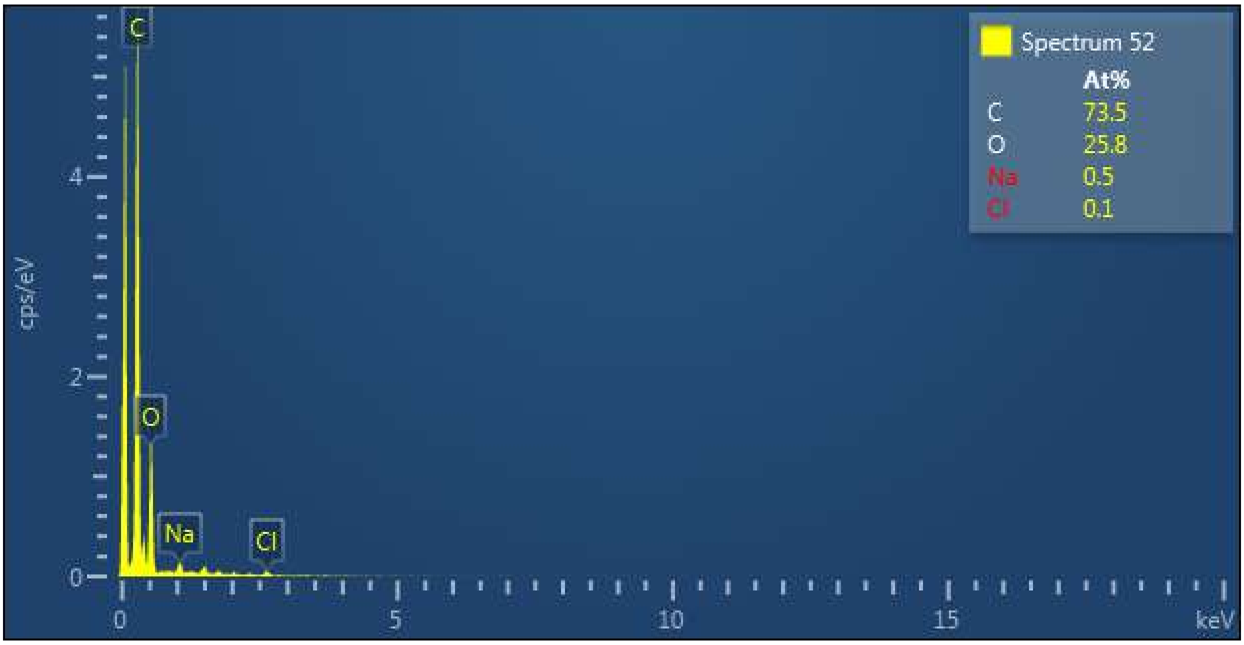

Label:	Spectrum 52
Source:	Acquired
Created:	23/01/2020 10:19:11
Livetime:	60.0s
Process Time:	4
Accelerating Voltage:	15.00kV
Magnification:	32 x
Working Distance:	10.0mm
Specimen Tilt (degrees):	0.0
Elevation (degrees):	35.0
Azimuth (degrees):	0.0
Number Of Channels:	2048
Energy Range (keV):	20 keV
Energy per Channel (eV):	10.0eV
Detector Type Id:	29
Detector Type:	X-Max
Window Type:	SATW
Pulse Pile Up Correction:	Succeeded
Primary Detector:	2617
Primary Detector Serial Number:	77871-X080

Element	Line Type	Apparent Concentration	k Ratio	Wt%	Wt% Sigma	Atomic %	Standard Label	Factory Standard	Standard Calibration Date
C	K series	8.84	0.08837	67.29	0.58	73.54	C Vit	Yes	
O	K series	3.93	0.01321	31.49	0.58	25.84	SiO2	Yes	
Na	K	0.18	0.0007	0.82	0.09	0.47	Albite	Yes	

	series		5						
Cl	K series	0.07	0.00063	0.40	0.06	0.15	NaCl	Yes	
Total:				100.00		100.00			

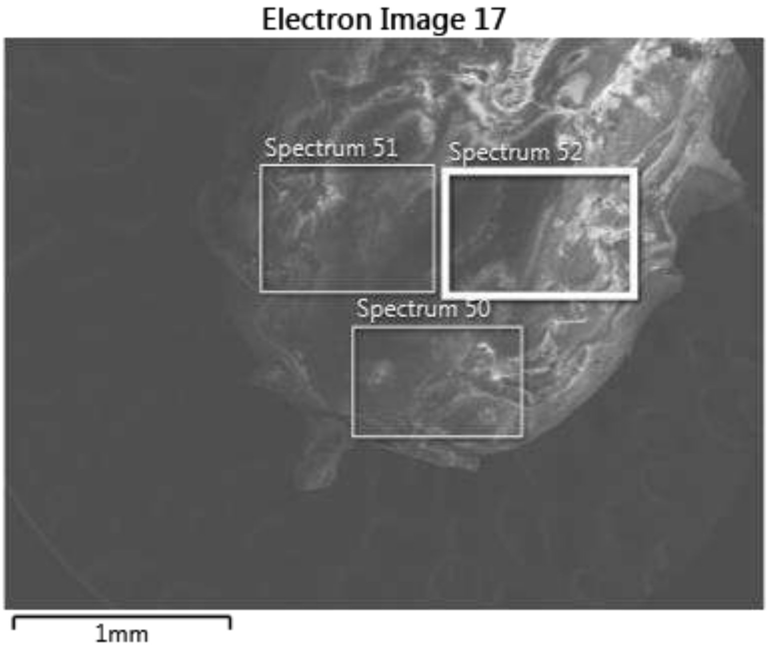

23/01/2020 10:28:16
Project 1
Rumania 4
Ventral 1 15 Kv

Electron Image 18

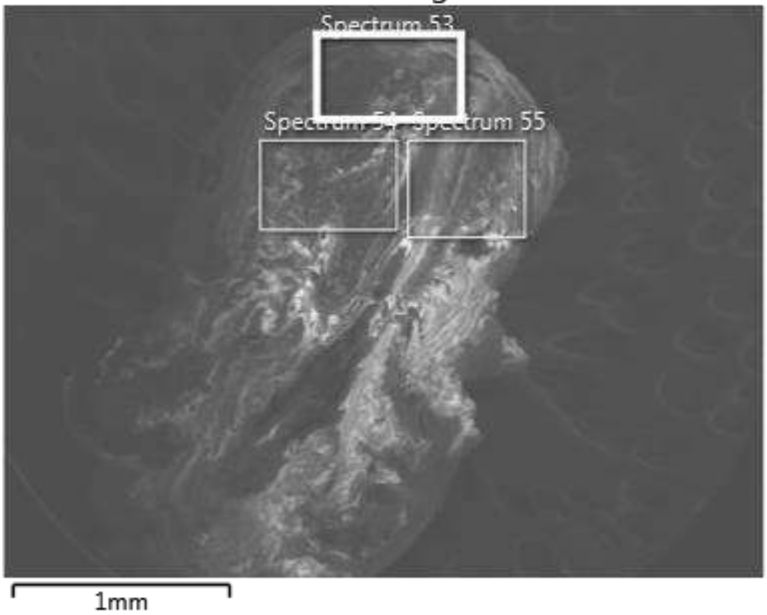

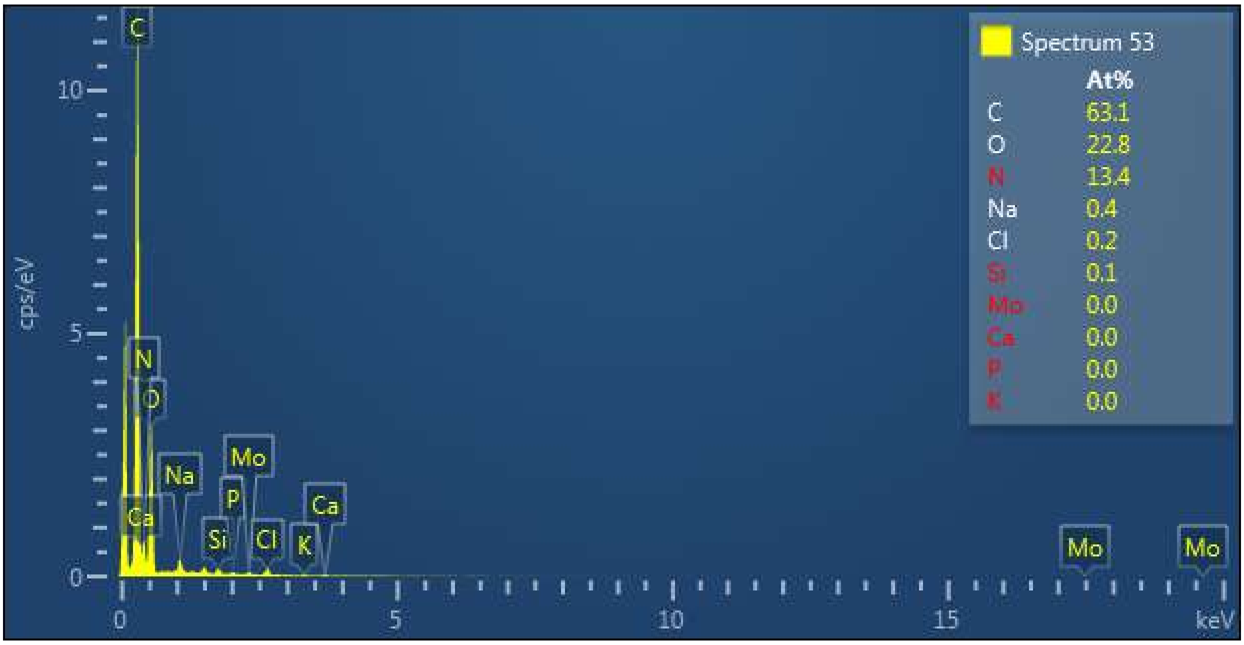

Label:	Spectrum 53
Source:	Acquired
Created:	23/01/2020 10:28:16
Livetime:	60.3s
Process Time:	4
Accelerating Voltage:	15.00kV
Magnification:	32 x
Working Distance:	10.0mm
Specimen Tilt (degrees):	0.0
Elevation (degrees):	35.0
Azimuth (degrees):	0.0
Number Of Channels:	2048
Energy Range (keV):	20 keV
Energy per Channel (eV):	10.0eV
Detector Type Id:	29
Detector Type:	X-Max
Window Type:	SATW
Pulse Pile Up Correction:	Succeeded
Primary Detector:	2617
Primary Detector Serial Number:	77871-X080

Element	Line Type	Apparent Concentration	k Ratio	Wt%	Wt% Sigma	Atomic %	Standard Label	Factory Standard	Standard Calibration Date
C	K series	21.50	0.21498	56.78	1.00	63.07	C Vit	Yes	
N	K series	5.69	0.01012	14.02	1.38	13.36	BN	Yes	
O	K	8.92	0.0300	27.32	0.58	22.79	SiO2	Yes	

	series		1						
Na	K series	0.43	0.00180	0.65	0.05	0.38	Albite	Yes	
Si	K series	0.14	0.00108	0.24	0.03	0.11	SiO2	Yes	
P	K series	0.07	0.00037	0.08	0.03	0.03	GaP	Yes	
Cl	K series	0.22	0.00193	0.41	0.03	0.15	NaCl	Yes	
K	K series	0.05	0.00039	0.08	0.03	0.03	KBr	Yes	
Ca	K series	0.06	0.00053	0.11	0.03	0.04	Wollastonite	Yes	
Mo	L series	0.14	0.00136	0.31	0.08	0.04	Mo	Yes	
Total:				100.00		100.00			

Electron Image 18

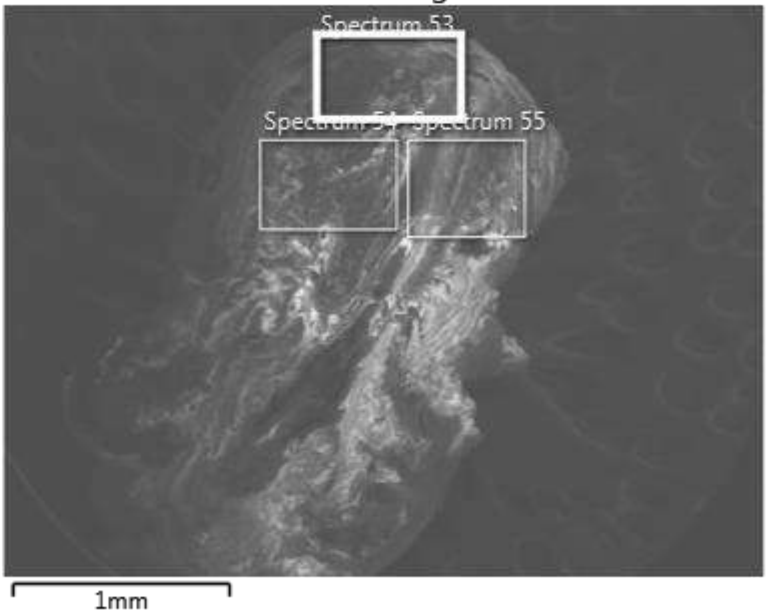

23/01/2020 10:29:27
Project 1
Rumania 4
Ventral 1 15 Kv

Electron Image 18

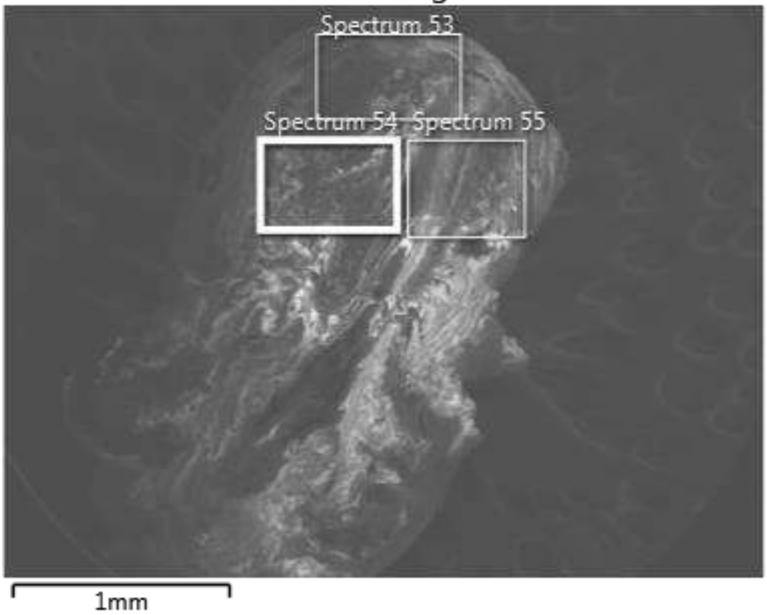

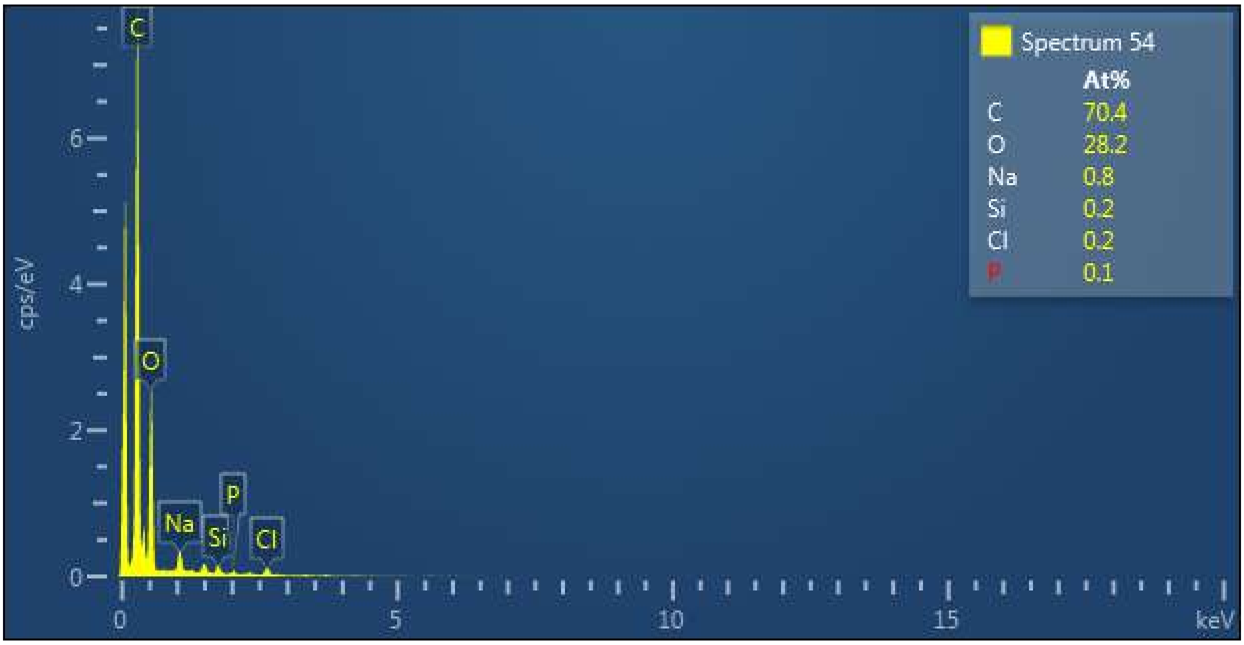

Label:	Spectrum 54
Source:	Acquired
Created:	23/01/2020 10:29:27
Livetime:	60.2s
Process Time:	4
Accelerating Voltage:	15.00kV
Magnification:	32 x
Working Distance:	10.0mm
Specimen Tilt (degrees):	0.0
Elevation (degrees):	35.0
Azimuth (degrees):	0.0
Number Of Channels:	2048
Energy Range (keV):	20 keV
Energy per Channel (eV):	10.0eV
Detector Type Id:	29
Detector Type:	X-Max
Window Type:	SATW
Pulse Pile Up Correction:	Succeeded
Primary Detector:	2617
Primary Detector Serial Number:	77871-X080

Element	Line Type	Apparent Concentration	k Ratio	Wt%	Wt% Sigma	Atomic %	Standard Label	Factory Standard	Standard Calibration Date
C	K series	12.06	0.12056	63.41	0.46	70.39	C Vit	Yes	
O	K series	7.17	0.02412	33.88	0.46	28.24	SiO2	Yes	
Na	K	0.51	0.0021	1.46	0.09	0.85	Albite	Yes	

	serie s		7						
Si	K serie s	0.14	0.0011 2	0.46	0.05	0.22	SiO2	Yes	
P	K serie s	0.09	0.0005 2	0.21	0.04	0.09	GaP	Yes	
Cl	K serie s	0.17	0.0014 6	0.58	0.05	0.22	NaCl	Yes	
Total:				100.0 0		100.0 0			

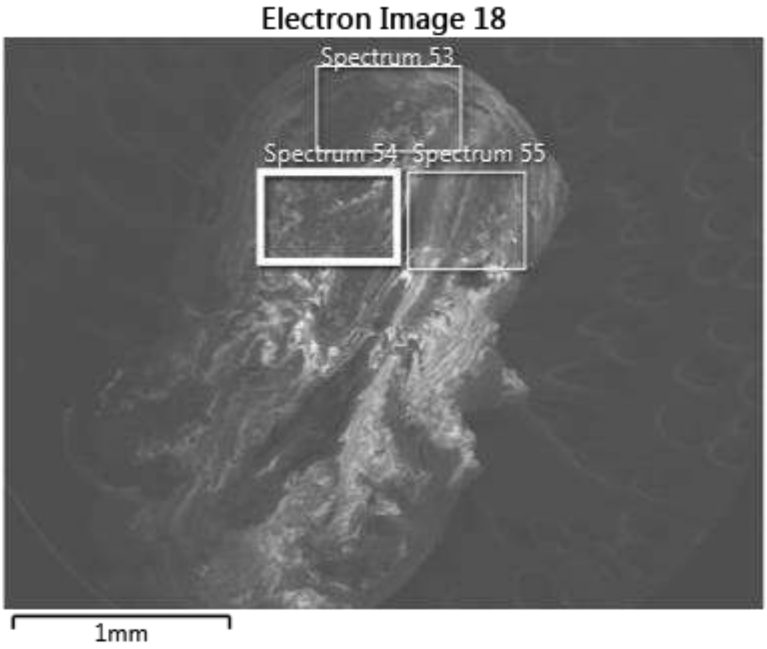

23/01/2020 10:30:37
Project 1
Rumania 4
Ventral 1 15 Kv

Electron Image 18

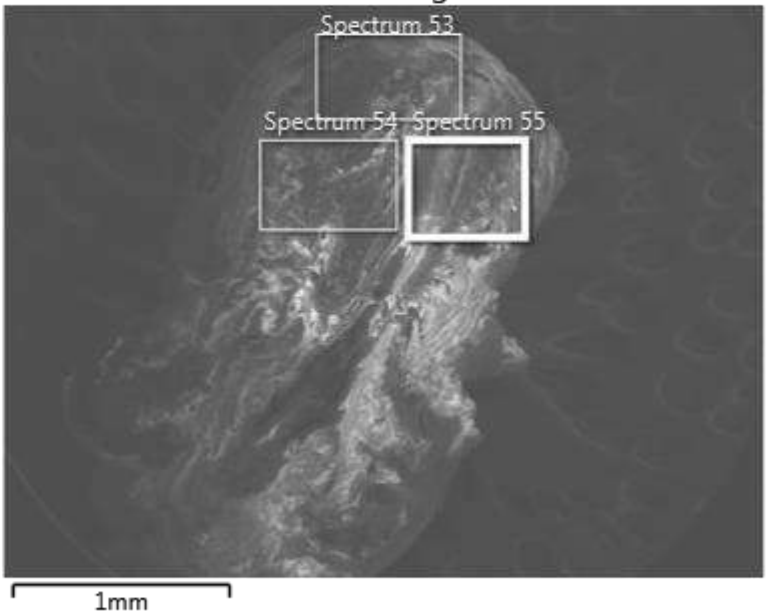

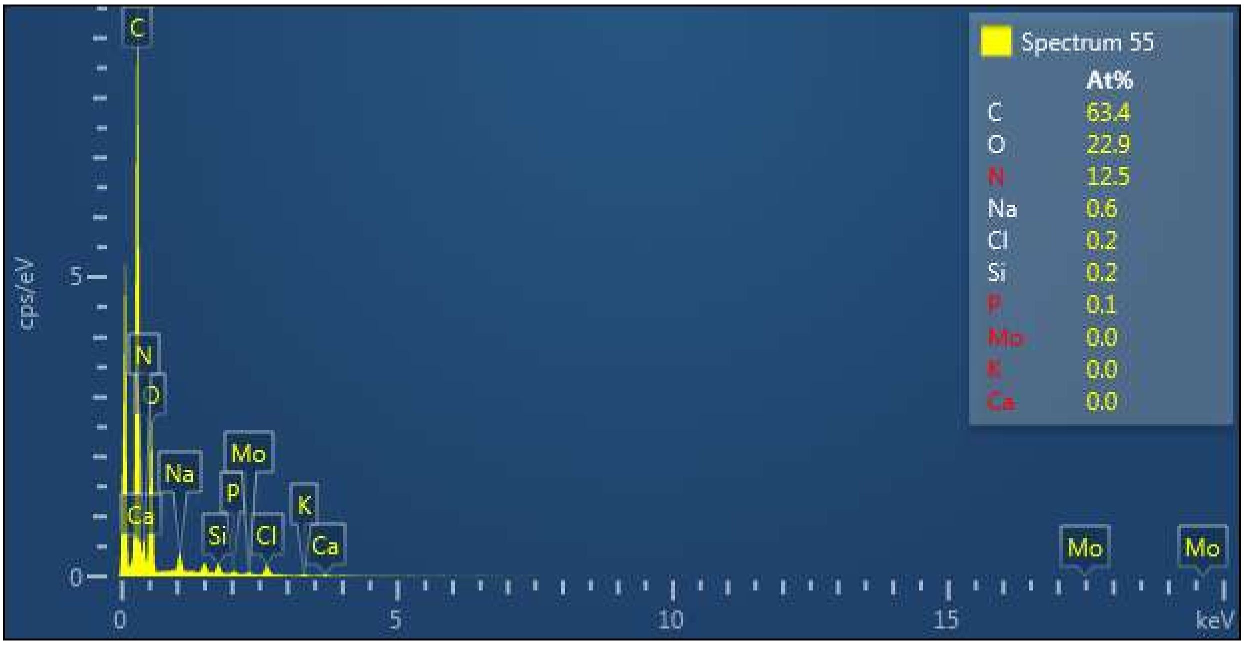

Label:	Spectrum 55
Source:	Acquired
Created:	23/01/2020 10:30:37
Livetime:	60.4s
Process Time:	4
Accelerating Voltage:	15.00kV
Magnification:	32 x
Working Distance:	10.0mm
Specimen Tilt (degrees):	0.0
Elevation (degrees):	35.0
Azimuth (degrees):	0.0
Number Of Channels:	2048
Energy Range (keV):	20 keV
Energy per Channel (eV):	10.0eV
Detector Type Id:	29
Detector Type:	X-Max
Window Type:	SATW
Pulse Pile Up Correction:	Succeeded
Primary Detector:	2617
Primary Detector Serial Number:	77871-X080

Element	Line Type	Apparent Concentration	k Ratio	Wt%	Wt% Sigma	Atomic %	Standard Label	Factory Standard	Standard Calibration Date
C	K series	17.25	0.17250	56.88	1.10	63.41	C Vit	Yes	
N	K series	4.39	0.00782	13.07	1.52	12.50	BN	Yes	
O	K	7.57	0.0254	27.38	0.64	22.92	SiO2	Yes	

	series		7						
Na	K series	0.54	0.00228	0.98	0.06	0.57	Albite	Yes	
Si	K series	0.20	0.00159	0.42	0.03	0.20	SiO2	Yes	
P	K series	0.09	0.00050	0.13	0.03	0.06	GaP	Yes	
Cl	K series	0.27	0.00232	0.58	0.04	0.22	NaCl	Yes	
K	K series	0.05	0.00046	0.12	0.03	0.04	KBr	Yes	
Ca	K series	0.05	0.00047	0.11	0.03	0.04	Wollastonite	Yes	
Mo	L series	0.12	0.00122	0.33	0.09	0.05	Mo	Yes	
Total:				100.00		100.00			

Electron Image 18

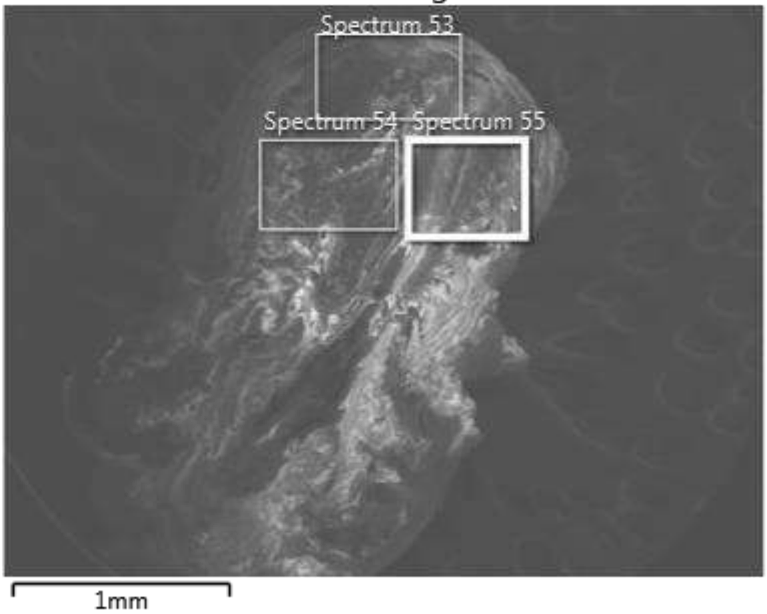

23/01/2020 12:21:35
Project 1
Rumania 5
Pecho 1 15 Kv

Electron Image 19

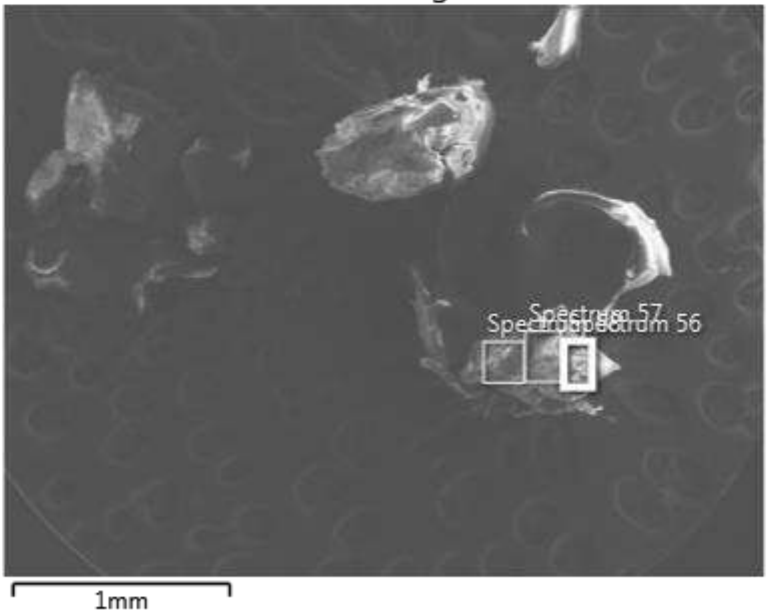

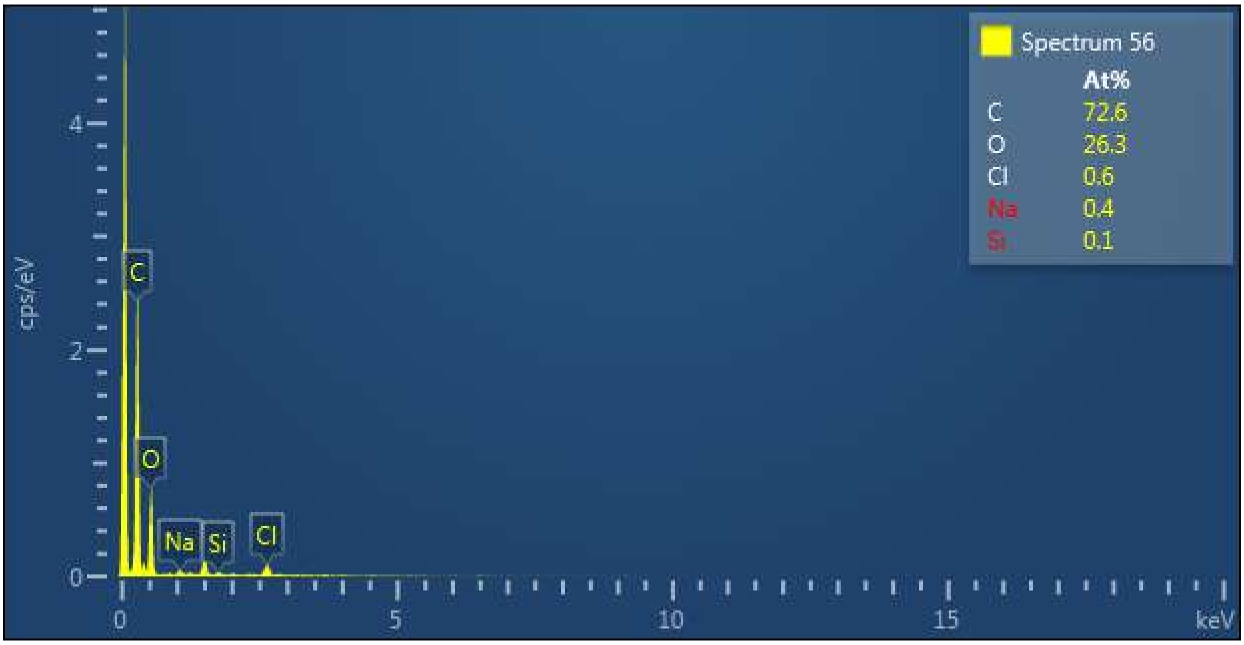

Label:	Spectrum 56
Source:	Acquired
Created:	23/01/2020 12:21:35
Livetime:	60.1s
Process Time:	4
Accelerating Voltage:	15.00kV
Magnification:	32 x
Working Distance:	10.0mm
Specimen Tilt (degrees):	0.0
Elevation (degrees):	35.0
Azimuth (degrees):	0.0
Number Of Channels:	2048
Energy Range (keV):	20 keV
Energy per Channel (eV):	10.0eV
Detector Type Id:	29
Detector Type:	X-Max
Window Type:	SATW
Pulse Pile Up Correction:	Succeeded
Primary Detector:	2617
Primary Detector Serial Number:	77871-X080

Element	Line Type	Apparent Concentration	k Ratio	Wt%	Wt% Sigma	Atomic %	Standard Label	Factory Standard	Standard Calibration Date
C	K series	4.02	0.04016	65.71	0.79	72.58	C Vit	Yes	
O	K series	2.17	0.00731	31.71	0.78	26.30	SiO2	Yes	
Na	K	0.07	0.0003	0.62	0.12	0.36	Albite	Yes	

	series		2						
Si	K series	0.03	0.00025	0.31	0.07	0.15	SiO2	Yes	
Cl	K series	0.16	0.00142	1.65	0.14	0.62	NaCl	Yes	
Total:				100.00		100.00			

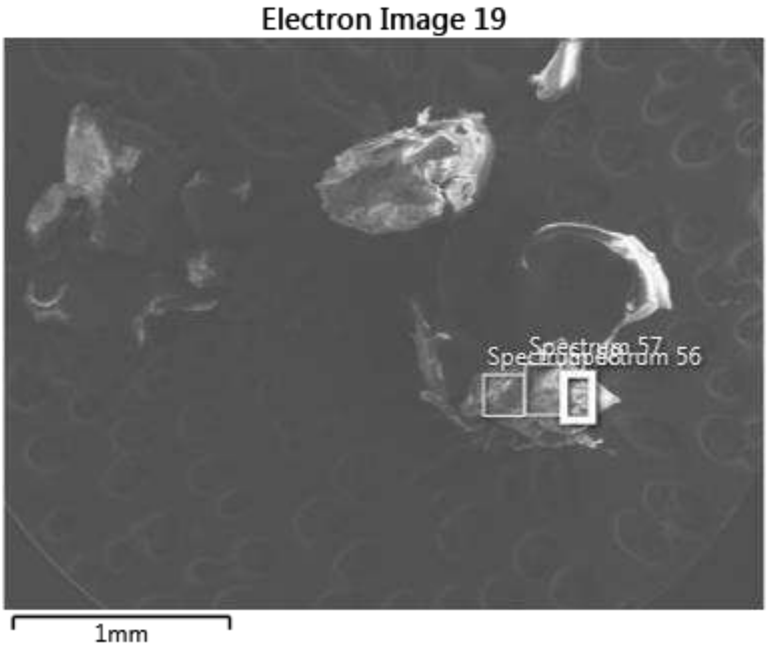

23/01/2020 12:22:43
Project 1
Rumania 5
Pecho 1 15 Kv

Electron Image 19

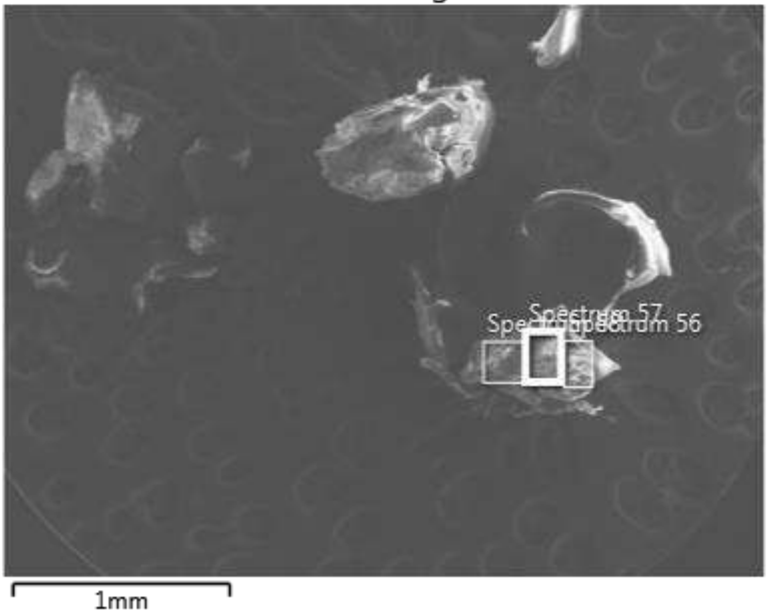

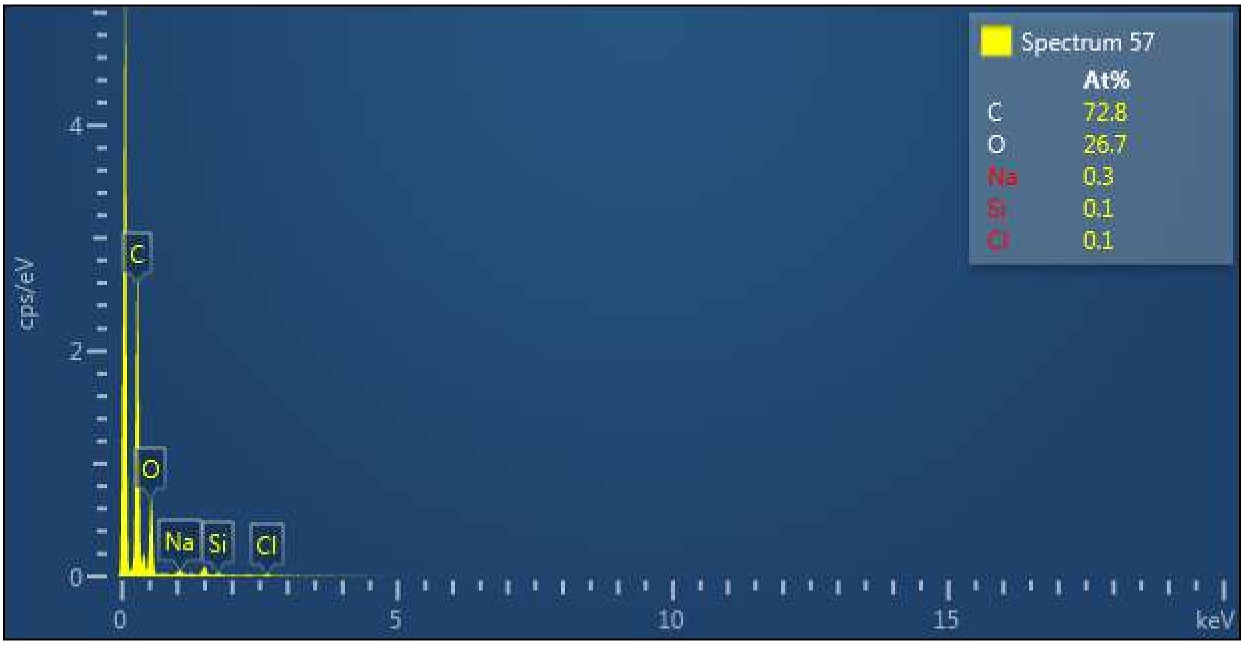

Label:	Spectrum 57
Source:	Acquired
Created:	23/01/2020 12:22:43
Livetime:	60.1s
Process Time:	4
Accelerating Voltage:	15.00kV
Magnification:	32 x
Working Distance:	10.0mm
Specimen Tilt (degrees):	0.0
Elevation (degrees):	35.0
Azimuth (degrees):	0.0
Number Of Channels:	2048
Energy Range (keV):	20 keV
Energy per Channel (eV):	10.0eV
Detector Type Id:	29
Detector Type:	X-Max
Window Type:	SATW
Pulse Pile Up Correction:	Succeeded
Primary Detector:	2617
Primary Detector Serial Number:	77871-X080

Element	Line Type	Apparent Concentration	k Ratio	Wt%	Wt% Sigma	Atomic %	Standard Label	Factory Standard	Standard Calibration Date
C	K series	4.20	0.04195	66.46	0.84	72.79	C Vit	Yes	
O	K series	1.99	0.00669	32.44	0.83	26.67	SiO2	Yes	
Na	K	0.05	0.0002	0.48	0.13	0.28	Albite	Yes	

	series		1						
Si	K series	0.03	0.00021	0.28	0.08	0.13	SiO2	Yes	
Cl	K series	0.03	0.00026	0.34	0.09	0.13	NaCl	Yes	
Total:				100.00		100.00			

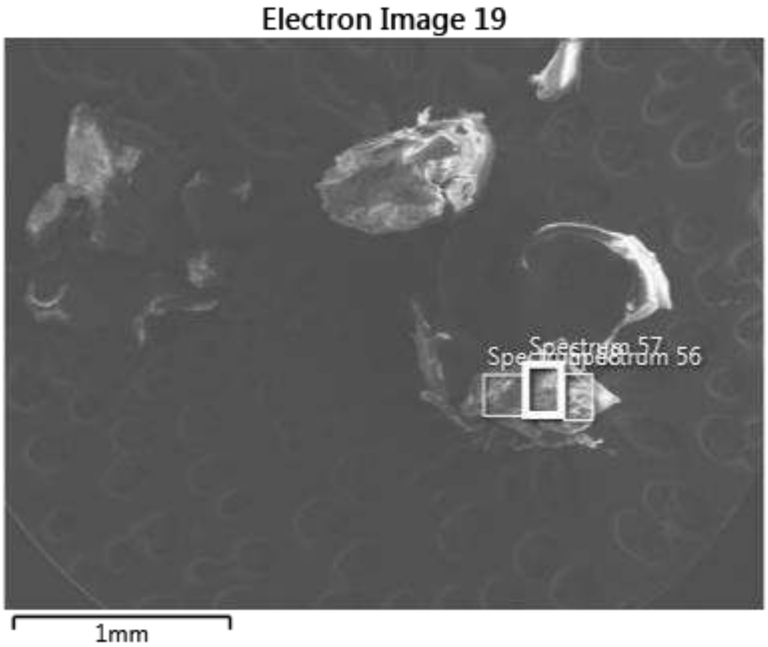

23/01/2020 12:23:52
Project 1
Rumania 5
Pecho 1 15 Kv

Electron Image 19

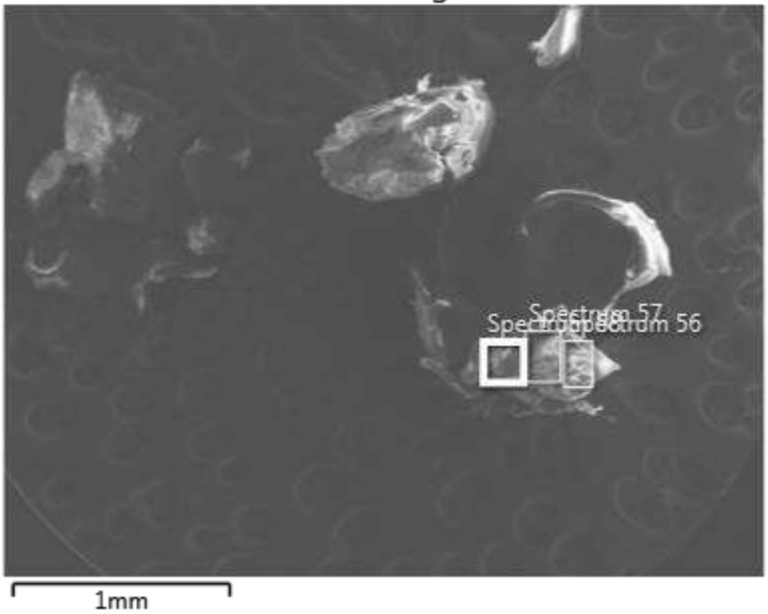

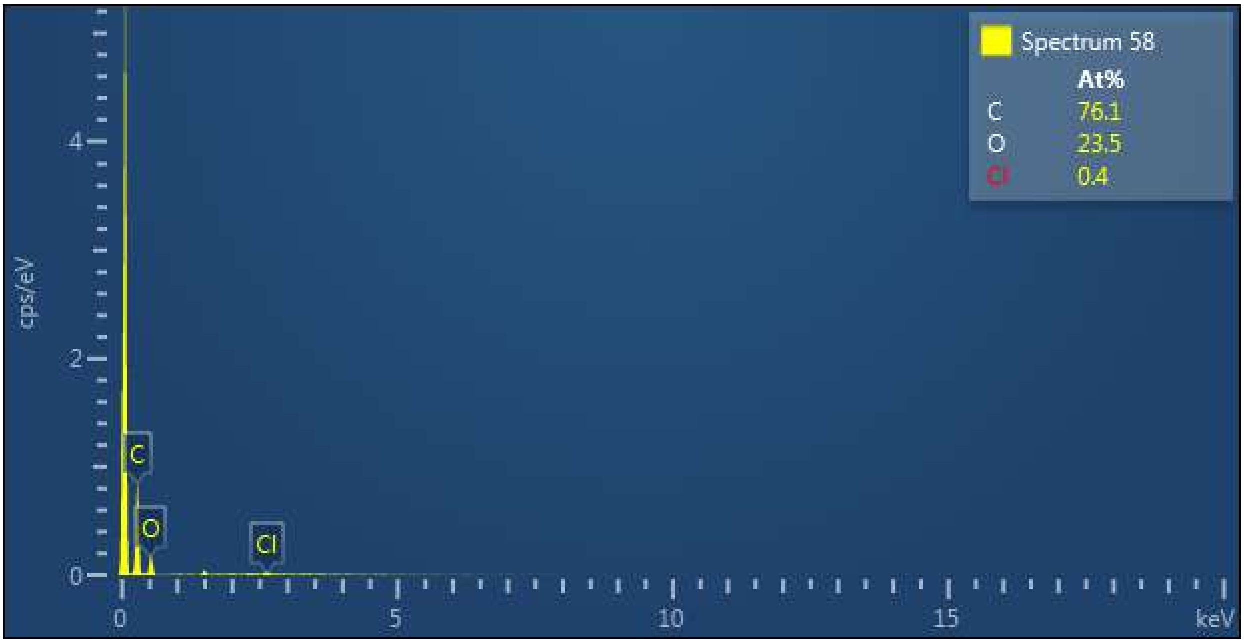

Label:	Spectrum 58
Source:	Acquired
Created:	23/01/2020 12:23:52
Livetime:	60.1s
Process Time:	4
Accelerating Voltage:	15.00kV
Magnification:	32 x
Working Distance:	10.0mm
Specimen Tilt (degrees):	0.0
Elevation (degrees):	35.0
Azimuth (degrees):	0.0
Number Of Channels:	2048
Energy Range (keV):	20 keV
Energy per Channel (eV):	10.0eV
Detector Type Id:	29
Detector Type:	X-Max
Window Type:	SATW
Pulse Pile Up Correction:	Succeeded
Primary Detector:	2617
Primary Detector Serial Number:	77871-X080

Element	Line Type	Apparent Concentration	k Ratio	Wt%	Wt% Sigma	Atomic %	Standard Label	Factory Standard	Standard Calibration Date
C	K series	1.39	0.01393	70.04	1.41	76.06	C Vit	Yes	
O	K series	0.54	0.00181	28.88	1.40	23.55	SiO2	Yes	
Cl	K	0.03	0.0002	1.08	0.30	0.40	NaCl	Yes	

	serie s		7						
Total:				100.0 0		100.0 0			

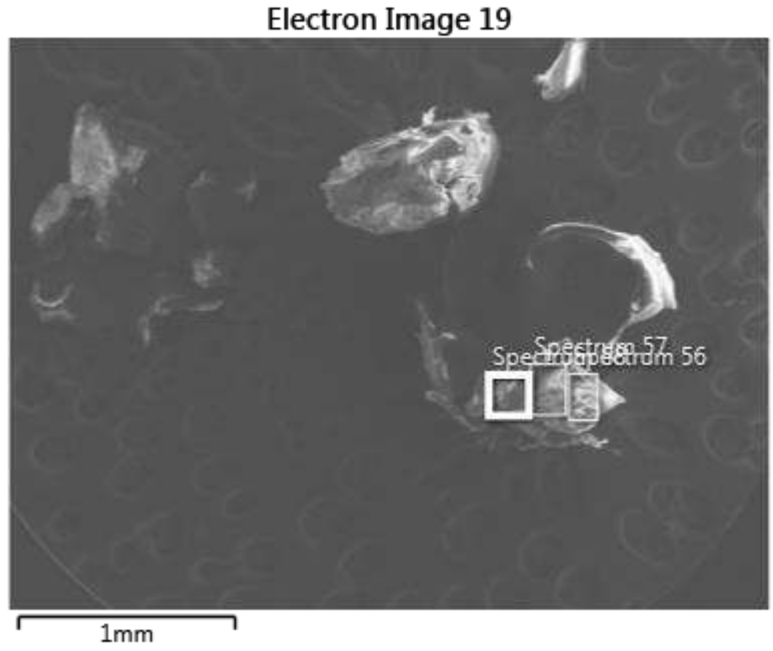

23/01/2020 12:25:45
Project 1
Rumania 5
Ventral 1 15 Kv

Electron Image 20

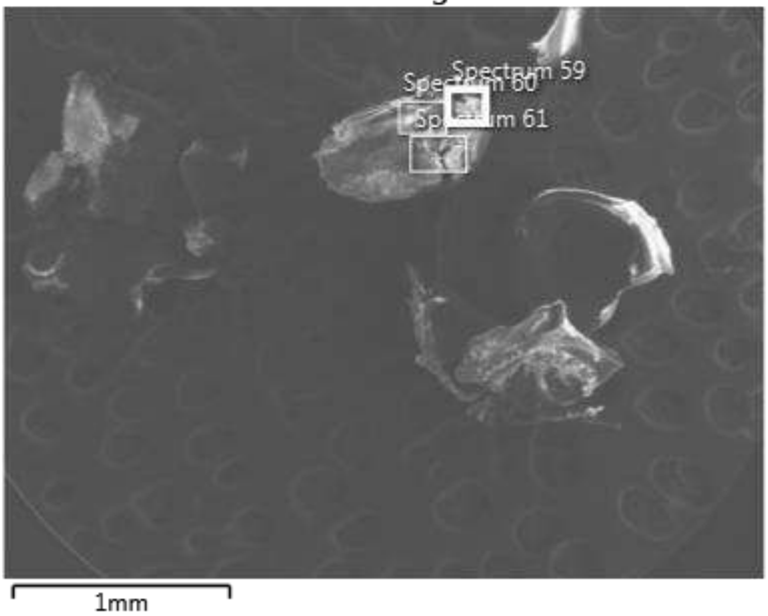

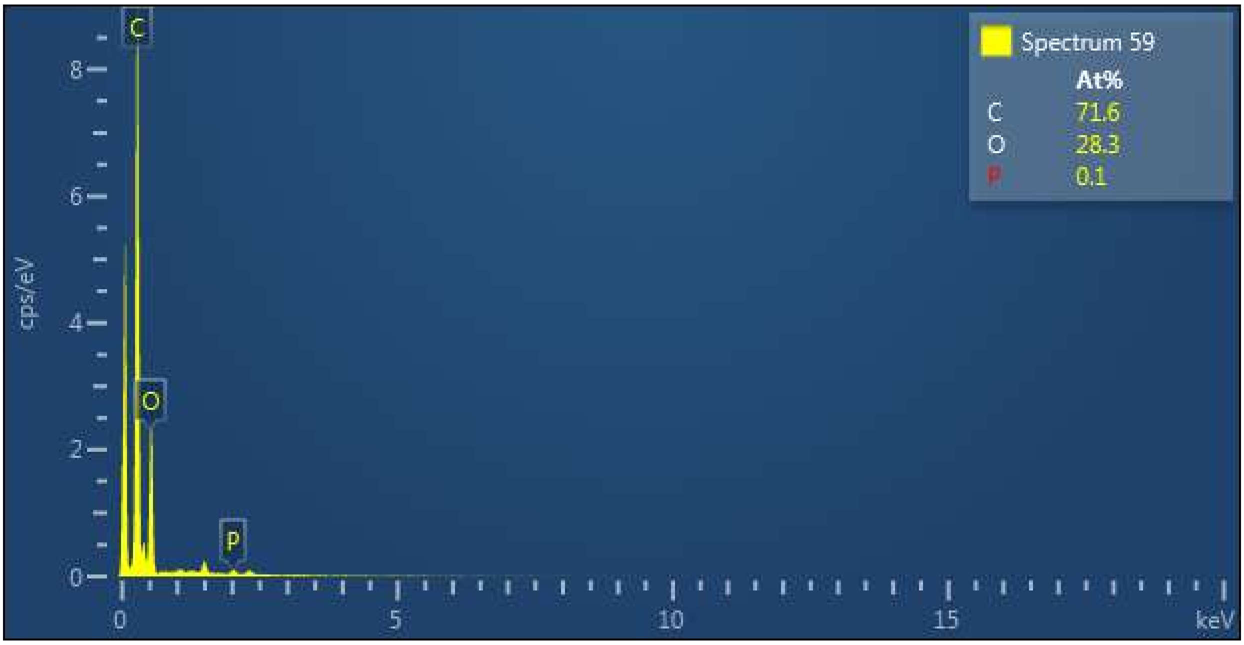

Label:	Spectrum 59
Source:	Acquired
Created:	23/01/2020 12:25:45
Livetime:	60.1s
Process Time:	4
Accelerating Voltage:	15.00kV
Magnification:	32 x
Working Distance:	10.0mm
Specimen Tilt (degrees):	0.0
Elevation (degrees):	35.0
Azimuth (degrees):	0.0
Number Of Channels:	2048
Energy Range (keV):	20 keV
Energy per Channel (eV):	10.0eV
Detector Type Id:	29
Detector Type:	X-Max
Window Type:	SATW
Pulse Pile Up Correction:	Succeeded
Primary Detector:	2617
Primary Detector Serial Number:	77871-X080

Element	Line Type	Apparent Concentration	k Ratio	Wt%	Wt% Sigma	Atomic %	Standard Label	Factory Standard	Standard Calibration Date
C	K series	13.69	0.13694	65.31	0.45	71.58	C Vit	Yes	
O	K series	6.92	0.02330	34.36	0.45	28.27	SiO2	Yes	
P	K	0.14	0.0008	0.34	0.05	0.14	GaP	Yes	

	serie s		1						
Total:				100.0 0		100.0 0			

Electron Image 20

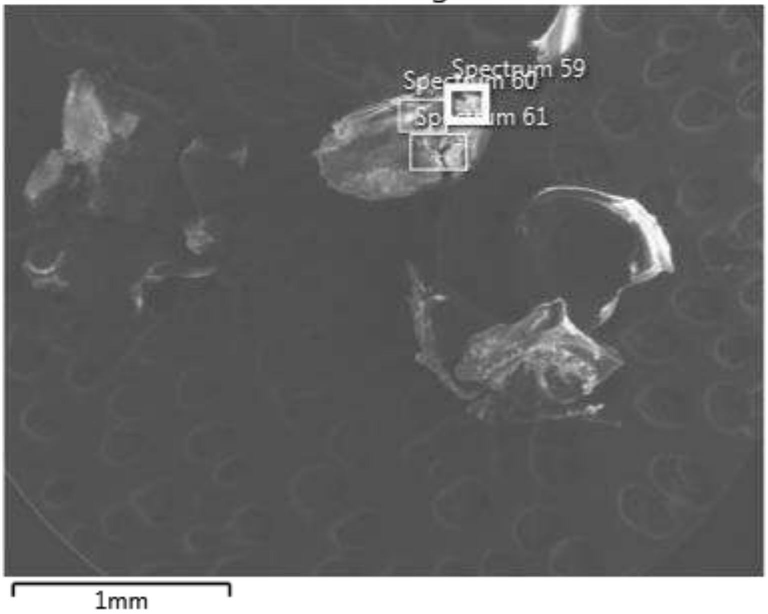

23/01/2020 12:26:53
Project 1
Rumania 5
Ventral 1 15 Kv

Electron Image 20

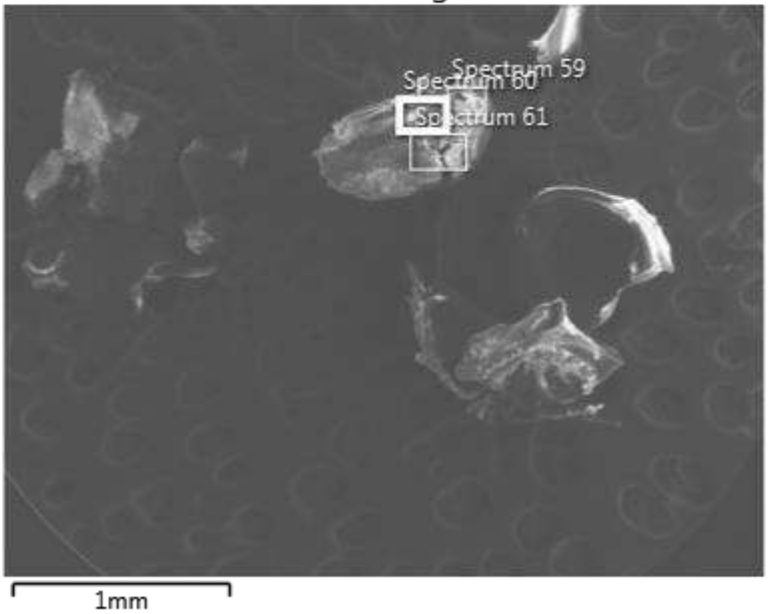

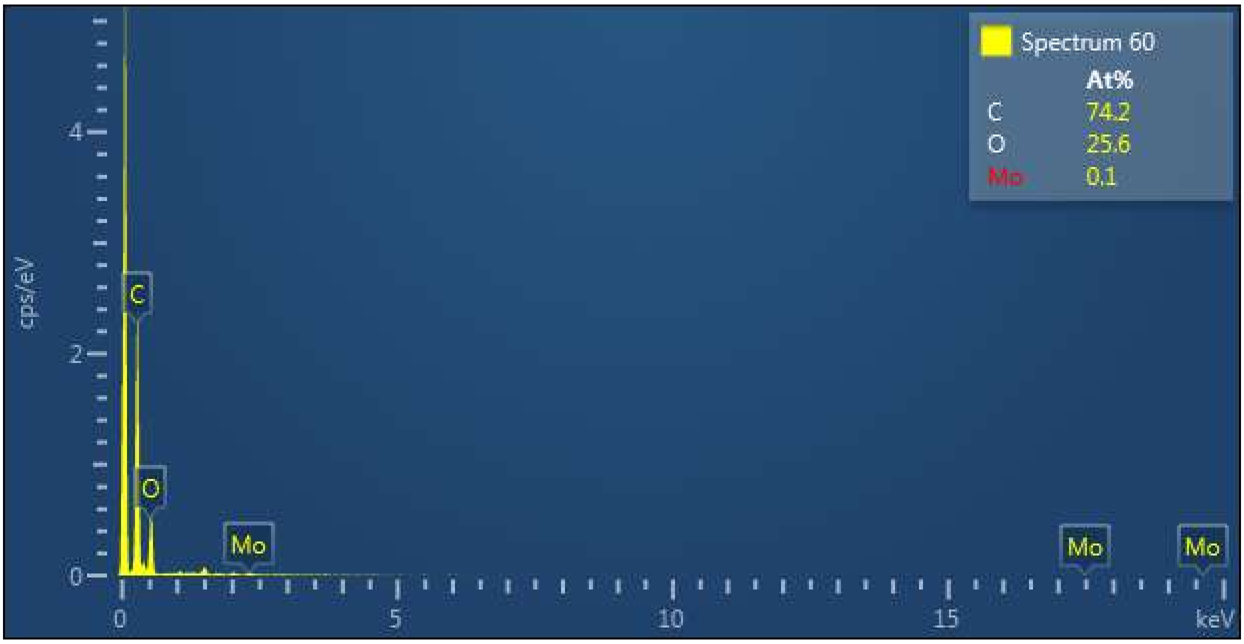

Label:	Spectrum 60
Source:	Acquired
Created:	23/01/2020 12:26:53
Livetime:	60.2s
Process Time:	4
Accelerating Voltage:	15.00kV
Magnification:	32 x
Working Distance:	10.0mm
Specimen Tilt (degrees):	0.0
Elevation (degrees):	35.0
Azimuth (degrees):	0.0
Number Of Channels:	2048
Energy Range (keV):	20 keV
Energy per Channel (eV):	10.0eV
Detector Type Id:	29
Detector Type:	X-Max
Window Type:	SATW
Pulse Pile Up Correction:	Succeeded
Primary Detector:	2617
Primary Detector Serial Number:	77871-X080

Element	Line Type	Apparent Concentration	k Ratio	Wt%	Wt% Sigma	Atomic %	Standard Label	Factory Standard	Standard Calibration Date
C	K series	3.75	0.03745	67.82	0.94	74.25	C Vit	Yes	
O	K series	1.56	0.00526	31.16	0.92	25.61	SiO2	Yes	
Mo	L	0.06	0.0006	1.02	0.29	0.14	Mo	Yes	

	serie s		0						
Total:				100.00		100.00			

Electron Image 20

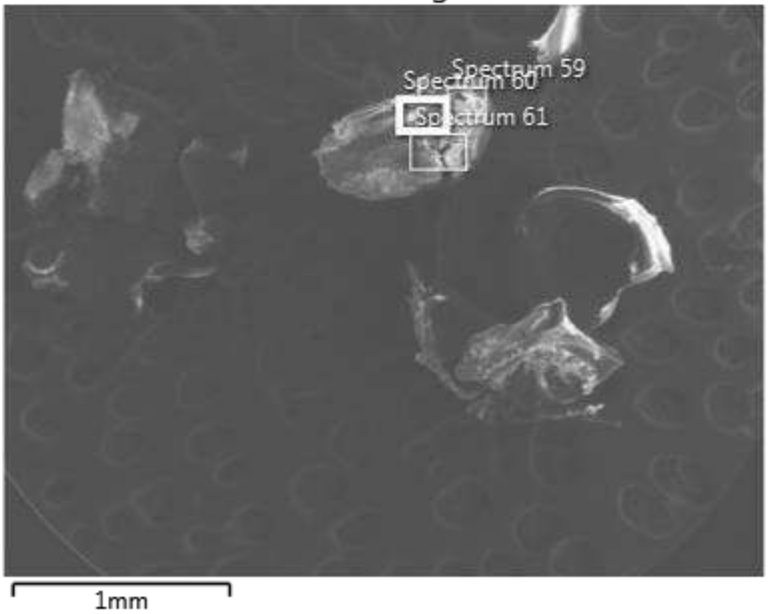

23/01/2020 12:28:00
Project 1
Rumania 5
Ventral 1 15 Kv

Electron Image 20

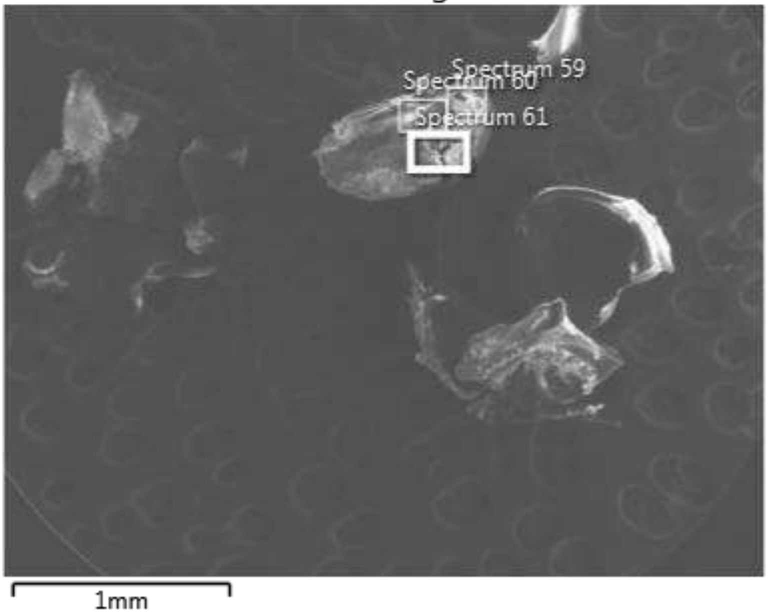

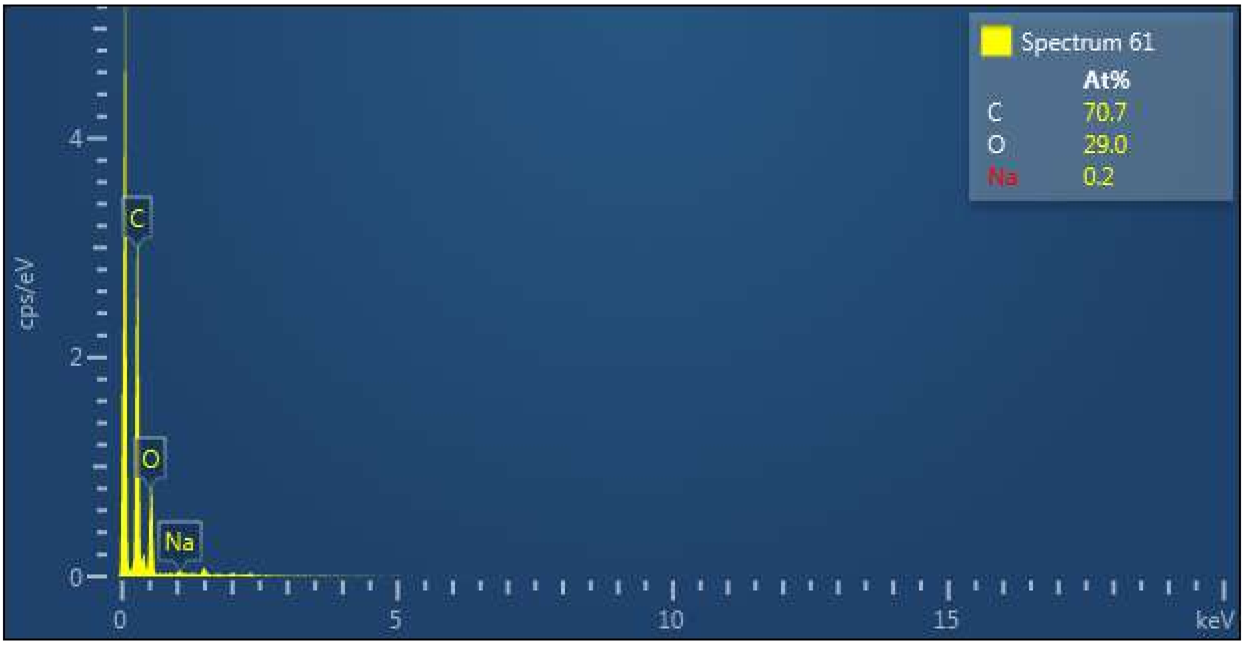

Label:	Spectrum 61
Source:	Acquired
Created:	23/01/2020 12:28:00
Livetime:	60.0s
Process Time:	4
Accelerating Voltage:	15.00kV
Magnification:	32 x
Working Distance:	10.0mm
Specimen Tilt (degrees):	0.0
Elevation (degrees):	35.0
Azimuth (degrees):	0.0
Number Of Channels:	2048
Energy Range (keV):	20 keV
Energy per Channel (eV):	10.0eV
Detector Type Id:	29
Detector Type:	X-Max
Window Type:	SATW
Pulse Pile Up Correction:	Succeeded
Primary Detector:	2617
Primary Detector Serial Number:	77871-X080

Element	Line Type	Apparent Concentration	k Ratio	Wt%	Wt% Sigma	Atomic %	Standard Label	Factory Standard	Standard Calibration Date
C	K series	4.72	0.04724	64.37	0.76	70.72	C Vit	Yes	
O	K series	2.50	0.00841	35.21	0.76	29.04	SiO2	Yes	
Na	K	0.05	0.0002	0.42	0.12	0.24	Albite	Yes	

	serie s		1						
Total:				100.0 0		100.0 0			

Electron Image 20

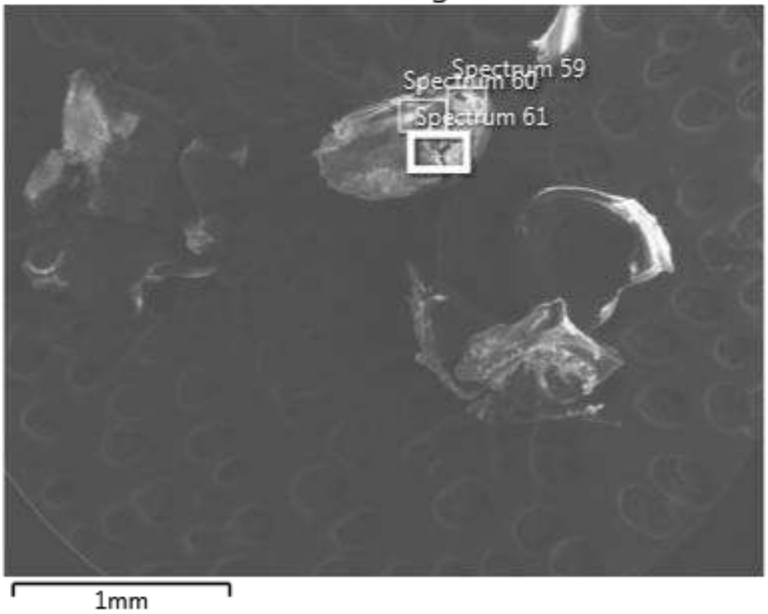

23/01/2020 12:30:19
Project 1
Rumania 6
Pecho 1 15 Kv

Electron Image 21

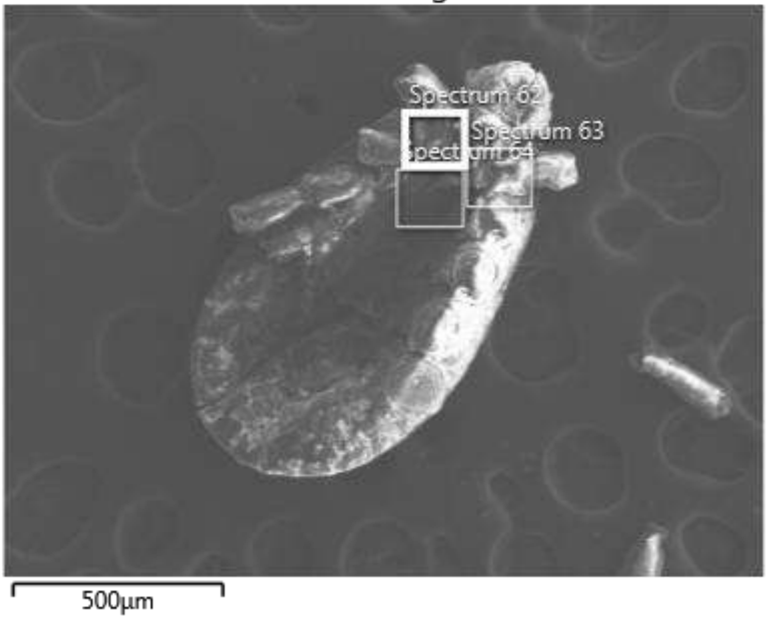

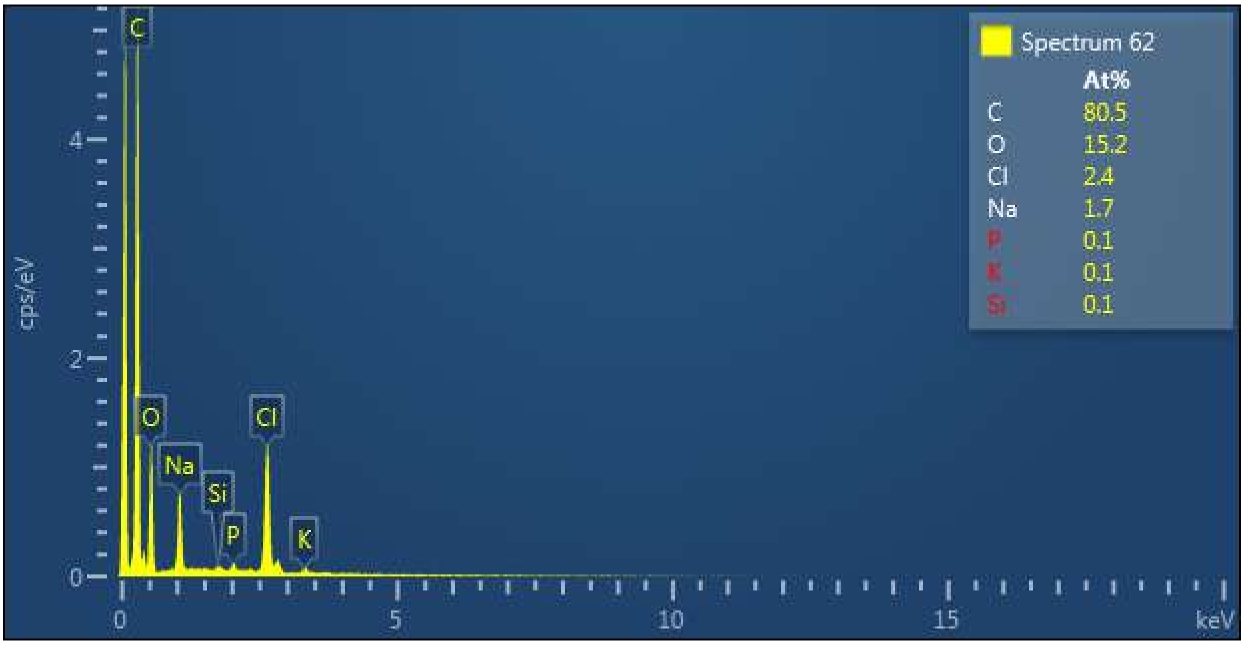

Label:	Spectrum 62
Source:	Acquired
Created:	23/01/2020 12:30:19
Livetime:	60.3s
Process Time:	4
Accelerating Voltage:	15.00kV
Magnification:	62 x
Working Distance:	10.0mm
Specimen Tilt (degrees):	0.0
Elevation (degrees):	35.0
Azimuth (degrees):	0.0
Number Of Channels:	2048
Energy Range (keV):	20 keV
Energy per Channel (eV):	10.0eV
Detector Type Id:	29
Detector Type:	X-Max
Window Type:	SATW
Pulse Pile Up Correction:	Succeeded
Primary Detector:	2617
Primary Detector Serial Number:	77871-X080

Element	Line Type	Apparent Concentration	k Ratio	Wt%	Wt% Sigma	Atomic %	Standard Label	Factory Standard	Standard Calibration Date
C	K series	9.39	0.09387	71.99	0.50	80.46	C Vit	Yes	
O	K series	3.45	0.01162	18.06	0.44	15.15	SiO2	Yes	
Na	K	1.26	0.0053	2.94	0.10	1.72	Albite	Yes	

	series		3						
Si	K series	0.04	0.00032	0.12	0.04	0.06	SiO2	Yes	
P	K series	0.15	0.00081	0.29	0.05	0.13	GaP	Yes	
Cl	K series	2.07	0.01808	6.33	0.15	2.40	NaCl	Yes	
K	K series	0.09	0.00079	0.28	0.06	0.10	KBr	Yes	
Total:				100.00		100.00			

Electron Image 21

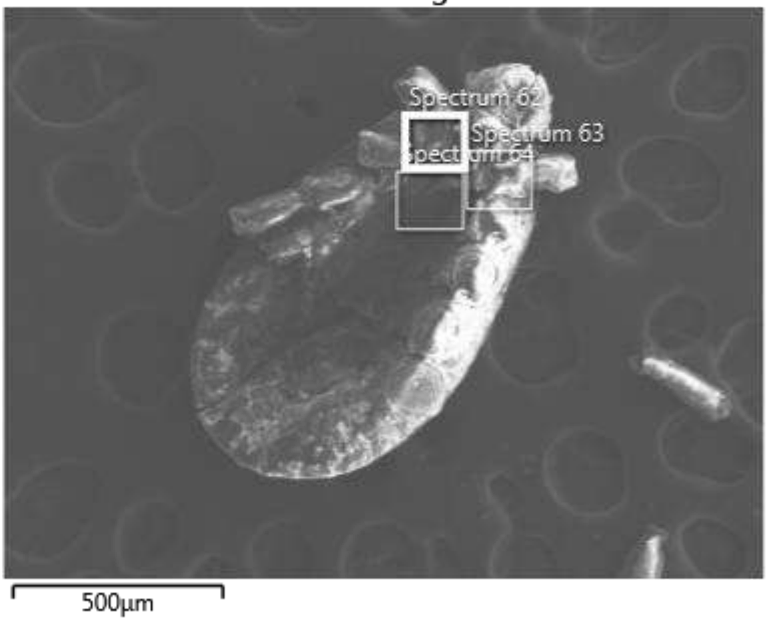

23/01/2020 12:31:30
Project 1
Rumania 6
Pecho 1 15 Kv

Electron Image 21

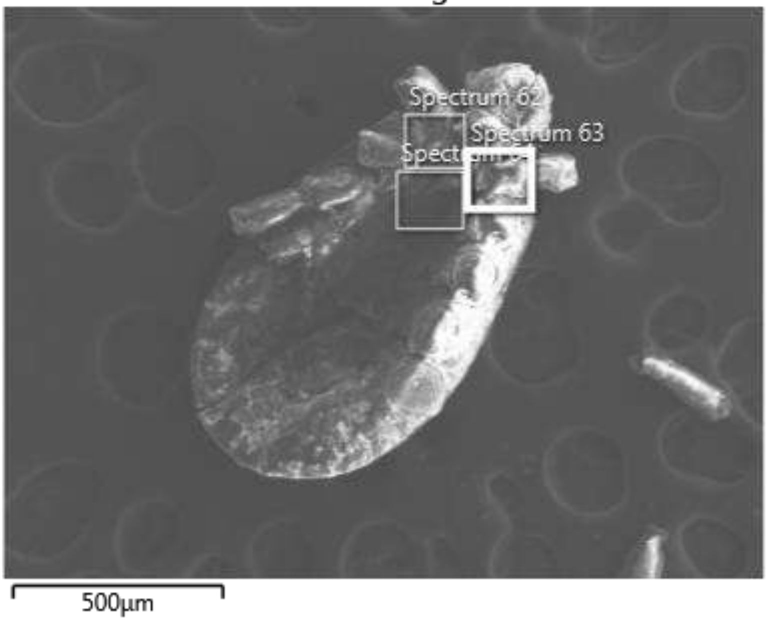

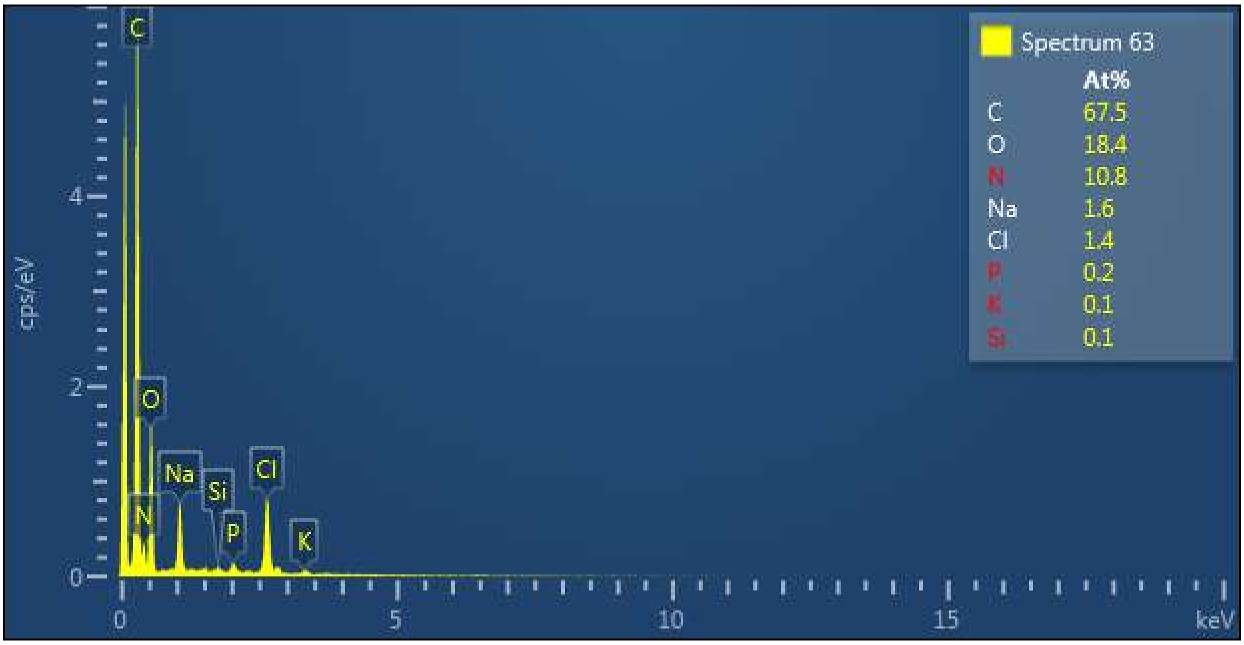

Label:	Spectrum 63
Source:	Acquired
Created:	23/01/2020 12:31:30
Livetime:	60.0s
Process Time:	4
Accelerating Voltage:	15.00kV
Magnification:	62 x
Working Distance:	10.0mm
Specimen Tilt (degrees):	0.0
Elevation (degrees):	35.0
Azimuth (degrees):	0.0
Number Of Channels:	2048
Energy Range (keV):	20 keV
Energy per Channel (eV):	10.0eV
Detector Type Id:	29
Detector Type:	X-Max
Window Type:	SATW
Pulse Pile Up Correction:	Succeeded
Primary Detector:	2617
Primary Detector Serial Number:	77871-X080

Element	Line Type	Apparent Concentration	k Ratio	Wt%	Wt% Sigma	Atomic %	Standard Label	Factory Standard	Standard Calibration Date
C	K series	10.87	0.10865	59.94	0.95	67.49	C Vit	Yes	
N	K series	2.75	0.00490	11.21	1.19	10.82	BN	Yes	
O	K	4.70	0.0158	21.79	0.52	18.42	SiO2	Yes	

	series		1						
Na	K series	1.25	0.00528	2.67	0.10	1.57	Albite	Yes	
Si	K series	0.05	0.00040	0.13	0.03	0.06	SiO2	Yes	
P	K series	0.20	0.00112	0.35	0.04	0.15	GaP	Yes	
Cl	K series	1.36	0.01192	3.66	0.11	1.40	NaCl	Yes	
K	K series	0.10	0.00085	0.26	0.05	0.09	KBr	Yes	
Total:				100.00		100.00			

Electron Image 21

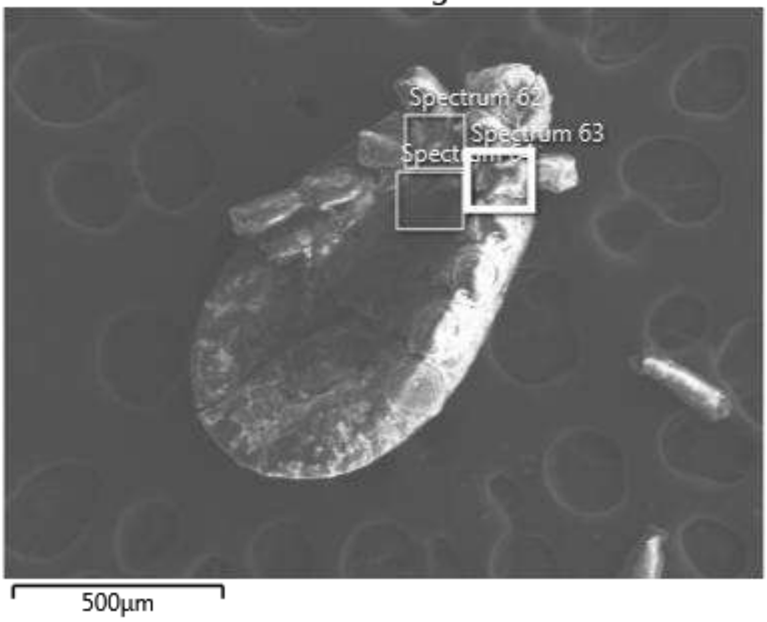

23/01/2020 12:32:42
Project 1
Rumania 6
Pecho 1 15 Kv

Electron Image 21

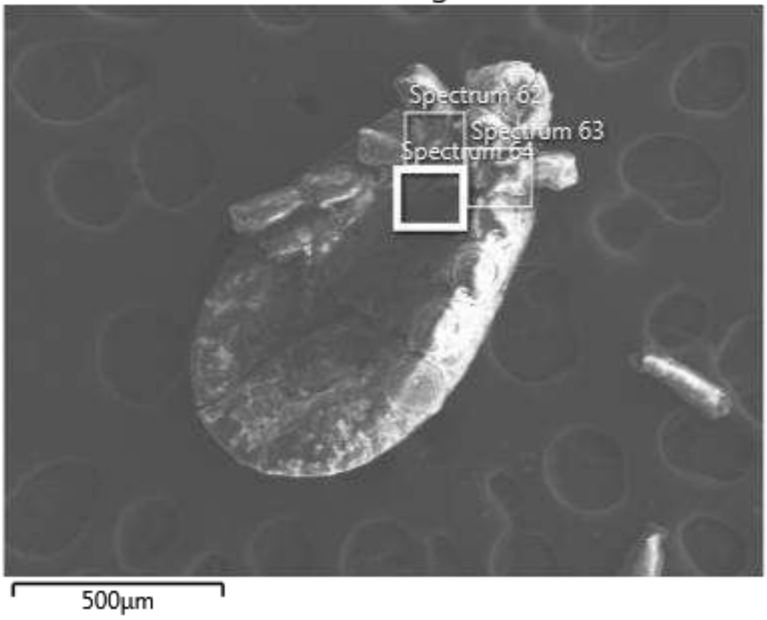

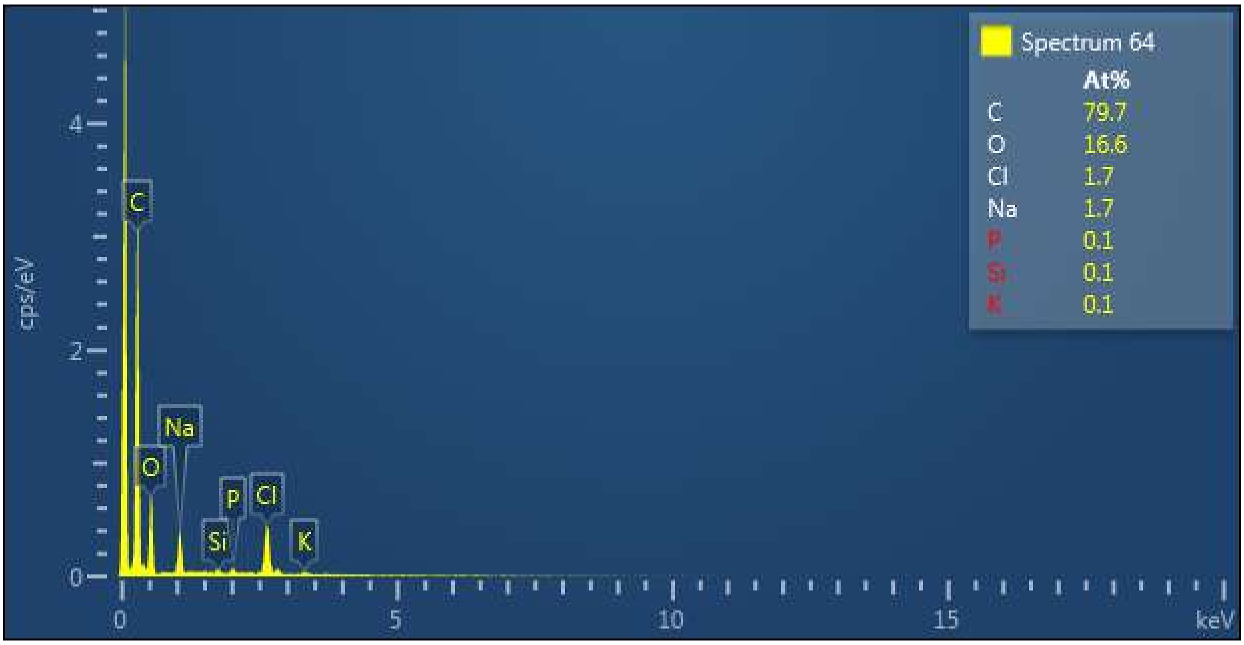

Label:	Spectrum 64
Source:	Acquired
Created:	23/01/2020 12:32:42
Livetime:	60.2s
Process Time:	4
Accelerating Voltage:	15.00kV
Magnification:	62 x
Working Distance:	10.0mm
Specimen Tilt (degrees):	0.0
Elevation (degrees):	35.0
Azimuth (degrees):	0.0
Number Of Channels:	2048
Energy Range (keV):	20 keV
Energy per Channel (eV):	10.0eV
Detector Type Id:	29
Detector Type:	X-Max
Window Type:	SATW
Pulse Pile Up Correction:	Succeeded
Primary Detector:	2617
Primary Detector Serial Number:	77871-X080

Element	Line Type	Apparent Concentration	k Ratio	Wt%	Wt% Sigma	Atomic %	Standard Label	Factory Standard	Standard Calibration Date
C	K series	5.58	0.05584	71.80	0.69	79.68	C Vit	Yes	
O	K series	2.03	0.00682	19.90	0.62	16.58	SiO2	Yes	
Na	K	0.64	0.0026	2.89	0.14	1.68	Albite	Yes	

	series		9						
Si	K series	0.05	0.00037	0.26	0.06	0.12	SiO2	Yes	
P	K series	0.09	0.00049	0.33	0.07	0.14	GaP	Yes	
Cl	K series	0.77	0.00673	4.55	0.17	1.71	NaCl	Yes	
K	K series	0.05	0.00039	0.26	0.08	0.09	KBr	Yes	
Total:				100.00		100.00			

Electron Image 21

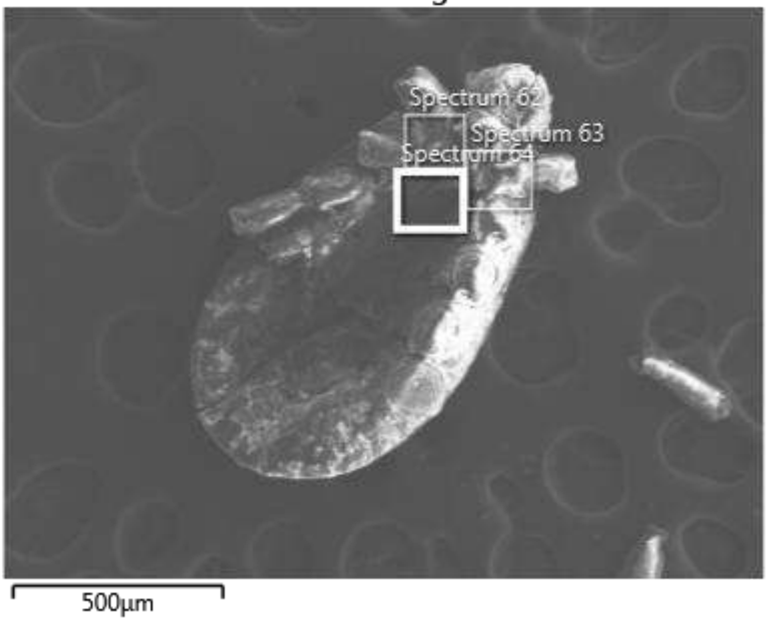

23/01/2020 12:34:56
Project 1
Rumania 6
Ventral 1 15 Kv

Electron Image 22

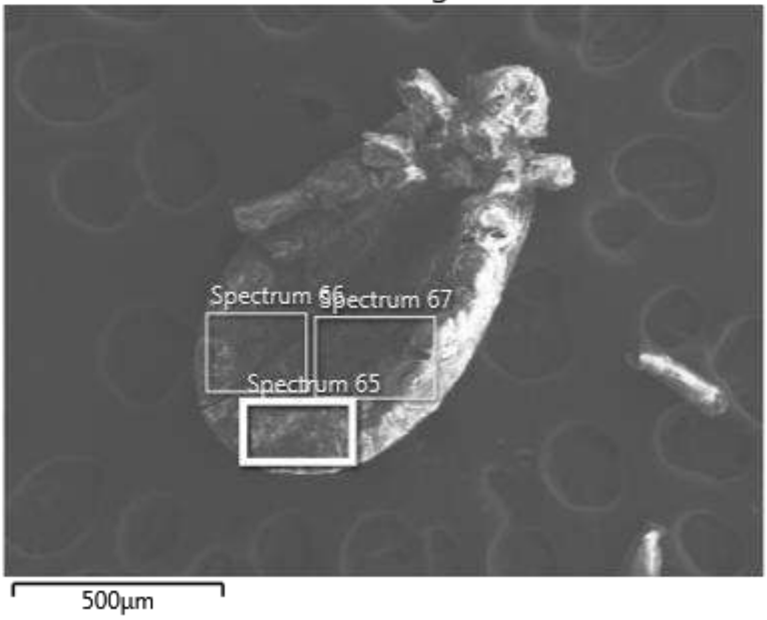

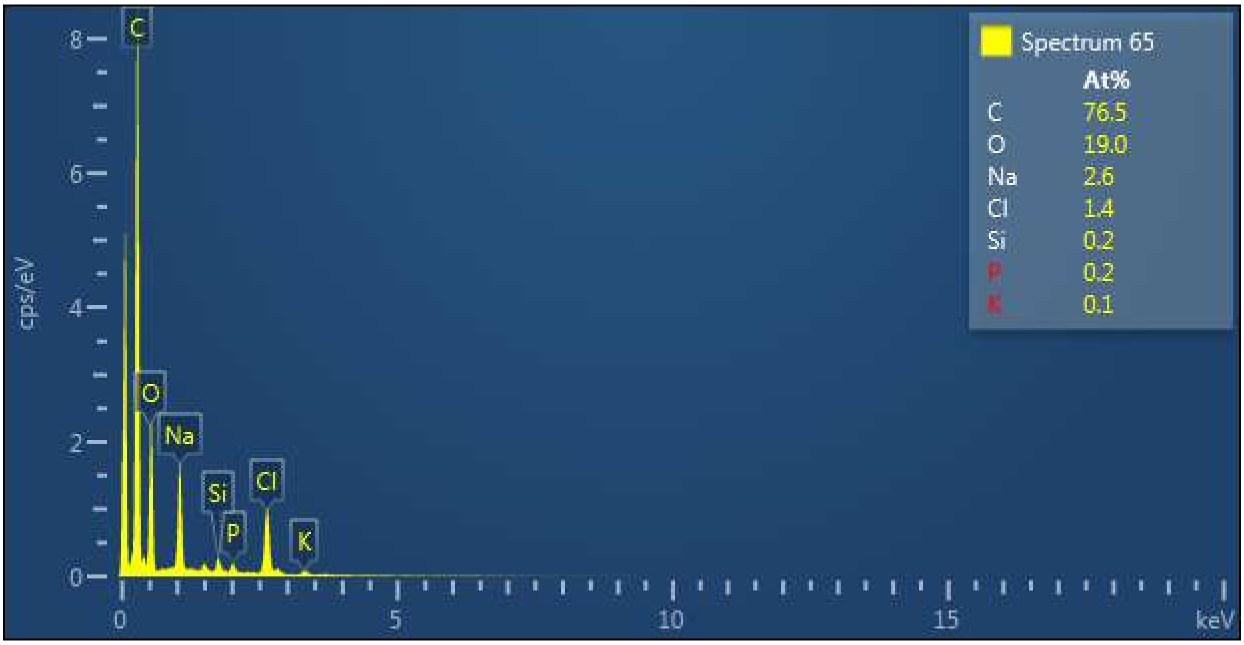

Label:	Spectrum 65
Source:	Acquired
Created:	23/01/2020 12:34:56
Livetime:	60.1s
Process Time:	4
Accelerating Voltage:	15.00kV
Magnification:	62 x
Working Distance:	10.0mm
Specimen Tilt (degrees):	0.0
Elevation (degrees):	35.0
Azimuth (degrees):	0.0
Number Of Channels:	2048
Energy Range (keV):	20 keV
Energy per Channel (eV):	10.0eV
Detector Type Id:	29
Detector Type:	X-Max
Window Type:	SATW
Pulse Pile Up Correction:	Succeeded
Primary Detector:	2617
Primary Detector Serial Number:	77871-X080

Element	Line Type	Apparent Concentration	k Ratio	Wt%	Wt% Sigma	Atomic %	Standard Label	Factory Standard	Standard Calibration Date
C	K series	14.75	0.14746	68.15	0.43	76.49	C Vit	Yes	
O	K series	6.69	0.02251	22.53	0.38	18.99	SiO2	Yes	
Na	K	2.69	0.0113	4.45	0.10	2.61	Albite	Yes	

	series		6						
Si	K series	0.25	0.00200	0.51	0.04	0.25	SiO2	Yes	
P	K series	0.26	0.00146	0.37	0.04	0.16	GaP	Yes	
Cl	K series	1.72	0.01499	3.70	0.09	1.41	NaCl	Yes	
K	K series	0.14	0.00117	0.29	0.04	0.10	KBr	Yes	
Total:				100.00		100.00			

Electron Image 22

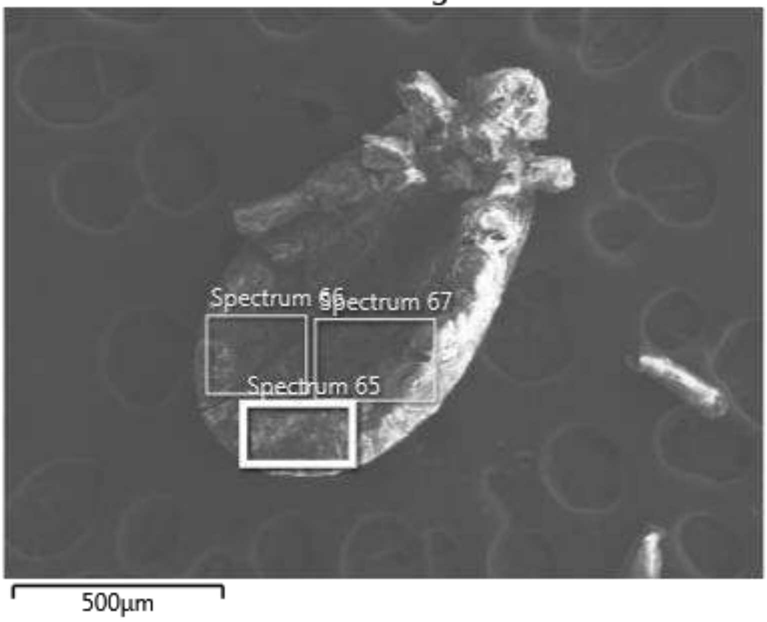

23/01/2020 12:36:08
Project 1
Rumania 6
Ventral 1 15 Kv

Electron Image 22

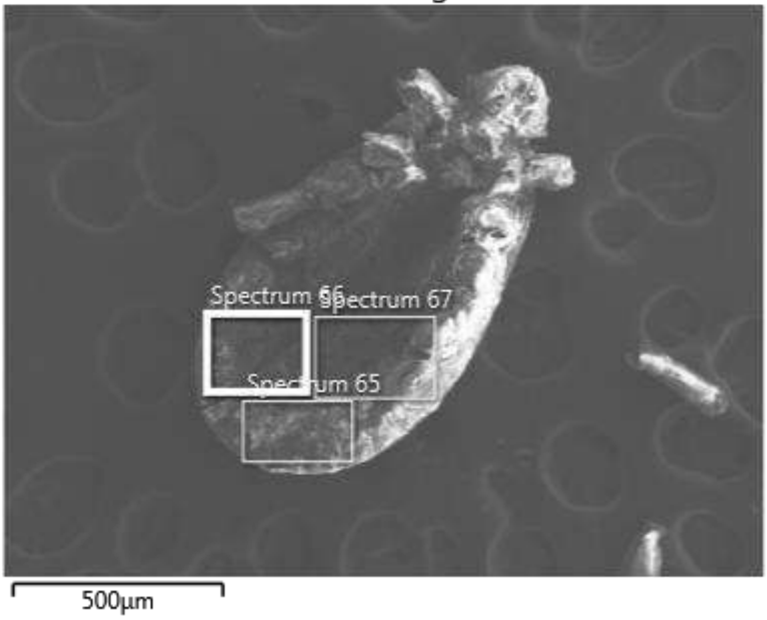

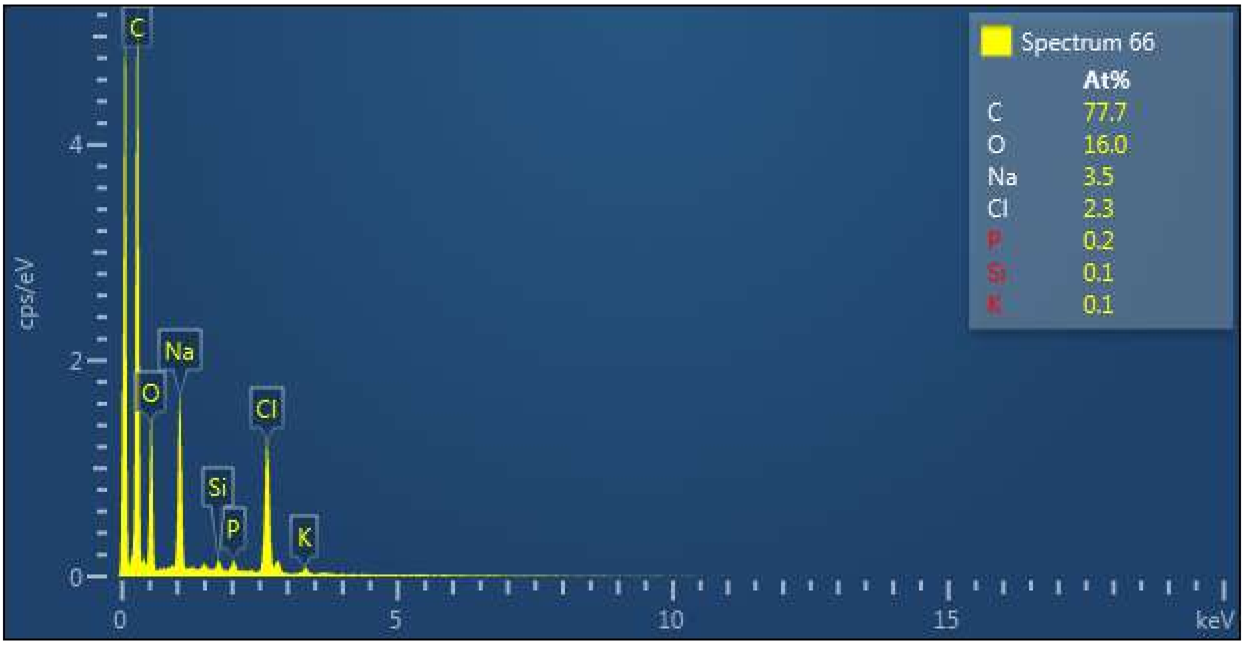

Label:	Spectrum 66
Source:	Acquired
Created:	23/01/2020 12:36:08
Livetime:	60.3s
Process Time:	4
Accelerating Voltage:	15.00kV
Magnification:	62 x
Working Distance:	10.0mm
Specimen Tilt (degrees):	0.0
Elevation (degrees):	35.0
Azimuth (degrees):	0.0
Number Of Channels:	2048
Energy Range (keV):	20 keV
Energy per Channel (eV):	10.0eV
Detector Type Id:	29
Detector Type:	X-Max
Window Type:	SATW
Pulse Pile Up Correction:	Succeeded
Primary Detector:	2617
Primary Detector Serial Number:	77871-X080

Element	Line Type	Apparent Concentration	k Ratio	Wt%	Wt% Sigma	Atomic %	Standard Label	Factory Standard	Standard Calibration Date
C	K series	9.30	0.09301	68.32	0.50	77.73	C Vit	Yes	
O	K series	4.13	0.01390	18.78	0.41	16.04	SiO2	Yes	
Na	K	2.80	0.0118	5.87	0.14	3.49	Albite	Yes	

	series		1						
Si	K series	0.11	0.00090	0.30	0.04	0.15	SiO2	Yes	
P	K series	0.20	0.00114	0.37	0.04	0.16	GaP	Yes	
Cl	K series	2.14	0.01874	5.99	0.14	2.31	NaCl	Yes	
K	K series	0.13	0.00114	0.37	0.05	0.13	KBr	Yes	
Total:				100.00		100.00			

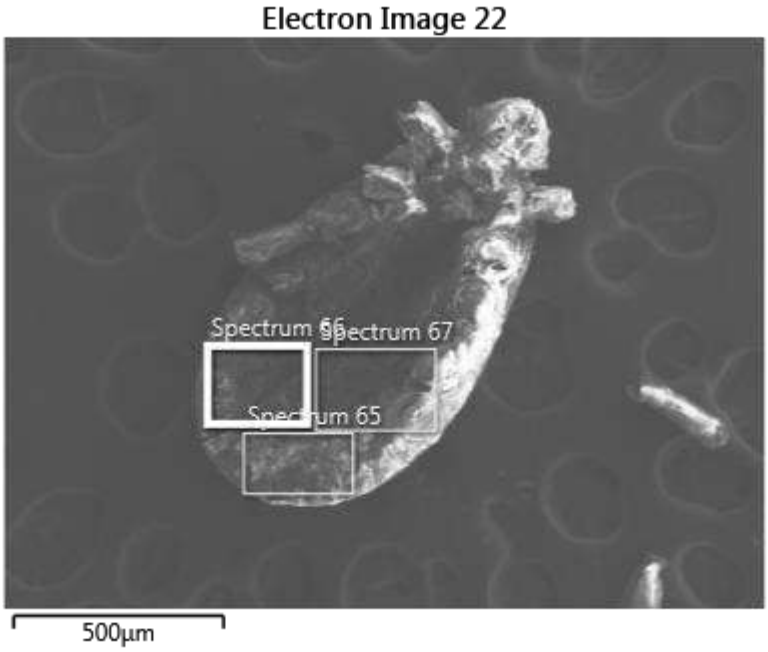

23/01/2020 12:37:20
Project 1
Rumania 6
Ventral 1 15 Kv

Electron Image 22

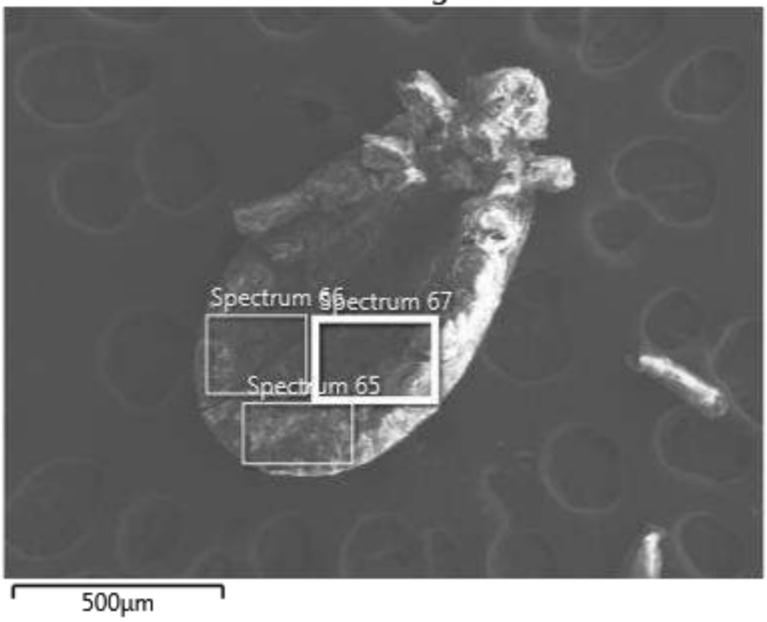

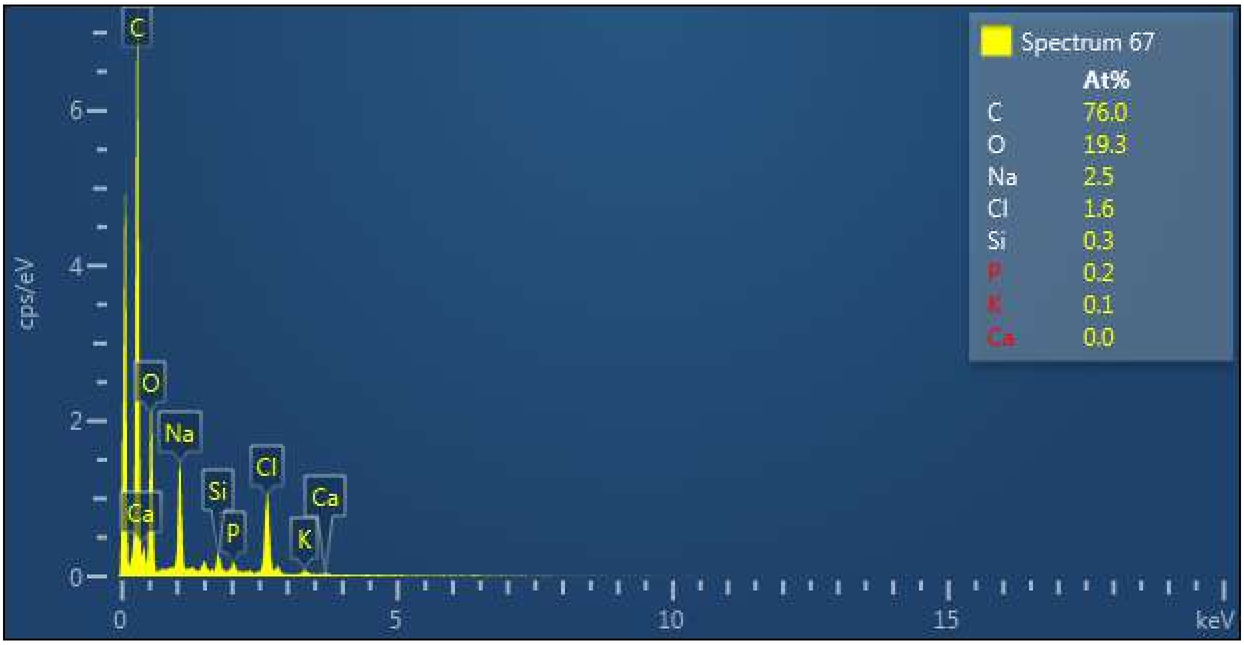

Label:	Spectrum 67
Source:	Acquired
Created:	23/01/2020 12:37:20
Livetime:	60.3s
Process Time:	4
Accelerating Voltage:	15.00kV
Magnification:	62 x
Working Distance:	10.0mm
Specimen Tilt (degrees):	0.0
Elevation (degrees):	35.0
Azimuth (degrees):	0.0
Number Of Channels:	2048
Energy Range (keV):	20 keV
Energy per Channel (eV):	10.0eV
Detector Type Id:	29
Detector Type:	X-Max
Window Type:	SATW
Pulse Pile Up Correction:	Succeeded
Primary Detector:	2617
Primary Detector Serial Number:	77871-X080

Element	Line Type	Apparent Concentration	k Ratio	Wt%	Wt% Sigma	Atomic %	Standard Label	Factory Standard	Standard Calibration Date
C	K series	13.18	0.13185	67.39	0.46	75.99	C Vit	Yes	
O	K series	6.41	0.02157	22.81	0.41	19.31	SiO2	Yes	
Na	K	2.43	0.0102	4.27	0.10	2.51	Albite	Yes	

	serie s		7						
Si	K serie s	0.28	0.0022 2	0.60	0.04	0.29	SiO2	Yes	
P	K serie s	0.24	0.0013 7	0.37	0.04	0.16	GaP	Yes	
Cl	K serie s	1.82	0.0159 4	4.15	0.10	1.59	NaCl	Yes	
K	K serie s	0.12	0.0010 4	0.27	0.04	0.09	KBr	Yes	
Ca	K serie s	0.06	0.0005 5	0.14	0.04	0.05	Wollastoni te	Yes	
Total:				100.0 0		100.0 0			

Electron Image 22

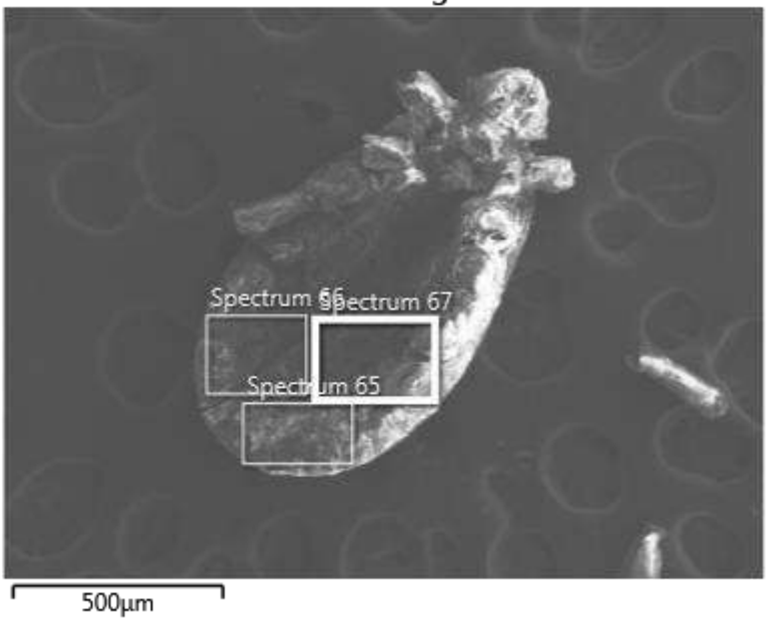

23/01/2020 12:40:11
Project 1
Rumania 7
Pecho 1 15 Kv

Electron Image 23

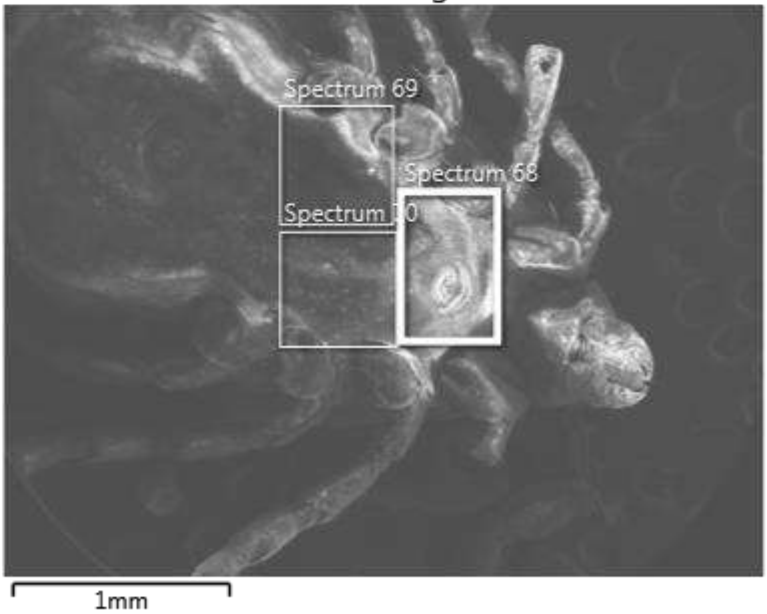

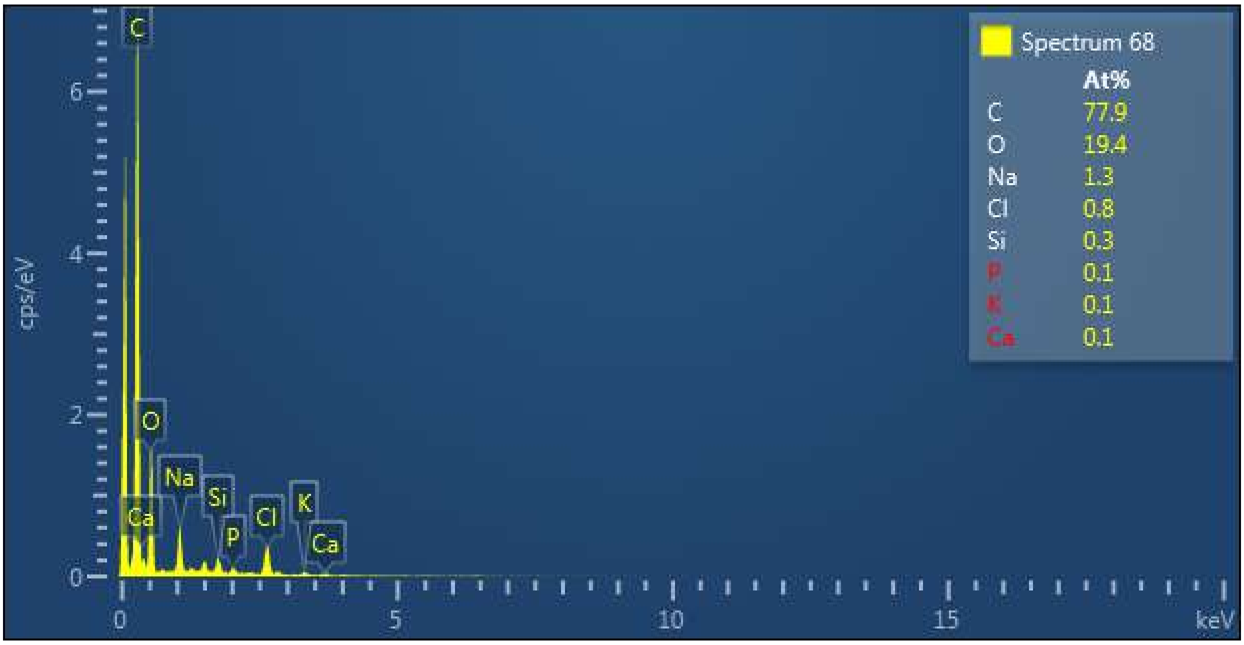

Label:	Spectrum 68
Source:	Acquired
Created:	23/01/2020 12:40:11
Livetime:	60.4s
Process Time:	4
Accelerating Voltage:	15.00kV
Magnification:	32 x
Working Distance:	10.0mm
Specimen Tilt (degrees):	0.0
Elevation (degrees):	35.0
Azimuth (degrees):	0.0
Number Of Channels:	2048
Energy Range (keV):	20 keV
Energy per Channel (eV):	10.0eV
Detector Type Id:	29
Detector Type:	X-Max
Window Type:	SATW
Pulse Pile Up Correction:	Succeeded
Primary Detector:	2617
Primary Detector Serial Number:	77871-X080

Element	Line Type	Apparent Concentration	k Ratio	Wt%	Wt% Sigma	Atomic %	Standard Label	Factory Standard	Standard Calibration Date
C	K series	12.78	0.12778	70.72	0.50	77.90	C Vit	Yes	
O	K series	4.66	0.01568	23.43	0.47	19.38	SiO2	Yes	
Na	K	0.94	0.0039	2.32	0.09	1.33	Albite	Yes	

	series		6						
Si	K series	0.22	0.00177	0.66	0.05	0.31	SiO2	Yes	
P	K series	0.16	0.00087	0.32	0.04	0.14	GaP	Yes	
Cl	K series	0.65	0.00567	2.06	0.08	0.77	NaCl	Yes	
K	K series	0.09	0.00076	0.27	0.05	0.09	KBr	Yes	
Ca	K series	0.07	0.00060	0.21	0.05	0.07	Wollastonite	Yes	
Total:				100.00		100.00			

Electron Image 23

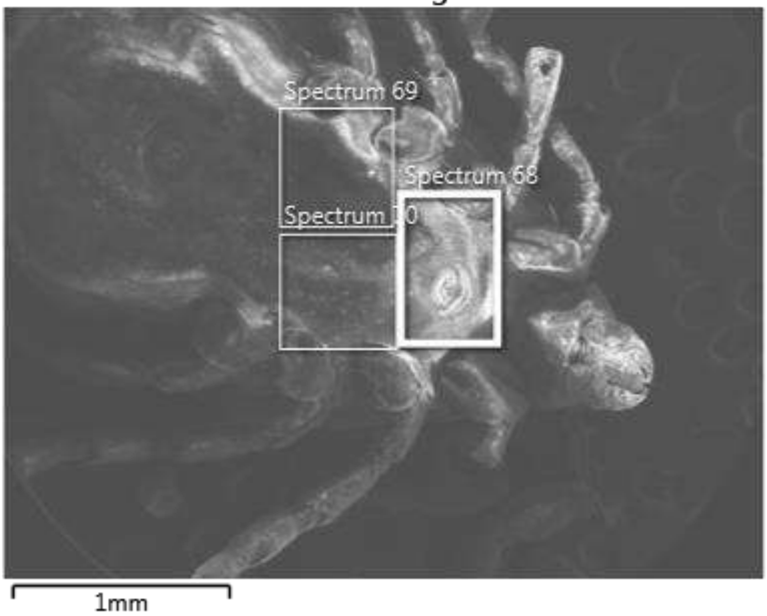

23/01/2020 12:41:22
Project 1
Rumania 7
Pecho 1 15 Kv

Electron Image 23

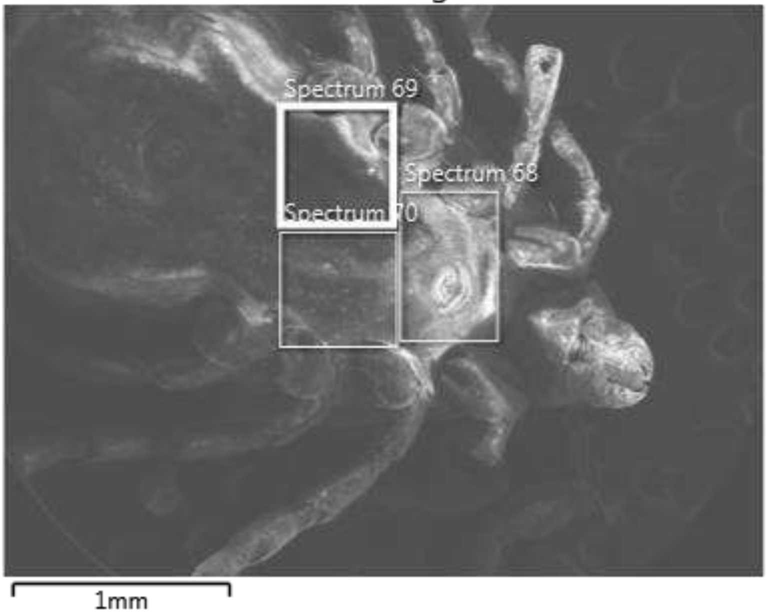

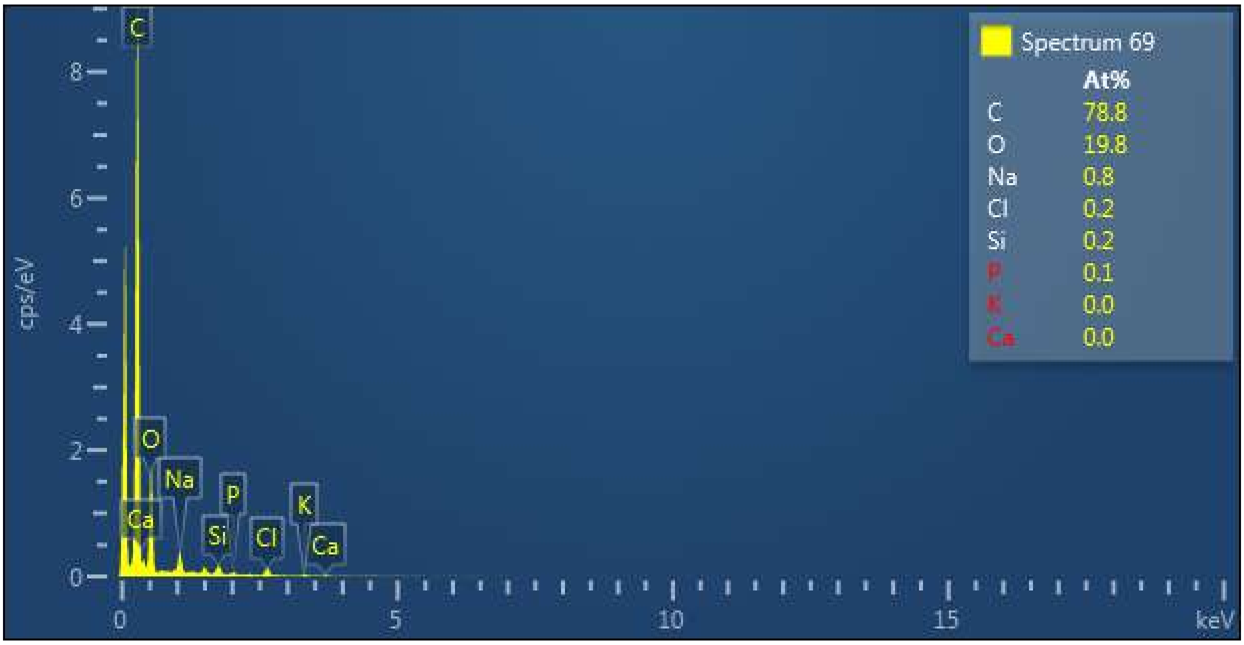

Label:	Spectrum 69
Source:	Acquired
Created:	23/01/2020 12:41:22
Livetime:	60.3s
Process Time:	4
Accelerating Voltage:	15.00kV
Magnification:	32 x
Working Distance:	10.0mm
Specimen Tilt (degrees):	0.0
Elevation (degrees):	35.0
Azimuth (degrees):	0.0
Number Of Channels:	2048
Energy Range (keV):	20 keV
Energy per Channel (eV):	10.0eV
Detector Type Id:	29
Detector Type:	X-Max
Window Type:	SATW
Pulse Pile Up Correction:	Succeeded
Primary Detector:	2617
Primary Detector Serial Number:	77871-X080

Element	Line Type	Apparent Concentration	k Ratio	Wt%	Wt% Sigma	Atomic %	Standard Label	Factory Standard	Standard Calibration Date
C	K series	16.31	0.16312	72.71	0.48	78.79	C Vit	Yes	
O	K series	4.92	0.01656	24.38	0.46	19.83	SiO2	Yes	
Na	K	0.54	0.0022	1.33	0.08	0.75	Albite	Yes	

	series		8						
Si	K series	0.18	0.00140	0.52	0.04	0.24	SiO2	Yes	
P	K series	0.08	0.00043	0.16	0.04	0.07	GaP	Yes	
Cl	K series	0.21	0.00186	0.67	0.05	0.24	NaCl	Yes	
K	K series	0.04	0.00034	0.12	0.04	0.04	KBr	Yes	
Ca	K series	0.04	0.00032	0.11	0.04	0.04	Wollastonite	Yes	
Total:				100.00		100.00			

Electron Image 23

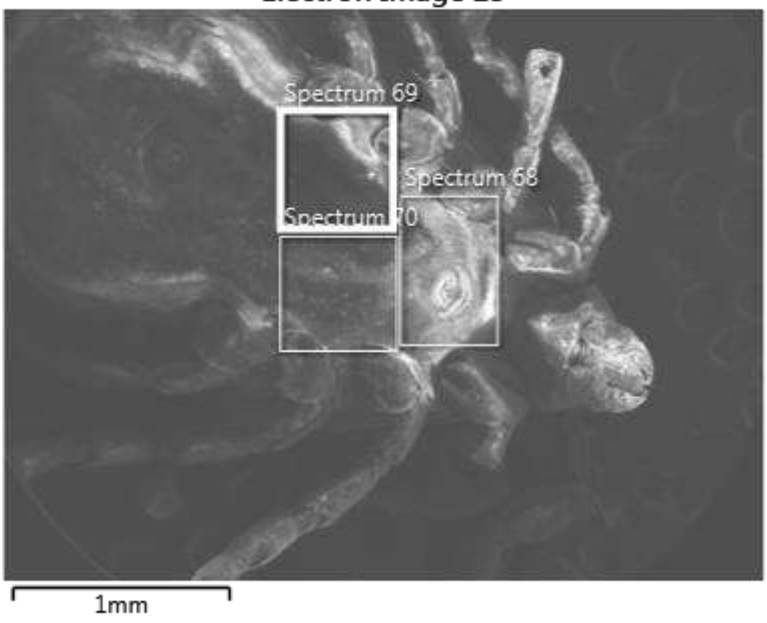

23/01/2020 12:42:33
Project 1
Rumania 7
Pecho 1 15 Kv

Electron Image 23

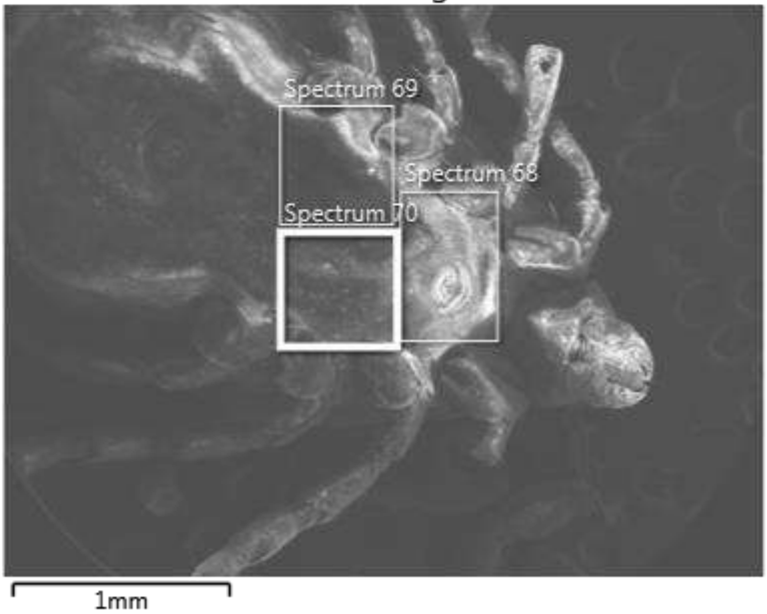

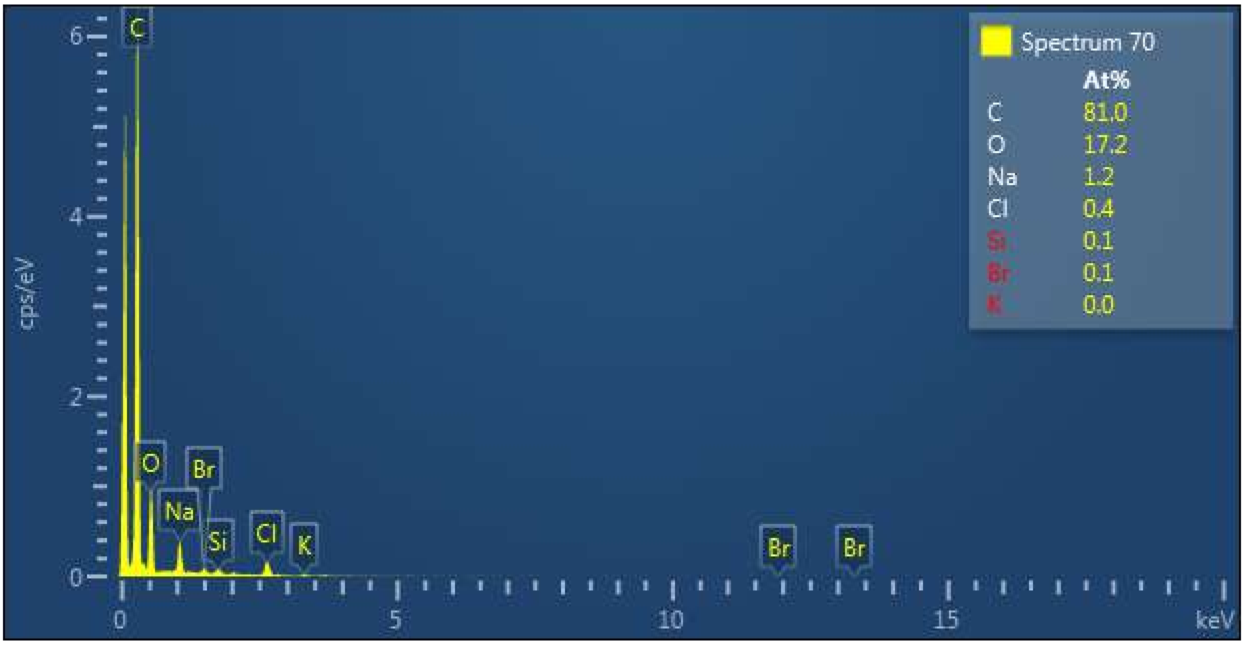

Label:	Spectrum 70
Source:	Acquired
Created:	23/01/2020 12:42:33
Livetime:	60.3s
Process Time:	4
Accelerating Voltage:	15.00kV
Magnification:	32 x
Working Distance:	10.0mm
Specimen Tilt (degrees):	0.0
Elevation (degrees):	35.0
Azimuth (degrees):	0.0
Number Of Channels:	2048
Energy Range (keV):	20 keV
Energy per Channel (eV):	10.0eV
Detector Type Id:	29
Detector Type:	X-Max
Window Type:	SATW
Pulse Pile Up Correction:	Succeeded
Primary Detector:	2617
Primary Detector Serial Number:	77871-X080

Element	Line Type	Apparent Concentration	k Ratio	Wt%	Wt% Sigma	Atomic %	Standard Label	Factory Standard	Standard Calibration Date
C	K series	11.29	0.11288	74.72	0.54	80.96	C Vit	Yes	
O	K series	2.93	0.00986	21.15	0.52	17.20	SiO2	Yes	
Na	K	0.61	0.0025	2.06	0.10	1.17	Albite	Yes	

	series		6						
Si	K series	0.06	0.00049	0.26	0.05	0.12	SiO2	Yes	
Cl	K series	0.26	0.00226	1.14	0.07	0.42	NaCl	Yes	
K	K series	0.03	0.00027	0.14	0.04	0.05	KBr	Yes	
Br	L series	0.10	0.00091	0.54	0.10	0.09	KBr	Yes	
Total:				100.00		100.00			

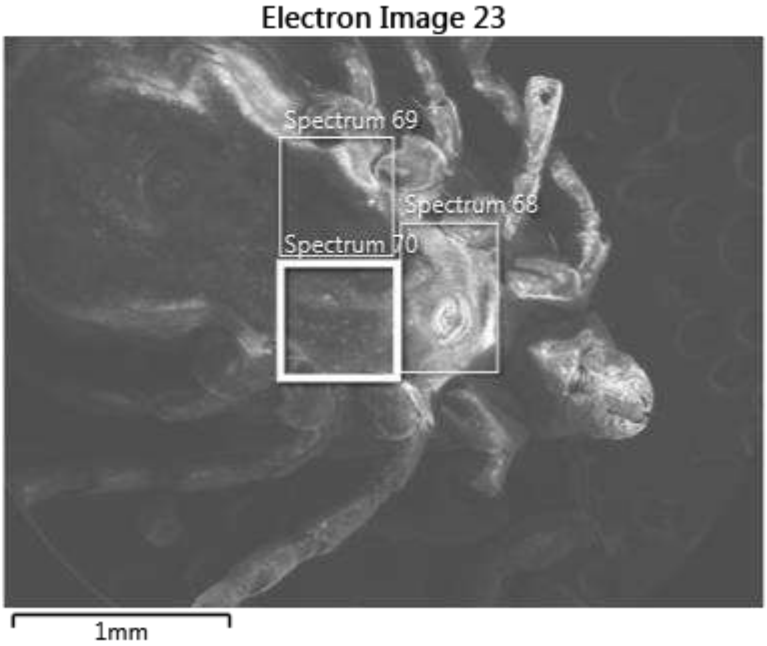

23/01/2020 12:44:26
Project 1
Rumania 7
Ventral 1 15 Kv

Electron Image 24

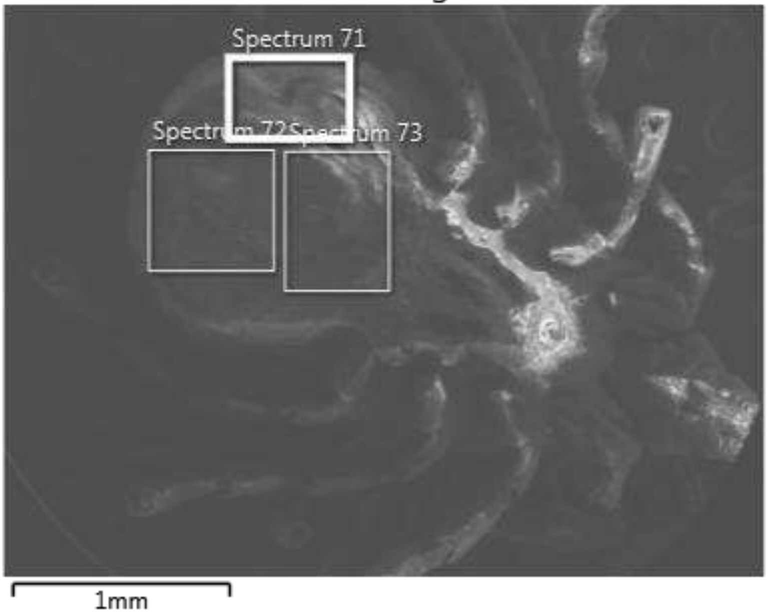

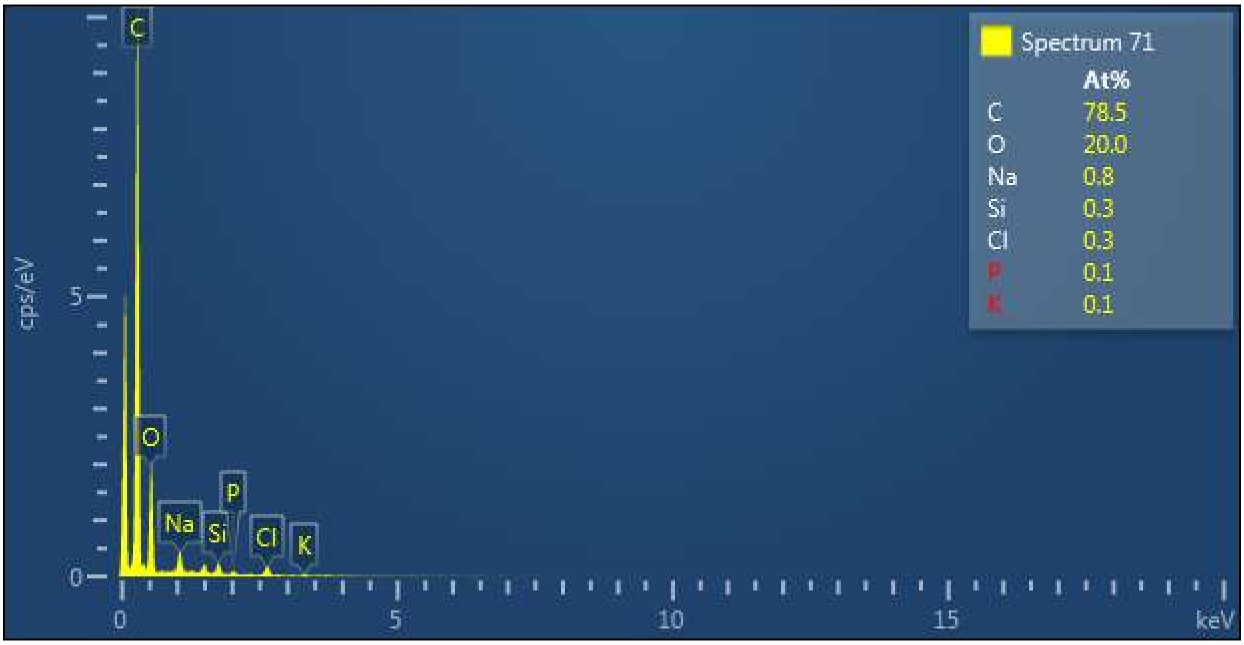

Label:	Spectrum 71
Source:	Acquired
Created:	23/01/2020 12:44:26
Livetime:	60.4s
Process Time:	4
Accelerating Voltage:	15.00kV
Magnification:	32 x
Working Distance:	10.0mm
Specimen Tilt (degrees):	0.0
Elevation (degrees):	35.0
Azimuth (degrees):	0.0
Number Of Channels:	2048
Energy Range (keV):	20 keV
Energy per Channel (eV):	10.0eV
Detector Type Id:	29
Detector Type:	X-Max
Window Type:	SATW
Pulse Pile Up Correction:	Succeeded
Primary Detector:	2617
Primary Detector Serial Number:	77871-X080

Element	Line Type	Apparent Concentration	k Ratio	Wt%	Wt% Sigma	Atomic %	Standard Label	Factory Standard	Standard Calibration Date
C	K series	18.15	0.18146	72.29	0.43	78.48	C Vit	Yes	
O	K series	5.71	0.01922	24.57	0.42	20.02	SiO2	Yes	
Na	K	0.63	0.0026	1.37	0.07	0.77	Albite	Yes	

	series		8						
Si	K series	0.24	0.00188	0.61	0.04	0.28	SiO2	Yes	
P	K series	0.12	0.00070	0.22	0.04	0.09	GaP	Yes	
Cl	K series	0.27	0.00240	0.75	0.05	0.28	NaCl	Yes	
K	K series	0.07	0.00063	0.20	0.04	0.07	KBr	Yes	
Total:				100.00		100.00			

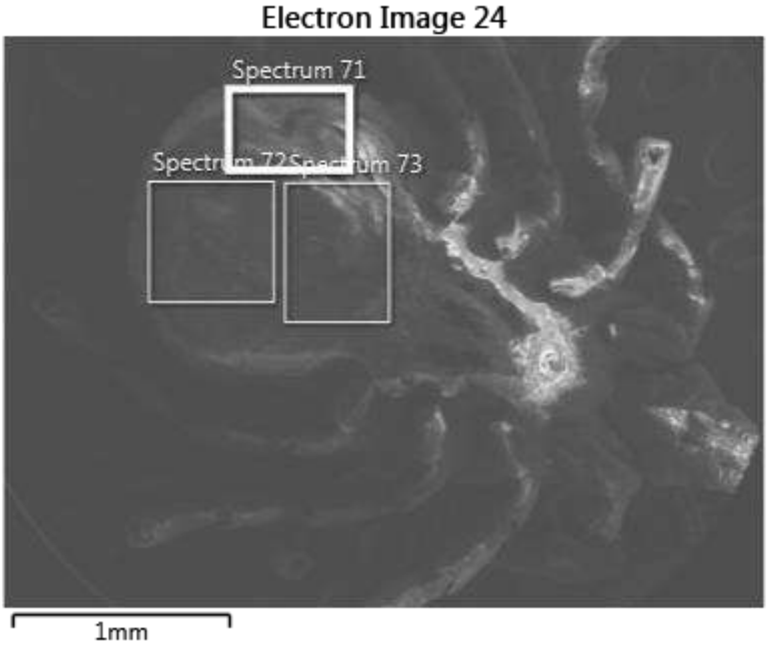

23/01/2020 12:45:37
Project 1
Rumania 7
Ventral 1 15 Kv

Electron Image 24

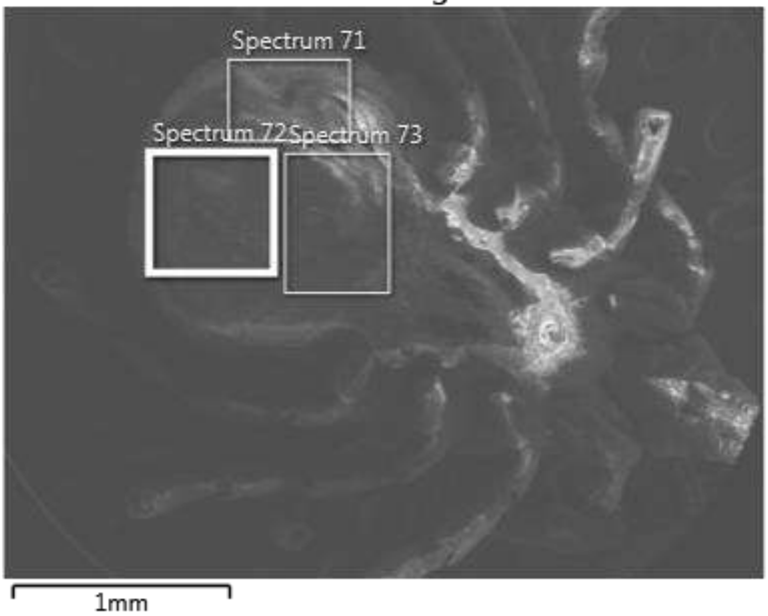

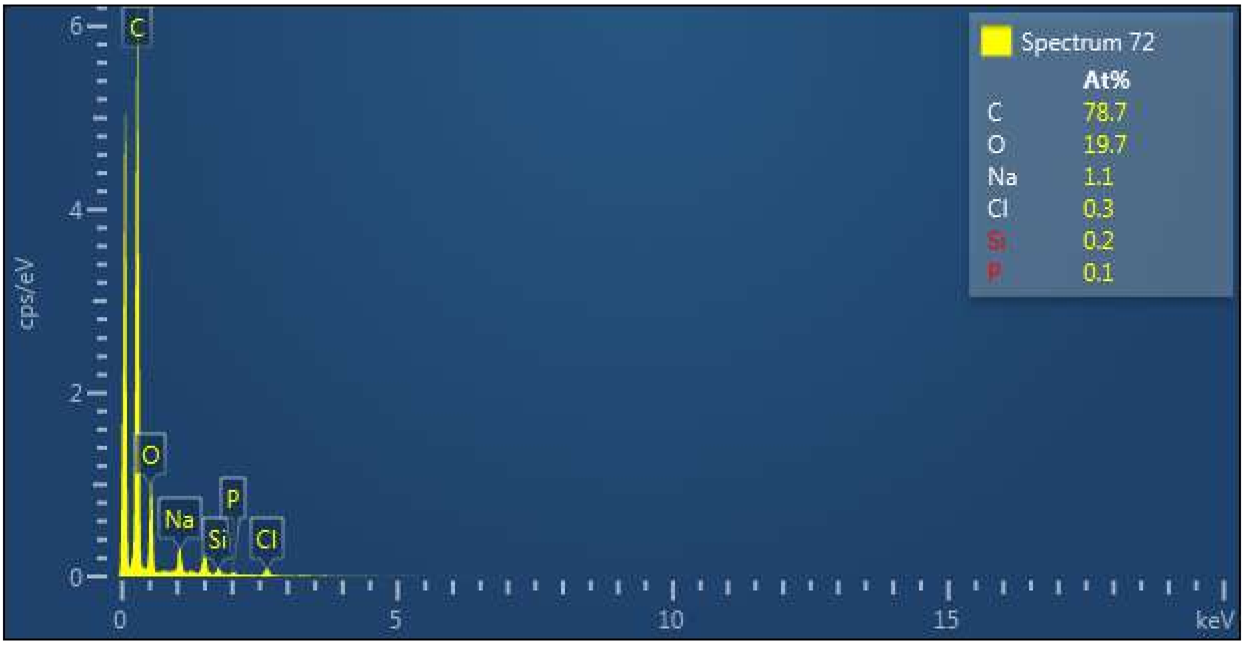

Label:	Spectrum 72
Source:	Acquired
Created:	23/01/2020 12:45:37
Livetime:	60.5s
Process Time:	4
Accelerating Voltage:	15.00kV
Magnification:	32 x
Working Distance:	10.0mm
Specimen Tilt (degrees):	0.0
Elevation (degrees):	35.0
Azimuth (degrees):	0.0
Number Of Channels:	2048
Energy Range (keV):	20 keV
Energy per Channel (eV):	10.0eV
Detector Type Id:	29
Detector Type:	X-Max
Window Type:	SATW
Pulse Pile Up Correction:	Succeeded
Primary Detector:	2617
Primary Detector Serial Number:	77871-X080

Element	Line Type	Apparent Concentration	k Ratio	Wt%	Wt% Sigma	Atomic %	Standard Label	Factory Standard	Standard Calibration Date
C	K series	9.77	0.09766	72.53	0.54	78.66	C Vit	Yes	
O	K series	3.00	0.01011	24.20	0.53	19.71	SiO2	Yes	
Na	K	0.48	0.0020	1.93	0.11	1.09	Albite	Yes	

	series		3						
Si	K series	0.09	0.00071	0.43	0.05	0.20	SiO2	Yes	
P	K series	0.06	0.00034	0.20	0.05	0.08	GaP	Yes	
Cl	K series	0.14	0.00120	0.70	0.07	0.26	NaCl	Yes	
Total:				100.00		100.00			

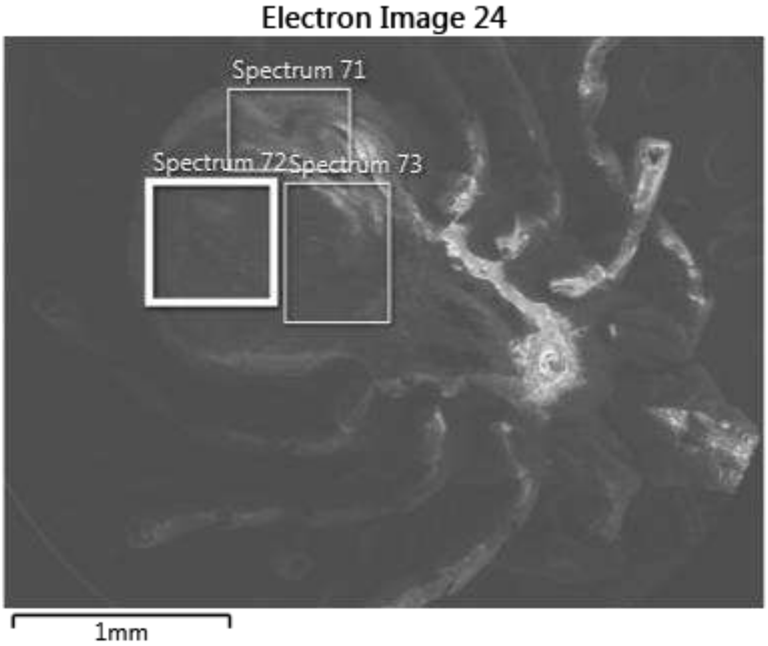

23/01/2020 12:46:48
Project 1
Rumania 7
Ventral 1 15 Kv

Electron Image 24

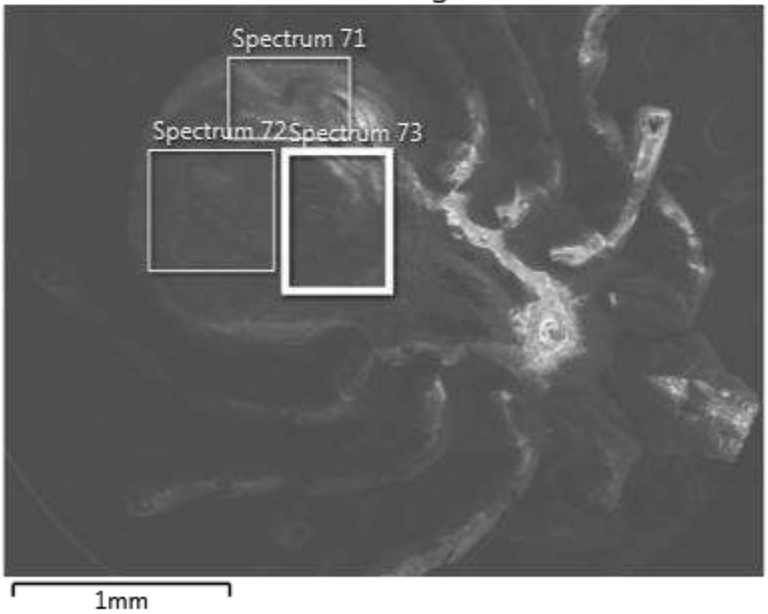

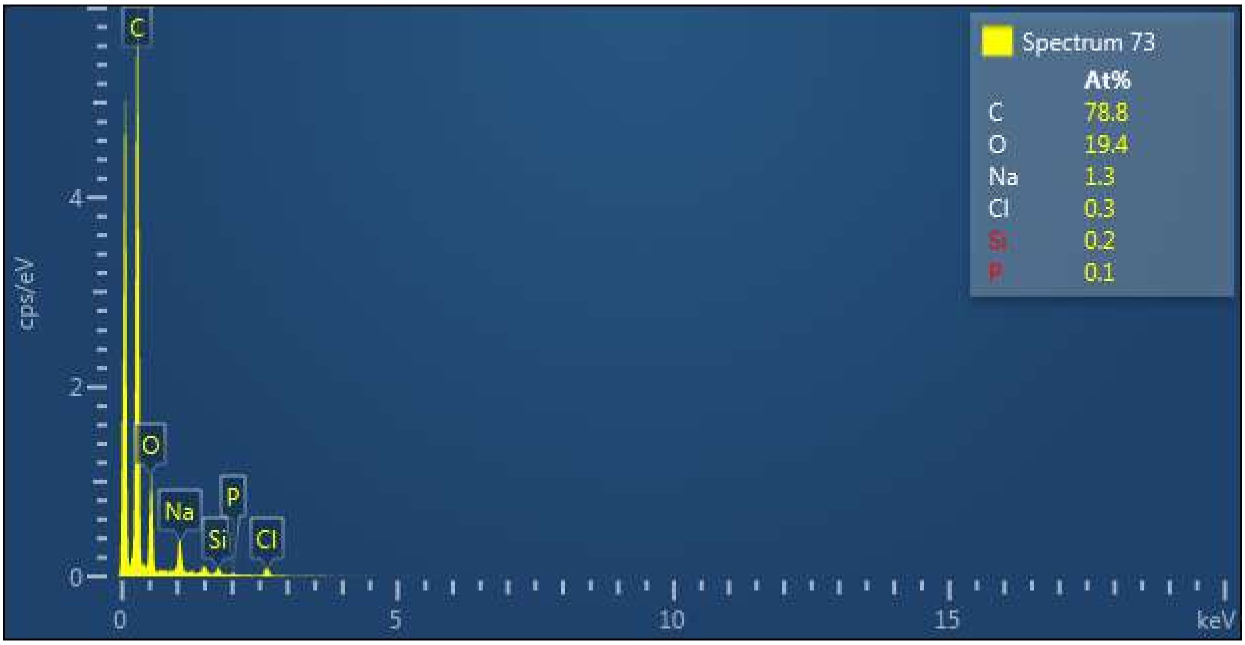

Label:	Spectrum 73
Source:	Acquired
Created:	23/01/2020 12:46:48
Livetime:	60.5s
Process Time:	4
Accelerating Voltage:	15.00kV
Magnification:	32 x
Working Distance:	10.0mm
Specimen Tilt (degrees):	0.0
Elevation (degrees):	35.0
Azimuth (degrees):	0.0
Number Of Channels:	2048
Energy Range (keV):	20 keV
Energy per Channel (eV):	10.0eV
Detector Type Id:	29
Detector Type:	X-Max
Window Type:	SATW
Pulse Pile Up Correction:	Succeeded
Primary Detector:	2617
Primary Detector Serial Number:	77871-X080

Element	Line Type	Apparent Concentration	k Ratio	Wt%	Wt% Sigma	Atomic %	Standard Label	Factory Standard	Standard Calibration Date
C	K series	9.76	0.09764	72.59	0.55	78.78	C Vit	Yes	
O	K series	2.96	0.00996	23.76	0.54	19.36	SiO2	Yes	
Na	K	0.59	0.0025	2.37	0.12	1.34	Albite	Yes	

	series		1						
Si	K series	0.09	0.00067	0.41	0.05	0.19	SiO2	Yes	
P	K series	0.04	0.00024	0.14	0.04	0.06	GaP	Yes	
Cl	K series	0.14	0.00125	0.73	0.06	0.27	NaCl	Yes	
Total:				100.00		100.00			

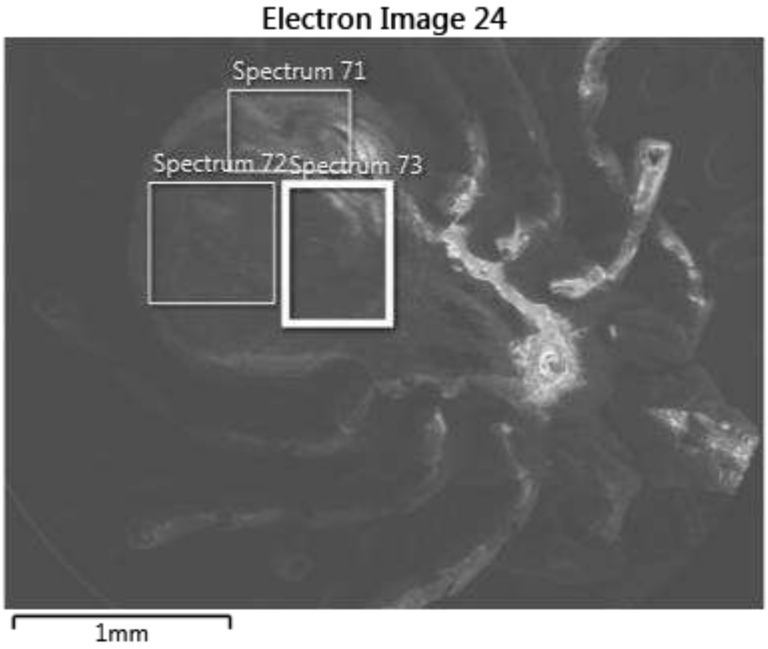

23/01/2020 12:49:07
Project 1
Rumania 8
Pecho 1 15 Kv

Electron Image 25

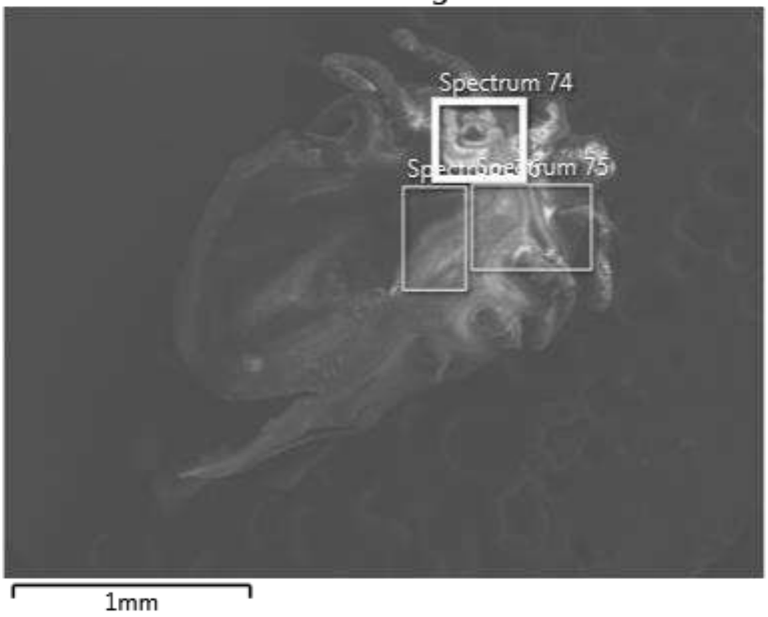

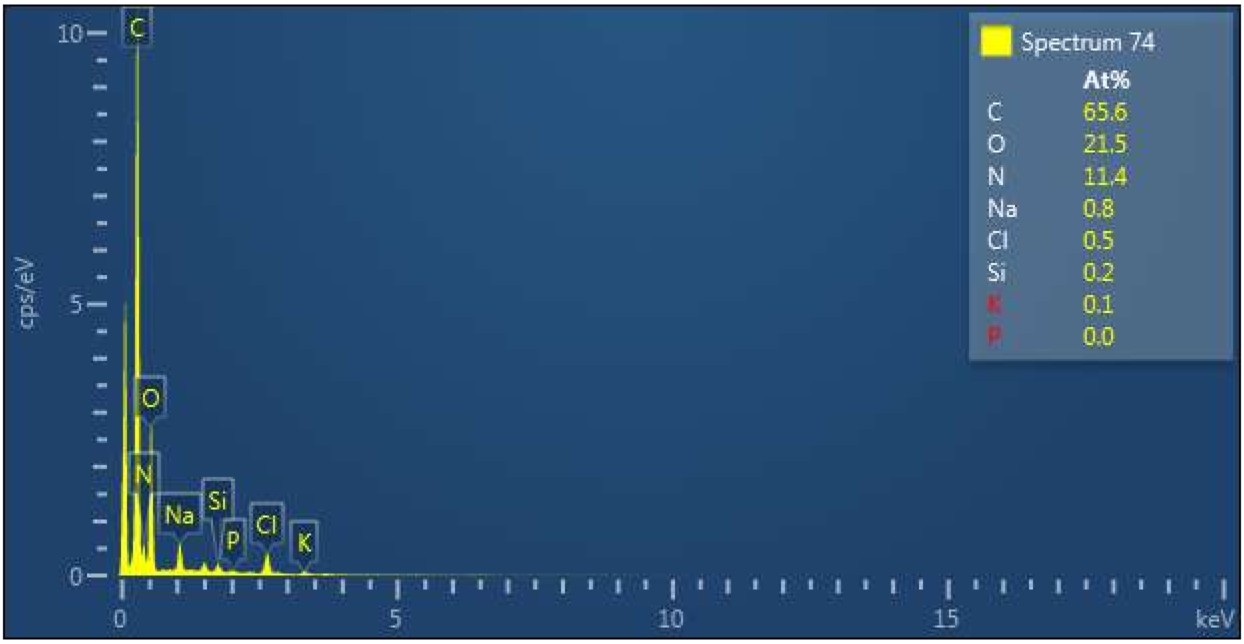

Label:	Spectrum 74
Source:	Acquired
Created:	23/01/2020 12:49:07
Livetime:	60.1s
Process Time:	4
Accelerating Voltage:	15.00kV
Magnification:	35 x
Working Distance:	10.0mm
Specimen Tilt (degrees):	0.0
Elevation (degrees):	35.0
Azimuth (degrees):	0.0
Number Of Channels:	2048
Energy Range (keV):	20 keV
Energy per Channel (eV):	10.0eV
Detector Type Id:	29
Detector Type:	X-Max
Window Type:	SATW
Pulse Pile Up Correction:	Succeeded
Primary Detector:	2617
Primary Detector Serial Number:	77871-X080

Element	Line Type	Apparent Concentration	k Ratio	Wt%	Wt% Sigma	Atomic %	Standard Label	Factory Standard	Standard Calibration Date
C	K series	18.89	0.18889	59.00	0.81	65.58	C Vit	Yes	
N	K series	4.26	0.00758	11.94	1.04	11.39	BN	Yes	
O	K	7.82	0.0263	25.71	0.48	21.46	SiO2	Yes	

	series		2						
Na	K series	0.89	0.00374	1.42	0.06	0.83	Albite	Yes	
Si	K series	0.18	0.00141	0.33	0.03	0.16	SiO2	Yes	
P	K series	0.08	0.00043	0.10	0.03	0.04	GaP	Yes	
Cl	K series	0.65	0.00566	1.28	0.05	0.48	NaCl	Yes	
K	K series	0.11	0.00095	0.21	0.03	0.07	KBr	Yes	
Total:				100.00		100.00			

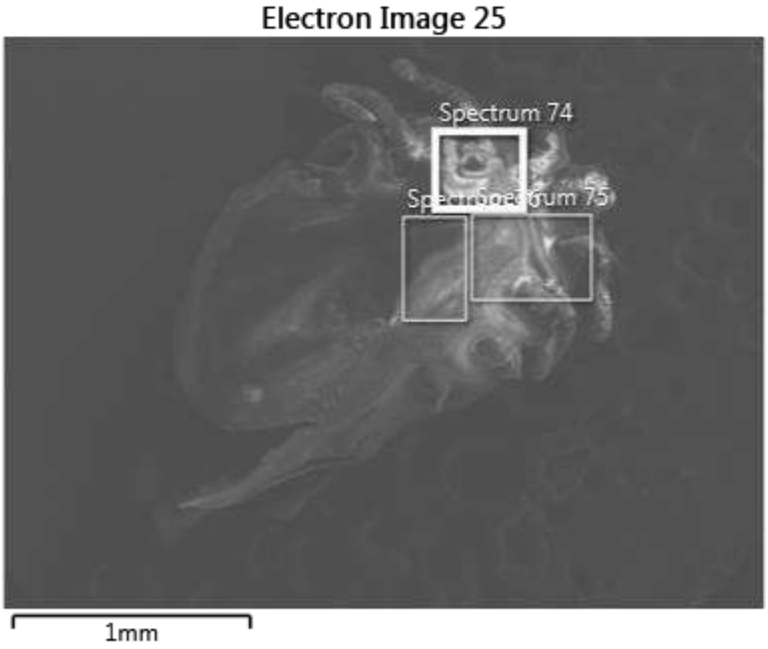

23/01/2020 12:50:20
Project 1
Rumania 8
Pecho 1 15 Kv

Electron Image 25

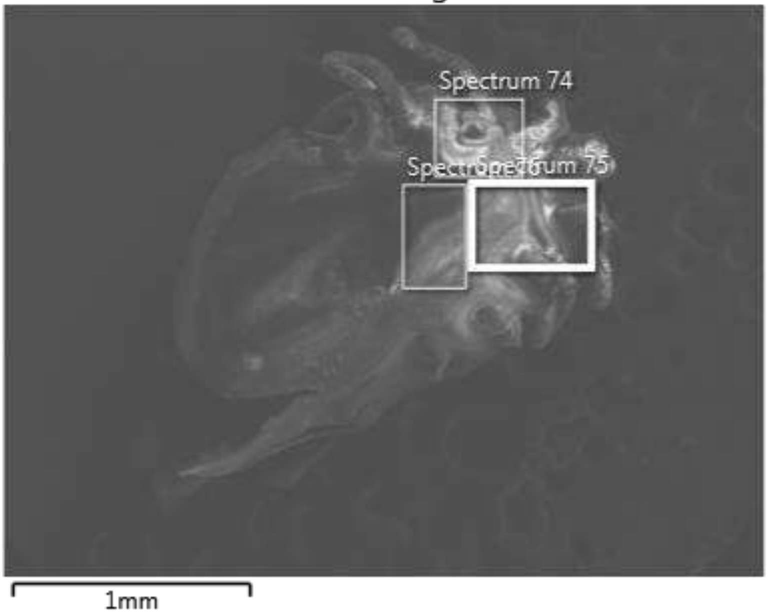

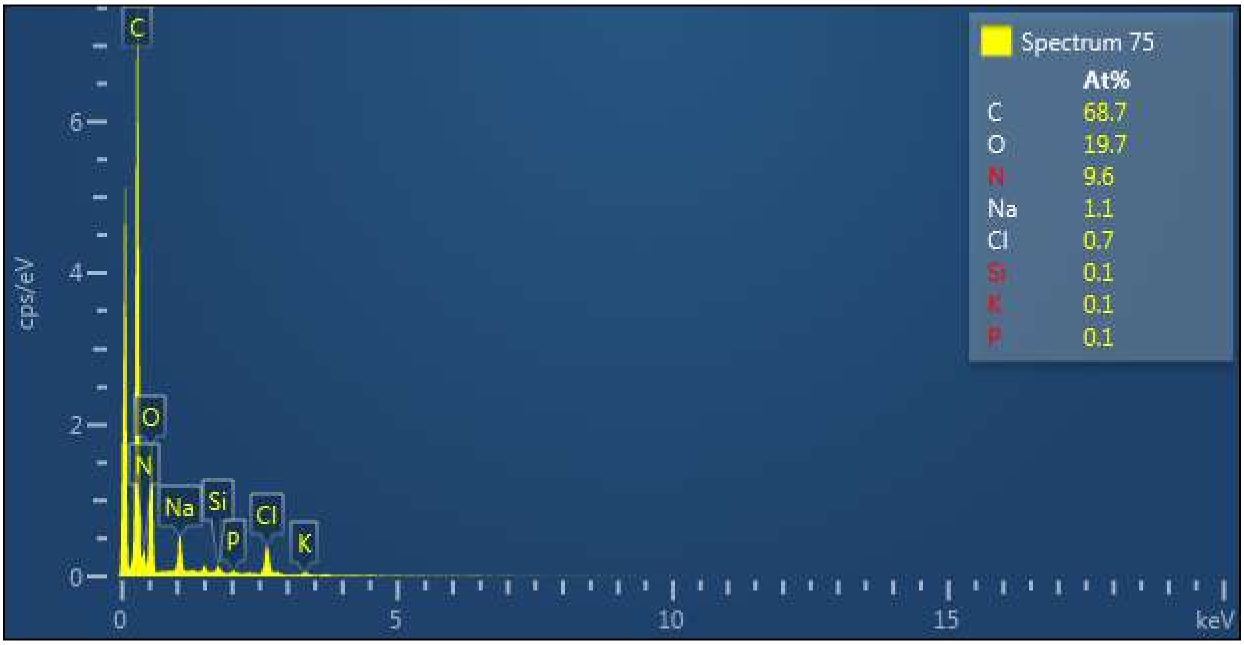

Label:	Spectrum 75
Source:	Acquired
Created:	23/01/2020 12:50:20
Livetime:	60.5s
Process Time:	4
Accelerating Voltage:	15.00kV
Magnification:	35 x
Working Distance:	10.0mm
Specimen Tilt (degrees):	0.0
Elevation (degrees):	35.0
Azimuth (degrees):	0.0
Number Of Channels:	2048
Energy Range (keV):	20 keV
Energy per Channel (eV):	10.0eV
Detector Type Id:	29
Detector Type:	X-Max
Window Type:	SATW
Pulse Pile Up Correction:	Succeeded
Primary Detector:	2617
Primary Detector Serial Number:	77871-X080

Element	Line Type	Apparent Concentration	k Ratio	Wt%	Wt% Sigma	Atomic %	Standard Label	Factory Standard	Standard Calibration Date
C	K series	13.43	0.13435	61.92	1.00	68.65	C Vit	Yes	
N	K series	2.42	0.00430	10.10	1.28	9.60	BN	Yes	
O	K	5.08	0.0170	23.71	0.55	19.73	SiO2	Yes	

	series		9						
Na	K series	0.84	0.00354	1.86	0.08	1.08	Albite	Yes	
Si	K series	0.10	0.00077	0.25	0.04	0.12	SiO2	Yes	
P	K series	0.08	0.00042	0.14	0.03	0.06	GaP	Yes	
Cl	K series	0.65	0.00570	1.81	0.08	0.68	NaCl	Yes	
K	K series	0.08	0.00070	0.22	0.04	0.08	KBr	Yes	
Total:				100.00		100.00			

Electron Image 25

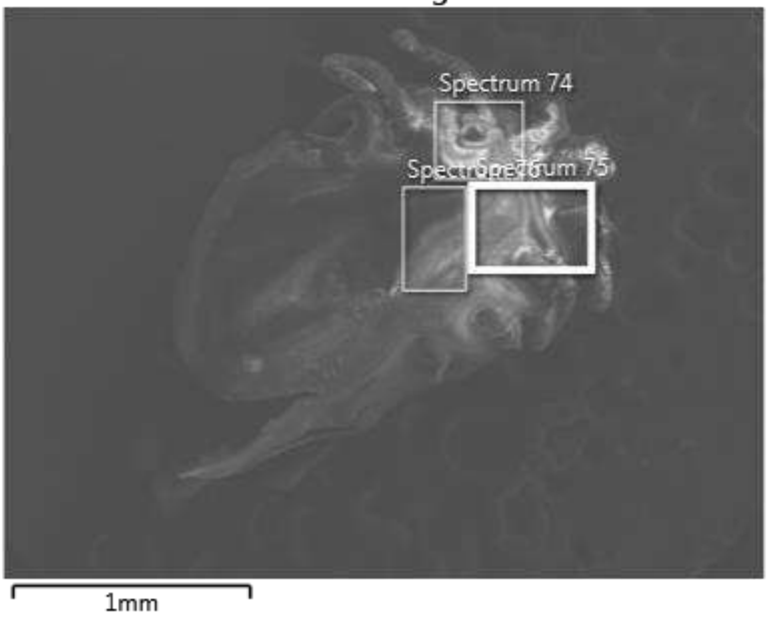

23/01/2020 12:51:31
Project 1
Rumania 8
Pecho 1 15 Kv

Electron Image 25

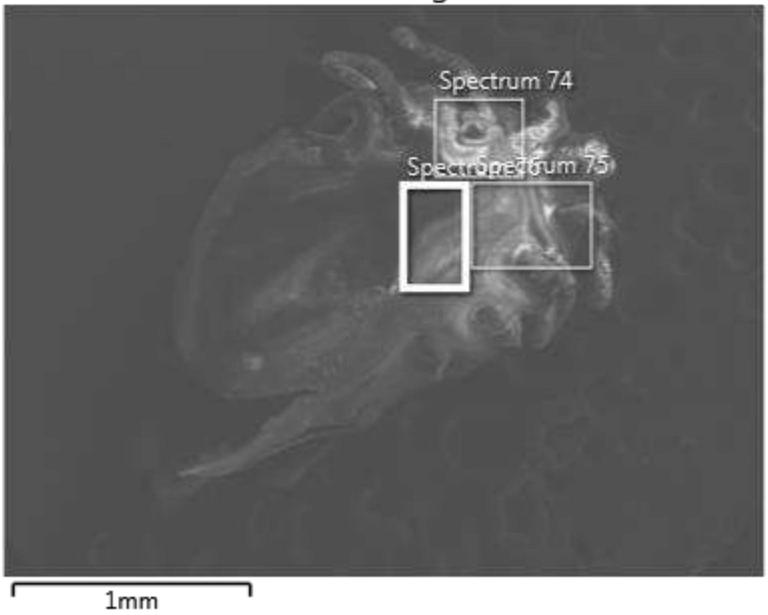

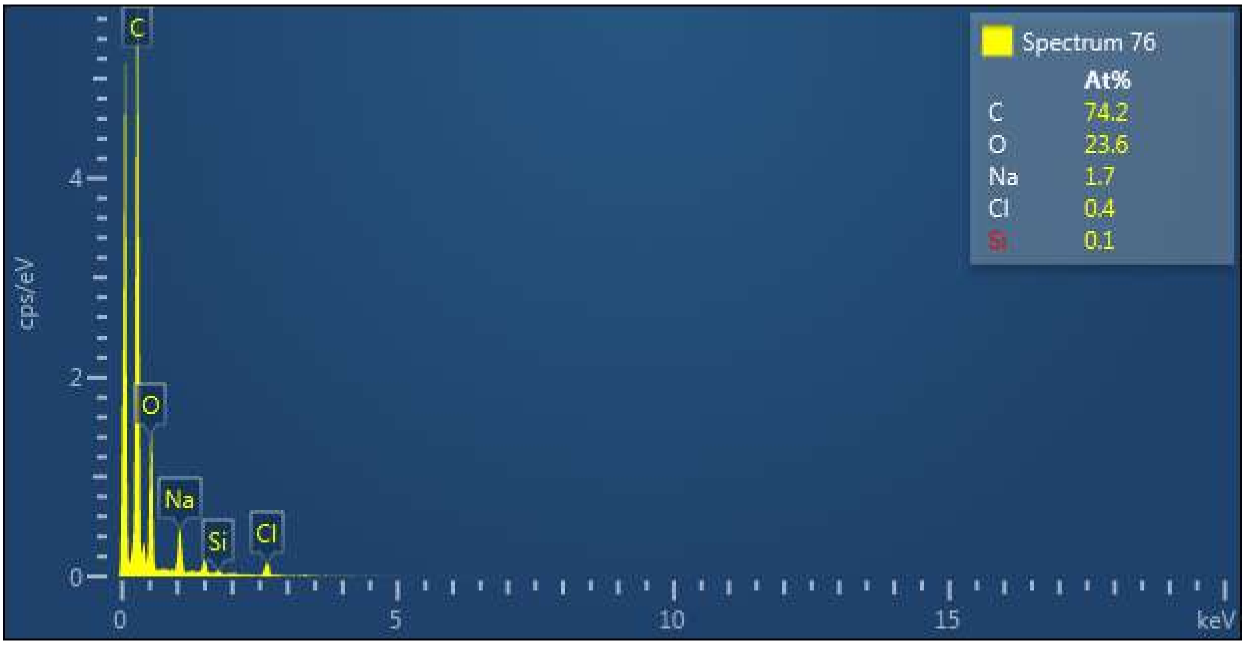

Label:	Spectrum 76
Source:	Acquired
Created:	23/01/2020 12:51:31
Livetime:	60.3s
Process Time:	4
Accelerating Voltage:	15.00kV
Magnification:	35 x
Working Distance:	10.0mm
Specimen Tilt (degrees):	0.0
Elevation (degrees):	35.0
Azimuth (degrees):	0.0
Number Of Channels:	2048
Energy Range (keV):	20 keV
Energy per Channel (eV):	10.0eV
Detector Type Id:	29
Detector Type:	X-Max
Window Type:	SATW
Pulse Pile Up Correction:	Succeeded
Primary Detector:	2617
Primary Detector Serial Number:	77871-X080

Element	Line Type	Apparent Concentration	k Ratio	Wt%	Wt% Sigma	Atomic %	Standard Label	Factory Standard	Standard Calibration Date
C	K series	9.03	0.09032	67.31	0.54	74.24	C Vit	Yes	
O	K series	4.08	0.01373	28.47	0.54	23.57	SiO2	Yes	
Na	K	0.77	0.0032	2.92	0.13	1.68	Albite	Yes	

	series		4						
Si	K series	0.05	0.00037	0.21	0.04	0.10	SiO2	Yes	
Cl	K series	0.23	0.00200	1.09	0.07	0.41	NaCl	Yes	
Total:				100.00		100.00			

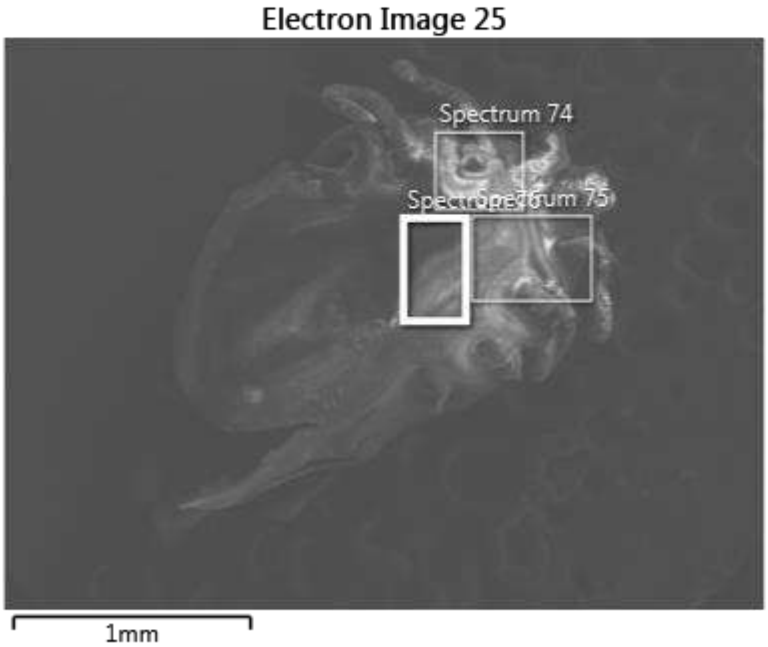

23/01/2020 12:53:29
Project 1
Rumania 8
Ventral 1 15 Kv

Electron Image 26

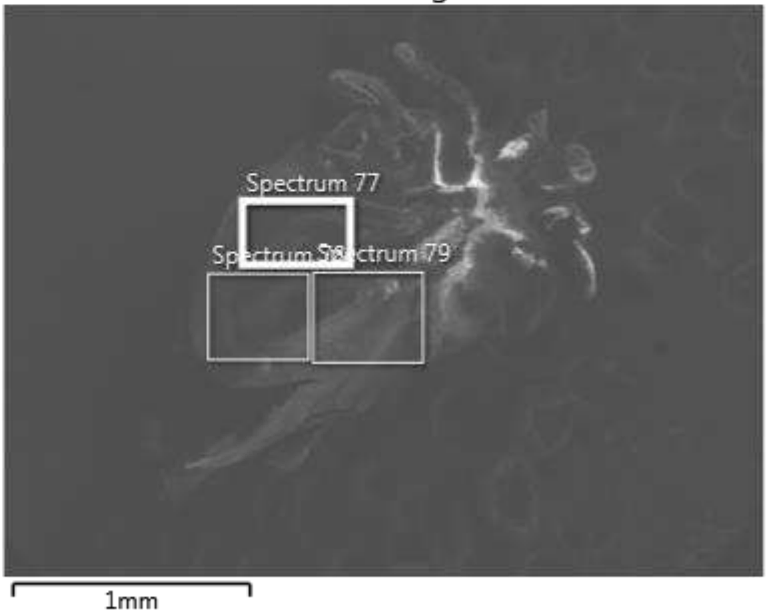

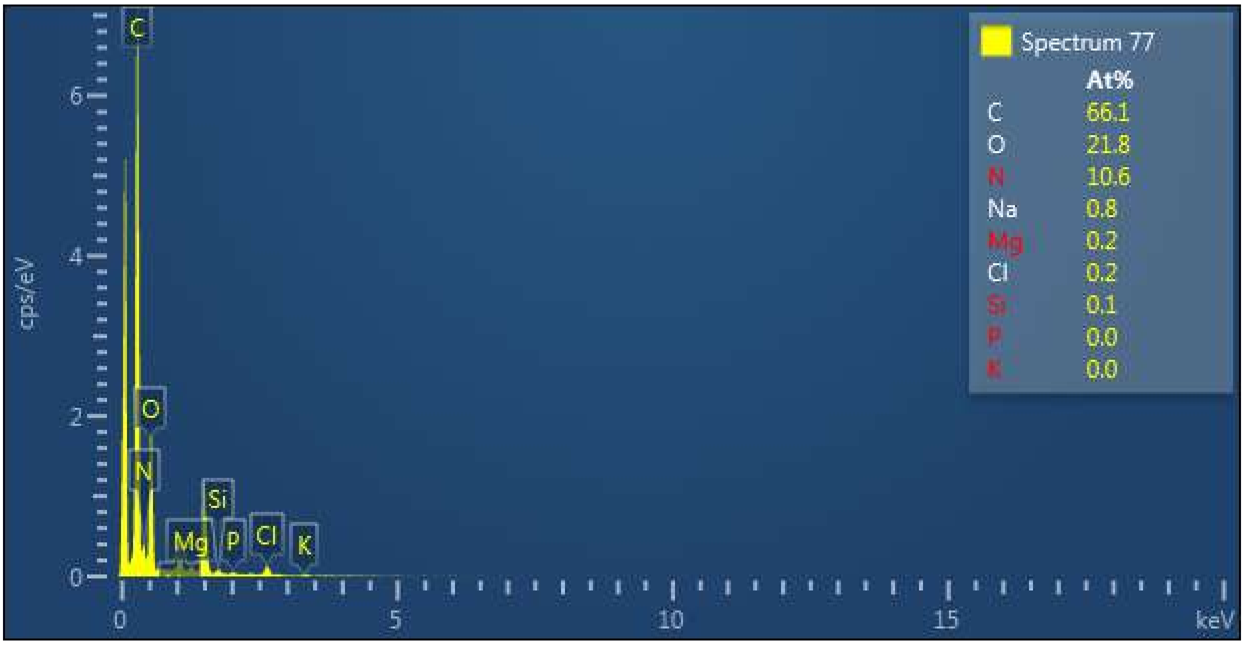

Label:	Spectrum 77
Source:	Acquired
Created:	23/01/2020 12:53:29
Livetime:	60.5s
Process Time:	4
Accelerating Voltage:	15.00kV
Magnification:	35 x
Working Distance:	10.0mm
Specimen Tilt (degrees):	0.0
Elevation (degrees):	35.0
Azimuth (degrees):	0.0
Number Of Channels:	2048
Energy Range (keV):	20 keV
Energy per Channel (eV):	10.0eV
Detector Type Id:	29
Detector Type:	X-Max
Window Type:	SATW
Pulse Pile Up Correction:	Succeeded
Primary Detector:	2617
Primary Detector Serial Number:	77871-X080

Element	Line Type	Apparent Concentration	k Ratio	Wt%	Wt% Sigma	Atomic %	Standard Label	Factory Standard	Standard Calibration Date
C	K series	12.84	0.12841	59.67	1.07	66.11	C Vit	Yes	
N	K series	2.52	0.00448	11.12	1.41	10.57	BN	Yes	
O	K	5.14	0.0172	26.19	0.63	21.79	SiO2	Yes	

	series		9						
Na	K series	0.57	0.00242	1.45	0.08	0.84	Albite	Yes	
Mg	K series	0.14	0.00092	0.44	0.05	0.24	MgO	Yes	
Si	K series	0.10	0.00077	0.29	0.04	0.14	SiO2	Yes	
P	K series	0.06	0.00031	0.11	0.03	0.05	GaP	Yes	
Cl	K series	0.20	0.00172	0.61	0.05	0.23	NaCl	Yes	
K	K series	0.04	0.00034	0.12	0.03	0.04	KBr	Yes	
Total:				100.00		100.00			

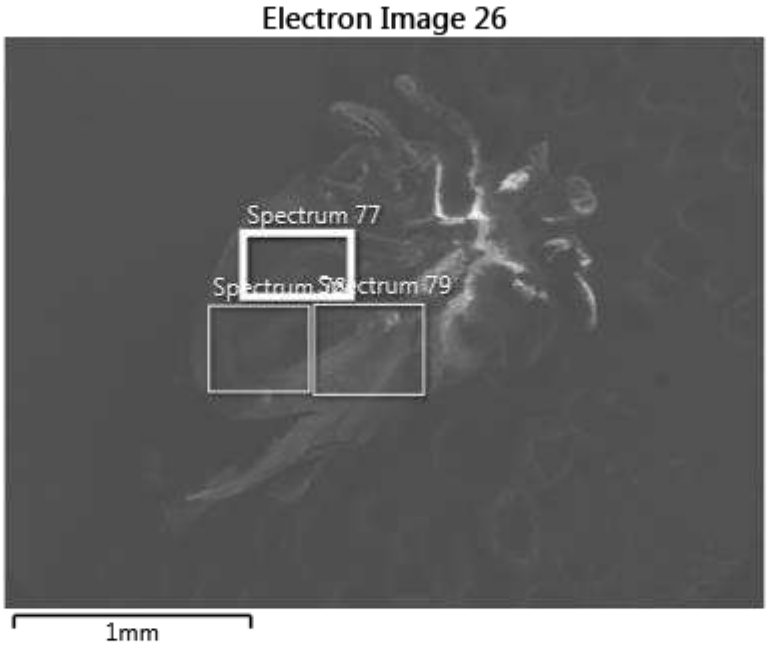

23/01/2020 12:54:42
Project 1
Rumania 8
Ventral 1 15 Kv

Electron Image 26

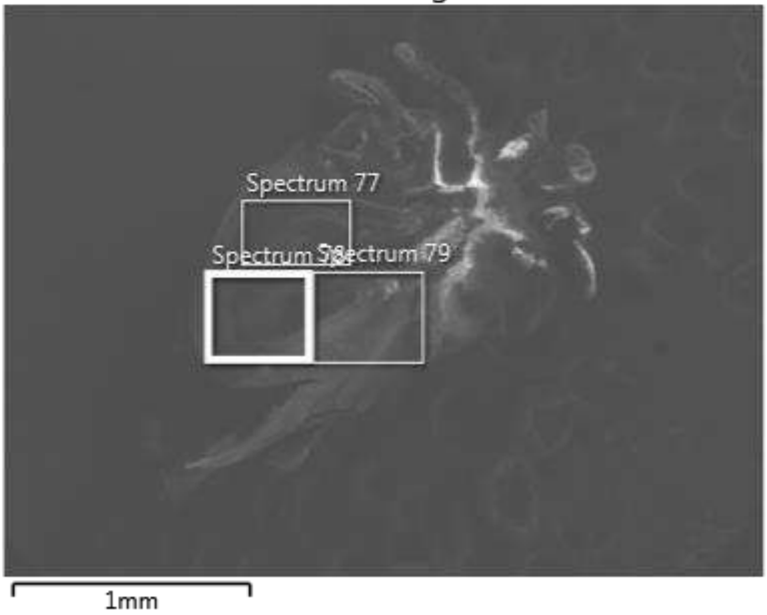

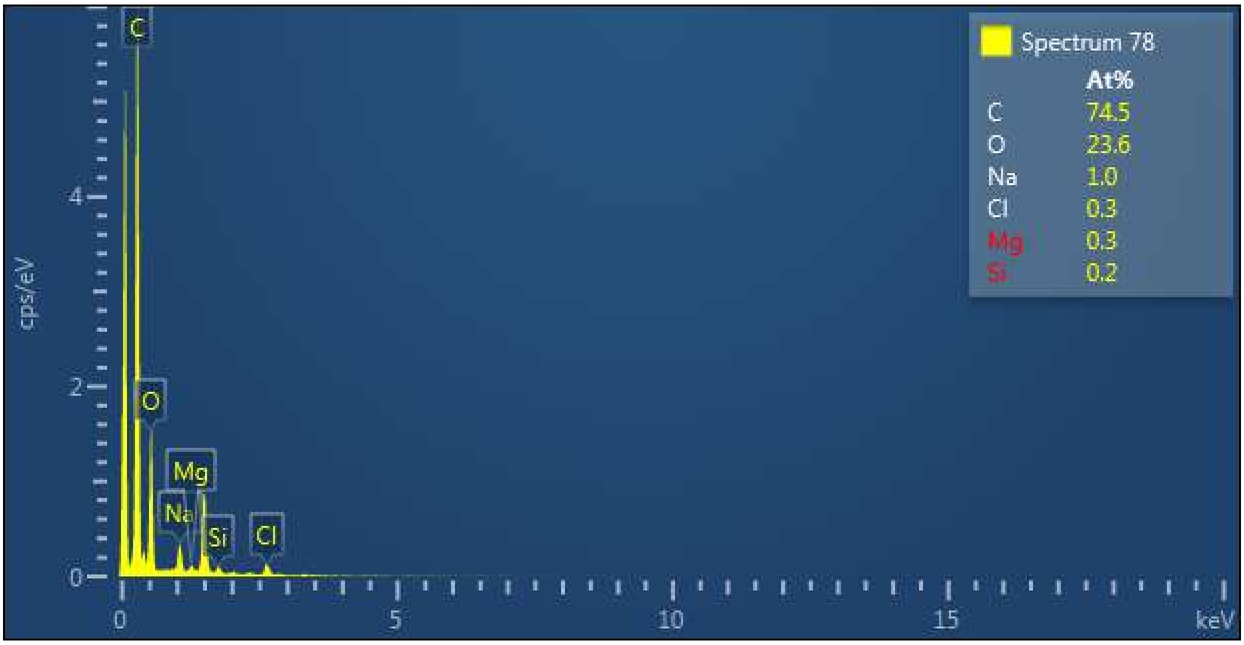

Label:	Spectrum 78
Source:	Acquired
Created:	23/01/2020 12:54:42
Livetime:	60.2s
Process Time:	4
Accelerating Voltage:	15.00kV
Magnification:	35 x
Working Distance:	10.0mm
Specimen Tilt (degrees):	0.0
Elevation (degrees):	35.0
Azimuth (degrees):	0.0
Number Of Channels:	2048
Energy Range (keV):	20 keV
Energy per Channel (eV):	10.0eV
Detector Type Id:	29
Detector Type:	X-Max
Window Type:	SATW
Pulse Pile Up Correction:	Succeeded
Primary Detector:	2617
Primary Detector Serial Number:	77871-X080

Element	Line Type	Apparent Concentration	k Ratio	Wt%	Wt% Sigma	Atomic %	Standard Label	Factory Standard	Standard Calibration Date
C	K series	9.67	0.09673	67.74	0.53	74.52	C Vit	Yes	
O	K series	4.27	0.01437	28.61	0.52	23.63	SiO2	Yes	
Na	K	0.49	0.0020	1.78	0.11	1.02	Albite	Yes	

	series		6						
Mg	K series	0.11	0.00073	0.50	0.06	0.27	MgO	Yes	
Si	K series	0.12	0.00091	0.50	0.05	0.23	SiO2	Yes	
Cl	K series	0.19	0.00167	0.87	0.07	0.33	NaCl	Yes	
Total:				100.00		100.00			

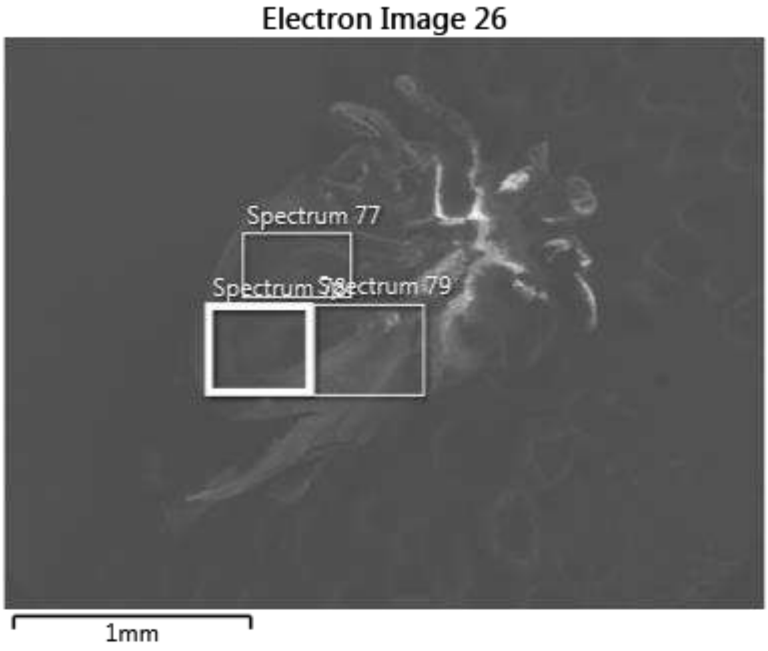

23/01/2020 12:55:53
Project 1
Rumania 8
Ventral 1 15 Kv

Electron Image 26

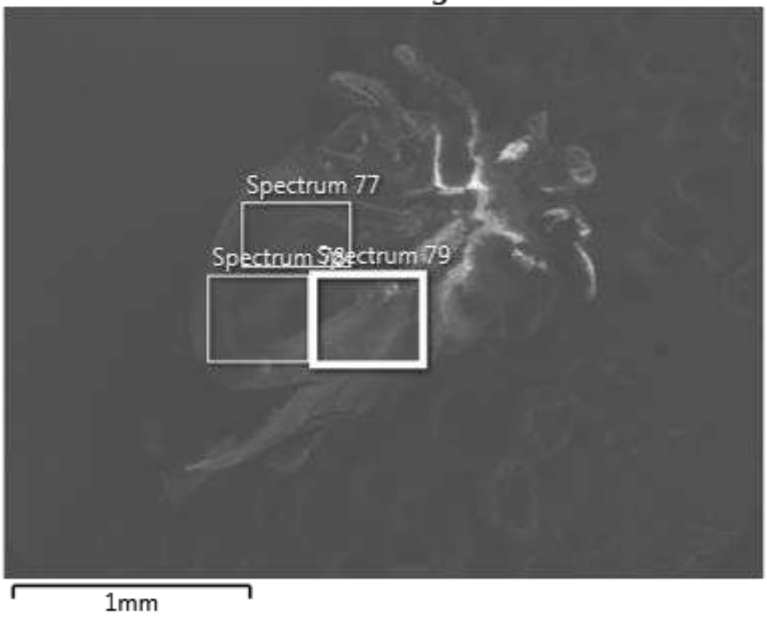

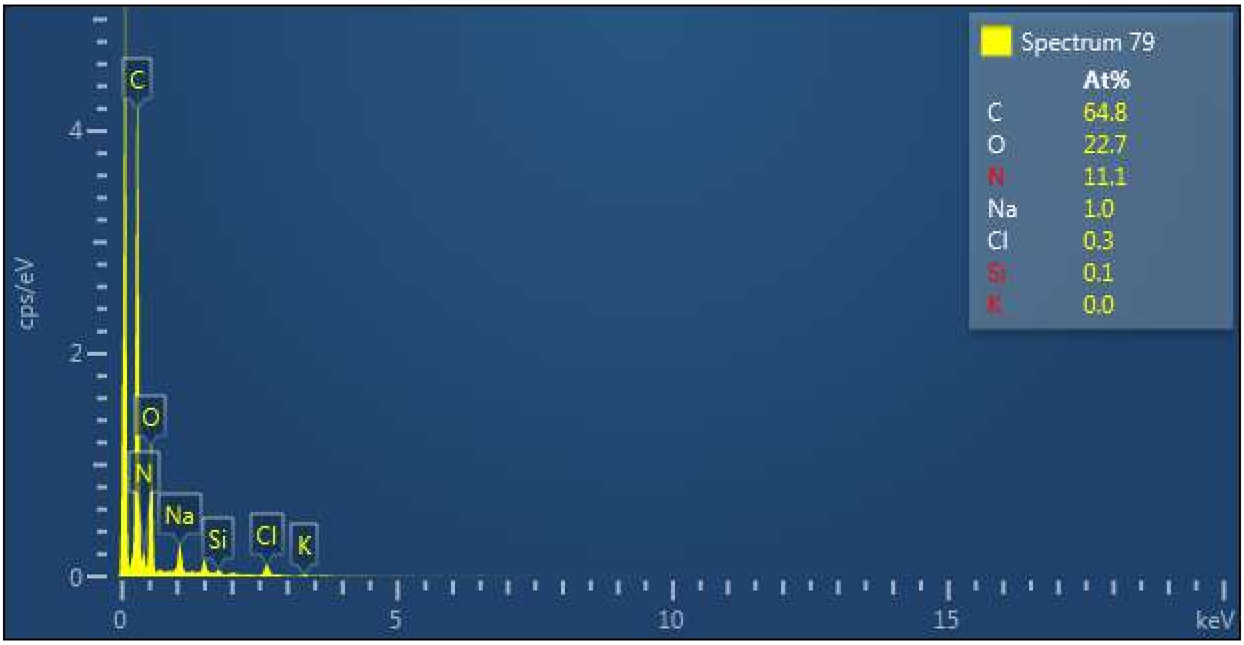

Label:	Spectrum 79
Source:	Acquired
Created:	23/01/2020 12:55:53
Livetime:	60.3s
Process Time:	4
Accelerating Voltage:	15.00kV
Magnification:	35 x
Working Distance:	10.0mm
Specimen Tilt (degrees):	0.0
Elevation (degrees):	35.0
Azimuth (degrees):	0.0
Number Of Channels:	2048
Energy Range (keV):	20 keV
Energy per Channel (eV):	10.0eV
Detector Type Id:	29
Detector Type:	X-Max
Window Type:	SATW
Pulse Pile Up Correction:	Succeeded
Primary Detector:	2617
Primary Detector Serial Number:	77871-X080

Element	Line Type	Apparent Concentration	k Ratio	Wt%	Wt% Sigma	Atomic %	Standard Label	Factory Standard	Standard Calibration Date
C	K series	7.93	0.07932	58.27	1.21	64.76	C Vit	Yes	
N	K series	1.72	0.00307	11.63	1.59	11.08	BN	Yes	
O	K	3.44	0.0115	27.21	0.76	22.70	SiO2	Yes	

	series		9						
Na	K series	0.43	0.00180	1.70	0.11	0.99	Albite	Yes	
Si	K series	0.05	0.00040	0.23	0.04	0.11	SiO2	Yes	
Cl	K series	0.17	0.00148	0.82	0.07	0.31	NaCl	Yes	
K	K series	0.03	0.00024	0.13	0.04	0.05	KBr	Yes	
Total:				100.00		100.00			

Electron Image 26

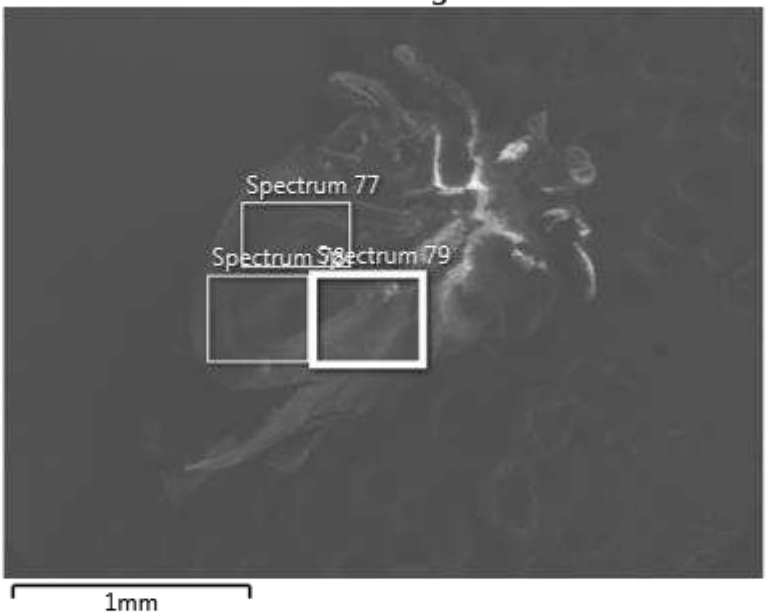

23/01/2020 12:58:48
Project 1
Rumania 9
Pecho 1 15 Kv

Electron Image 27

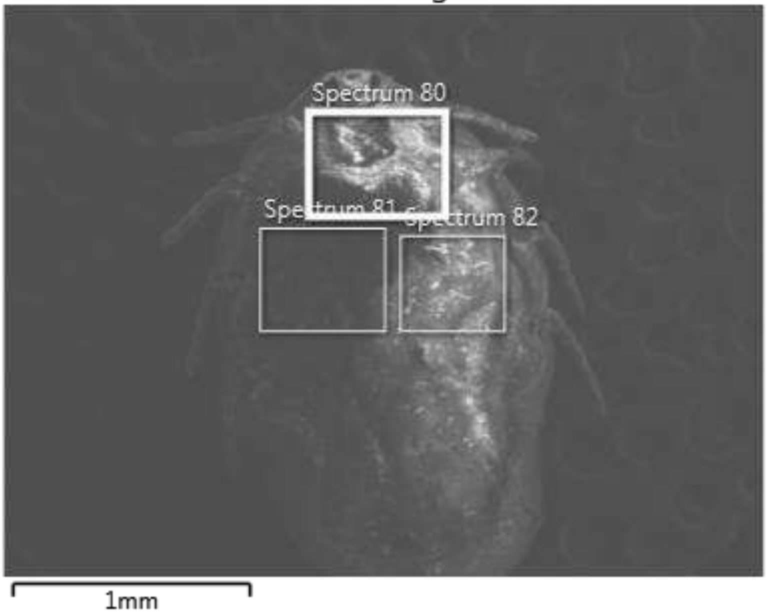

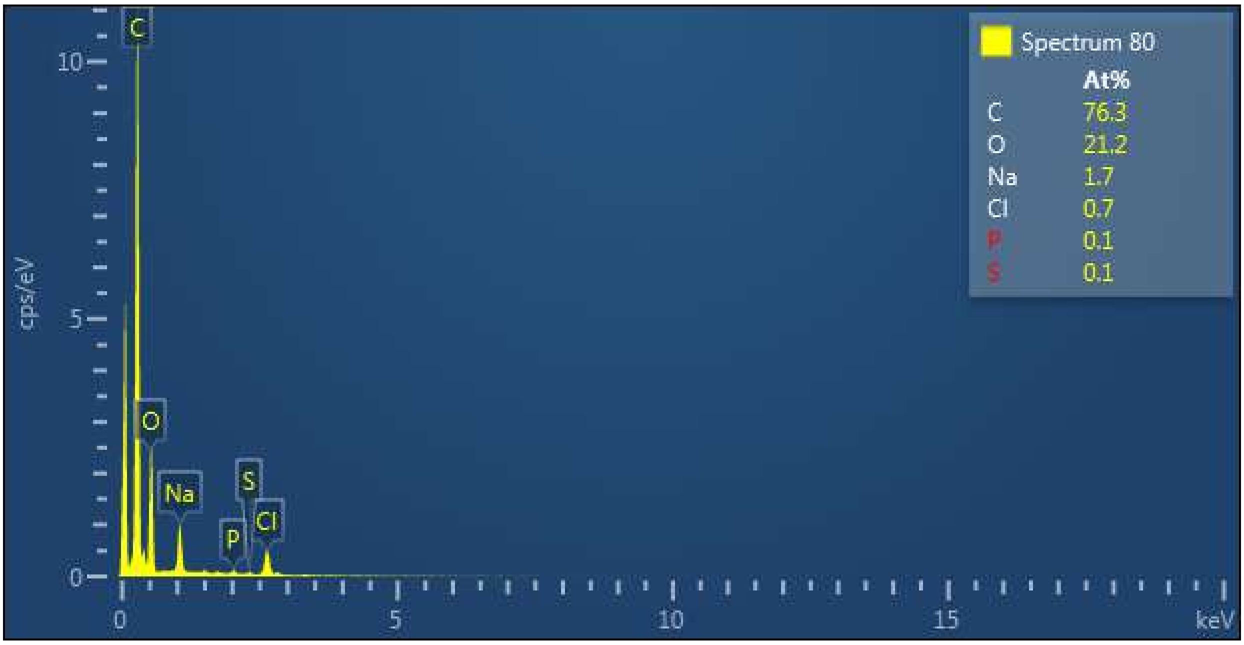

Label:	Spectrum 80
Source:	Acquired
Created:	23/01/2020 12:58:48
Livetime:	60.4s
Process Time:	4
Accelerating Voltage:	15.00kV
Magnification:	35 x
Working Distance:	10.0mm
Specimen Tilt (degrees):	0.0
Elevation (degrees):	35.0
Azimuth (degrees):	0.0
Number Of Channels:	2048
Energy Range (keV):	20 keV
Energy per Channel (eV):	10.0eV
Detector Type Id:	29
Detector Type:	X-Max
Window Type:	SATW
Pulse Pile Up Correction:	Succeeded
Primary Detector:	2617
Primary Detector Serial Number:	77871-X080

Element	Line Type	Apparent Concentration	k Ratio	Wt%	Wt% Sigma	Atomic %	Standard Label	Factory Standard	Standard Calibration Date
C	K series	17.27	0.17274	69.22	0.38	76.29	C Vit	Yes	
O	K series	7.15	0.02405	25.56	0.38	21.15	SiO2	Yes	
Na	K	1.58	0.0066	2.91	0.09	1.67	Albite	Yes	

	series		7						
P	K series	0.15	0.00084	0.23	0.04	0.10	GaP	Yes	
S	K series	0.07	0.00059	0.15	0.03	0.06	FeS2	Yes	
Cl	K series	0.82	0.00719	1.93	0.07	0.72	NaCl	Yes	
Total:				100.00		100.00			

Electron Image 27

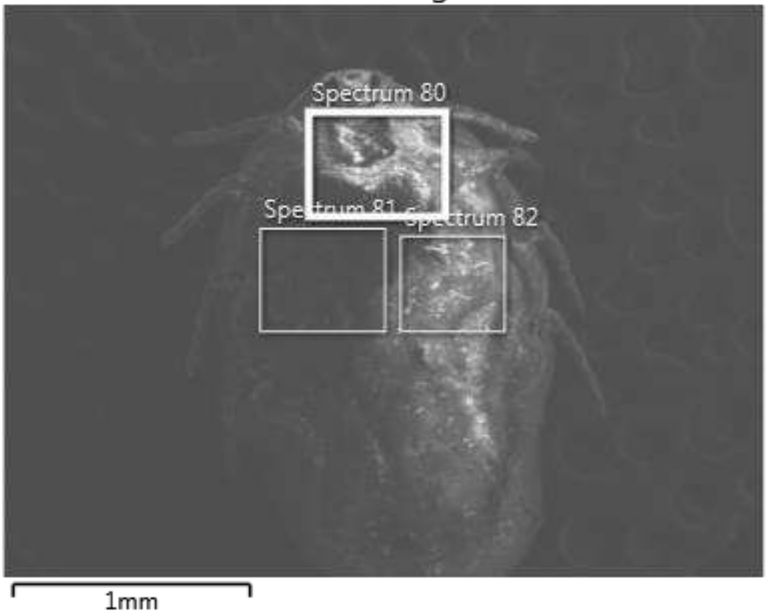

23/01/2020 12:59:58
Project 1
Rumania 9
Pecho 1 15 Kv

Electron Image 27

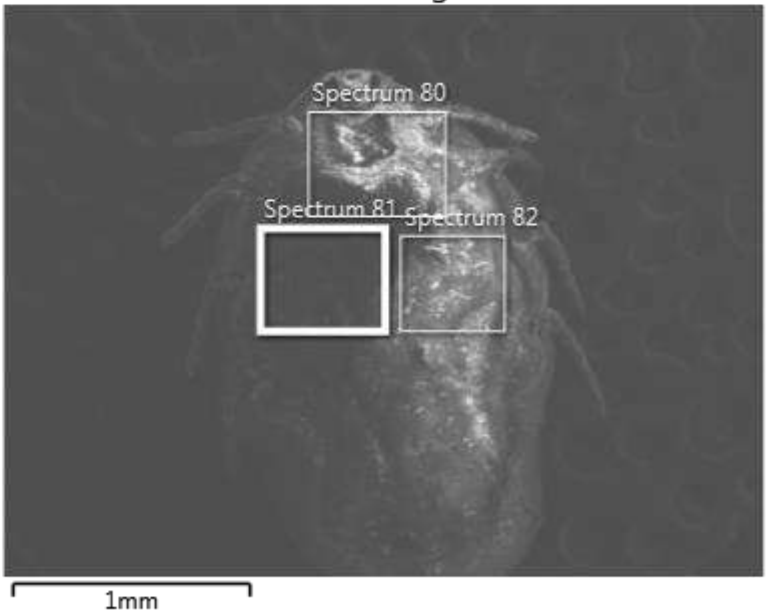

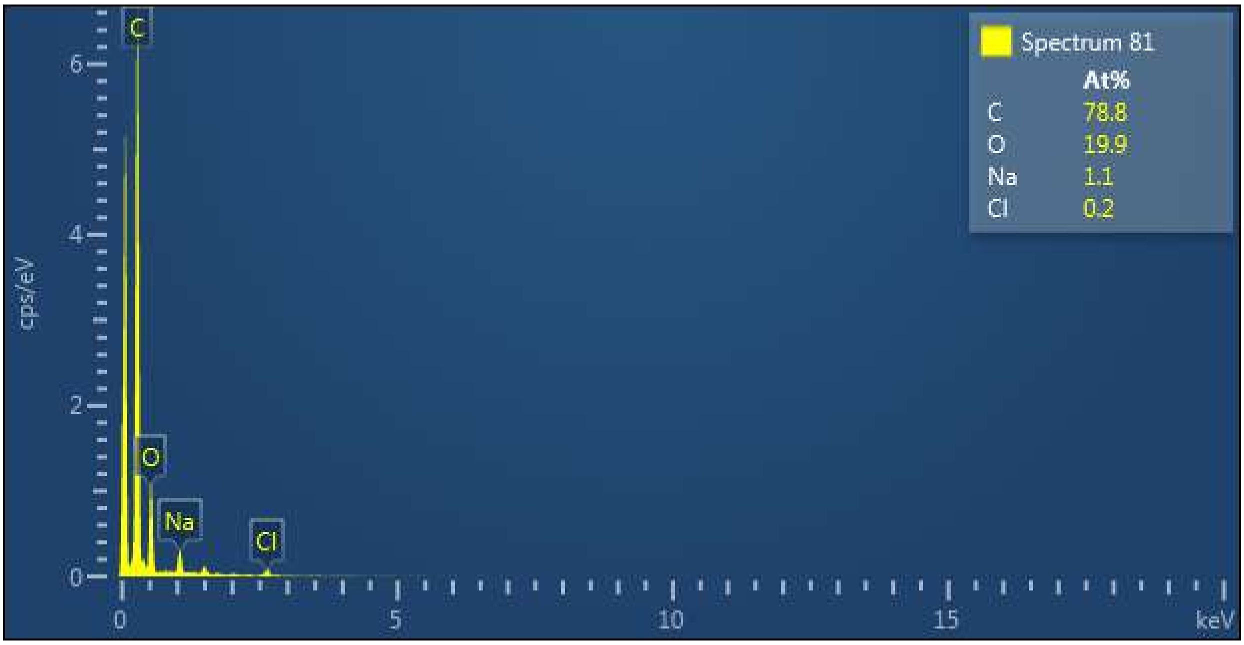

Label:	Spectrum 81
Source:	Acquired
Created:	23/01/2020 12:59:58
Livetime:	60.3s
Process Time:	4
Accelerating Voltage:	15.00kV
Magnification:	35 x
Working Distance:	10.0mm
Specimen Tilt (degrees):	0.0
Elevation (degrees):	35.0
Azimuth (degrees):	0.0
Number Of Channels:	2048
Energy Range (keV):	20 keV
Energy per Channel (eV):	10.0eV
Detector Type Id:	29
Detector Type:	X-Max
Window Type:	SATW
Pulse Pile Up Correction:	Succeeded
Primary Detector:	2617
Primary Detector Serial Number:	77871-X080

Element	Line Type	Apparent Concentration	k Ratio	Wt%	Wt% Sigma	Atomic %	Standard Label	Factory Standard	Standard Calibration Date
C	K series	10.70	0.10699	72.93	0.53	78.79	C Vit	Yes	
O	K series	3.14	0.01058	24.55	0.53	19.91	SiO2	Yes	
Na	K	0.48	0.0020	1.88	0.11	1.06	Albite	Yes	

	series		3						
Cl	K series	0.13	0.00113	0.64	0.06	0.23	NaCl	Yes	
Total:				100.00		100.00			

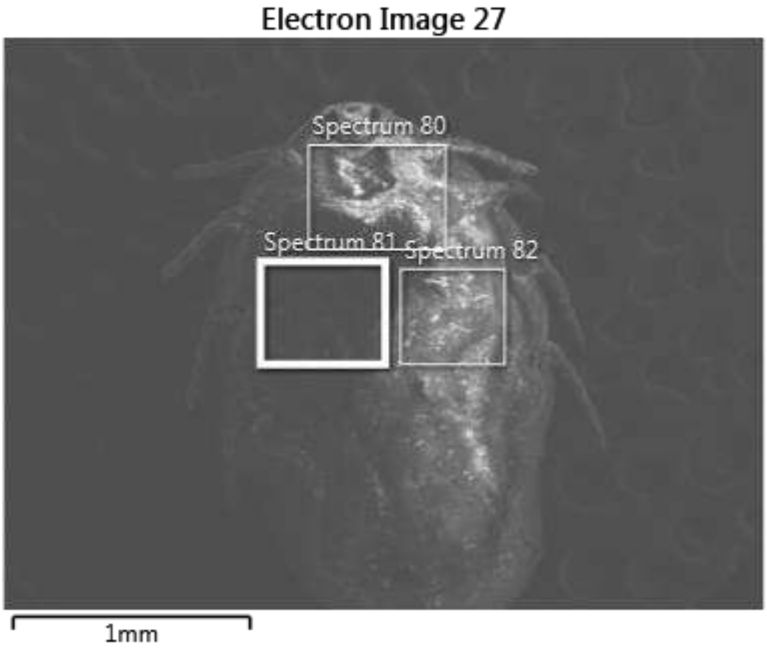

23/01/2020 13:01:08
Project 1
Rumania 9
Pecho 1 15 Kv

Electron Image 27

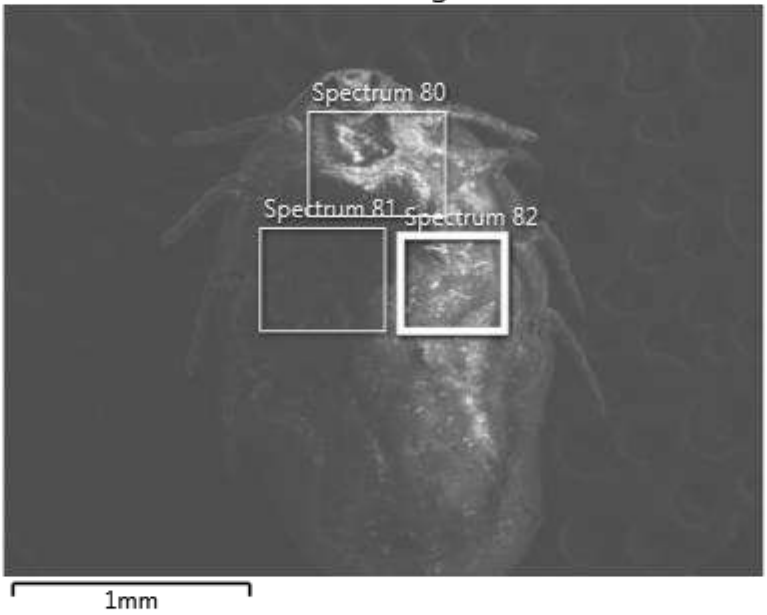

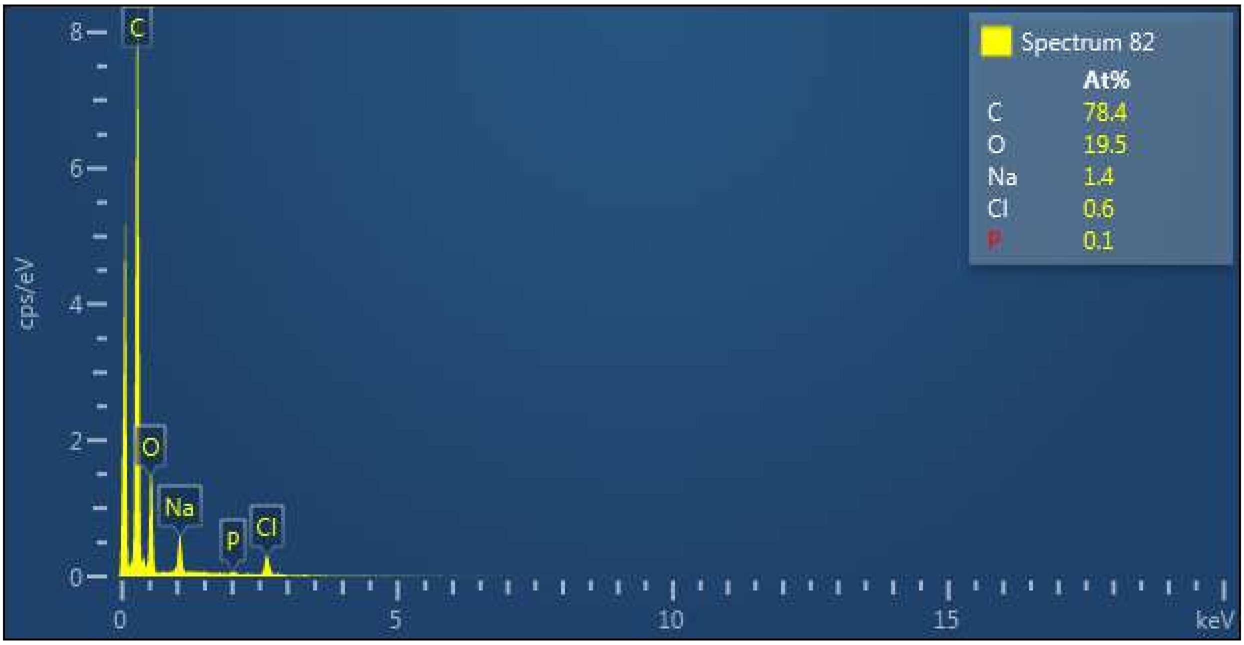

Label:	Spectrum 82
Source:	Acquired
Created:	23/01/2020 13:01:08
Livetime:	60.3s
Process Time:	4
Accelerating Voltage:	15.00kV
Magnification:	35 x
Working Distance:	10.0mm
Specimen Tilt (degrees):	0.0
Elevation (degrees):	35.0
Azimuth (degrees):	0.0
Number Of Channels:	2048
Energy Range (keV):	20 keV
Energy per Channel (eV):	10.0eV
Detector Type Id:	29
Detector Type:	X-Max
Window Type:	SATW
Pulse Pile Up Correction:	Succeeded
Primary Detector:	2617
Primary Detector Serial Number:	77871-X080

Element	Line Type	Apparent Concentration	k Ratio	Wt%	Wt% Sigma	Atomic %	Standard Label	Factory Standard	Standard Calibration Date
C	K series	13.37	0.13367	71.88	0.44	78.40	C Vit	Yes	
O	K series	4.47	0.01505	23.85	0.44	19.53	SiO2	Yes	
Na	K	0.92	0.0038	2.44	0.10	1.39	Albite	Yes	

	series		9						
P	K series	0.08	0.00043	0.17	0.04	0.07	GaP	Yes	
Cl	K series	0.49	0.00432	1.67	0.08	0.62	NaCl	Yes	
Total:				100.00		100.00			

Electron Image 27

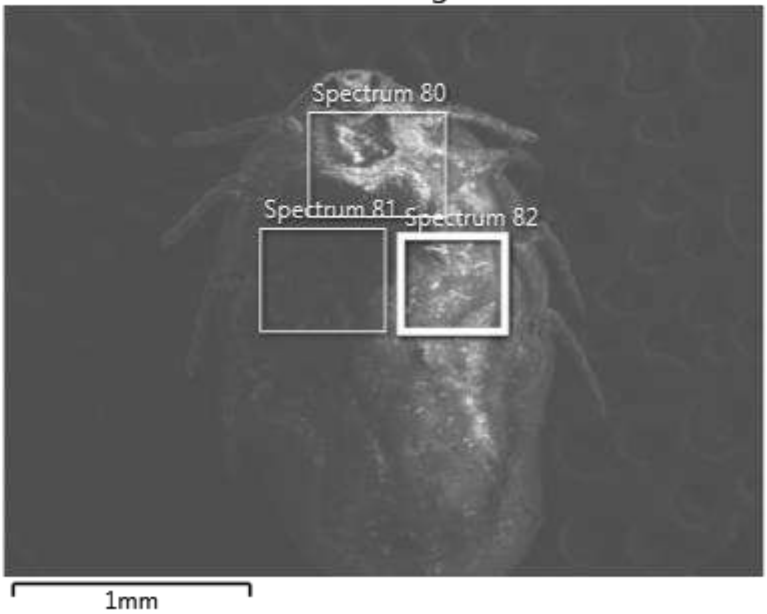

23/01/2020 13:03:06
Project 1
Rumania 9
Ventral 1 15 Kv

Electron Image 28

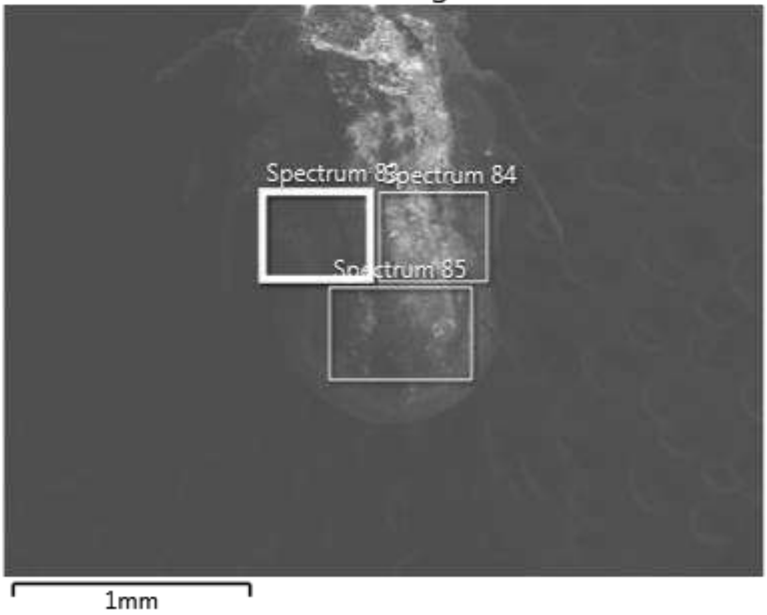

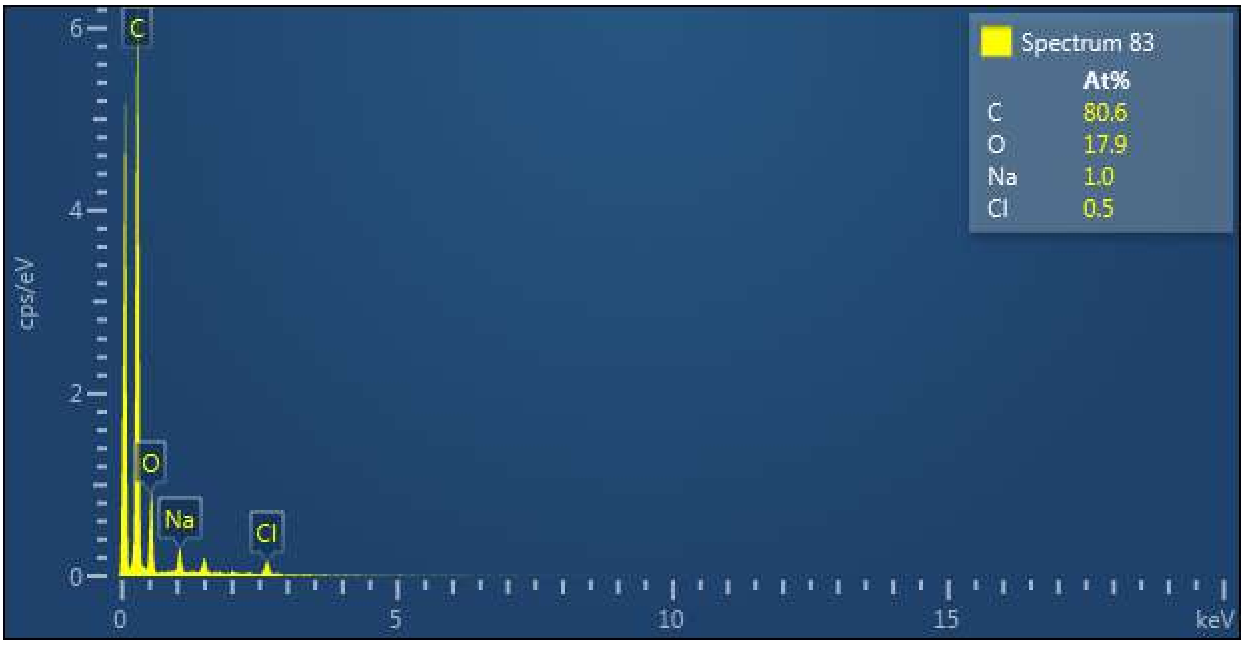

Label:	Spectrum 83
Source:	Acquired
Created:	23/01/2020 13:03:06
Livetime:	60.0s
Process Time:	4
Accelerating Voltage:	15.00kV
Magnification:	35 x
Working Distance:	10.0mm
Specimen Tilt (degrees):	0.0
Elevation (degrees):	35.0
Azimuth (degrees):	0.0
Number Of Channels:	2048
Energy Range (keV):	20 keV
Energy per Channel (eV):	10.0eV
Detector Type Id:	29
Detector Type:	X-Max
Window Type:	SATW
Pulse Pile Up Correction:	Succeeded
Primary Detector:	2617
Primary Detector Serial Number:	77871-X080

Element	Line Type	Apparent Concentration	k Ratio	Wt%	Wt% Sigma	Atomic %	Standard Label	Factory Standard	Standard Calibration Date
C	K series	9.91	0.09911	74.76	0.53	80.60	C Vit	Yes	
O	K series	2.62	0.00882	22.13	0.53	17.91	SiO2	Yes	
Na	K	0.45	0.0018	1.80	0.11	1.02	Albite	Yes	

	series		9						
Cl	K series	0.25	0.00221	1.30	0.09	0.48	NaCl	Yes	
Total:				100.00		100.00			

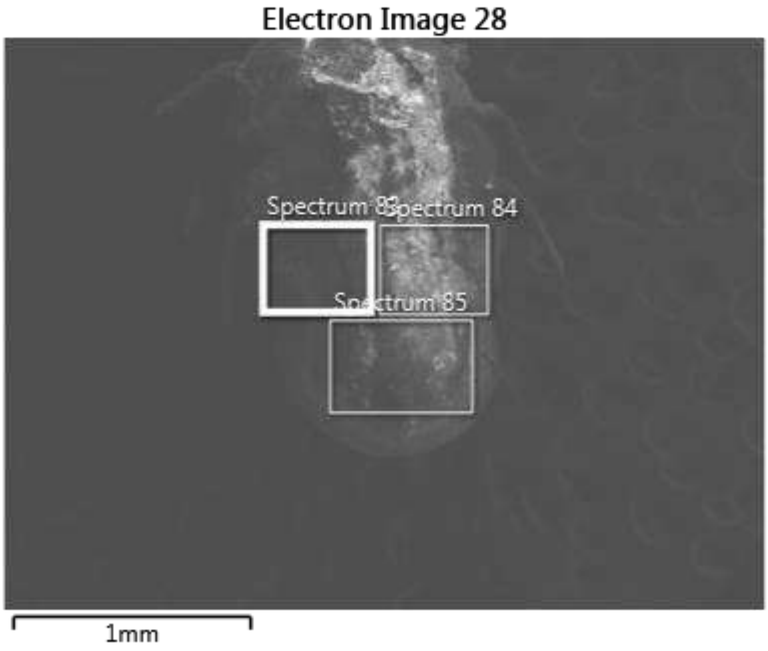

23/01/2020 13:04:17
Project 1
Rumania 9
Ventral 1 15 Kv

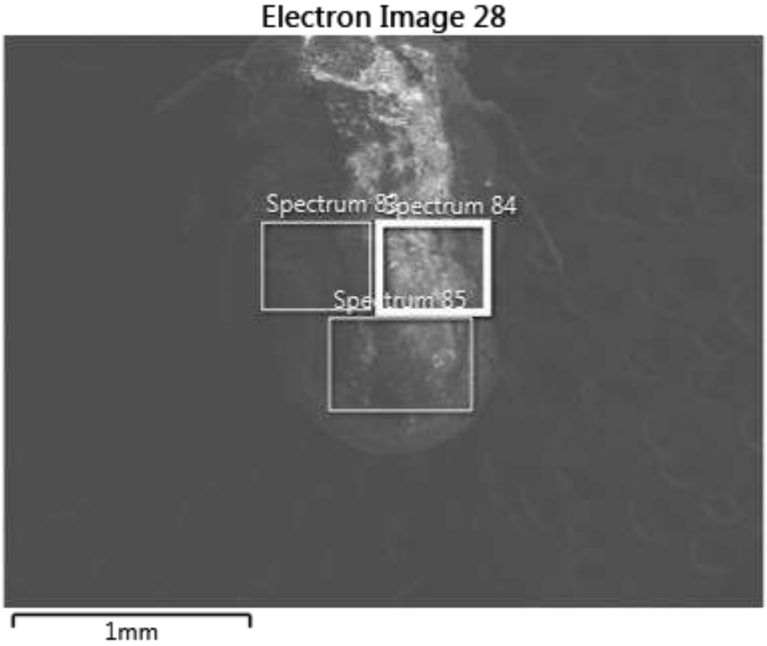

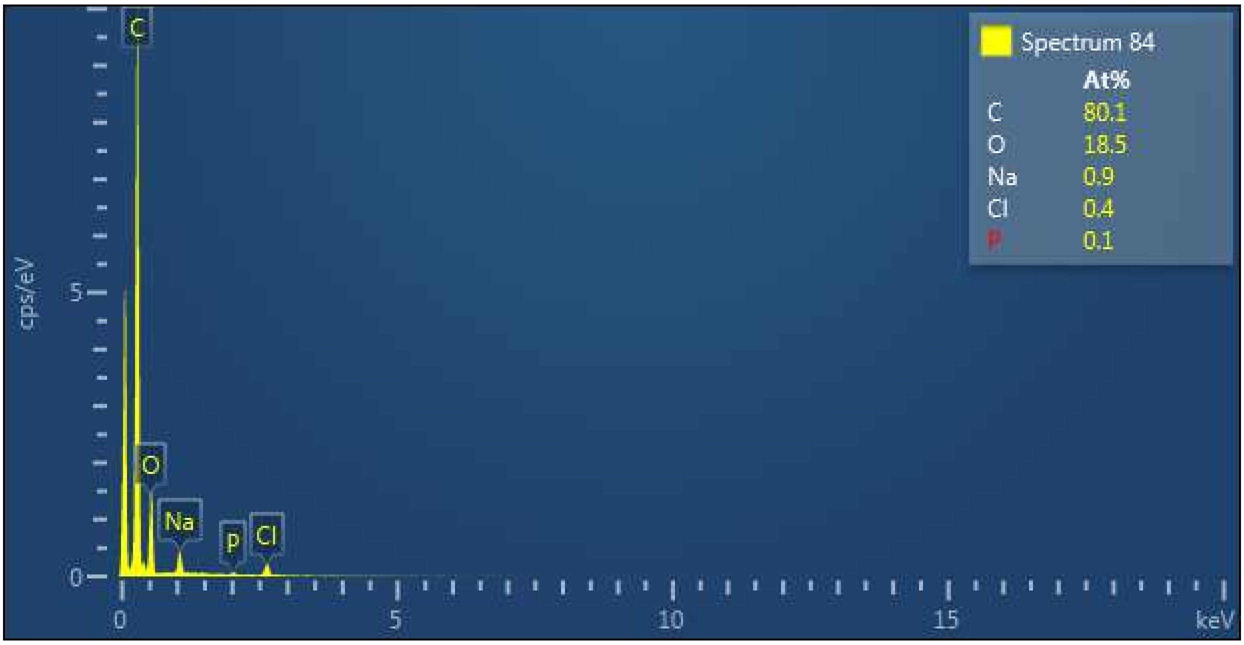

Label:	Spectrum 84
Source:	Acquired
Created:	23/01/2020 13:04:17
Livetime:	60.4s
Process Time:	4
Accelerating Voltage:	15.00kV
Magnification:	35 x
Working Distance:	10.0mm
Specimen Tilt (degrees):	0.0
Elevation (degrees):	35.0
Azimuth (degrees):	0.0
Number Of Channels:	2048
Energy Range (keV):	20 keV
Energy per Channel (eV):	10.0eV
Detector Type Id:	29
Detector Type:	X-Max
Window Type:	SATW
Pulse Pile Up Correction:	Succeeded
Primary Detector:	2617
Primary Detector Serial Number:	77871-X080

Element	Line Type	Apparent Concentration	k Ratio	Wt%	Wt% Sigma	Atomic %	Standard Label	Factory Standard	Standard Calibration Date
C	K series	15.83	0.15835	74.22	0.42	80.10	C Vit	Yes	
O	K series	4.36	0.01468	22.81	0.42	18.48	SiO2	Yes	
Na	K	0.66	0.0027	1.67	0.08	0.94	Albite	Yes	

	series		9						
P	K series	0.08	0.00046	0.17	0.04	0.07	GaP	Yes	
Cl	K series	0.35	0.00302	1.12	0.06	0.41	NaCl	Yes	
Total:				100.00		100.00			

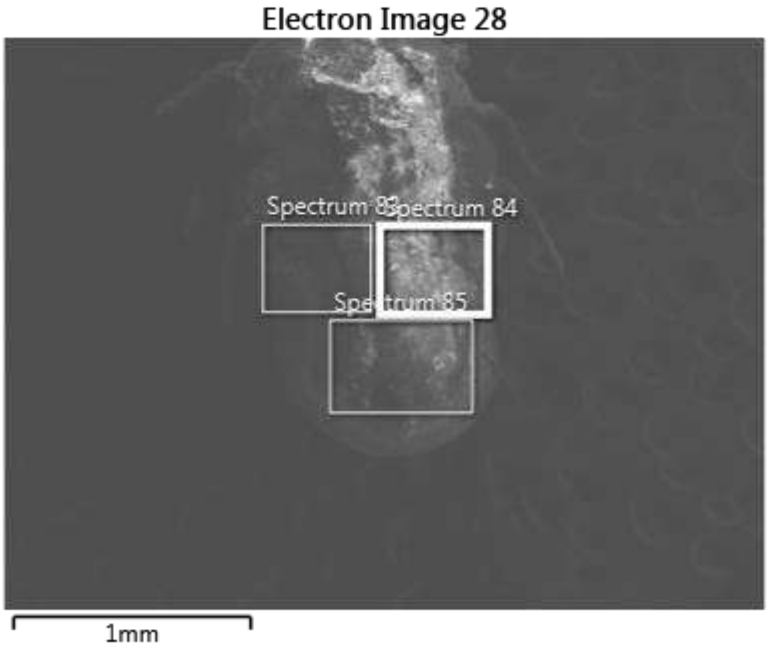

23/01/2020 13:05:30
Project 1
Rumania 9
Ventral 1 15 Kv

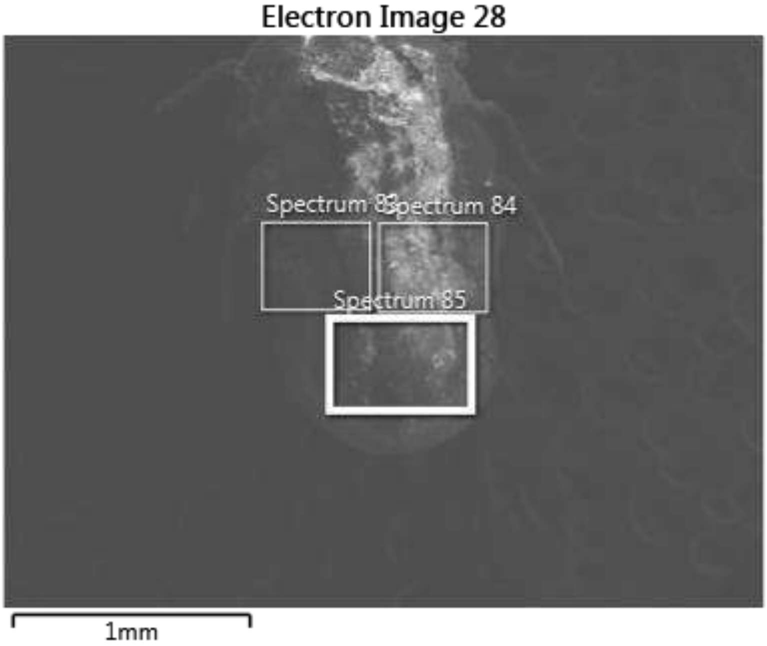

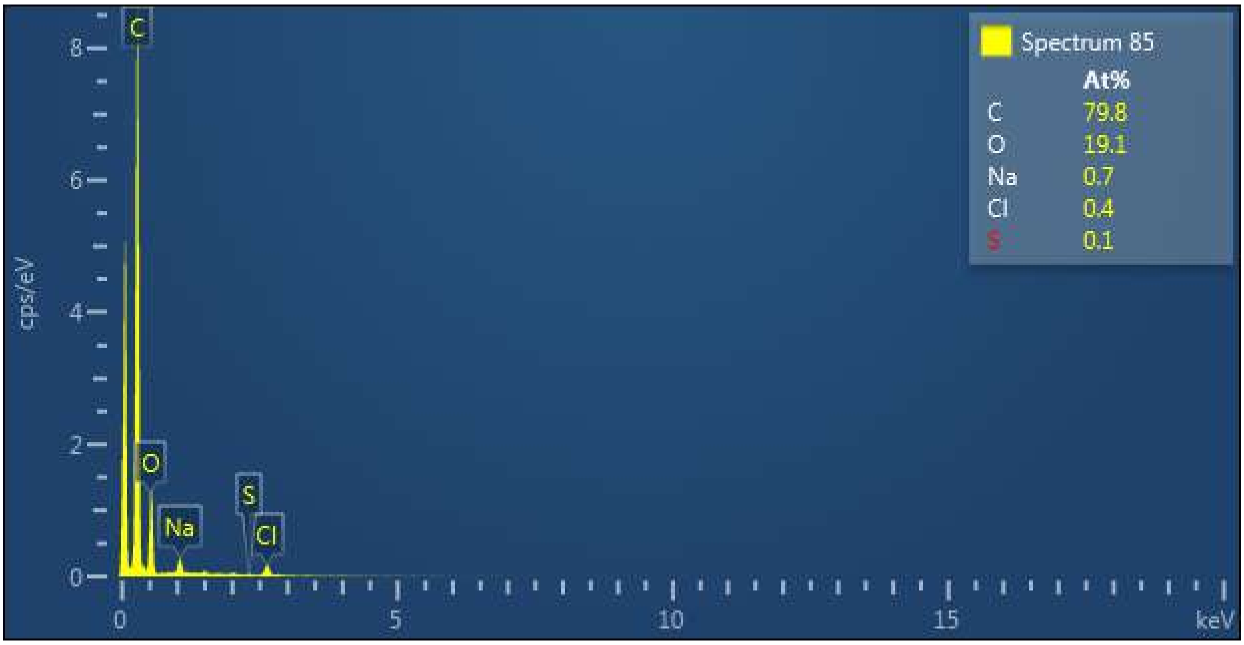

Label:	Spectrum 85
Source:	Acquired
Created:	23/01/2020 13:05:30
Livetime:	60.4s
Process Time:	4
Accelerating Voltage:	15.00kV
Magnification:	35 x
Working Distance:	10.0mm
Specimen Tilt (degrees):	0.0
Elevation (degrees):	35.0
Azimuth (degrees):	0.0
Number Of Channels:	2048
Energy Range (keV):	20 keV
Energy per Channel (eV):	10.0eV
Detector Type Id:	29
Detector Type:	X-Max
Window Type:	SATW
Pulse Pile Up Correction:	Succeeded
Primary Detector:	2617
Primary Detector Serial Number:	77871-X080

Element	Line Type	Apparent Concentration	k Ratio	Wt%	Wt% Sigma	Atomic %	Standard Label	Factory Standard	Standard Calibration Date
C	K series	13.54	0.13536	74.07	0.46	79.83	C Vit	Yes	
O	K series	3.80	0.01280	23.58	0.46	19.08	SiO2	Yes	
Na	K	0.39	0.0016	1.18	0.08	0.66	Albite	Yes	

	series		4						
S	K series	0.04	0.00032	0.14	0.04	0.06	FeS2	Yes	
Cl	K series	0.27	0.00235	1.03	0.07	0.38	NaCl	Yes	
Total:				100.00		100.00			

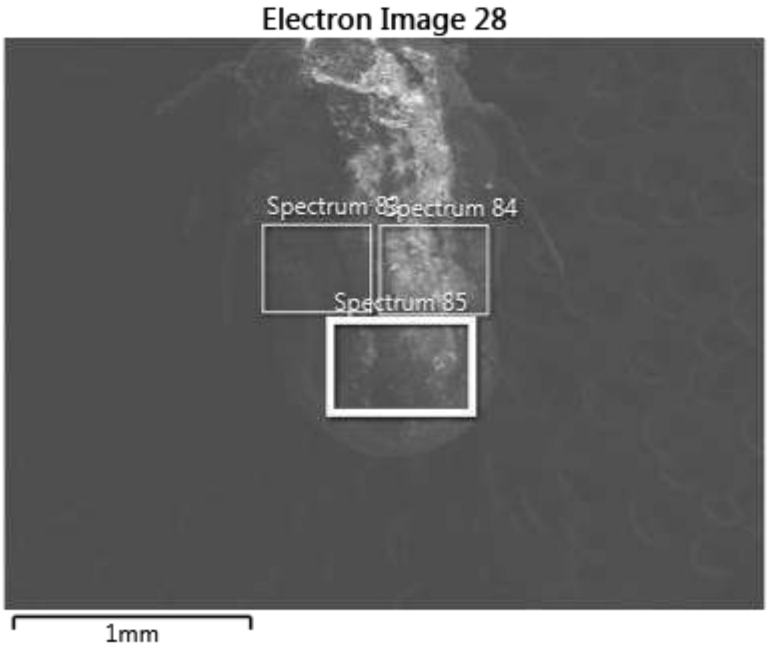

23/01/2020 13:20:00
Project 1
Rumania 10
Pecho 1 15 Kv

Electron Image 29

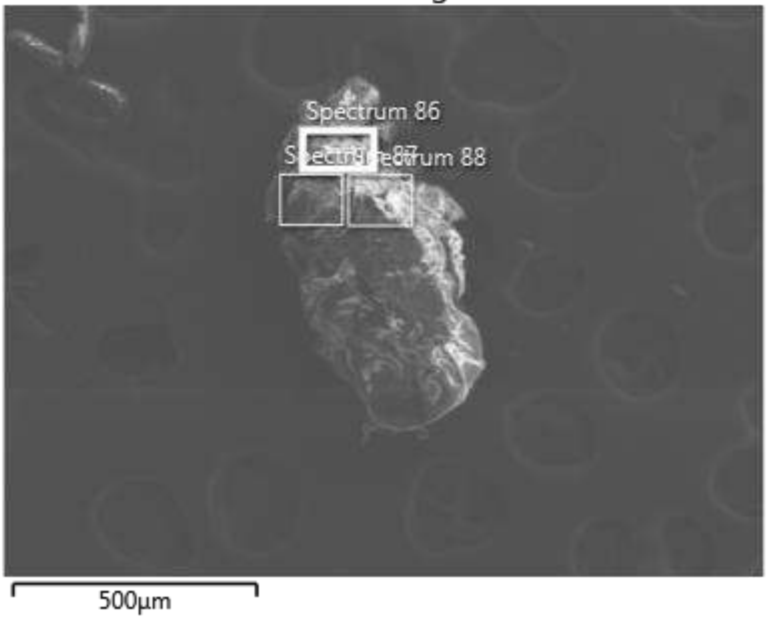

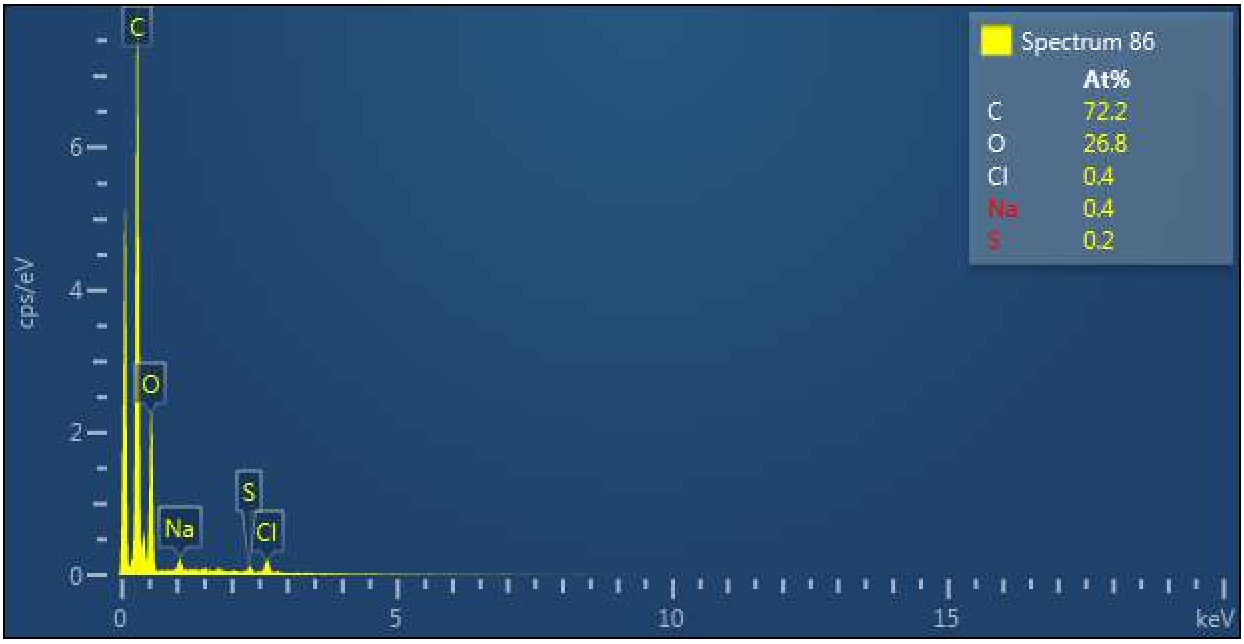

Label:	Spectrum 86
Source:	Acquired
Created:	23/01/2020 13:20:00
Livetime:	60.0s
Process Time:	4
Accelerating Voltage:	15.00kV
Magnification:	72 x
Working Distance:	10.0mm
Specimen Tilt (degrees):	0.0
Elevation (degrees):	35.0
Azimuth (degrees):	0.0
Number Of Channels:	2048
Energy Range (keV):	20 keV
Energy per Channel (eV):	10.0eV
Detector Type Id:	29
Detector Type:	X-Max
Window Type:	SATW
Pulse Pile Up Correction:	Succeeded
Primary Detector:	2617
Primary Detector Serial Number:	77871-X080

Element	Line Type	Apparent Concentration	k Ratio	Wt%	Wt% Sigma	Atomic %	Standard Label	Factory Standard	Standard Calibration Date
C	K series	12.42	0.12416	65.45	0.46	72.24	C Vit	Yes	
O	K series	6.72	0.02261	32.29	0.46	26.75	SiO2	Yes	
Na	K	0.26	0.0010	0.72	0.07	0.42	Albite	Yes	

	series		9						
S	K series	0.13	0.00113	0.42	0.05	0.18	FeS2	Yes	
Cl	K series	0.33	0.00290	1.12	0.07	0.42	NaCl	Yes	
Total:				100.00		100.00			

Electron Image 29

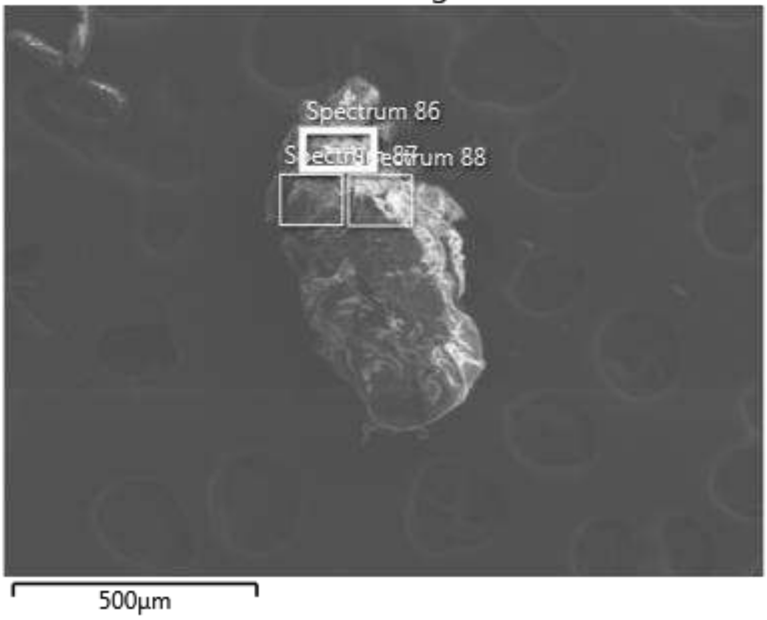

23/01/2020 13:21:11
Project 1
Rumania 10
Pecho 1 15 Kv

Electron Image 29

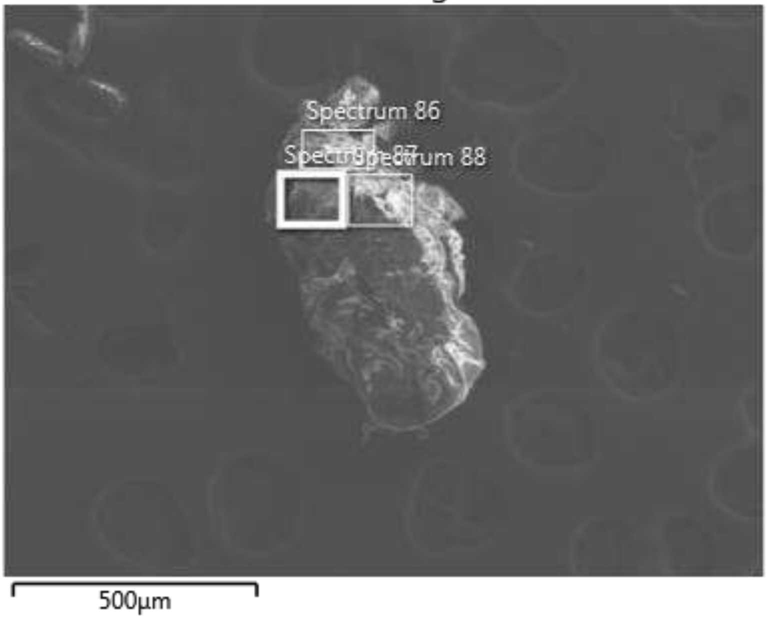

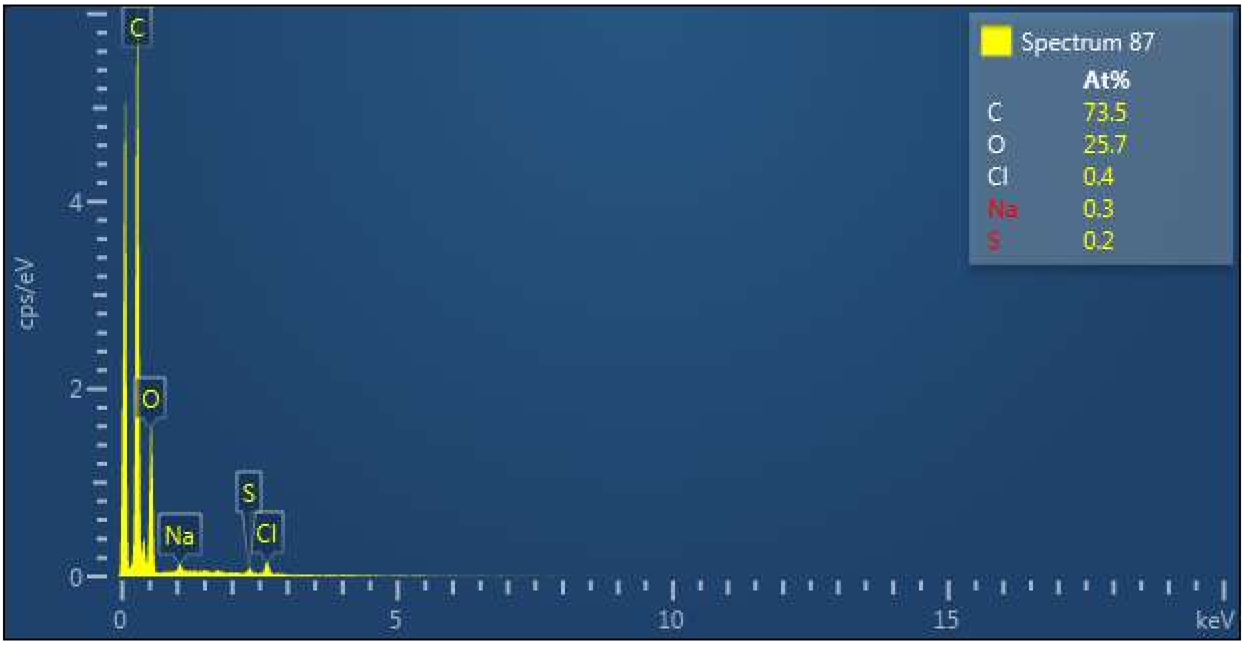

Label:	Spectrum 87
Source:	Acquired
Created:	23/01/2020 13:21:11
Livetime:	60.3s
Process Time:	4
Accelerating Voltage:	15.00kV
Magnification:	72 x
Working Distance:	10.0mm
Specimen Tilt (degrees):	0.0
Elevation (degrees):	35.0
Azimuth (degrees):	0.0
Number Of Channels:	2048
Energy Range (keV):	20 keV
Energy per Channel (eV):	10.0eV
Detector Type Id:	29
Detector Type:	X-Max
Window Type:	SATW
Pulse Pile Up Correction:	Succeeded
Primary Detector:	2617
Primary Detector Serial Number:	77871-X080

Element	Line Type	Apparent Concentration	k Ratio	Wt%	Wt% Sigma	Atomic %	Standard Label	Factory Standard	Standard Calibration Date
C	K series	9.42	0.09423	66.90	0.53	73.48	C Vit	Yes	
O	K series	4.60	0.01549	31.13	0.53	25.67	SiO2	Yes	
Na	K	0.13	0.0005	0.50	0.08	0.29	Albite	Yes	

	serie s		5						
S	K serie s	0.09	0.0007 7	0.40	0.06	0.16	FeS2	Yes	
Cl	K serie s	0.23	0.0020 2	1.08	0.08	0.40	NaCl	Yes	
Total:				100.0 0		100.0 0			

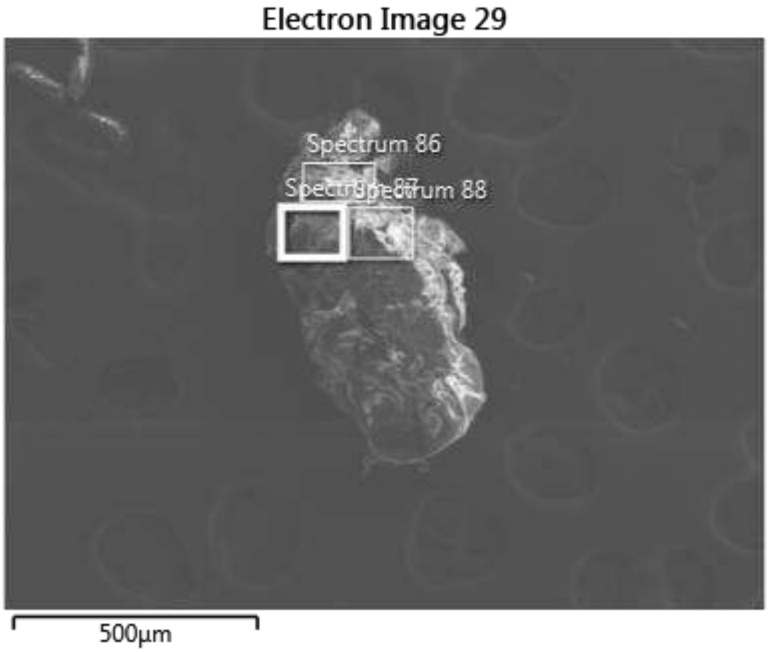

23/01/2020 13:22:22
Project 1
Rumania 10
Pecho 1 15 Kv

Electron Image 29

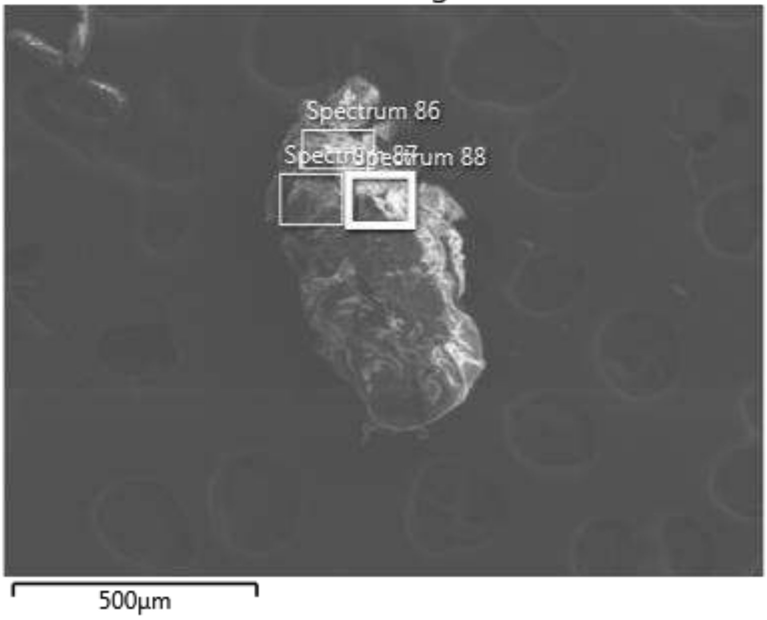

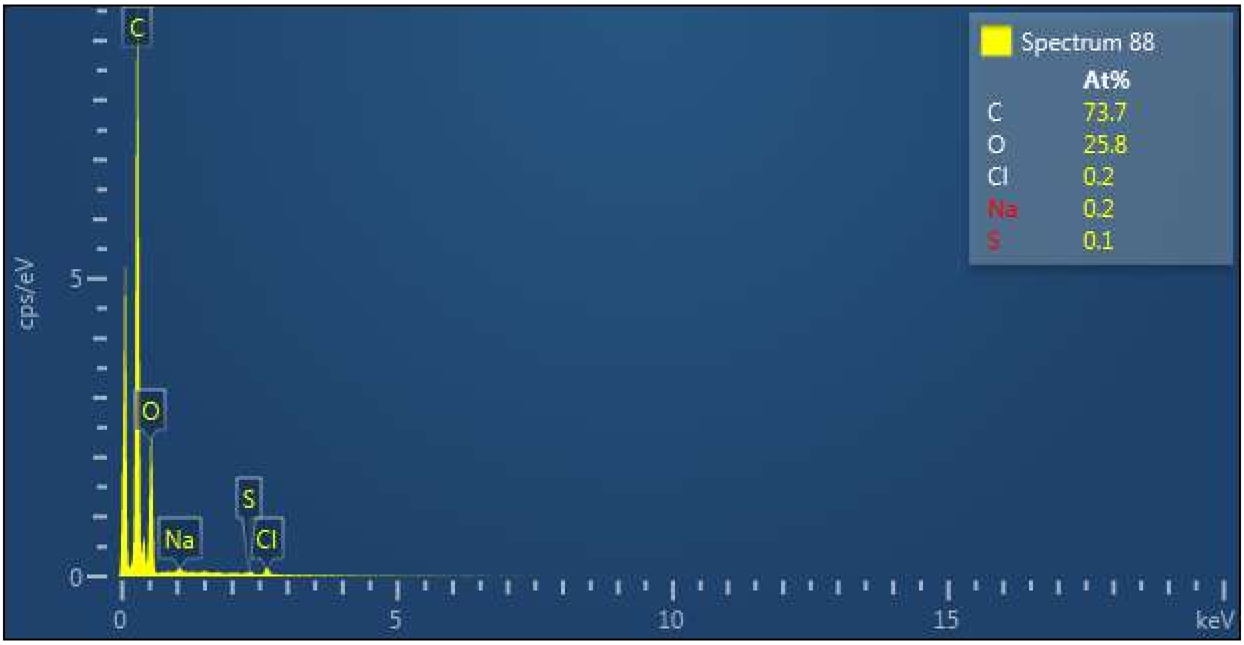

Label:	Spectrum 88
Source:	Acquired
Created:	23/01/2020 13:22:22
Livetime:	60.3s
Process Time:	4
Accelerating Voltage:	15.00kV
Magnification:	72 x
Working Distance:	10.0mm
Specimen Tilt (degrees):	0.0
Elevation (degrees):	35.0
Azimuth (degrees):	0.0
Number Of Channels:	2048
Energy Range (keV):	20 keV
Energy per Channel (eV):	10.0eV
Detector Type Id:	29
Detector Type:	X-Max
Window Type:	SATW
Pulse Pile Up Correction:	Succeeded
Primary Detector:	2617
Primary Detector Serial Number:	77871-X080

Element	Line Type	Apparent Concentration	k Ratio	Wt%	Wt% Sigma	Atomic %	Standard Label	Factory Standard	Standard Calibration Date
C	K series	14.83	0.14830	67.41	0.44	73.70	C Vit	Yes	
O	K series	6.74	0.02268	31.43	0.44	25.79	SiO2	Yes	
Na	K	0.13	0.0005	0.33	0.06	0.19	Albite	Yes	

	serie s		3						
S	K serie s	0.06	0.0005 1	0.18	0.04	0.08	FeS2	Yes	
Cl	K serie s	0.20	0.0017 5	0.64	0.05	0.24	NaCl	Yes	
Total:				100.0 0		100.0 0			

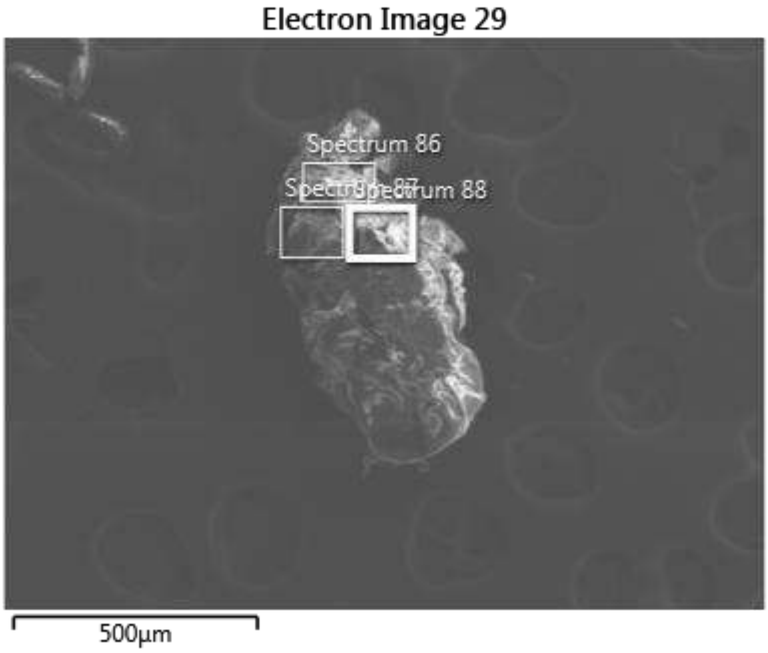

23/01/2020 13:24:03
Project 1
Rumania 10
Ventral 1 15 Kv

Electron Image 30

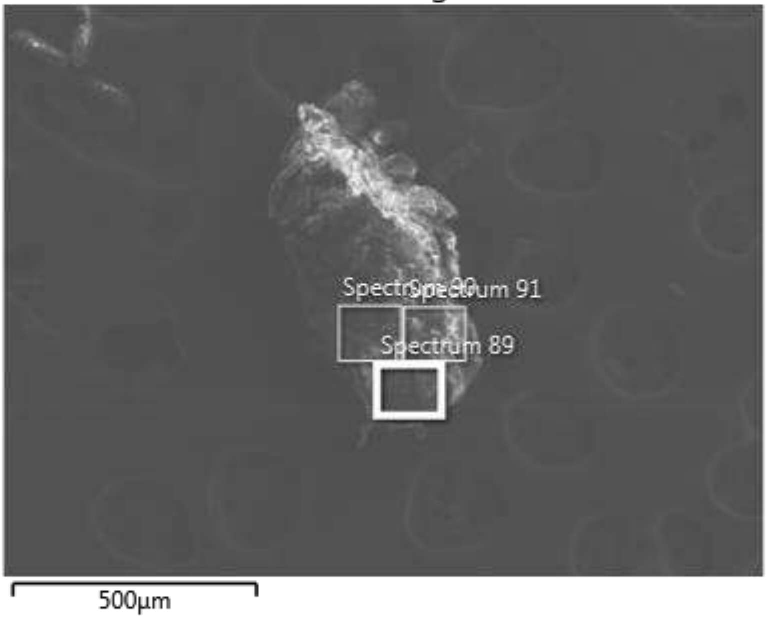

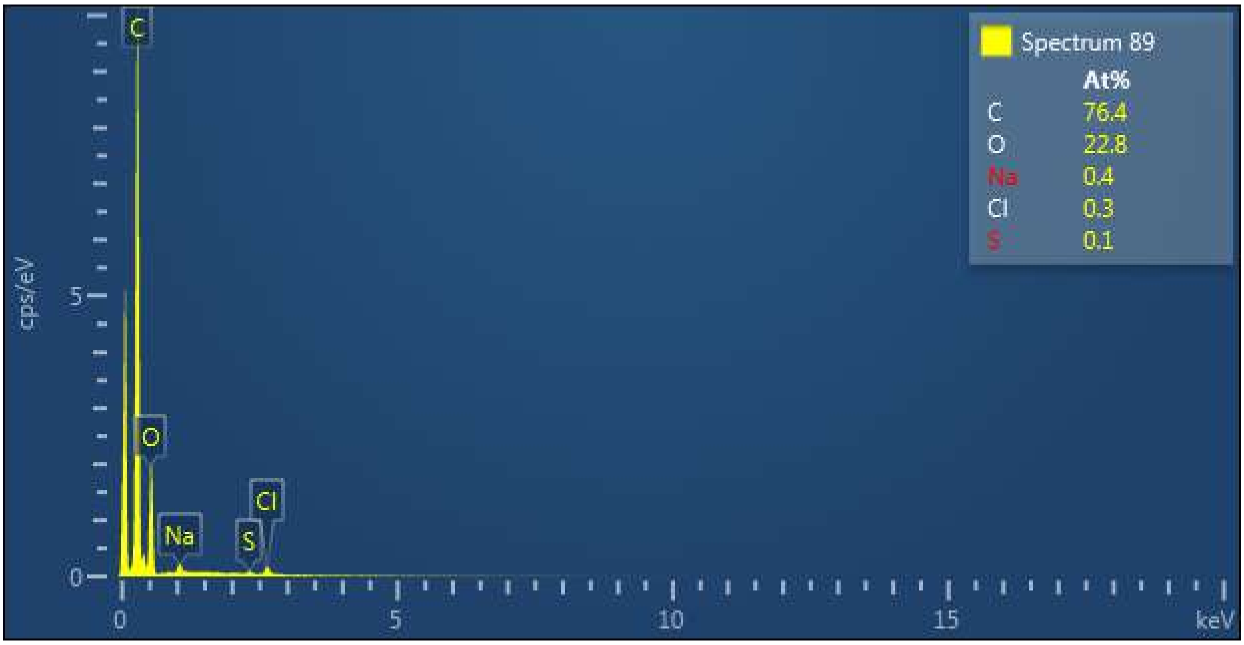

Label:	Spectrum 89
Source:	Acquired
Created:	23/01/2020 13:24:03
Livetime:	60.3s
Process Time:	4
Accelerating Voltage:	15.00kV
Magnification:	72 x
Working Distance:	10.0mm
Specimen Tilt (degrees):	0.0
Elevation (degrees):	35.0
Azimuth (degrees):	0.0
Number Of Channels:	2048
Energy Range (keV):	20 keV
Energy per Channel (eV):	10.0eV
Detector Type Id:	29
Detector Type:	X-Max
Window Type:	SATW
Pulse Pile Up Correction:	Succeeded
Primary Detector:	2617
Primary Detector Serial Number:	77871-X080

Element	Line Type	Apparent Concentration	k Ratio	Wt%	Wt% Sigma	Atomic %	Standard Label	Factory Standard	Standard Calibration Date
C	K series	16.04	0.16038	70.38	0.42	76.43	C Vit	Yes	
O	K series	5.93	0.01996	28.01	0.42	22.84	SiO2	Yes	
Na	K	0.25	0.0010	0.64	0.07	0.36	Albite	Yes	

	series		7						
S	K series	0.08	0.00072	0.25	0.05	0.10	FeS2	Yes	
Cl	K series	0.23	0.00204	0.73	0.06	0.27	NaCl	Yes	
Total:				100.00		100.00			

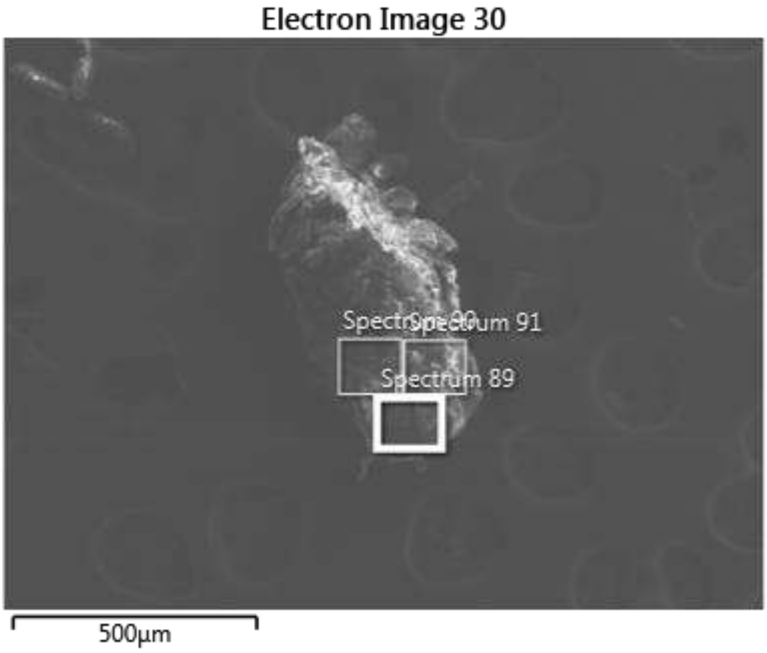

23/01/2020 13:25:14
Project 1
Rumania 10
Ventral 1 15 Kv

Electron Image 30

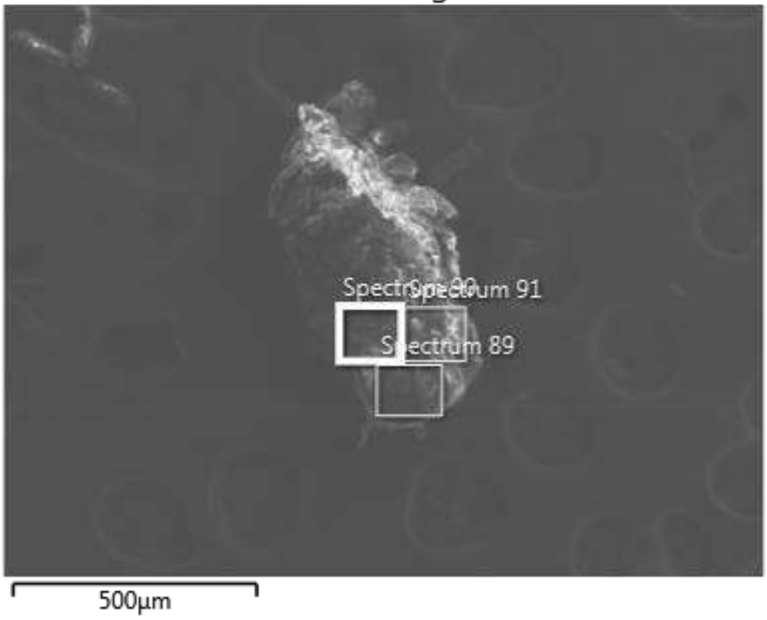

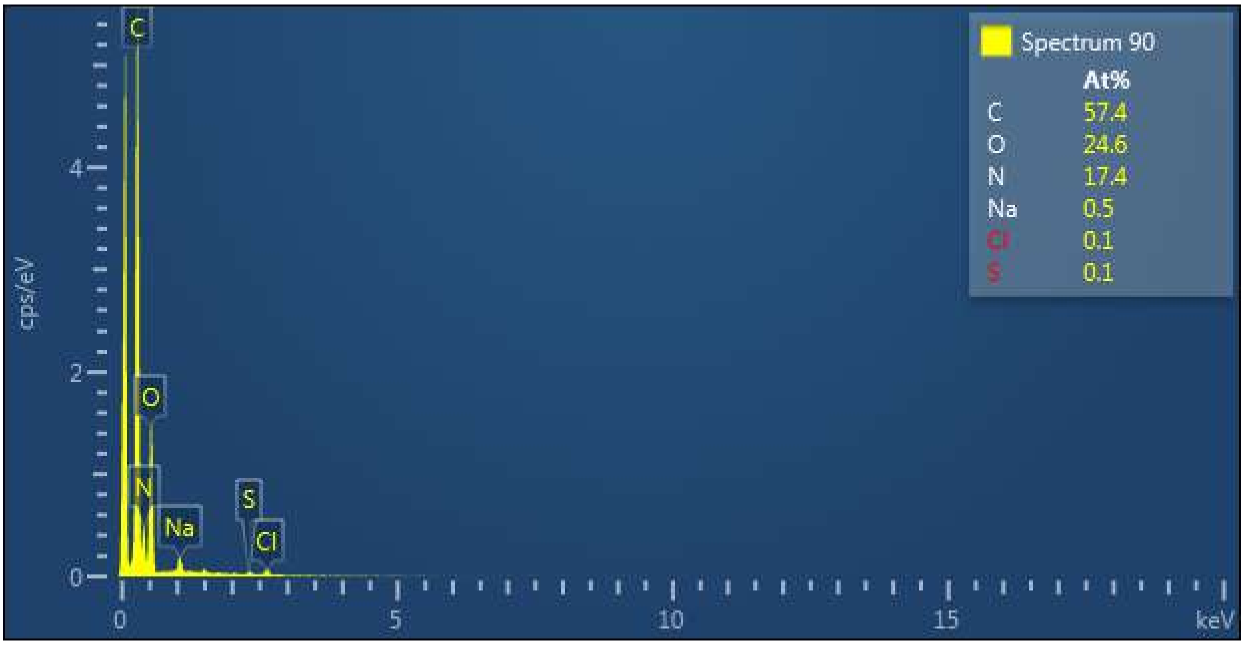

Label:	Spectrum 90
Source:	Acquired
Created:	23/01/2020 13:25:14
Livetime:	60.3s
Process Time:	4
Accelerating Voltage:	15.00kV
Magnification:	72 x
Working Distance:	10.0mm
Specimen Tilt (degrees):	0.0
Elevation (degrees):	35.0
Azimuth (degrees):	0.0
Number Of Channels:	2048
Energy Range (keV):	20 keV
Energy per Channel (eV):	10.0eV
Detector Type Id:	29
Detector Type:	X-Max
Window Type:	SATW
Pulse Pile Up Correction:	Succeeded
Primary Detector:	2617
Primary Detector Serial Number:	77871-X080

Element	Line Type	Apparent Concentration	k Ratio	Wt%	Wt% Sigma	Atomic %	Standard Label	Factory Standard	Standard Calibration Date
C	K series	8.91	0.08914	51.28	0.95	57.38	C Vit	Yes	
N	K series	3.83	0.00682	18.09	1.32	17.36	BN	Yes	
O	K	4.45	0.0149	29.23	0.68	24.55	SiO2	Yes	

	series		7						
Na	K series	0.25	0.00106	0.84	0.08	0.49	Albite	Yes	
S	K series	0.05	0.00039	0.17	0.04	0.07	FeS2	Yes	
Cl	K series	0.09	0.00083	0.38	0.05	0.14	NaCl	Yes	
Total:				100.00		100.00			

Electron Image 30

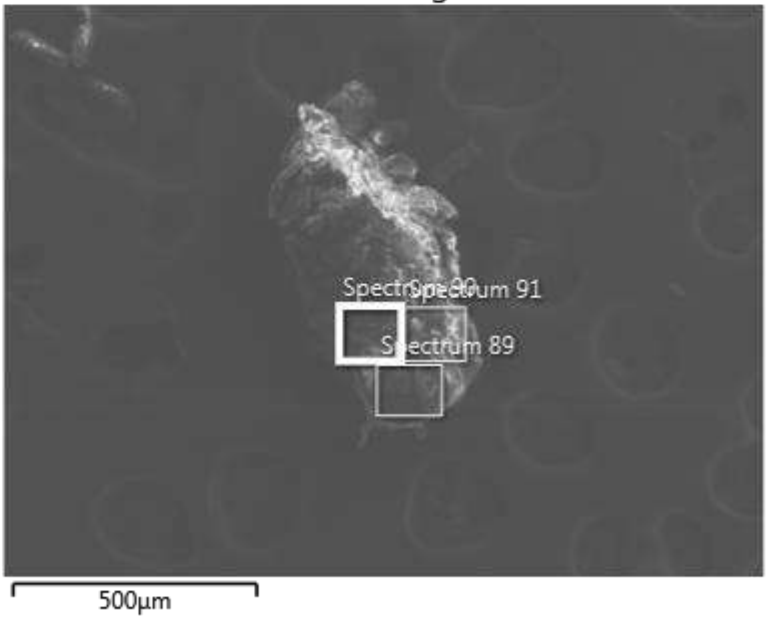

23/01/2020 13:26:26
Project 1
Rumania 10
Ventral 1 15 Kv

Electron Image 30

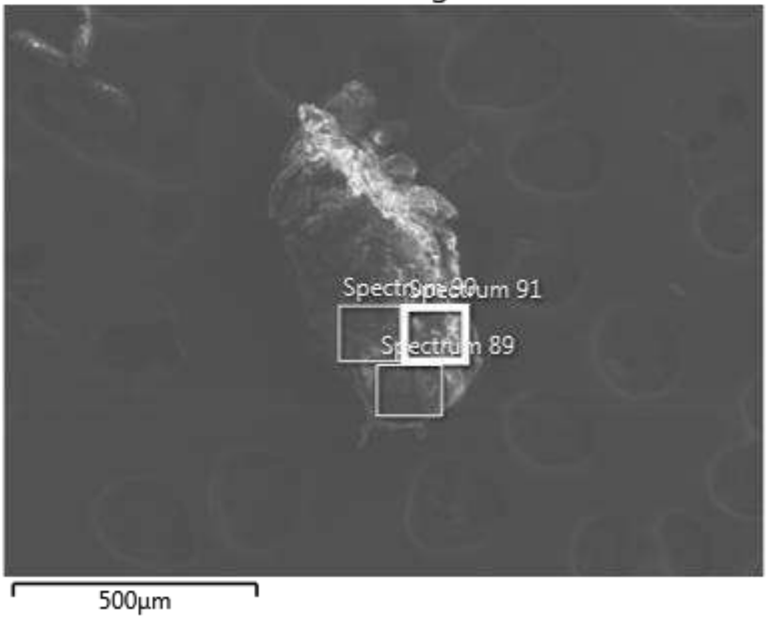

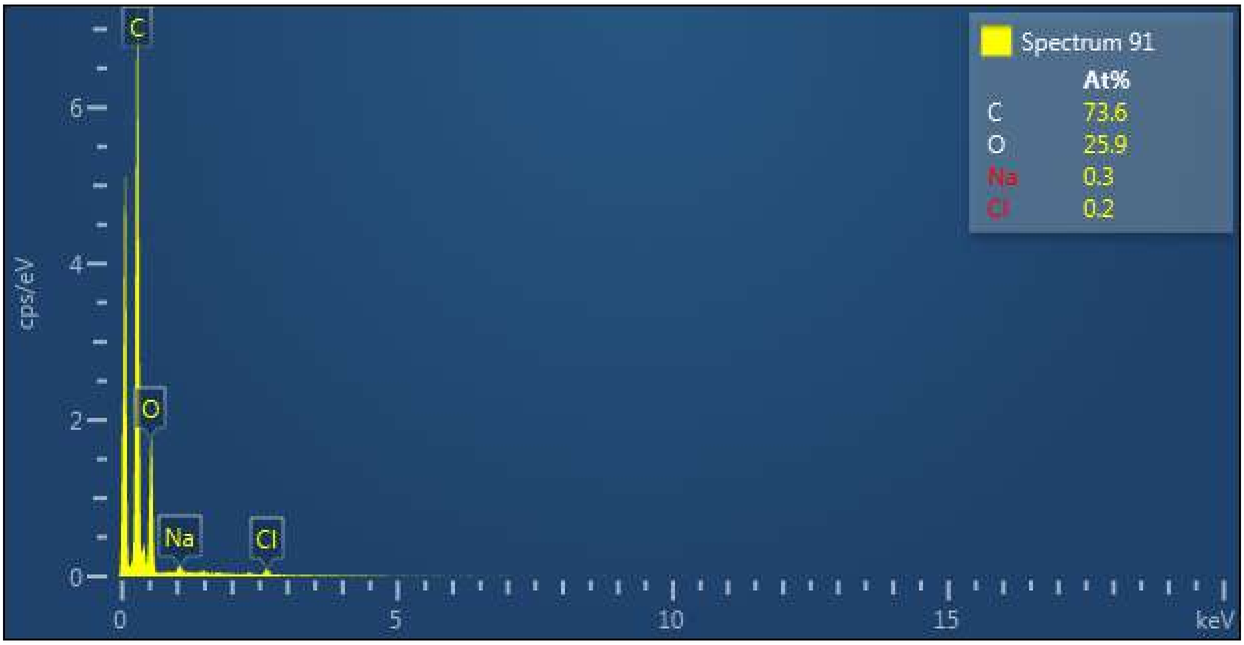

Label:	Spectrum 91
Source:	Acquired
Created:	23/01/2020 13:26:26
Livetime:	60.1s
Process Time:	4
Accelerating Voltage:	15.00kV
Magnification:	72 x
Working Distance:	10.0mm
Specimen Tilt (degrees):	0.0
Elevation (degrees):	35.0
Azimuth (degrees):	0.0
Number Of Channels:	2048
Energy Range (keV):	20 keV
Energy per Channel (eV):	10.0eV
Detector Type Id:	29
Detector Type:	X-Max
Window Type:	SATW
Pulse Pile Up Correction:	Succeeded
Primary Detector:	2617
Primary Detector Serial Number:	77871-X080

Element	Line Type	Apparent Concentration	k Ratio	Wt%	Wt% Sigma	Atomic %	Standard Label	Factory Standard	Standard Calibration Date
C	K series	11.42	0.11421	67.35	0.50	73.60	C Vit	Yes	
O	K series	5.13	0.01728	31.56	0.50	25.89	SiO2	Yes	
Na	K	0.14	0.0006	0.51	0.08	0.29	Albite	Yes	

	series		1						
Cl	K series	0.14	0.00120	0.59	0.06	0.22	NaCl	Yes	
Total:				100.00		100.00			

Electron Image 30

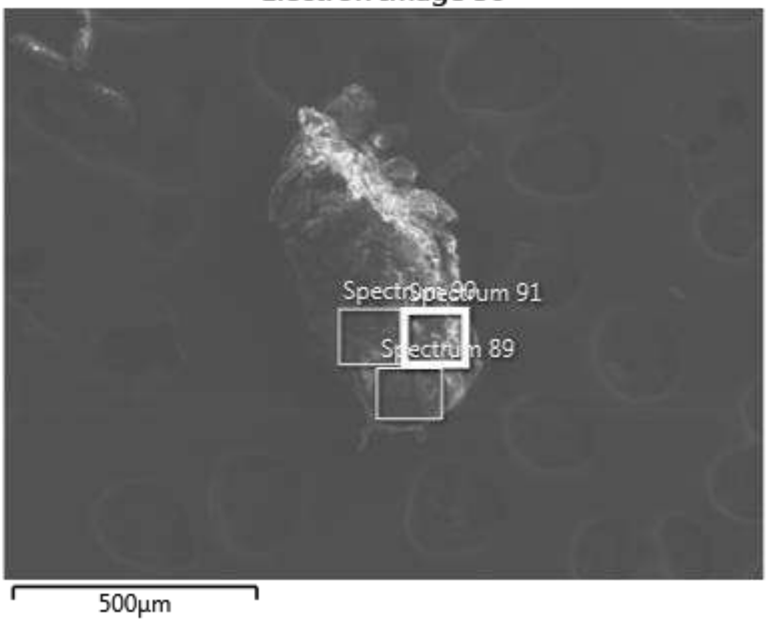

Supplement: DATA S2 — Quantitative spectra of chemical elements of tick exoskeleton. [file Data_Sheet_2.PDF]
